# Specific quinone reductase 2 inhibitors reduce metabolic burden and reverse Alzheimer’s disease phenotype in mice

**DOI:** 10.1172/JCI162120

**Published:** 2023-10-02

**Authors:** Nathaniel L. Gould, Gila R. Scherer, Silvia Carvalho, Khriesto Shurrush, Haneen Kayyal, Efrat Edry, Alina Elkobi, Orit David, Maria Foqara, Darshit Thakar, Tommaso Pavesi, Vijendra Sharma, Matthew Walker, Matthew Maitland, Orly Dym, Shira Albeck, Yoav Peleg, Nicolas Germain, Ilana Babaev, Haleli Sharir, Maya Lalzar, Boris Shklyar, Neta Hazut, Mohammad Khamaisy, Maxime Lévesque, Gilles Lajoie, Massimo Avoli, Gabriel Amitai, Bruce Lefker, Chakrapani Subramanyam, Brian Shilton, Haim Barr, Kobi Rosenblum

**Affiliations:** 1 Sagol Department of Neurobiology, University of Haifa, Haifa, Israel.; 2 Wohl Institute for Drug Discovery of the Nancy and Stephen Grand Israeli National Center for Personalized Medicine (GINCPM), Weizmann Institute of Science, Rehovot, Israel.; 3 The Centre for Genetic Manipulation in the Brain, University of Haifa, Haifa, Israel.; 4 Department of Biomedical Sciences, University of Windsor, Windsor, Ontario, Canada.; 5 Department of Biochemistry, The University of Western Ontario, London, Ontario, Canada.; 6 Life Sciences Core Facilities, Weizmann Institute of Science, Rehovot, Israel.; 7 Bioinformatics Service Unit and; 8 Bioimaging Unit, Faculty of Natural Sciences, University of Haifa, Haifa, Israel.; 9 Montreal Neurological Institute-Hospital and Department of Neurology and Neurosurgery, McGill University, Montreal, Quebec, Canada.

**Keywords:** Neuroscience, Neurodegeneration, Signal transduction

## Abstract

Biological aging can be described as accumulative, prolonged metabolic stress and is the major risk factor for cognitive decline and Alzheimer’s disease (AD). Recently, we identified and described a quinone reductase 2 (QR2) pathway in the brain, in which QR2 acts as a removable memory constraint and metabolic buffer within neurons. QR2 becomes overexpressed with age, and it is possibly a novel contributing factor to age-related metabolic stress and cognitive deficit. We found that, in human cells, genetic removal of QR2 produced a shift in the proteome opposing that found in AD brains while simultaneously reducing oxidative stress. We therefore created highly specific QR2 inhibitors (QR2is) to enable evaluation of chronic QR2 inhibition as a means to reduce biological age–related metabolic stress and cognitive decline. QR2is replicated results obtained by genetic removal of QR2, while local QR2i microinjection improved hippocampal and cortical-dependent learning in rats and mice. Continuous consumption of QR2is in drinking water improved cognition and reduced pathology in the brains of AD-model mice (5xFAD), with a noticeable between-sex effect on treatment duration. These results demonstrate the importance of QR2 activity and pathway function in the healthy and neurodegenerative brain and what we believe to be the great therapeutic potential of QR2is as first-in-class drugs.

## Graphical abstract

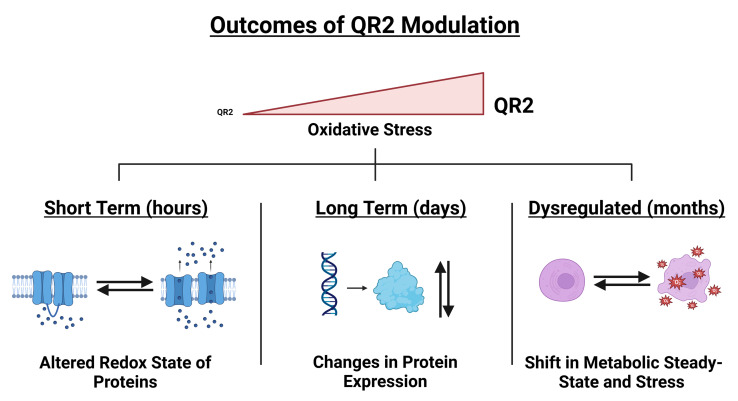

## Introduction

Accumulating metabolic dysfunction in the ageing brain creates chronic stress, which disrupts homeostasis and results in a wide spectrum of pathologies that risk the occurrence of dementia, such as Alzheimer’s disease (AD) (1, 2). Considerable efforts to address this have been aimed at the integrated stress response (ISR) pathway (3), which allows neurons to react to metabolic perturbations and restore homeostasis. Targeting the ISR also reverses cognitive deficits in rodent models (4, 5), and is therefore a prime subject of drug development (2). However, an age-related metabolic risk factor called quinone reductase 2 (QR2 or NQO2) was recently found to act in the brain (6) as part of a previously unknown pathway involved in memory formation, possibly contributing both to metabolic stress and memory decline (7, 8, 9). In this QR2 pathway, salient experiences induce dopamine (DA) or acetylcholine (ACh) release, depending on the type of information and brain structure involved (6, 7), which drive an increase in micro-RNA-182 (miR-182) levels, which, in turn, transiently reduce QR2 expression (6, 7). The result of this QR2 pathway activation is enhanced memory formation for first time (10, 11) experiences, allowing important events to stand out from continuously perceived familiar or trivial information (7). Critically, elements within this pathway that are upstream to QR2 removal, among other things, are lost or dysregulated with age, including DA, ACh, and miR-182 (12, 13). This is also true for melatonin, an endogenous and nonspecific inhibitor of QR2, a function that is thought to convey some of the antioxidant properties of melatonin (14). Loss of these QR2-controlling elements is injurious two-fold — first, the removal of QR2 from interneurons is critical for novel memory formation and this process is lost (7); and, second, chronically elevated levels of QR2 cause oxidative/metabolic stress (6, 8, 15). Importantly, in a rodent model of scopolamine-induced amnesia this pathway is blocked and QR2 levels remain high (16). By directly inhibiting QR2 in this model, memory deficits are reversed (6). Due to these findings, the fact that QR2 KO–model mice are viable (17), and the limited knowledge about downstream QR2 effects, we screened for changes that occur on the proteome level in human cells in which QR2 was selectively removed. In order to compare the chronic effects of high-level versus low-level QR2 expression, we used HCT116 cells that express high levels of QR2 (18), in which we carried out QR2 CRISPRi (19). We found that QR2 removal caused a shift in protein expression, including increases in mitochondrial mRNA translation and oxidative phosphorylation proteins, mRNA transcription and translation machinery, and reduction in cell-cell junction and cell-matrix interaction proteins and pyruvate kinase M1/2 (PKM), among others. Since these results are remarkably opposed to changes found in AD brains (20) and taking into account that QR2 is a removable memory constraint in rodents, we hypothesized that by selectively inhibiting QR2, age-related metabolic stress and cognitive decline may simultaneously be tackled, in parallel and in addition to ISR- and other existing therapeutic targets (2, 3, 21).

QR2 inhibitors (QR2is) have previously been made, in efforts to develop anti-malarial drugs, research melatonin, cancer, and basic biochemistry (22–24). However, even though QR2 has only one other closely related enzyme, QR1 (NQO1) (25), making it a comparatively simpler target to inhibit specifically, the existing inhibitors for QR2 remain either nonspecific, insoluble/nonbioavailable, or toxic, thus limiting even the current gold-standard inhibitor, S29434 (26), which we have previously used (6, 16). S29434 was originally made in efforts to create melatonin receptor ligands, requires problematic formulation (16), and concentrations required in systemic administration can inhibit QR1 and bind melatonin receptors (26).

We therefore created small molecule QR2is that enable safe and effective evaluation of chronic QR2 inhibition in vitro and in vivo as a way to reduce biological age–related metabolic stress and cognitive decline. We carried out a high throughput screen (HTS) to assay approximately 200,000 compounds against QR2 activity. Following concentration-response hit validation using standard and orthogonal assays against QR2 and QR1, a series of diverse sulfonamides were identified and selected as a lead series. Structure-activity-relationship (SAR) studies of the sulfonamide series’ effective chemical domains resulted in a family of specific QR2is, with potential for further expansion. These inhibitors possess high selectivity and potency against QR2, while showing extremely low toxicity. A water-soluble version of the most potent QR2i was used to obtain a crystal structure, providing insight into the unique interactions these inhibitors make with QR2. By using these QR2is, we hypothesize that (a) cells expressing QR2, such as neural or HCT116 cells, will display lowered metabolic stress, (b) rodents will demonstrate better cognitive abilities and (c) a profound AD mouse model (5XFAD) (27), at an advanced age, will show a marked improvement in cognition and reduction in brain pathology, as afforded by the reduction in QR2-mediated metabolic stress. Failure to reject these hypotheses will, in our opinion, place QR2 as a genuine first-in-class drug target to tackle age-related metabolic stress in the brain (1) and other organs (28), and help delay onset of AD symptoms.

## Results

### QR2 KO in a human cell line induces functional proteomic changes antagonistic to that of the cortex of patients with AD.

**Figure 1:**
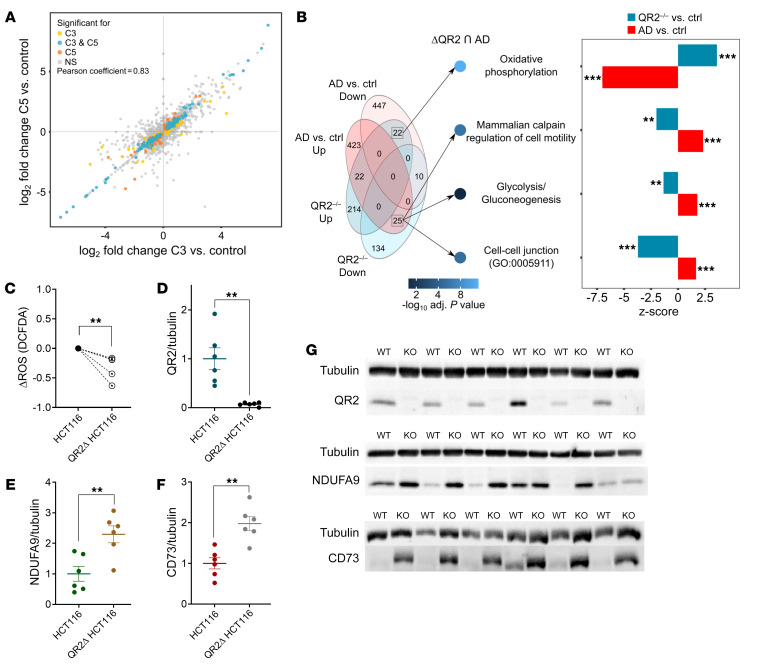
QR2 KO in a human cell line induces functional proteomic changes antagonistic to that of the cortex of patients with Alzheimer’s disease. (**A**) Two independent QR2Δ HCT cell lines (C3 and C5) show similar patterns of changes in protein expression compared with control cell lines (NS) with significant correlation (Pearson *r* = 0.83, *P* < 0.0001). (**B**) Overlap between differentially expressed proteins from the current study and from DLPFC tissues of patients with AD compared with controls, reported in Johnson et al., (20). Left: Venn diagram presenting numbers of overlapping proteins. Middle: Significance of enrichment of 4 functional categories representing contrasting effects of QR2 KO and AD. Right: z-score and enrichment significance for the same 4 functional categories within each of the QR2Δ cell lines and AD sets separately. Enrichment was considered significant for FDR adjusted *P* value <0.05. (**C**) QR2 KO in HCT116 cells significantly lowers baseline ROS levels (*n* = 5 per group; unpaired *t* test, *P* = 0.0078). (**D**) Ablation of QR2 in QR2Δ HCT116 confirm QR2 KO (*n* = 6 per group; unpaired *t* test, *P* = 0.0019). (**E**) QR2 KO significantly increases NDUFA9 levels (*n* = 6 per group; unpaired *t* test, *P* = 0.0055). (**F**) QR2 KO significantly increases CD73 levels (*n* = 6 per group; unpaired *t* test, *P* = 0.0012). (**G**) Immunoblot images of QR2, NDUFA9, CD73, and tubulin from QR2Δ HCT116 cells and isogenic controls. Unless stated otherwise, data are shown as mean ± SEM; **P < 0.01; ***P < 0.001.

There is evidence of QR2-mediated metabolic stress in human cells (6, 8, 15). However, it is unknown how QR2 activity may affect the proteome in response to the chronic stress it generates. We therefore used CRISPR-mediated QR2 KO in HCT116 cells (QR2Δ cells; available in Supplemental Methods; supplemental material available online with this article; https://doi.org/10.1172/JCI162120DS1). Proteomic analysis (Supplemental Methods) showed that the QR2Δ cell lines C3 and C5 produced highly similar results when compared with the isogenic control cell line, C1 (Figure 1A and Supplemental Data File 1A). The comparison provided several functional groups of proteins that differed significantly following QR2 KO (Supplemental Data File 1B). QR2 KO affected the expression of mitochondrial proteins (Supplemental Figure 1A), including increased expression of mitochondrial mRNA translation and oxidative phosphorylation proteins. Contrastingly, proteins involved in glycolysis and the pentose phosphate pathway had decreased expression in QR2Δ cells. Other proteins with increased expression in QR2Δ cells were related to mRNA transcription and translation. Many other QR2Δ-downregulated proteins were involved in cell-cell junction and cell-matrix interactions (Supplemental Figure 1B). Since impairment of oxidative phosphorylation and cell-cell interactions are among the leading processes involved in AD, we sought to compare the changes found in the proteome of QR2Δ versus control to the changes found in AD versus control(20). A distinct antagonistic profile is seen between QR2Δ and AD (Figure 1B and Supplemental Data File 1C), with oxidative phosphorylation–related proteins being the dominant group with contrasting effects between QR2Δ and AD. Therefore, the gene-set enrichment information found here points to a possible contribution of QR2 to the AD phenotype. In agreement with previous QR2 interference studies, a significant reduction in cellular ROS levels is seen in the QR2Δ cells compared with the isogenic controls (Figure 1C), and QR2 was not detected by immunoblot in the expected band in the QR2Δ cells (Figure 1D). In order to validate the results found in the proteomic analysis, 2 targets corresponding to some of the functional groups identified were chosen based on the availability of reliable antibodies, including NDUFA9 and CD73. We found that, in agreement with the liquid chromatography/mass spectrometry (LC-MS) results obtained, both NDUFA9 (Figure 1E) and CD73 (Figure 1F) were significantly increased in QR2Δ cells (Figure 1G). Since genetic deletion of QR2 provides a proteomic profile antagonistic to AD and causes a reduction in metabolic stress, and since QR2 has been previously linked with AD and cognitive functions (6, 16, 17, 29, 30), it follows that QR2 inhibition is an attractive and, to our knowledge, novel candidate for neurodegenerative disease treatment in general and for AD drug development specifically.

### HTS-identified sulfonamide compounds further developed by SAR provide highly potent, selective, and soluble QR2 inhibitors.

**Figure 2:**
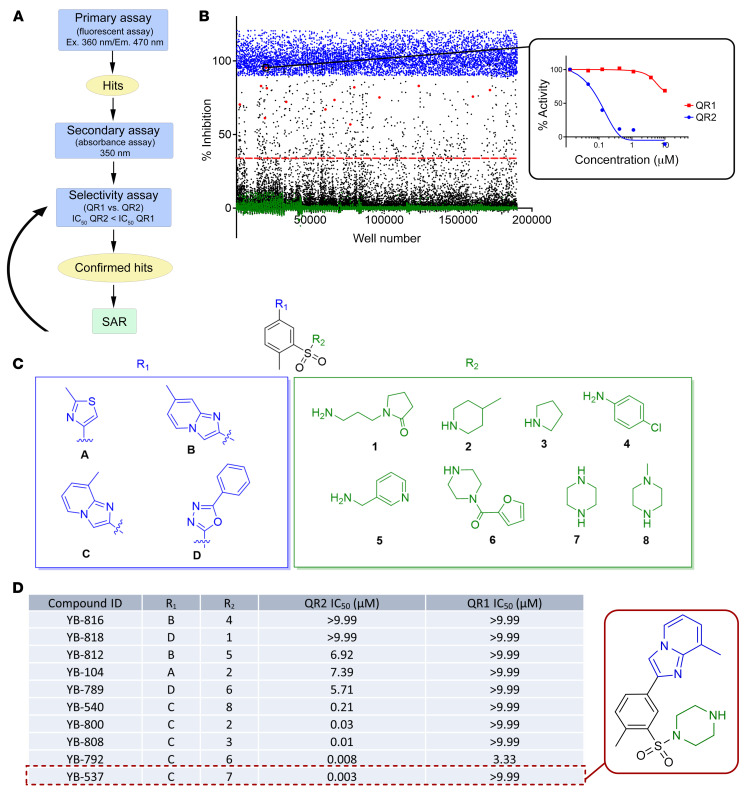
HTS-identified sulfonamide compounds further developed by SAR provide highly potent, selective, and soluble QR2i. (**A**) Procedural overview of compound screening and inhibitor development. (**B**) A QR2 activity assay based HTS against approximately 200,000 compounds was carried out, followed by concentration-response validation of hits (≥ 30% inhibition), using both primary and orthogonal assays. This was also carried out with the closely related QR1. Blue circles, inhibitor control; green circles, neutral control; black circles, compounds; red circles, sulfonamide series; red dotted line, 30% inhibition threshold. (**C**) A series of promising hits characterized by a sulfonamide (top, black) were identified, having active heterocycle (left, blue) and amine (right, green) groups that were amenable to SAR. (**D**) Comparative SAR evaluation of the new sulfonamide inhibitors using different analogues and structural combinations.

A drug discovery campaign strategy was initiated to screen compounds for QR2-specific, cell-free inhibition and in vitro activity, followed by SAR optimization (Figure 2A) to achieve highly specific and potent QR2 inhibitor synthesis. In order to establish a varied and chemically amenable starting point, we developed a high-throughput QR2 assay (Supplemental Methods) that we screened against a library with approximately 200,000 compounds. Compounds with at least 30% inhibition in the primary assay were then validated using a concentration response in both the standard assay — using dihydro-benzylnicotinamide (BNAH) cofactor fluorescence decay as a readout — as well as an orthogonal assay — in which BNAH absorption was measured instead (Supplemental Methods). Compounds that replicated the initial hit result were then assayed similarly against QR1 to evaluate compound specificity (Figure 2B). From the HTS, a chemically tractable and attractive series of compounds containing a sulfone flanked by an amine and a heterocycle were identified and selected for SAR development (Figure 2C, Supplemental Methods, Supplemental Table 1, and Supplemental Table 2). Analysis of the SAR data revealed that the imidazo[1,2-a]pyridine heterocycles gave high activity of the sulfonamide and proved to be more potent compared with other heterocycles that were evaluated (Figure 2D), forming crucial Pi-Pi interactions with the protein, attributing to its potency, as was later found in the resolved crystal structure. Thus, several highly selective and potent QR2is were made, allowing breadth and scope for future modifications and further improvement.

### QR2is bind target in vitro and reproduce QR2-KO results in isogenic controls.

**Figure 3:**
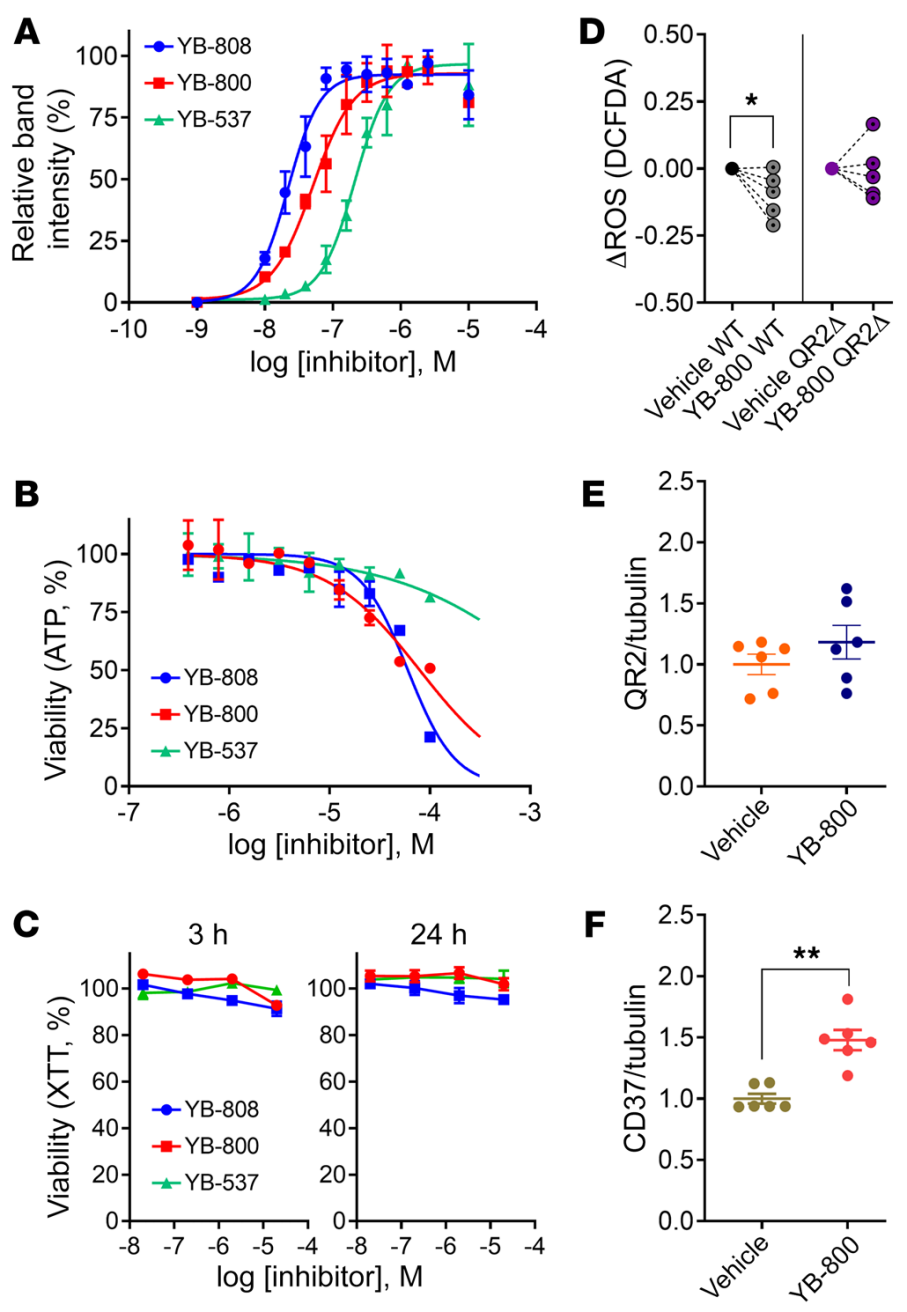
QR2is bind target in vitro and reproduce QR2-KO results in isogenic controls. (**A**) Isothermal dose−response of QR2 stabilization by ligand-target binding of different inhibitors tested (at 73°C) using CETSA (*n* = 3 per group; EC50; YB-808 = 13 nM; YB-800 = 34 nM; YB-537 = 129 nM). (**B**) Cell viability measured with Cell-Titer-Glo assays in response to 72 hour incubations with increasing doses of 3 different inhibitors, using THLE2 cells (LD50; YB-808 = 59.704 μM, *n* = 2; YB-800 = 78.401 μM, *n* = 2; YB-537 >100 μM, *n* = 3). (**C**) Repeat validation of toxicity assays, using XTT in HEK293 cells at physiologically relevant concentrations of 3 inhibitors, shows no toxicity following either 3- or 24 hour periods of inhibitor treatment (*n* = 3). (**D**) A 3 hour incubation with 20 μM YB-800 reduces induced ROS levels in HCT116 (*n* = 5 per group; unpaired *t* test, *P* = 0.0338) while no change is seen in HCT116 QR2Δ cells following the treatment (*n* = 5 per group; unpaired *t* test, *P* = 0.8464). (**E**) QR2 expression is unchanged in HCT116 cells treated for 4 days with 2 μM YB-800 (*n* = 6 per group; unpaired *t* test, *P* = 0.2851). (**F**) CD73 expression is significantly increased in HCT116 cells following 4-day incubation with 2 μM YB-800 (*n* = 6 per group; Mann-Whitney test, *P* = 0.0022). Data are shown as mean ± SEM; *P < 0.05; **P < 0.01.

To establish direct target engagement and cell membrane permeability, a cellular thermal shift assay (CETSA) was carried out, using leading QR2is YB’s-537, 800, and 808. Following a 1 hour preincubation with 5 μM of the QR2is or vehicle, thermal aggregation curves (Taggs) of QR2 expressed in HEK293T were measured in increasing temperatures. All QR2is increased QR2 thermal stability compared with vehicle (Supplemental Figure 2A). Based on the Tagg curves we selected 73°C for isothermal dose-response fingerprint (IDTRF) experiments (Supplemental Figure 2B). The ITDRF allowed the relative quantification of the binding of each of the QR2is to QR2, measured as the EC_50_ (Figure 3A). This shows that the inhibitors can penetrate the cell membrane and directly bind the target QR2 protein within. We evaluated the safety of the QR2is using cell toxicity and viability assays. Toxicity was assessed in an ATP depletion assay using THLE-2 cells following a 72 hour exposure to a dose-response of QR2is. Only 1 of the QR2is exhibited an IC_50_ under 10 μM (PCM-0212354; Supplemental Table 1), while the leading QR2is displayed much higher values (Figure 3B), exemplified by YB-537. Cell viability was assessed by XTT assay in HEK239T cells, using a dose response, with 3- or 24-hour incubations of the leading QR2is. No sign of toxicity was seen (Figure 3C). Next, we wanted to assess if, similar to QR2 KO, QR2is can reduce cellular ROS in HCT116 cells and see whether any observable effect is occluded in QR2Δ cells. We used YB-800, which showed an EC_50_ between YB-808 and YB-537 (Figure 3A). ROS levels were significantly reduced 3 hours after treatment with 20 μM YB-800 in the WT but not QR2Δ HCT116 cells (Figure 3D), indicating a QR2-specific effect of the inhibitor. We then assessed WT HCT116 cells by immunoblot (Supplemental Figure 2, C and D), after being treated with YB-800 (2 μM) for 4 consecutive days. QR2 expression was unaltered (Figure 3E); however, CD73 showed a significant increase (Figure 3F), similar to QR2Δ. Thus, the QR2is can bind native QR2 in human cells and replicate the effects seen using genetic QR2 removal.

### Specific QR2is enhance cortical and hippocampal learning in mice and rats.

**Figure 4:**
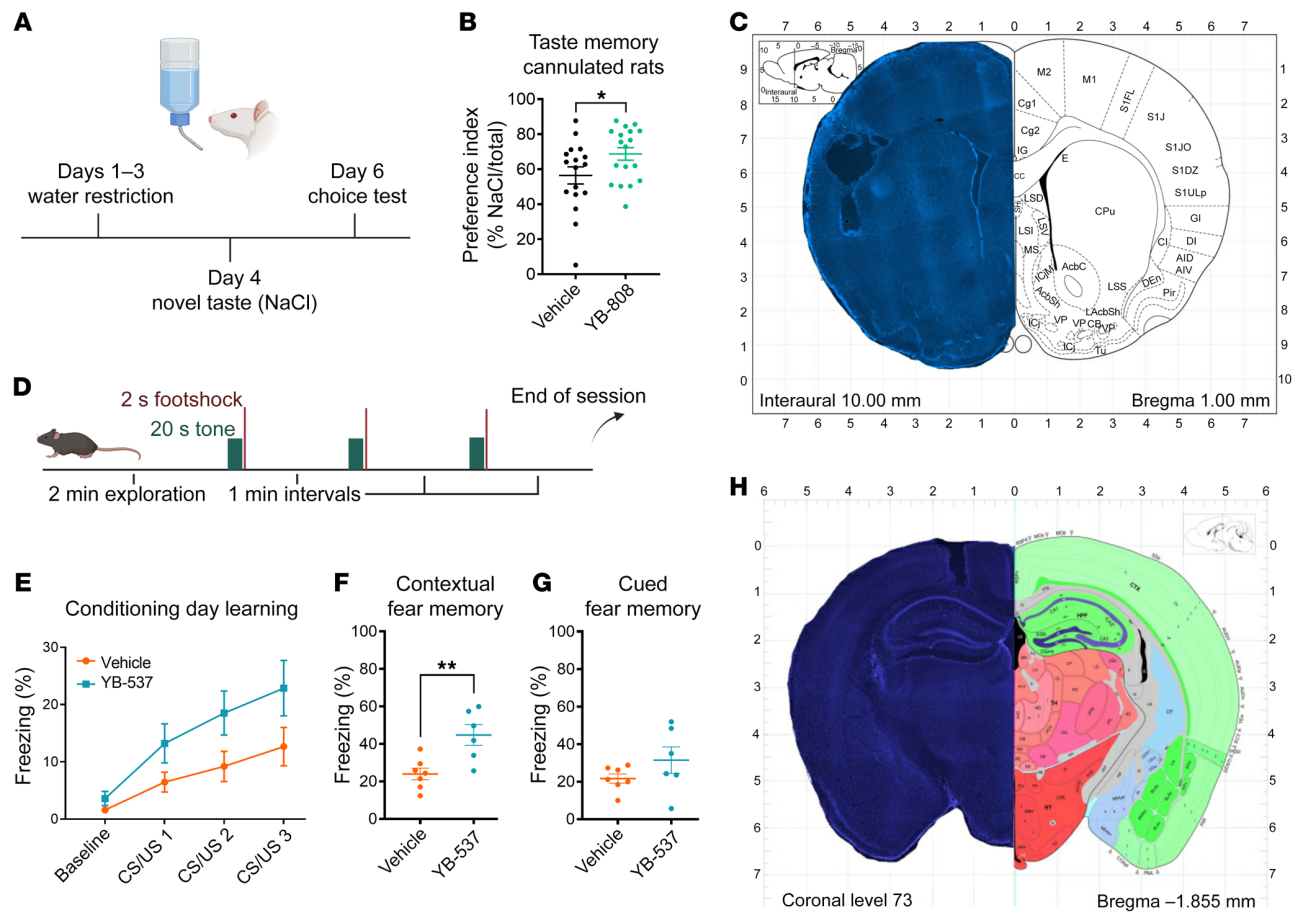
Specific QR2is enhance cortical and hippocampal learning in mice and rats. (**A**) Outline of novel taste learning in rats. (**B**) Rats that were microinjected with 20 μM YB-808 to the aIC (*n* = 18) drank significantly more NaCl than the vehicle control (*n* = 17) group (unpaired *t* test, *P* = 0.0498). (**C**) Cannula placement in the rat aIC.(**D**) Outline of DFC in mice. (**E**) Mice microinjected with 5 μM YB-537 (*n* = 6) or vehicle (*n* = 7) both show normal inter-trial learning (2-Way RM ANOVA, trial: *P* < 0.0001, Groups: *P* = 0.0723). (**F**) Mice that were microinjected with 5 μM YB-537 (*n* = 6) freeze significantly more than vehicle (*n* = 7) control in response to the context (unpaired *t* test, *P* = 0.0056). (**G**) Mice that were microinjected with 5 μM YB-537 (*n* = 6) showed no difference in freezing compared with vehicle (*n* = 7) control in response to the cue (unpaired *t* test, *P* = 0.1904). (**H**) Cannula placement in the mouse CA1 formation. Data are shown as mean ± SEM; *P < 0.05; **P < 0.005.

QR2 elimination or inhibition has been shown to improve rodent memory in several tasks (7, 16, 17), while high levels of QR2 have been correlated with memory impairment (31). In order to evaluate the efficacy of some of the leading QR2is in vivo, we carried out double-blind experiments using 2 different modalities (Supplemental Methods), measuring memory that is dependent on different brain areas. First, cortical memory was tested using incidental novel taste learning (see Methods), in which an unfamiliar, but palatable and safe, taste is given for the first time, and the memory for this newly learned taste is then assessed 2 days later. This is done by measuring the volume of the taste consumed upon subsequent exposure to it (32) (Figure 4A). Rats microinjected with YB-808 (1 μL of 20 μM) via cannula to the anterior insular cortex (aIC; the primary gustatory cortex) drank significantly more NaCl than the control group, therefore displaying a stronger memory of the safe taste (Figure 4, B and C). Next, hippocampal-dependent learning was evaluated using delay fear conditioning (DFC; see Methods) (33). Mice were cannulated to the CA1 region of the hippocampus and were microinjected with YB-537 (1 μL of 5 μM, Figure 4, D and H) or vehicle. Mice displayed normal learning during conditioning (Figure 4E), with mice microinjected with YB-537 to CA1 showing significantly increased freezing in response to the conditioned context upon reexposure the following day, indicating enhanced hippocampal-dependent memory (Figure 4F). No significant difference in freezing levels in response to the cue was seen between groups (Figure 4G), indicating no change to amygdala-dependent memory. In order to evaluate any possible effect of acute QR2i microinjection on metabolic stress in the mouse brain, ISR activation was measured by quantifying total and phosphorylated eukaryotic initiation factor 2 α (eIF2α) levels (Supplemental Figure 3A) and lipid peroxidation product, 4-hydroxynonenal (4-HNE; Supplemental Figure 3B), by immunoblot 3 hours after the injection. No significant reduction in these metabolic markers was detected. These experiments demonstrated the ability of the QR2is to replicate behavioral results previously obtained using genetically induced QR2 elimination or inhibition (6, 7).

### YB-537 bound to human QR2 shows conserved ligand-target interactions that are absent in the closely related QR1.

**Figure 5:**
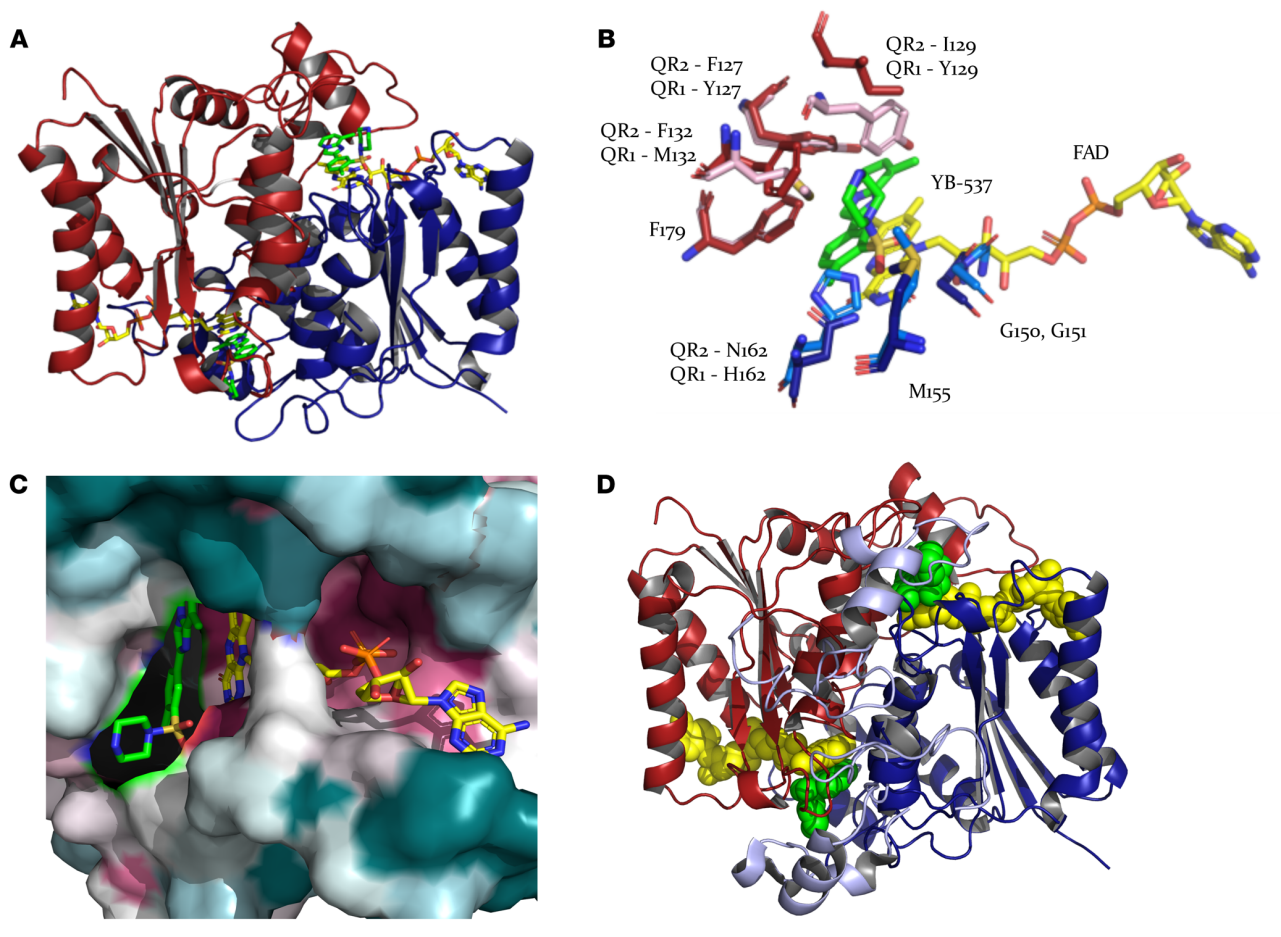
YB-537 bound to hQR2 shows conserved ligand-target interactions that are absent in the closely related QR1. (**A**) Ribbon representation of the hQR2 homodimer (dimer A, red and dimer B, blue) with stick representation of FAD (yellow) and YB-537 (green). (**B**) Critical interactions with amino acids in hQR2 (blue and red) are absent in hQR1 (cyan and pink) due to differences in the amino acid sequence and structure of the 2 enzymes. Specifically, hQR2 has I129, F132, and N162, the latter of which forms an important hydrogen bond with YB-537, that are replaced with Y129, M132, and H162, respectively, in hQR1. (**C**) Consurf analysis (https://consurf.tau.ac.il/) showed that the amino acids interacting with the FAD prosthetic group are highly conserved across hQR1 and hQR2, as indicated by the maroon color in the ConSurf representation. However, the amino acids interacting with YB-537 are less conserved, as indicated by the turquoise color in the ConSurf representation. (**D**) Ribbon representation of hQR1 dimer (red and blue) and the FAD molecule (yellow). Additionally, a 43 amino acid residue C-terminus is shown in white, which is absent in hQR2. This C-terminus structure may physically hinder YB-537, shown in green, from accessing the catalytic site. The figures were created using the program PyMOL (58).

To make QR2-ligand crystallization amenable, enable oral administration of the inhibitors, and eliminate undesirable formulations, HCl was conjugated to YB-537 (Supplemental Methods). This resulted in complete solution of YB-537 in water. Purified human QR2 (hQR2) was then cocrystalized in the presence of the water-soluble inhibitor (Supplemental Methods; Figure 5A). The resolved crystal structure of hQR2 in complex with YB-537 revealed that the protein exists as a homodimer, consistent with previous reports (25). YB-537 was found to bind to each monomer of the hQR2 homodimer, forming interactions with amino acids from both monomers as well as with the Flavin-adenine dinucleotide (FAD) cofactor. The plane of YB-537 stacks up parallel to the isoalloxazine ring of the FAD cofactor with an average distance of 3.5 Å between the planes of the two. Each FAD moiety forms 17 contacts within 3.5 Å to atoms from YB-537 and 48 contacts to 17 amino acids from one monomer, namely H12, S17, F18, N19, S21, P103, L104, Y105, W106, F107, T148, T149, G150, G151, Y156, E194, and R201. YB-537 binds to the catalytic site through a series of hydrophobic and hydrogen bonds with both FAD and amino acids from both QR2 monomers (Figure 5B). Specifically, YB-537 forms 6 contacts with G150, G151, and M155 and a hydrogen bond with N162 from one monomer (A-blue) and 8 contacts to F127, I129, F132, and F179 from the other monomer (B-red). These amino acid residues may be important for the binding and selectivity of YB-537 toward hQR2.

To estimate the evolutionary conservation of amino acids in hQR2 and related proteins, we used the ConSurf server (34). The server generated multiple sequence alignments with 150 homologous proteins and predicted the conservation of amino acids based on their evolutionary history. This clearly showed high conservation among the amino acids interacting with FAD (Figure 5C), while showing far less conservation with those interacting with YB-537 (Figure 5C). Superposition of the catalytic site of QR2, in complex with YB-537, with QR1 (PDB-ID code 2F1O, Figure 5B) revealed that,while some catalytic site amino acids were strictly conserved, others such as I129, F132, and N162 in QR2 are Tyr, Met, and His, respectively, in QR1. This, the additional 43-residues of the C-terminus of QR1 (Figure 5D), and other contrasting features (Supplemental Figure 4) may explain the observed more than 6,000-times higher specificity of YB-537 to QR2 compared with QR1.

### Ingestion of YB-537 in drinking water for 1 month improves cognitive function in 9-month-old 5xFAD female mice.

**Figure 6:**
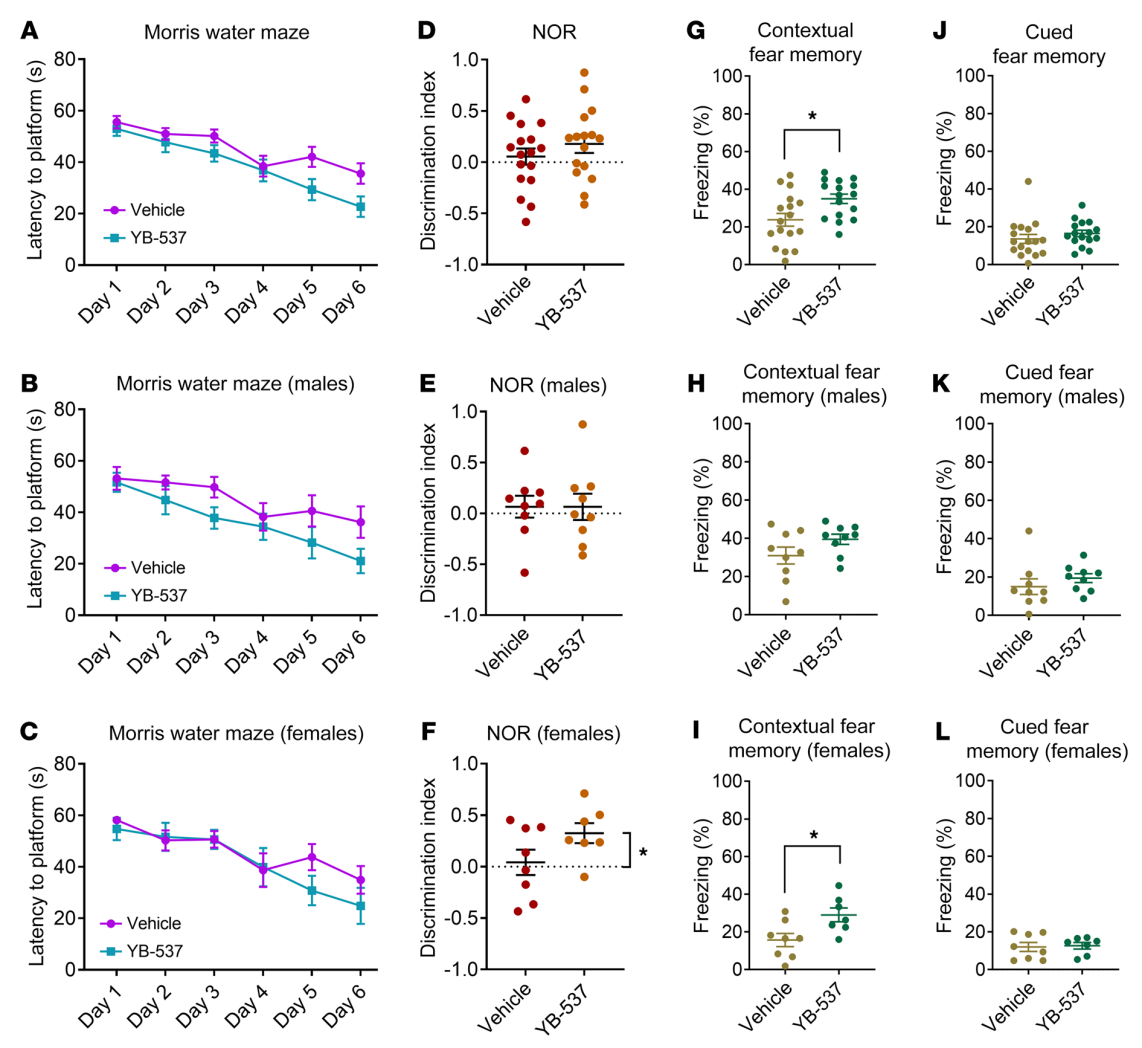
Ingestion of YB-537 in drinking water significantly improves cognitive function in 9-month-old 5xFAD female mice. (**A**) Mice drinking YB-537 trend toward faster spatial learning in MWM (2-way RM ANOVA, treatment: *P* = 0.0694). (**B**) Male mice drinking YB-537 show similar spatial learning in MWM to controls (2-way RM ANOVA, *P* = 0.1115). (**C**) Female mice drinking YB-537 show similar spatial learning in MWM to controls (2-way RM ANOVA, *P* = 0.4272). (**D**) Mice drinking YB-537 trend toward novel object discrimination (1 sample *t* test, *P* = 0.0603), and controls do not (1 sample *t* test, *P* = 0.5004). (**E**) Male mice drinking YB-537 and controls do not show novel object discrimination (1 sample *t* test, YB-537 *P* = 0.6302; vehicle *P* = 0.5600). (**F**) Female mice drinking YB-537 show novel object discrimination (1 sample *t* test *P* = 0.0154), while controls do not (1 sample *t* test *P* = 0.7447). (**G**) Mice drinking YB-537 freeze significantly more than controls in response to the conditioned context (unpaired *t* test, *P* = 0.0131). (**H**) Male mice drinking YB-537 freeze similarly to controls in response to the conditioned context (unpaired *t* test, *P* = 0.1168). (**I**) Female mice drinking YB-537 freeze significantly more than controls in response to the conditioned context (unpaired *t* test, *P* = 0.0207). (**J**) Mice drinking YB-537 freeze similarly to controls in response to the conditioned cue (Mann-Whitney test, *P* = 0.1000). (**K**) Male mice drinking YB-537 freeze insignificantly more than controls in response to the conditioned cue (Mann-Whitney test, *P* = 0.0939). (**L**) Female mice drinking YB-537 freeze similarly to controls in response to the conditioned cue (unpaired *t* test, *P* = 0.8243). *n* for all experiments: YB-537, 16 (9 males and 7 females); vehicle, 17 (9 males and 8 females). Data are shown as mean ± SEM; *P < 0.05.

We next aimed to test the effect of QR2 inhibition in AD model mice using YB-537, taking advantage of its extremely high specificity, solubility, and lack of toxicity. First, we determined YB-537 pharmacokinetics (PK) and oral bioavailability and assessed any acute observable toxicity in mice. It was found that YB-537 was well tolerated at 50 mg/kg per os (p.o.) or 10 mg/kg i.v. with no discernable adverse symptoms at any time up to 24 hours following administration (Supplemental Pharmacokinetic Study in the Supplemental Materials). YB-537 was 82% bioavailable p.o., and peak concentrations of 203 ng/g (equivalent to approximately 500 nM YB-537) were detected in the brain approximately one hour after oral administration (Supplemental Figure 5, A and B). We therefore opted to chronically deliver YB-537 to AD-model mice via their drinking water, so they may freely ingest the inhibitor at 50 mg/kg, with minimal intervention or trauma, for 1 month. We chose 9-month-old 5xFAD (27) male and female mice in double-blind experiments, so that well-progressed symptoms and pathologies would be present, to mimic clinically relevant cases in human patients (35), as has been previously described in this mouse model. Other than an improvement in nest quality (36) over time seen in animals receiving YB-537 (starting at age 8 months and ending at age 9 months), no changes to general physical parameters or wellbeing were observed over the 1 month of YB-537 ingestion (Supplemental Figure 6, A–J). Animals received the treatment for a week, and, on week 2, behavioral experiments commenced, including Morris water maze (MWM) and novel object recognition (NOR). These were carried out in parallel, with half of the animals (including both sexes and treatments) undergoing 1 of the paradigms in week 2, and the other in week 3. We found that in the MWM, the escape latency of the group receiving YB-537 trended to be faster than the control group (Figure 6A), and a similar pattern was seen both in male (Figure 6B) and female (Figure 6C) mice. Since learning in this paradigm was slow, and performance was poor even following 6 days of training (using 4 learning trials a day), a test day was not carried out and instead only the learning rate was measured. In the NOR task, mice receiving YB-537 trended to discern the novel object (Figure 6D), but no object discrimination was observed in any of the male mice (Figure 6E). Contrastingly, female mice receiving YB-537 significantly preferred to investigate — and were able to discern the novel object — while females receiving vehicle did not (Figure 6F). Finally, during week 4, all the mice underwent DFC. Both control mice and those receiving YB-537 in drinking water showed similar learning curves during conditioning, regardless of sex (Supplemental Figure 7). Mice receiving YB-537 froze significantly more in response to the conditional context (Figure 6G) compared with controls. While the male mice groups did not significantly differ in response to context (Figure 6H), female mice receiving YB-537 froze significantly more than controls in response to the conditioned context (Figure 6I). Mice receiving YB-537 did not show any difference in freezing in response to the conditioned cue (Figure 6J), and neither did the male and female groups (Figure 6, K and L). Overall, throughout a month-long experiment during which 8-to-9-month-old 5xFAD mice of both sexes received 50 mg/kg of YB-537 in their drinking water, no adverse effects were seen, and an improvement in nest quality and cognitive function was measured, the latter mainly in females.

### Drinking YB-537 for 1 month significantly reduces brain pathologies associated with dementia in 9-month-old 5xFAD mice.

**Figure 7:**
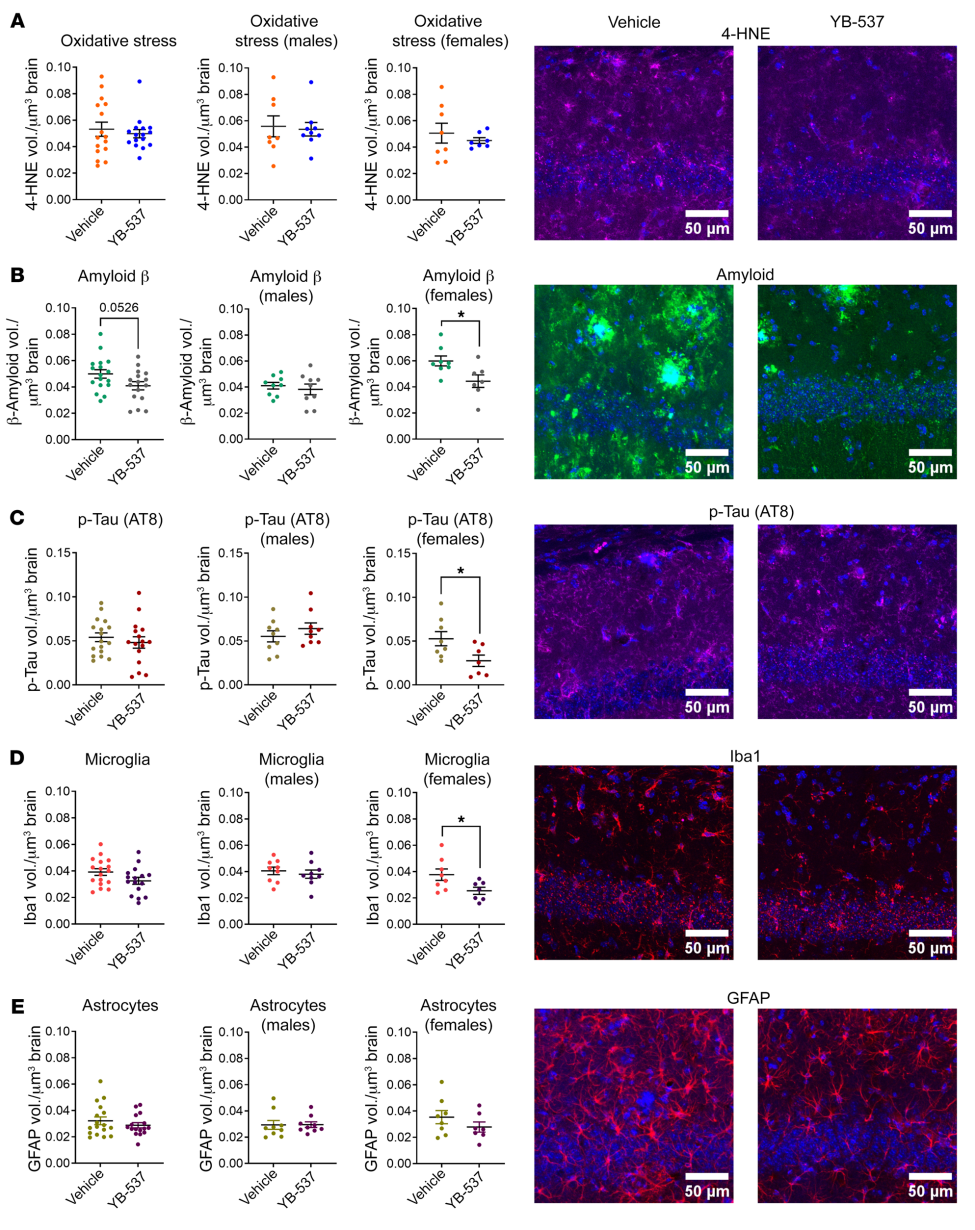
Drinking YB-537 for 1 month significantly reduces brain pathologies associated with dementia in the dorsal CA1 hippocampal formation of 9-month-old 5xFAD female mice. (**A**) Oxidative stress, as indicated by 4-HNE, is not significantly altered in 5xFAD mice CA1 following 1 month of drinking YB-537 (both sexes, Mann-Whitney test, *P* = 0.8965; males, Mann-Whitney test, *P* = 0.6730; females, unpaired *t* test, *P* = 0.8155), but tends to alter distribution in the total population (F test, *P* = 0.0516), and significantly changes distribution in females (F test, *P* = 0.0052). (**B**) Amyloid β shows a trend of reduction following 1 month of drinking YB-537 in the CA1 of the total 5xFAD mouse population (unpaired *t* test, *P* = 0.0526), is unchanged in the male population (unpaired *t* test, *P* = 0.5643), and is significantly reduced in the female population (unpaired *t* test, *P* = 0.0237). (**C**) p-tau is unchanged following 1 month of drinking YB-537 in the CA1 of total 5xFAD mouse (unpaired *t* test, *P* = 0.4678) and male (unpaired *t* test, *P* = 0.3432) populations, but is significantly reduced in the females (unpaired *t* test, *P* = 0.0337). (**D**) Iba1 is insignificantly reduced in 5xFAD mouse CA1 following 1 month of drinking YB-537 in the total population (unpaired *t* test, *P* = 0.0738), is unchanged in the male population (unpaired *t* test, *P* = 0.5625), but is significantly reduced in the female population (unpaired *t* test, *P* = 0.0380). (**E**) GFAP is unchanged in 5xFAD mouse CA1 following 1 month of drinking YB-537 in the total population (Mann-Whitney test, *P* = 0.6567), is unchanged in the male population (Mann-Whitney test, *P* = 0.6048), and is unchanged in the female population (unpaired *t* test, *P* = 0.2654). *n* for all experiments: YB-537, 16 (9 males and 7 females); vehicle, 17 (9 males and 8 females). Data are shown as mean ± SEM. *P < 0.05. Scale bars: 50 μm.

Following the behavioral experiments, we assessed brain pathologies associated with AD present in the 5xFAD mice (37). Therefore, 5 days following the last behavioral experiment, and 1 month after the start of YB-537 consumption, mice were sacrificed, and coronal brain sections were used for IHC. Since the CA1 region of the hippocampus is affected during AD pathogenesis (37), and this brain region is involved in the behavioral paradigms used here, we acquired images of CA1. This was done with an Olympus IX83 confocal microscope using the same settings, antibodies, and exposures in all mice. Images were analyzed using Imaris (Bitplane; see methods). For each mouse, 3 coronal sections were used per antibody. In each section, the same region of CA1 was acquired, using the same image frame size and resolution, at all possible depths of the section — using a Z-stack of the whole section — to allow measurement of the fluorescent antibody marker volume, which was normalized to the brain volume from which it was taken. First, we measured oxidative stress, as indicated by 4-HNE (38) (Abcam, ab48506). No difference in average 4-HNE measurement was seen between mice receiving YB-537 or controls, though YB-537 reduced high- and low-percentile measurements (vehicle upper quartile 0.0719, lower quartile 0.0370; YB-537 upper quartile 0.0541, lower quartile 0.0418), tending to alter distribution (F test, *P* = 0.0516), which was much more tightly centered around the mean compared with controls (i.e. the controls showed greater variability; Figure 7A). Males showed no difference between groups (Figure 7A). Females did not show any changes in mean 4-HNE levels, but had a significantly different distribution (F test, *P* = 0.0052), with all animals receiving YB-537 showing measurements centered around the mean (vehicle upper quartile 0.0697, lower quartile 0.0308; YB-537 upper quartile 0.0514, lower quartile 0.0409), while controls showed far greater variability (Figure 7A). Amyloid β was measured, and a reduction trend was seen in mice receiving YB-537 compared with controls (Figure 7B), though no changes were seen in males (Figure 7B). In contrast, female mice treated with YB-537 showed significantly reduced amyloid β (Figure 7B). When measuring p-tau (AT8) (37), no changes were seen in the total population (Figure 7C) or males (Figure 7C), but a significant reduction in p-tau was seen in females (Figure 7C). Next, microglia activation was evaluated in CA1 (Iba1), and no change in the total (Figure 7D, left histogram) or male (Figure 7D, middle histogram) populations was seen, but a significant reduction in females receiving YB-537 was measured (Figure 7D). Astrocyte activation (GFAP), did not significantly change in the total, male, or female populations (Figure 7E). Thus, 9-month-old 5xFAD mice showed a reduction in AD-related pathologies in CA1 following 1 month of YB-537 ingestion, with females (Supplemental Figure 9) showing far more significant reduction in pathology than males (Supplemental Figure 10). In the cortex, neither male nor female mice showed a significant reduction in 4-HNE, p-tau, or amyloid β (Supplemental Figure 8, A–C). However, a significant reduction was measured in Iba1 in both total and female populations, and a significant reduction in GFAP was measured in both total and male populations (Supplemental Figure 8, D and E, Supplemental Figure 11, and Supplemental Figure 12). Whole-brain soluble and insoluble amyloid β 42 levels were not significantly reduced in the total mice population, males or females (Supplemental Figure 13). Overall, following 1 month of chronic YB-537 ingestion, female 5xFAD mice showed a greater reduction in AD-associated brain pathologies compared with the more modest effect found in males, mirroring the behavioral results obtained from these mice.

### Ingestion of YB-537 in drinking water for 4 months improves cognitive function in 9-month-old 5xFAD male mice.

**Figure 8:**
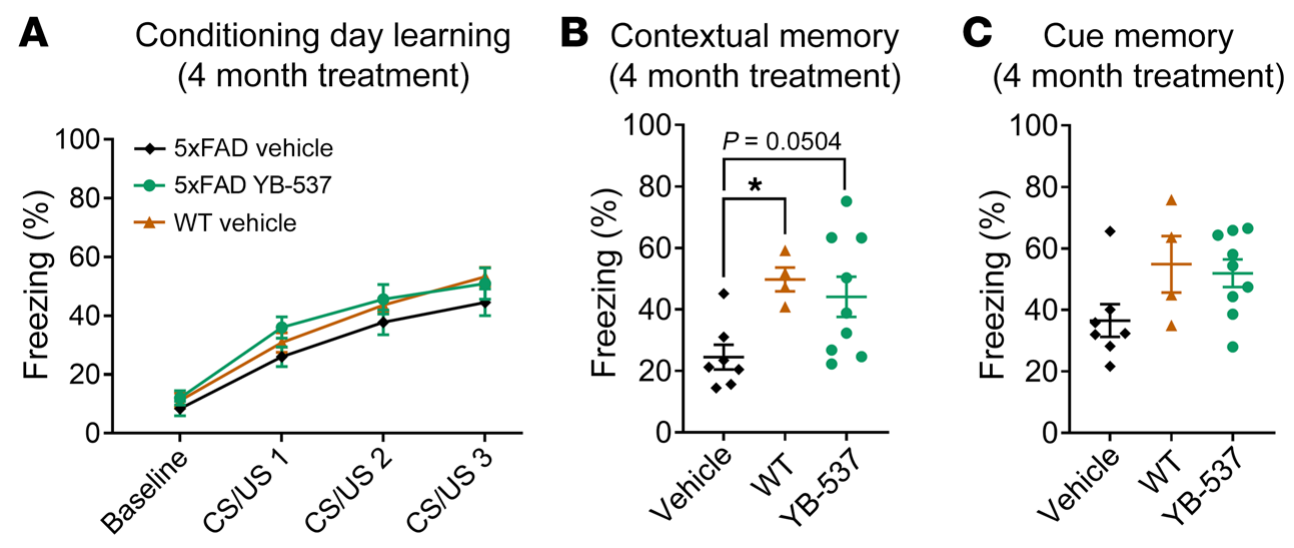
Ingestion of YB-537 in drinking water for 4 months improves cognitive function in 9-month-old 5xFAD male mice. (**A**) Male 5xFAD mice with or without YB-537 in drinking water and WT mice all show normal learning during delay-fear conditioning, with no difference observed across groups (2-Way RM ANOVA, trial: *P* < 0.0001, groups: *P* = 0.3303). (**B**) Male 5xFAD mice that received vehicle freeze less (*P =* 0.05) than WT mice in response to the conditioned context, and 5xFAD mice that received YB-537 for 4 months in their drinking water freeze similarly to WT mice (1-way ANOVA, *P =* 0.0239; Tukey’s multiple comparison, YB-537 versus vehicle, *P =* 0.0504; YB-537 versus WT, *P =* 0.8108; vehicle versus WT, *P =* 0.0420). (**C**) Male 5xFAD mice that received vehicle tend to freeze less in response to the conditioned cue in comparison to 5xFAD mice that received YB-537 for 4 months in their drinking water, and WT mice (1-way ANOVA, *P =* 0.0835). *n* for all experiments: YB-537, 9; Vehicle, 7; WT, 4. Data are shown as mean ± SEM. *P < 0.05.

Since 1 month of chronic YB-537 ingestion rescued cognitive and pathological phenotypes more efficiently in female compared with male 5xFAD mice, we hypothesized that males may require a longer dosing regimen than females in order achieve a similar therapeutic effect. We therefore ran an additional experiment where we treated 5-month-old male 5xFAD mice with 50 mg/kg of YB-537 in drinking water for 4 months (starting at the age of 5 months, and ending at the age of 9 months, similar to the experiment described above), and compared cognitive performance to male 5xFAD and WT control mice of the same age not being treated with YB-537, using DFC in double-blind experiments. We found that, while all male mouse groups showed similar learning curves during conditioning (Figure 8A), 5xFAD mice receiving YB-537 for 4 months froze similar to WT mice, who froze significantly more than control 5xFAD mice, in response to the conditioned context in the DFC test (Figure 8B). Upon reexposure to the cue, 5xFAD mice receiving YB-537 and WT mice both froze more than control 5xFAD mice, but not to a significant extent (Figure 8C). These results indicate that male 5xFAD mice tend to show improved hippocampal dependent cognitive performance following a more prolonged, 4-month treatment with YB-537, starting at an earlier age.

### Drinking YB-537 for 4 months significantly reduces brain pathologies associated with dementia in 9-month-old 5xFAD male mice.

**Figure 9:**
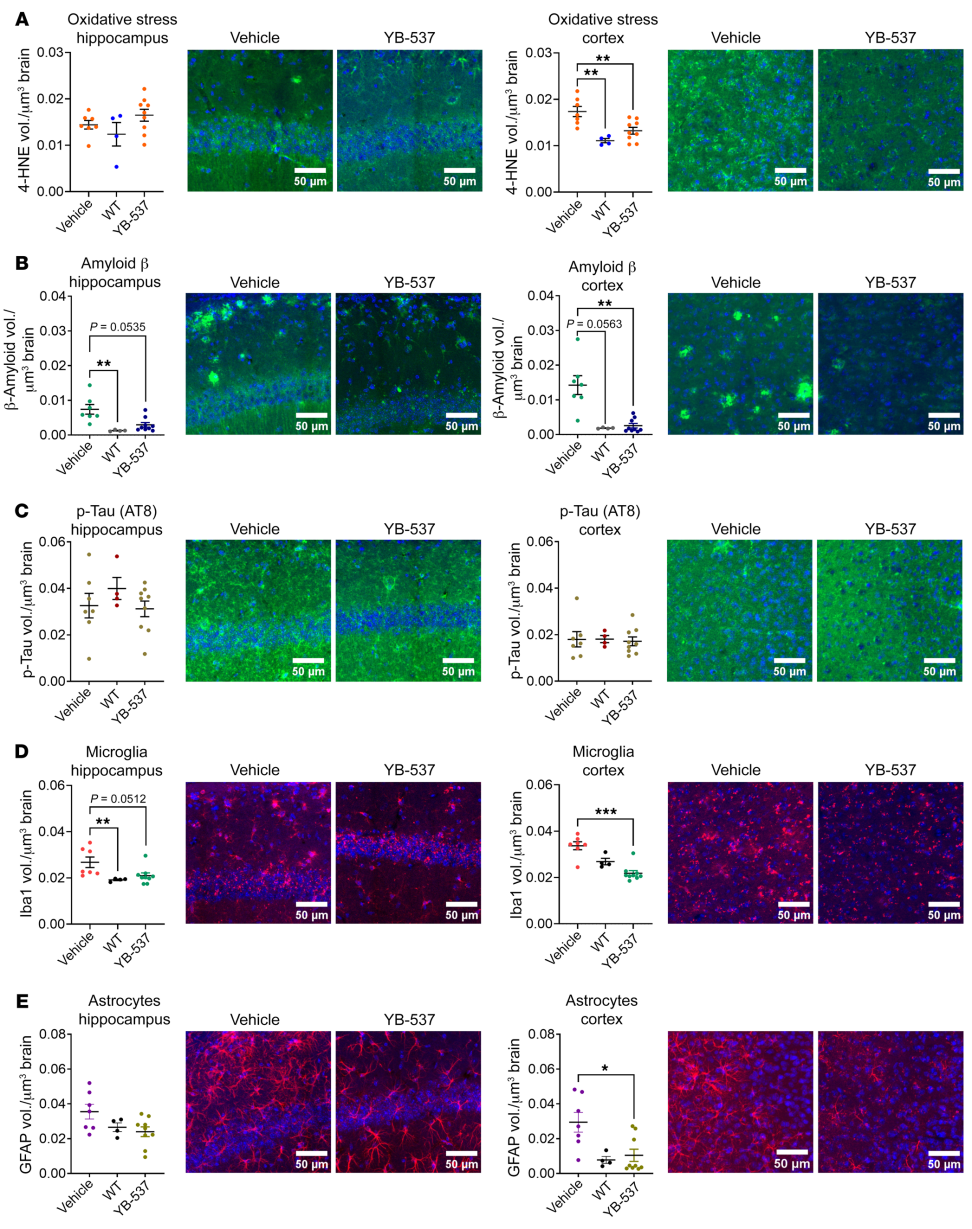
Drinking YB-537 for 4 months significantly reduces brain pathologies associated with dementia in the brains of 9-month-old 5xFAD male mice. (**A**) 4-HNE is unchanged in the hippocampus (1-way ANOVA, *P =* 0.1926), but is significantly reduced in the cortex (1-way ANOVA, *P =* 0.0009; Tukey’s multiple comparison, YB-537 versus vehicle, *P =* 0.0070; YB-537 versus WT, *P =* 0.3029; vehicle versus WT, *P =* 0.0013) of 9-month-old male 5xFAD mice following 4-months of YB-537 consumption in drinking water. (**B**) Amyloid β is reduced in the hippocampus (Kruskal-Wallis test, *P* < 0.0001; Dunn’s multiple comparison, vehicle versus WT, *P =* 0.0013; vehicle versus YB-537, *P =* 0.0535; WT versus YB-537, *P =* 0.2789), and significantly reduced in the cortex (Kruskal-Wallis test, *P =* 0.0003; Dunn’s multiple comparison, vehicle versus WT, *P =* 0.0563; vehicle versus YB-537, *P =* 0.0026; WT versus YB-537, *P* > 0.9999) of 9-month-old male 5xFAD mice, following 4-months of YB-537 consumption in drinking water. (**C**) p-tau is unchanged in the hippocampus (1-way ANOVA, *P* = 0.4520) and the cortex (1-way ANOVA, *P =* 0.9557) of 9-month-old male 5xFAD mice, following 4-months of YB-537 consumption in drinking water. (**D**) Iba1 is reduced in the hippocampus (Kruskal-Wallis test, *P =* 0.0011; Dunn’s multiple comparison, vehicle versus WT, *P =* 0.0058; vehicle versus YB-537, *P =* 0.0512; WT versus YB-537, *P =* 0.6510), and significantly reduced in the cortex (Kruskal-Wallis test, *P* < 0.0001; Dunn’s multiple comparison, vehicle versus WT, *P =* 0.6638; vehicle versus YB-537, *P =* 0.0008; WT versus YB-537, *P =* 0.2284) of 9-month-old male 5xFAD mice, following 4 months of YB-537 consumption in drinking water. (**E**) GFAP is insignificantly reduced in the hippocampus (1-way ANOVA, *P =* 0.0589), and significantly reduced in the cortex (Kruskal-Wallis test, *P =* 0.0116; Dunn’s multiple comparison, vehicle versus WT, *P* = 0.1450; vehicle versus YB-537, *P =* 0.0215; WT versus YB-537, *P* > 0.9999) of 9-month-old male 5xFAD mice, following 4 months of YB-537 consumption in drinking water. *n* for all experiments: YB-537, 9; vehicle, 7; WT, 4. Data are shown as mean ± SEM. *P < 0.05; **P < 0.005; ***P < 0.0005. Scale bars: 50 μm.

Following the behavioral results obtained with 4-month YB-537 treatment in the male 5xFAD mice, we evaluated AD-related brain pathologies using IHC, similar to the 1-month treatment experiment (Supplemental Figures 14–17). The marker for oxidative stress, 4-HNE, did not show any changes in the hippocampus, and no differences were seen in result distribution (Figure 9A, left), similar to males following 1 month of treatment. However, a significant reduction in 4-HNE was measured in the cortex following 4 months of YB-537 treatment, bringing oxidative stress levels down to those seen in WT control mice (Figure 9A, right). In contrast with results from the 1 month treatment, following 4 months of YB-537 ingestion, male 5xFAD mice showed a significant reduction in amyloid β in the cortex (Figure 9B, right), and tended to show less amyloid β in the hippocampus (Figure 9B, left). Levels of p-tau remained unchanged across all 3 groups, both in the hippocampus (Figure 9C, left) and cortex (Figure 9C, right). Microglia activation levels tended to be reduced in the hippocampus (Figure 9D, left), and were significantly reduced in the cortex (Figure 9D, right) following 4 months of YB-537 consumption in the male 5xFAD mice. This was also seen with astrocyte activation, in which a reduction trend was found in the hippocampus (Figure 9E, left), and a significant reduction was measured in the cortex (Figure 9E, right). Overall, a significant reduction in brain pathologies was seen across the cortex and hippocampus of 9-month-old male 5xFAD mice, following 4 months of chronic YB-537 ingestion.

## Discussion

Age-related cognitive decline and prolonged metabolic stress in the brain represent major medical challenges in the modern era (39). A central approach toward dealing with mild cognitive impairment and dementia lies in tackling dysfunctional metabolism and incurred inflammation in the brain (1, 40). Recently, QR2 was proposed to be a novel contributor to the metabolic aging of the brain (6, 7, 16). QR2 normally operates within a pathway that enacts oxidative eustress (41), as QR2 generates physiological levels of ROS that modulates neuronal activity (7, 10). In health, this QR2 pathway is activated hours after a novel experience, causing the transient removal of QR2 from neurons and reducing ROS and inhibitory interneuron excitability (7, 10). As a result, QR2 removal resets neuronal population excitation/inhibition dynamics, enhancing long-term memory formation for new, salient information. This critical mechanism allows rodents to differentiate between salient and unimportant information and appropriately adapt behavior throughout life, as subsequently to QR2 removal the newly learned information stabilizes for very long periods of time. With age, factors that dynamically govern and control QR2 levels deteriorate, while baseline QR2 levels increase (20). These include diminishing levels of melatonin, DA, ACh, and miR-182 (12, 13, 14), which either drive QR2 removal or inhibit QR2 activity. With control of QR2 expression and redox modulation levels lost, newly learned information is more poorly remembered, as ever increasing QR2 expression turns oxidative eustress to oxidative stress (7, 15).

Here, we found that removing such chronically high levels of QR2 results in a proteome that is functionally opposed to that seen in AD brains (20), where high QR2 expression levels are found (16, 30), and in which QR2 polymorphisms adversely affect pathology (29), strengthening the case for QR2 inhibition as a therapeutic avenue.

To date, a number of QR2is have been developed (22), providing valuable insight into a number of subjects involving QR2. Some were originally created to investigate melatonin and its receptors (22), while others are inhibitors for Abl1 and other cancer-related kinases (23, 42). Notably, of the latter, Imatinib (Gleevec) is an FDA-approved drug, with multiple other non-Abl1 off-targets apart from QR2 (43). Despite severe side effects and poor brain bioavailability (44), there was initial promise for Imatinib/Gleevec as an AD drug, by inhibiting γ-secretase activating protein (45). However, Gleevec treatment in patients with AD has yet to succeed, though efforts in this direction have been recently pursued (44). Ultimately, all of the existing QR2is are either derived from small molecules with other known targets (46); are intended to investigate non-QR2 related function (24, 43); have toxic properties (24); or lack solubility/bioavailability (16). In order to therapeutically address this recently described contributor to age-related metabolic stress and anterograde amnesia expressly, specifically, and directly, we set out to develop a highly selective and safe QR2i to meet the standards required to enable first-in-class AD drug development.

SAR studies of QR2-inhibiting molecules discovered by HTS demonstrated that, at either end of a central sulfonamide linker, several amines and heterocycles strongly inhibited QR2 and could be added in various combinations and modules and allow potential development of hundreds of inhibitor variants. The medicinal chemistry carried out initially, based on this approach, resulted in a family of highly effective, QR2 selective, nontoxic inhibitors, shown to directly bind QR2 in human cells. Of these, some of the most promising were tested for water solubility and in vivo efficacy, the latter by way of measuring the known and previously demonstrated memory-enhancing effect of QR2 inhibition in rodent models (6, 7, 16, 17). Though displaying limited water solubility, the QR2is showed promising results in double-blind behavioral experiments, enhancing cortical memory in rats and hippocampal memory in mice. Both 4-HNE and eIF2α phosphorylation levels showed insignificant reduction 3 hours after YB-537 microinjection in the mouse hippocampus. Subsequently, further medicinal chemistry provided 2 water soluble QR2is (YB-537 and YB-540). Using water-soluble YB-537, the most promising of the QR2is, a crystal structure was resolved, showing the unique features through which YB-537 exhibits its distinct QR2/QR1 inhibition profile. Specifically, while Asn162 in QR2, which is His162 in QR1, provides a hydrogen bond to plant YB-537 within the catalytic site and over the FAD prosthetic group, several other residues found only in QR2 also favorably interact electrostatically with the inhibitor. Further selectivity is likely caused by the 43aa C-terminus structure missing in QR2, which, in QR1, may physically hinder YB-537 inclusion to the catalytic site, as would residues within the QR1 catalytic site itself, such as Y127 and Y129. Ultimately, YB-537 displays more than 6,000-fold higher binding affinity to QR2 than to QR1, which is far greater than the present gold-standard QR2 inhibitor, S29434, which we used as a positive control. Critically, water-soluble YB-537 is 82% bioavailable p.o. and can enter the mouse brain at relevant concentrations with a clearance half life of approximately 1 hour upon acute oral dosing and is nontoxic and well tolerated, enabling long-term oral dosing studies.

Taking advantage of this, we found that by providing YB-537 in the drinking water of 5xFAD mice for 1 month, a significant reduction in brain pathologies and improvement in memory was observed, though this was unexpectedly mainly seen in females. While male and female 5xFAD mice tended to show quicker learning in the MWM, only females demonstrated significant improvement in NOR and DFC paradigms compared with controls. The more potent effect of QR2i in female mice following 1 month of treatment was also noted when measuring brain pathologies. In the hippocampus, both p-tau and amyloid β were significantly diminished following QR2i in the female mouse hippocampus, and microglia activation was also significantly reduced in female mice only. Microglia also showed reduced activation in the female cortex. Interestingly, average 4-HNE levels remained unchanged, but, in females, the number of animals with higher or lower levels of 4-HNE was significantly reduced. This revealed higher homogeneity in 4-HNE levels within the QR2i-group females, compared with much higher heterogeneity in oxidative stress in the control females. Males, in comparison, showed a reduction in astrocyte activation in the cortex, with no other significant changes in any of the other markers, either in the hippocampus or cortex. When assessing amyloid β42 in the whole brain, an insignificant reduction was seen in the soluble but not insoluble fraction in both males and females. Since males showed a slower, more muted response to QR2i than females, we carried out an additional 4 month treatment regimen with male mice and compared them to 5xFAD controls and WT mice, to test whether a longer treatment period would show increased effect in the male population. The longer QR2i treatment did result in much more significant effect, as male 5xFAD mice ingesting YB-537 for 4 months showed similar contextual memory to WT mice, and brain pathologies showed far greater reduction across brain areas. This included significantly reduced 4-HNE in the cortex, with 4 months of QR2i lowering the oxidative stress marker in the 5xFAD mice to levels measured in WT mice. Amyloid β tended to show a reduction in the hippocampus and showed significant reduction in the cortex, while astroglia activation markers GFAP and Iba1 were both similarly reduced in the 5xFAD mice, following the 4 month treatment period. YB-537 therefore reduces brain pathologies and reverses cognitive deficits in the 5xFAD mouse model at an advanced age, with sex-dependent variation in results and a more rapid effect in females. Why females respond to QR2is so much faster than males will be the subject of future studies and may be related to estrogen metabolism (47, 48), though this does not exclude other possibilities, such as those pertaining mitochondrial function and NAD/H homeostasis (8, 15, 49). Our limited knowledge of QR2 function does not yet allow us to portray how QR2 and QR2is may differentially affect certain subpopulations, mitochondrial function (e.g., health and turnover and mitochondrial cofactor metabolism) and inflammation (e.g., ROS, CD73, and adenosine). These open questions and what we believe to be the exciting new research proposing that QR2 regulates specific posttranslational histone modifications and epigenetics via protein-protein interaction (50), warrants future studies in both animal models and patient-derived induced pluripotent stem cells (iPSCs). The latter may be particularly well suited, since a recent study found that interpersonal QR2 variable expression was reliably correlated between brain samples and iPSC-derived neurons (51). An important outcome of prolonged QR2i in 9-month-old 5xFAD mice was the initial homogenization of 4-HNE levels in the brain after 1 month of QR2i and eventual significant reduction in 4-HNE after 4 months, in females and males, respectively. QR2 is a metabolic buffer, which becomes a metabolic stressor with age (7) or when given certain substrates (15, 52, 53). The removal of extreme 4-HNE values from the brains of mice treated with QR2i after 1 month, and a reduction in 4-HNE values after 4 months, may point to a redox-stabilizing effect, perhaps indicative of reemerging metabolic homeostasis. Currently, major efforts are being made to find new ways to slow metabolic aging (51), particularly in the postmitotic neurons of the brain. Therefore, it is of key importance to find new ways to help correct age-related deficits in brain metabolic homeostasis, while enhancing cognition. Here, we show that QR2is answer both demands and cause no adverse side effects.

With the recent description of the QR2 pathway in the brain and its association with cognitive dysfunction and metabolic pathologies with age, we believe that an exceptional opportunity to tackle neurodegenerative diseases like AD has been found. The approach of QR2 inhibition for AD treatment may provide direct or combinatory therapeutic efficacy, along with other disease modifying drugs, such as ISRIB (54), PKR (4), and PERK (55) inhibitors, for which discovery and development are ongoing. The QR2is described here further enable the development of this strategy.

## Methods

### Study design

In this study we created orally available QR2is with the aim to replicate genetic QR2 removal phenotypes, such as reduction of metabolic stress and memory enhancement. Inhibitor specificity for QR2 against QR1 was measured using standardized fluorescent- and orthogonal-absorption activity assays. QR2is were evaluated for in vitro activity and toxicity in QR2-expressing HEK293FT (Thermo Fisher Scientific, R70007) and THLE-2 (ATCC, CRL-2706) cells, and occlusion experiments in QR2-KO cells (Supplemental Methods). QR2i-target interaction was evaluated by crystal structure. In vivo experiments were done blind (Supplemental Methods), using randomly allocated male Sprague Dawley rats (Envigo), male C57BL/6 mice (Envigo), and male and female 5xFAD mice (The Jackson Laboratory stock no. 034840-JAX), in which PK, learning behavior, and brain pathologies were measured (see Methods Behavior, and Supplemental Methods). Group size was based on previously published data for similar experiments and the use of a power calculator (https://www.stat.ubc.ca/~rollin/stats/ssize/n2.html).

### Biochemistry

H_2_DCFDA ROS assay was carried out according to established protocols, with some adjustment (Supplemental Methods) (6). Cell toxicity was measured via ATP depletion (CellTiter Glo, Promega) in THLE-2 cells (ATCC, CRL-2706), following 72 hours of exposure to the test compounds, and cell viability was assessed using XTT assay (Biological Industries) according to manufacturers protocols. CETSA was performed as previously described (53) with some modifications, using QR2 (Santa Cruz, sc-271665) and SOD1 (Santa Cruz, sc-17767) antibodies. SDS-PAGE and immunoblot imaging were carried out as previously described (55), using Tubulin (Sigma-Aldrich, SAB4500087), QR2 (Santa Cruz, sc-271665), NDUFA9 (AbCam, ab14713) and CD73 (Cell Signaling Technology, D7F9A) antibodies.

### Behavior

#### Incidental taste learning.

Rats were taught to drink from 2 pipettes each filled with 10 mL of water during a 20 minute period, over 3 days. On the fourth day, they were given a novel, palatable taste (2 pipettes each filled with 10 mL of 0.3% NaCl), 30 minutes after a local microinfusion of YB-808 or vehicle. On the fifth day, rats were once again given water in the pipettes. On the sixth day, the rats were presented with a choice test, in which they were given 2 pipettes of water and 2 pipettes of NaCl (10 mL in each pipette). The memory for the novel taste was then assessed after 20 minutes of liquid consumption by calculating a preference index as follows: (novel taste consumed/[novel taste consumed+water consumed]) × 100 (32, 56).

#### DFC.

DFC was done as previously described (7). Briefly, mice were given 2 minutes to explore the conditioning chambers, during which baseline freezing was measured. Then, a 20 second, 4 kHz, 80 dB tone was given, coterminating with the start of a 2 second, 0.5 mA foot-shock. This was repeated twice more in 1 minute intervals. Following the third and last bout, 1 minute was given before the animals were removed from the chambers. The next day, the animals were returned to the room under the same conditions and were placed back into the chambers, and freezing to the context was measured over the course of 5 minutes. The next day, the mice were brought to unfamiliar conditioning rooms and chambers. In this unfamiliar context, the protocol from the first day was repeated, except without the foot shocks. Freezing for the cue was recorded, starting from the sounding of the first tone. All measurements were taken with a Sentec stc-tb33usb-at camera, and analysis was done with Freeze Frame software (Actimetrics).

#### MWM.

MWM was carried out as previously described (57). Mice were trained 4 times a day, using 60 second trials every 30 minutes during which they were placed into the pool, each time from a different quadrant, and allowed to swim to find the escape platform. All trials were filmed with a video tracking system using EthoVision 14 (Noldus Information Technology), and escape latency, or time to find the submerged escape platform, was determined by video analysis.

#### NOR.

Following a 10 minute exploration of a 50 × 50 cm open-field arena, mice were returned to the arena the next day, which contained 2 identical objects. Mice were allowed to explore the objects and the arena 3 times for 10 minutes, with an inter-trial interval of 10 minutes. The following day, one of the objects was replaced with a novel object and the mice were returned to the arena and allowed to explore for 10 minutes. Mouse movement, exploration, and nuzzling was automatically recorded with an Ikegami ICD-49E camera with EthoVision 14 (Noldus Information Technology). Discrimination of the novel object was assessed by calculating (time exploring novel object – time exploring familiar object)/(time exploring novel object + time exploring familiar object).

### IHC and image analysis

Perfused and fixed brain slices were blocked with 10% normal donkey serum (DNS) and 0.2% triton (Sigma-Aldrich) in PBS. Antibodies, including 4-HNE (AbCam, ab48506), Iba1 (AbCam, ab5076), AT-8 (Thermo, MN1020), Amyloid β (AbCam, ab201060), diluted in PBS with 10% DNS, were incubated at 4°C overnight. Secondary antibodies including donkey-anti-goat Alexa Fluor 568 (AbCam, ab175704), donkey-anti-mouse Cy 5 (Jackson Immuno Research, 715-175-151), and donkey-anti-rabbit DyLight 488 (AbCam, ab98488) all diluted 1:500 in PBS with 1% BSA, were applied to the slices at RT for 2 hours the next day. Slices were mounted onto glass slides using DAPI containing Vectashield (H-1200). Images were taken using a confocal microscope (Olympus IX83). Three slices were used per mouse per antibody. Tiling images of the dorsal CA1 and cortex (Bregma: –1.58mm to –2.30mm) were taken using ×20 objective, acquiring a Z-stack of the whole section. Analysis of the images was done blind by an experimenter unaware of the experimental conditions or details, using Imaris (Bitplane) software. A surface reconstruction module was used to extract the data as volumes of the used markers (4-HNE, phospho-tau, amyloid β, GFAP, and Iba1). Marker volume and intensity were normalized to the corresponding brain volume. The normalized value was averaged for each triplicate and presented here.

### Statistics

Detailed statistics for the manuscript are available in the Supplemental Materials in Supplemental Table 3. Shapiro-Wilk normality tests were done for the collected data. Analysis of normally distributed data was done using parametric tests (i.e., unpaired 2-tailed Student’s *t* tests, 1-way ANOVA followed by Tukey’s or Šidák’s posthoc analysis) and for data not normally distributed, nonparametric tests (i.e., Mann-Whitney test or Kruskal-Wallis test followed by Dunn’s multiple comparisons tests). Data are presented as means ± SEM. All statistical analysis were done using GraphPad Prism 7 software, unless stated otherwise. P values less than 0.05 were considered statistically significant.

### Study approval

All animal experiments were approved by the University of Haifa Animal Care and Use Committee committee (license numbers 437, 488, 631, 635, 642 to Dr. Nathaniel Gould) and in compliance with the National Institutes of Health guidelines for the ethical treatment of animals.

### Data availability

Data presented is available in the Supporting Data Values file. See complete unedited blots in the supplemental material. Full sized images can be found at https://neurosenblum.haifa.ac.il/ hQR2 crystal structure is available at https://www.rcsb.org/structure/7O4D The proteomic data set is available upon request from the corresponding author.

## Author contributions

NLG led the project. NLG and KR designed the research and KR supervised the research. NLG, GRS, SC, KS, HK, EE, AE, O David, MF, DT, TP, VS, MW, MM, O Dym, SA, YP, NG, IB, HS, MK, M Lalzar, M Lévesque, and GL performed the research. NLG, GRS, SC, AE, MF, DT, TP, O. Dym, M Lalzar, MA, KS, NH, BL, CS, B Shilton, B Shklyar, VS, GA, and HB analyzed the data. NLG, GRS, O. Dym, KS, SC, BL, M Lalzar, and KR wrote the paper. All authors reviewed the manuscript.

## Supplementary Material

Supplemental data

Supplemental data set 1

Supporting data values

## Version 1 08/10/2023

In-Press Preview

## Version 2 10/02/2023

Electronic publication

## References

[1] Yin F (2016). Energy metabolism and inflammation in brain aging and Alzheimer’s disease. Free Radic Biol Med.

[2] Gal-Ben-Ari S (2019). PKR: a kinase to remember. Front Mol Neurosci.

[3] Storkebaum E (2023). Messenger RNA translation defects in neurodegenerative diseases. N Engl J Med.

[4] Segev Y (2015). PKR inhibition rescues memory deficit and ATF4 overexpression in ApoE ε4 human replacement mice. J Neurosci.

[5] Sharma V (2020). eIF2α controls memory consolidation via excitatory and somatostatin neurons. Nature.

[6] Gould NL (2020). Muscarinic-dependent miR-182 and QR2 expression regulation in the anterior insula enables novel taste learning. eNeuro.

[7] Gould NL (2020). Dopamine-Dependent QR2 pathway activation in CA1 interneurons enhances novel memory formation. J Neurosci.

[8] Hayat F (2021). The biochemical pathways of nicotinamide-derived pyridones. Int J Mol Sci.

[9] Makarov MV (2021). Chemical and biochemical reactivity of the reduced forms of nicotinamide riboside. ACS Chem Biol.

[10] Gould NL (2021). Somatostatin interneurons of the insula mediate QR2-dependent novel taste memory enhancement. eNeuro.

[11] Takeuchi T (2016). Locus coeruleus and dopaminergic consolidation of everyday memory. Nature.

[12] Li SC (2001). Aging cognition: from neuromodulation to representation. Trends Cogn Sci.

[13] Jawaid A (2019). Memory decline and its reversal in aging and neurodegeneration involve miR-183/96/182 biogenesis. Mol Neurobiol.

[14] Boutin JA (2016). Quinone reductase 2 as a promising target of melatonin therapeutic actions. Expert Opin Ther Targets.

[15] Sonavane M (2020). Dihydronicotinamide riboside promotes cell-specific cytotoxicity by tipping the balance between metabolic regulation and oxidative stress. PLoS One.

[16] Rappaport AN (2015). Expression of quinone reductase-2 in the cortex is a muscarinic acetylcholine receptor-dependent memory consolidation constraint. J Neurosci.

[17] Benoit CE (2010). Loss of quinone reductase 2 function selectively facilitates learning behaviors. J Neurosci.

[18] Rouillard AD (2016). The harmonizome: a collection of processed datasets gathered to serve and mine knowledge about genes and proteins. Database (oxford).

[19] Ran FA (2013). Genome engineering using the CRISPR-Cas9 system. Nat Protoc.

[20] Johnson ECB (2020). Large-scale proteomic analysis of Alzheimer’s disease brain and cerebrospinal fluid reveals early changes in energy metabolism associated with microglia and astrocyte activation. Nat Med.

[21] Tabet N (2006). Acetylcholinesterase inhibitors for Alzheimer’s disease: anti-inflammatories in acetylcholine clothing!. Age Ageing.

[22] Ferry G (2010). Old and new inhibitors of quinone reductase 2. Chem Biol Interact.

[23] Islam F (2022). The unusual cosubstrate specificity of NQO2: conservation throughout the amniotes and implications for cellular function. Front Pharmacol.

[24] Alnabulsi S (2016). Non-symmetrical furan-amidines as novel leads for the treatment of cancer and malaria. Eur J Med Chem.

[25] Zhao Q (1997). Unexpected genetic and structural relationships of a long-forgotten flavoenzyme to NAD(P)H:quinone reductase (DT-diaphorase). Proc Natl Acad Sci U S A.

[26] Boutin JA (2019). S29434, a quinone reductase 2 inhibitor: main biochemical and cellular characterization. Mol Pharmacol.

[27] Oakley H (2006). Intraneuronal beta-amyloid aggregates, neurodegeneration, and neuron loss in transgenic mice with five familial Alzheimer’s disease mutations: potential factors in amyloid plaque formation. J Neurosci.

[28] Schepers AG (2021). Identification of NQO2 as a protein target in small molecule modulation of hepatocellular function. ACS Chem Biol.

[29] Furney SJ (2011). Genome-wide association with MRI atrophy measures as a quantitative trait locus for Alzheimer’s disease. Mol Psychiatry.

[30] Hashimoto T, Nakai M (2011). Increased hippocampal quinone reductase 2 in Alzheimer’s disease. Neurosci Lett.

[31] Brouillette J, Quirion R (2008). Transthyretin: a key gene involved in the maintenance of memory capacities during aging. Neurobiol Aging.

[32] Merhav M, Rosenblum K (2008). Facilitation of taste memory acquisition by experiencing previous novel taste is protein-synthesis dependent. Learn Mem.

[33] Phillips RG, LeDoux JE (1992). Differential contribution of amygdala and hippocampus to cued and contextual fear conditioning. Behav Neurosci.

[34] Ashkenazy H (2016). ConSurf 2016: an improved methodology to estimate and visualize evolutionary conservation in macromolecules. Nucleic Acids Res.

[35] Livingston G (2017). Dementia prevention, intervention, and care. Lancet.

[36] Deacon R (2012). Assessing burrowing, nest construction, and hoarding in mice. J Vis Exp.

[38] Uchida K (2003). 4-Hydroxy-2-nonenal: a product and mediator of oxidative stress. Prog Lipid Res.

[39] López-Otín C (2013). Hallmarks of aging: an expanding universe. Cell.

[40] Massaad CA, Klann E (2011). Reactive oxygen species in the regulation of synaptic plasticity and memory. Antioxid Redox Signal.

[41] Niki E (2016). Oxidative stress and antioxidants: distress or eustress?. Arch Biochem Biophys.

[42] Winger JA (2009). The structure of the leukemia drug imatinib bound to human quinone reductase 2 (NQO2). BMC Struct Biol.

[43] Rix U (2010). A comprehensive target selectivity survey of the BCR-ABL kinase inhibitor INNO-406 by kinase profiling and chemical proteomics in chronic myeloid leukemia cells. Leukemia.

[44] Olsson B (2014). Imatinib treatment and Aβ42 in humans. Alzheimers Dement.

[45] He G (2010). Gamma-secretase activating protein is a therapeutic target for Alzheimer’s disease. Nature.

[46] Boutin JA (2008). Studies of the melatonin binding site location onto quinone reductase 2 by directed mutagenesis. Arch Biochem Biophys.

[47] Mierzejewska P (2021). An unusual nicotinamide derivative, 4-pyridone-3-carboxamide ribonucleoside (4PYR), is a novel endothelial toxin and oncometabolite. Exp Mol Med.

[48] Gaikwad NW (2009). Evidence for NQO2-mediated reduction of the carcinogenic estrogen ortho-quinones. Free Radic Biol Med.

[49] Giroud-Gerbetant J (2019). A reduced form of nicotinamide riboside defines a new path for NAD^+^ biosynthesis and acts as an orally bioavailable NAD^+^ precursor. Mol Metab.

[50] https://www.abstractsonline.com/pp8/#!/10619/presentation/84417.

[51] Lagomarsino VN (2021). Stem cell-derived neurons reflect features of protein networks, neuropathology, and cognitive outcome of their aged human donors. Neuron.

[52] Reybier K (2011). Insights into the redox cycle of human quinone reductase 2. Free Radic Res.

[53] Miettinen TP, Björklund M (2014). NQO2 is a reactive oxygen species generating off-target for acetaminophen. Mol Pharm.

[54] Sidrauski C (2015). The small molecule ISRIB reverses the effects of eIF2α phosphorylation on translation and stress granule assembly. Elife.

[55] Sharma V (2018). Local inhibition of PERK enhances memory and reverses age-related deterioration of cognitive and neuronal properties. J Neurosci.

[56] Rosenblum K (1993). Taste memory: the role of protein synthesis in gustatory cortex. Behav Neural Biol.

[57] Vorhees CV, Williams MT (2006). Morris water maze: procedures for assessing spatial and related forms of learning and memory. Nat Protoc.

[58] DeLano WL (2002). Pymol: an open-source molecular graphics tool. CCP4 Newsl Protein Crystallogr.

