# Supplementary material for: Specific quinone reductase 2 inhibitors reduce metabolic burden and reverse Alzheimer’s disease phenotype in mice
Source: J Clin Invest. 2023 Oct 2;133(19):e162120. doi: 10.1172/JCI162120 (PMC10541198; doi:10.1172/JCI162120)

Supplementary Methods

Cell lines

HCT116 colon carcinoma cells were a kind gift from the Vogelstein lab at the Ludwig Institute for Cancer Research, Johns Hopkins Medical School, Baltimore Maryland USA. All other cell lines were purchased from ATCC and grown at 37°C with 5% CO_2. HEK293T and HCT116 cells were cultured in DMEM (Gibco) supplemented with 10% FBS (Biological Industries), 100U/mL penicillin/streptomycin (Biological Industries) and 2mM of L-Glutamine (Biological Industries). Cells were grown in 10 mm plates until 80-90% confluency and passaged every 3-7 days at dilution ratios ranging from 1:5 to 1:30, depending on the initial density. The number of total passages did not exceed more than 5 in HCT116, or 20 in HEK293T, prior to the start of an experiment. THLE-2 (CRL-2706™) were grown in the BEGM Bullet Kit (CC-3170) from Lonza. Besides the additives contained in the kit, the medium was further supplemented with 5ng/mL EGF (Sigma), 70ng/mL phosphoethanolamine (Sigma) and 10% FBS (Biological Industries). The plates for the THLE-2 needed to be pre-coated with a mixture of 0.01mg/mL fibronectin, 0.05mg/mL of PureCol™ EZ Gel Solution (Sigma) and 0.01mg/mL of BSA dissolved in BEBM medium (Lonza). The coating medium was aspirated before seeding.

CRISPR/Cas9 Targeting of QR2

Additional details of the knockout procedure and corresponding figures have been previously published(23). Disruption of the *NQO2* gene at positions 46 and 108 within exon 4 was done using CRISPR/Cas9 dual nickase (plasmid pSpCas9n(BB)-2A-Puro (PX462), a kind gift from Dr. Feng Zhang, Broad Institute of MIT and Harvard, Cambridge Massachusetts (Addgene #48141). The target sequence was selected using a CRISPR design tool (http://crispr.mit.edu/),

which identified the guide sequences with the least off-targets. Vectors NQO24_46 and NQO24_108 were produced by cloning oligonucleotides corresponding to guide RNA (sgRNA) into PX462 as previously described(19). The vectors were then transfected into HCT116 cells (at 70% confluence) using Lipofectamine 2000 (Life Technologies), and 24 h later 0.7 µg/mL puromycin (Alfa Aesar) was added to the media for 72 h. Cells that survived were then collected and serially diluted into 96 well plates, with

puromycin supplemented media, to enable selection and expansion of plasmid containing colonies two weeks later.

Label-Free Mass Spectrometric Analysis

To prepare for mass spectrometry the parental C1 ($HCT116^{QR+}$), C3, and C5 (both $HCT1161^{QR2\Delta}$) were seeded in 10 cm plates (n=5). Cells were cultured until reaching about 80% confluency, at which point cells were collected, washed twice with PBS, lifted from plates with trypsin-EDTA (0.25%), and centrifuged at 1,000g; cell pellets were frozen at -80 °C. Frozen cell pellets were resuspended in 8 M urea, 50 mM ammonium bicarbonate (ABC), 10 mM DTT, 2% SDS and sonicated with a probe sonicator. Twenty-five µg of protein lysate, as quantified by Pierce™ 660 nm Protein Assay (ThermoFisher Scientific), was reduced in 10 mM DTT for 25 min and alkylated in 100 mM iodoacetamide for 25 min in the dark, followed by methanol precipitation. The protein pellet was resuspended in 200 µL of ABC and subjected to a sequential digest first with 250 ng of LysC (Wako Chemicals, USA) for 4 h, then 500 ng of Trypsin/LysC (Promega) for 16 h, followed by 500 ng of Trypsin (Promega) for an additional 4 h. Digestions were incubated at 37 °C with interval mixing at 600 rpm (30 s mix, 2 min pause) on a Thermomixer C (Eppendorf, catalogue #2231000667). After the last digestion, samples were acidified with 10% formic acid to pH 3 to 4 and centrifuged at 14,000g to pellet insoluble material.

Approximately 1 µg of peptide sample was injected onto a Waters M-Class nanoAcquity HPLC system coupled to an Orbitrap Elite mass spectrometer (ThermoFisher Scientific) operating in positive mode. Buffer A consisted of mass spectrometry grade water with 0.1% formic acid and buffer B consisted of acetonitrile with 0.1% formic acid (ThermoFisher Scientific). All samples were trapped for 5 min at a flow rate of 5 mL/min using 99% buffer A and 1% buffer B on a Symmetry BEH C18 Trapping Column (5 mm, 180 mm x 20 mm, Waters). Peptides were separated using a Peptide BEH C18 Column (130 A ,1.7 mm, 75 mm x 250 mm) operating at a flow rate of 300 nL/min at 35°C (Waters). Samples were separated using a non-linear gradient consisting of 1%–7% buffer B over 1 min, 7%–23% buffer B over 179 min and 23%–35% buffer B over 60 min, before increasing to 98% buffer B and washing. MS acquisition settings are provided (See table below).

Table 1. Mass Spectrometry Acquisition Settings

Instrument	Orbitrap Elite
Mass Range	400 to 1450 m/z
MS1 resolution (Orbitrap)	120K
MS1 AGC target	10^6
MS1 injection time	200 ms
Lock mass	445.120025
MS2 detection	IT
MS2 scan rate	rapid
MS2 AGC target	10^4
MS2 injection time	50 ms
Top N	20
Isolation width	2
MS2 activation	CID
Normalized collision energy	35
Dynamic exclusion	enabled
Minimum signal required	500
Exclusion duration	30s
Exclusion mass width low	0.5
Exclusion mass width high	1.5
Charge exclusion	unassigned, 1, >8

The mass spectrometry proteomics data have been deposited to the ProteomeXchange Consortium via the PRIDE partner repository with the dataset identifier PXD033944.

Differential Expression Analysis and Gene-Set Enrichment

Differential expression analysis was performed based on label-free quantitation (LFQ) intensity values calculated (see above) using Perseus (version 2.0.3.0)(59). Missing values (proteins below the detection limit) were imputed to the value of 2^{16} (Supplementary Table 1A). Then, the two QR2 KO lines and the control were pairwise compared and tested for differential expression using a non-parametric different

variance adjusted t-test. A permutational false discovery rate (FDR) procedure was done to account for false positive discovery (Supplementary Table 1B). For FDR adjusted q value <0.05 no significant difference was found between the two lines. The differentially expressed (DE) proteins included 317 proteins and 294 proteins for QR2 KO lines, C3 and C5, respectively. Of these, 182 proteins were DE in both lines. In order to compare the effect of QR2 CRISPRi between the two lines, Pearson correlation between QR2 CRISPRi vs. control log2 fold change values were calculated and tested (Pearson r=0.83, P=2.2e^{-16}). Since no significant differences were found between lines, and high Pearson correlation was found, we continued for gene-set enrichment analyses using a combined set of DE proteins from both lines. This set included 429 proteins in total, 258 with higher expression and 171 with lower expression in QR2Δ lines compared to control. In order to predict association between proteins among higher and among lower expressed proteins, a STRING(60) association network was calculated, with confidence parameter set to 0.8 (high confidence), omitting 'text mining' from the active interaction sources options and retaining only connected proteins in the network. We examined the overlap between the present set of 429 DE proteins and the list of proteins DE in DLPFC tissues of Alzheimer's disease compared to control reported in Johnson et al., (2020)(20). The latter list included 955 proteins (474 higher expressed and 478 lower expressed in AD compared to control tissues). Genes overlapping between the two lists and in opposite direction (QR2Δ down – AD up and vice versa) were examined for functional enrichment using ENRICHR(61) inspecting results from BioPlanet, KEGG and Gene Ontology databases. Functional enrichment was considered significant for FDR adjusted P value <0.05 (Supplementary Table 1C). For selected pathways with significant enrichment in the overlapping gene-set, we also verified enrichment in the dataset from the two studies separately. This was done using ENRICHR with the same criteria described above. Z-score for enrichment was calculated as ${(up - down)}/{\sqrt{N}}$ where up and down and the number of genes DE in both direction, and N is the total number of DE genes.

Western Blot

Samples in SDS sample buffer were loaded into 10-12.5% polyacrylamide gels, using equal protein quantities, and subjected to SDS-PAGE. Following electrophoresis, samples were transferred onto 0.2µm

nitrocellulose membranes (Bio-Rad) using Trans-Blot Turbo Transfer System (Bio-Rad), washed three times in TBST, blocked for 1 h in blocking buffer (Bio-Rad) and incubated overnight at 4°C with primary antibodies, including Tubulin (1:40,000, Sigma, SAB4500087), QR2 (1:1,000, Santa Cruz, sc-271665), NDUFA9 (1:500, AbCam, ab14713), CD73 (1:1,000, Cell-Signaling, D7F9A), 4-HNE (1:1000, AbCam, ab48506), amyloid β (1:1,000, AbCam, ab201060), eIF2α (1:1000, Cell Signaling, 9722) and phospho-(Ser51) eIF2α (1:1000, Cell Signaling, 9721), diluted in blocking buffer. The next day, the membranes were washed three times in TBST and incubated at room temperature (RT) for 1 h with 1:10,000 secondary antibodies conjugated to horseradish peroxidase, and following a further three times washes in TBST were immunoblotted with Westar Supernova (Cyanagen), imaged using a charge-coupled device camera and analyzed using Quantity One software (Bio-Rad).

H_2DCFDA Detection of Cellular ROS

The cells were harvested and resuspended in 1% clear medium (DMEM – with no phenol red, supplemented with 1% FBS, 1% L-Glutamine and 1% Penicillin-Streptomycin). A sterile poly-L-lysine- coated Nunc black, 96-well, clear flat-bottomed plate (Thermo Fisher Scientific) was seeded with 25,000 cells in 100 μL per well and incubated overnight. The next day, the medium was removed, and the wells carefully washed with PBS. H_2DCFDA (20 μM, Sigma-Aldrich) was added in a total volume of 100 μL to each well (a control group contained only PBS). The plate was then incubated in the dark at 37°C for 45 min. The H_2DCFDA solution was removed, and the wells were washed with PBS. After the PBS was removed, the treatments (100 μL per well) were applied. The plate was then incubated in the dark at 37°C for 3 h, before reading in a Tecan M200 Pro florescence microplate reader, using wavelengths Ex/Em = 485 nm/535 nm.

High Throughput Screen (HTS)

The enzymatic activity of QR2 (10 nM) was measured using menadione (both Sigma-Aldrich) as substrate and dihydro-benzylnicotinamide (BNAH; Tocris) as co-substrate(62). The reaction was performed in 50 mM Tris HCl, 150 mM NaCl 0.01%Tween-20 pH=8.5 (Sigma-Aldrich) at room temperature. Briefly, 3 μL of menadione alone (200 μM) or a mixture of menadione and QR2 (200 μM of

menadione and 10 nM of QR2) were dispensed in 1536-well black Nunc plates (Thermo-Fisher) using aMultidrop™ Combi Reagent Dispenser (Thermo-Fisher) and pre-incubated with compounds for 10min. BNAH (200 μM) was then added (3 μL) to the reaction using a BioNex Solutions BNX 1536 plate washer and the plates were incubated for another 10 min. Fluorescence intensity was followed in a PHERAstar FS multi-mode plate reader (BMG Labtech) using an optic module with excitation at 360 nM and emission at 470 nM. The initial HTS included approximately 200,000 compounds at a single concentration of 5 μM. The chemical libraries used were Selleck Chemicals Bioactives, the Drug-like set from Enamine, HitFinder collection from Maybridge, Spectrum Collection from Microsource, Lopac from Sigma-Aldrich and the diversity sets from ChemDiv and ChemBridge. Initial ''hit'' compounds were defined as causing >30% reduction of enzyme activity. Compounds suspected to be fluorescent were re-assayed in an absorbance assay at 350 nm using the same reaction but in a higher volume (100 μL), in a transparent plate (Greiner Bio-One). ''Hit'' compounds resulting from both the fluorescence and absorbance assay were tested in a concentration-response, and IC50 values were determined. In parallel, the same concentration response methodology was applied in a selectivity assay where QR2 enzyme was replaced by QR1 (10 nM; Sigma-Aldrich).

Synthesis of Novel QR2 Inhibitors

General

All reagents and solvents used for the synthesis were purchased from Sigma-Aldrich, Merck and Acros. Chemical building blocks were purchased from Enamine, Combi-Blocks and MolPort chemical Suppliers. Commercial reagents were used for synthesis without further purification. All solvents used for flash chromatography were HPLC grade. Reactions on microwave were done on Microwave reactor: Biotage Initiator+. Flash chromatography was performed using Merck Silica gel Kieselgel 60 (0.04-0.06 mm) or by atomized CombiFlash® Systems (Teledyne ISCO, USA) with RediSep Rf Normal-phase Flash Columns. Purification of the final compounds was performed using preparative HPLC; Waters Prep 2545 Preparative Chromatography System, with UV/Vis detector 2489, using XBridge® Prep C18 10 μm 10x250 mm Column (PN: 186003891, SN:161I3608512502). Reaction progress and compounds' purity

was monitored by Waters UPLC-MS system: Acquity UPLC® H class with PDA detector, and using

Acquity UPLC® BEH C18 1.7 µm 2.1x50 mm Column (PN:186002350, SN 02703533825836). MS-system: Waters, SQ detector 2. 1H and 13C NMR spectra were recorded on a Bruker Avance III -300 MHz, 400 MHz and 500 MHz spectrometer, equipped with QNP probe (Supplementary Table 3). Chemical shifts are reported in ppm on the δ scale and are calibrated according to the deuterated solvents. All J values are given in Hertz.

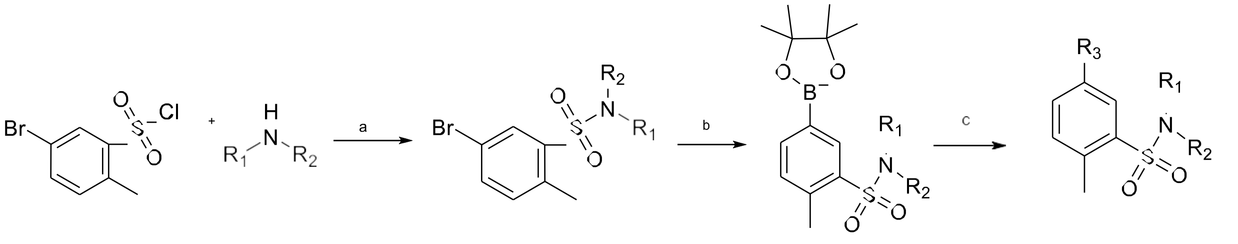

Scheme S1: **Synthesis of Novel QR2 Inhibitors**. Reaction conditions: (a) 5-bromo-2-methylbenzenesulfonyl chloride, DMAP, pyridine, desired amine (1), DCM, 0^0C, 30 min; (b) 5-bromo-2-methyl-N-benzenesulfonamide (2), Potassium acetate, bis(pinacolato)diboron, $PdCl_2$(dppf), 1,4-dioxane, 80^0C, 12h; (c) desired ((N-sulfamoyl)-4-methylphenyl)boronic ester (3), desired heterocycle with leaving group (R_3-Br/OTf), potassium carbonate, $PdCl_2$(dppf), 1,4-dioxane, 90^0C, 4-12h.

5-bromo-2-methyl-N-benzenesulfonamides *(2)*

5-bromo-2-methylbenzenesulfonyl chloride (1 eq.), DMAP (0.1 eq.) and pyridine (1.5 eq.) were added to a cooled round bottom flask with dichloromethane (0.2 M). The desired amine (1.4 eq.) was slowly added dropwise, and the mixture was stirred for 30 min. The reaction was monitored by LC-MS, after the completion of the reaction the crude mixture was washed with water, and the organic layer was washed with $NaHCO_3$ solution and brine, and then dried over Na_2SO_4. The Crude product was purified via Silica Gel chromatography (1% MeOH in DCM to 1%MeOH in Ethyl acetate gradient) to provide the products in 52-100% yield.

((N-sulfamoyl)-4-methylphenyl)boronic acids *(3)*

Potassium acetate (3 eq.), 5-bromo-2-methyl-N-benzenesulfonamide (1 eq.) and bis(pinacolato)diboron (1.2 eq.) were added to a microwave vial and dissolved in 1,4-dioxane (0.15 M). After degassing with Ar for 15 min, $PdCl_2$(dppf) (0.05 eq.) was added, and the reaction mixture was heated in a microwave at 80°C for 12 h. The reaction was monitored by LC-MS. The reaction mixture was cooled to room

temperature, and filtered through celite. The solvents were evaporated, and the crude product used as is in the next stage.

N-2-methyl-5-benzenesulfonamide ***(4)***

The desired ((N-sulfamoyl)-4-methylphenyl)boronic ester (crude) (1 eq.) ,the desired Het-Br or Het-OTf (1.5 eq.) , and potassium carbonate (3 eq.) were dissolved in 1,4-dioxane: water 2.5:1 (90 mM). The reaction mixture degassed for 15min with argon, then $PdCl_2$(dppf) (0.05 eq.) was added. The vial was heated in a sand bath to 90°C, for 4-12 h. The reaction was monitored by LC-MS. The reaction mixture was cooled to room temperature and filtered through celite, and washed several times with EtOAc. The solvents were evaporated and the crude product was purified on ISCO CombiFlash System using a silica gel column 12 g, DCM+ 1% MeOH to EA+ 1% MeOH gradient 25 min. Some of the final products were then subjected to preparative HPLC; Waters Prep 2545 Preparative Chromatography System, the solvents were evaporated to yield a range of white/off-white solids, the yield range was 2-56%.

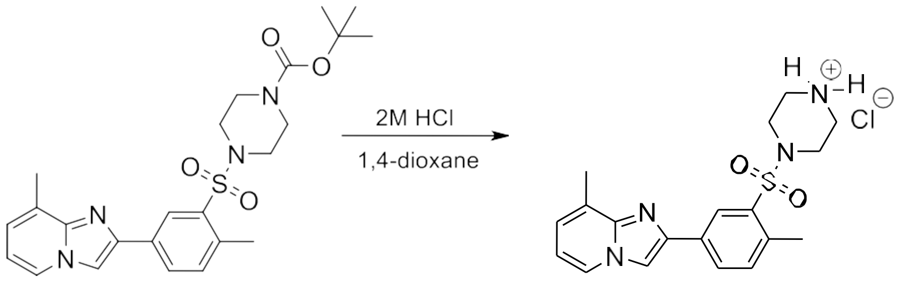

5 **6**

4-((2-methyl-5-(8-methylimidazo[1,2-a]pyridin-2-yl)phenyl)sulfonyl)piperazin-1-ium ***(6)***

Tert-butyl 4-((2-methyl-5-(8-methylimidazo[1,2-a]pyridin-2-yl)phenyl)sulfonyl)piperazine-1-carboxylate (**5**) (3.00 g, 6.37 mmol)was dissolved in 15 mL 1,4-dioxane then added to HCl 4 M in 1,4-dioxane (15 mL), and stirred for 6 h. Then, an additional HCl 4M in 1,4-dioxane (5 mL) was added and stirred for a further 12 h. The reaction was then heated to 60°C for 4 h and cooled to 0°C. The reaction mixture was diluted with 50 mL ether, to give a white solid which was filtered, and dried overnight under high vacuum to give 8-methyl-2-(4-methyl-3-(piperazin-1-ylsulfonyl)phenyl)imidazo[1,2-a]pyridine hydrochloride(**6**) with a 77.5% yield.

Cell Toxicity Assay

THLE-2 cells were exposed to the compounds in a 9-point, 2-fold dilution concentration response series with a 100 µM as upper limit, for 72 h. Following the 72 h of exposure to the test compounds, cell viability was determined by measuring the concentration of cellular ATP (CellTiter Glo, Promega). The luminescence signal was measured on a Pherastar FS multi-mode plate reader (BMG Labtech).

XTT Cell Viability Assay

HEK293FT cells were cultured with penicillin-streptomycin antibiotics in Invitrogen DMEM (Thermo Fisher Scientific) with 10% fetal bovine serum and L-glutamine, and grown to 80-90% confluence in 100 mm plates, while being passaged every 2–3 d. For the XTT assay (Biological Industries), cells were then collected, centrifuged, and resuspended in fresh Minimum Essential Medium, and seeded at 50,000 cells/well in 96- well, poly-L-lysine-coated Nunc black, clear flat-bottomed plates (Thermo Fisher Scientific). The next day, the cells were treated with increasing doses of QR2 inhibitors or vehicle for 3 h, and the XTT assay was then carried out as per the manufacturer's instructions.

Cellular Thermal Shift Assay (CETSA)

The CETSA assay was performed as described(54) with some modifications. Briefly, HEK293T cells were trypsinized (Biological Industries), washed in PBS (Sigma-Aldrich) and then suspended in PBS containing protease inhibitor cocktail (Roche). The suspended cells were then divided into two Eppendorf tubes and were treated with either compound or DMSO (Sigma-Aldrich) for 1h at 37ºC with shaking. Following treatment, each sample was divided into PCR tubes (100µL/tube) and subjected to a temperature gradient (ranging from 63 to 80ºC) for 3 minutes. Cell lysates were obtained by 3-cycles of freeze-thaw using liquid nitrogen and a thermal block set to 25ºC. Samples were then centrifuged at 15,000rpm at 4ºC for 20min and were subsequently analyzed by Western Blot of QR2 (1:100, Santa Cruz; sc-271665). The isothermal concentration response fingerprint (ITDRF) experiments were done using a constant temperature of 73ºC. Band intensities were normalized to the highest concentration and SOD1 levels (1:200, Santa Cruz; sc-17767). Analysis of the results were done in accordance to Jafari et al.(63) using GraphPad Prism software.

Cloning, Expression & Purification of QR2

Full length QR2 (1-232) with an N-terminal Avi-tag was cloned into the expression vector pET28- bdSumo. This vector was constructed by transferring the His14-bdSUMO cassette from the expression vector (designated K151) generously obtained from Prof. Dirk Görlich from the Max-Planck-Institute, Göttingen, Germany(64) into the expression vector pET28-TevH(65). Cloning was performed by the Restriction-Free (RF) method(66). The plasmid was co-transformed with pACYC184-BirA into BL21 (DE3). A 5 L culture was induced with 200 mM IPTG and grown at 15ºC ON. The culture was harvested and lysed by a cooled cell disrupter (Constant Systems) in lysis buffer (50 mM Tris pH 8, 0.5M NaCl, 20 mM Imidazole) containing 200 KU/100 mL lysozyme, 20 ug/mL DNase, 1 mM $MgCl_2$, 1 mM phenylmethylsulphonyl fluoride (PMSF) and protease inhibitor cocktail. After clarification of the soup by centrifugation, the lysate was incubated with 5 mL washed Ni beads (Adar Biotech, Israel) for 1 h at 4ºC. After removing the soup, the beads were washed 4 times with 50 mL lysis buffer. Avi-tag-QR2 eluted by incubation of the beads with 5mL cleavage buffer (50 mM Tris pH 8, 0.5 M NaCl and 0.4 mg bdSumo protease) for 2 h at RT. The soup containing the cleaved Avi-tag-RQ2 was removed, and an additional 5 mL cleavage buffer was added to the beads for 2 h at RT. The two elution solutions were combined, concentrated and applied to a size exclusion (SEC) column (HiLoad_16/60_Superdex200 prep-grade, GE Healthcare) equilibrated with 50 mM Tris 8.5, 150 mM NaCl. The pure biotinylated Avi-tag-QR2 which migrates as a single peak at 85 mL, was pooled and flash frozen in aliquots using liquid nitrogen and was stored at -80ºC.

Crystallization, Data Collection and Refinement of hQR2

Purified hQR2 was co-crystallized in the presence of FAD and YB-537, using the hanging drop vapor diffusion method and a Mosquito robot (TTP LabTech) at 19°C. The hQR2 crystals grew utilizing the precipitants 0.7 M ammonium tartrate diabasic and 50 mM Tris pH 8.5 and formed in the orthorhombic space group $P2_12_12_1$, with one dimer per asymmetric unit. Data to 2.25 Å resolution was collected in-

house, using a Rigaku RU-H3R X-ray instrument. All diffraction images were indexed and integrated using the XIA2 program(67), and the integrated reflections were scaled using the SCALA program(68). Structure factor amplitudes were calculated using TRUNCATE(69) from the CCP4 program suite. The structure of hQR2 was solved by molecular replacement with the program PHASER(70), using the hQR2 in complex with CL097 (PDB-ID code 5LBU). All steps of the atomic refinements were performed with the PHENIX.refine, Parallel PHENIX.phaser programs(71). The model was built into 2mFobs- DFcalc, and mFobs - DFcalc maps using COOT(72). The model was optimized using PDB_REDO(73), and was evaluated with MOLPROBIDITY(74). Electron density revealed unambiguous density for the bound FAD and YB-537. Details of the data collection and refinement statistics of the hQR2 structure are described in Table 2. The crystal structure was deposited in the PDB-ID code 7O4D.

Table 2. Data collection and refinement statistics for *h*QR2

**Data Collection**	
PDB code	7O4D
Space group	$P2_12_12_1$
Cell dimensions:	
a,b,c (Å)	57.03 83.36 106.49
α,β,γ (˚)	90, 90, 90
No. of copies in a.u.	2
Resolution (Å)	47.07-2.25
Upper resolution shell (Å)	2.33-2.25
Unique reflections	24,796 (2,419)
Completeness (%)	99.90 (99.38)
Average I/σ(I)	8.82 (3.51)
R-pim	0.06999 (0.2235)
**Refinement**	
Resolution range (Å)	47.07-2.25
No. of reflections (I/σ(I) > 0)	24,777
No. of reflections in test set	2419
R-working / R-free	0.1748 / 0.2256
No. of protein atoms	3599
No. of water molecules	116
Overall average B factor ($Å^2$)	19.24
Root mean square deviations:	
- bond length (Å)	0.011
- bond angle (°)	1.54
**Ramachandran Plot**	

Most favored (%)	96.71
Additionally allowed (%)	2.85
Disallowed (%)	0.44

* Values in parentheses refer to the data of the corresponding upper resolution shell

Subjects

Male Sprague Dawley rats 225-400g (Envigo); 8 week-old, 20-35g C57BL/6 male mice (Envigo); and male and female, 8-9 month old 5xFAD mice (The Jackson Laboratory stock #034840-JAX) were used. All animals were housed in the University of Haifa core facilities, in a temperature controlled environment (22-24), on a 12h light/12h dark cycle (light phase 07:00-19:00), with food and water provided ad libitum. All cages were enriched with cotton wool bedding and sections of piping, to provide additional hiding and nesting areas within the cage. All experiments were approved by the University of Haifa Animal Care and Use committee (license numbers 437, 488, 631, 635, 642). Animals were given 7 days of acclimatization before experimentation, and during the entire period, animals were handled in accordance with University of Haifa practices and standards, in compliance with the National Institutes of Health guidelines for the ethical treatment of animals.

Blinding Measures for Animal Experiments

For double blind experiments with animals, one experimenter prepared inhibitors and vehicle (both completely translucent liquids resembling water). Both vehicle and inhibitors were made using the same volume in identical vessels, once a week, and labelled with a code (i.e. – bottle AY, bottle XZ). A second experimenter then received bottles A and B, blind to their identity, and moved their contents into bottles with a different code, to which the first experimenter was blind (i.e. – bottle 1, bottle 2). Finally, another experimenter/s blind to the identity of either codes was/were given bottles AY and XZ, gave the animals the inhibitors and vehicle and carried out the behavioral experiments and analysis. Once data was collected and analyzed, the animal and treatment identity was revealed. Subsequent immunohistochemistry image analysis was done by another experimenter, blind and unaware of any of the groups, treatments, sex or other identities of the subjects.

Microinjections

Rats

Anesthesia was done with 0.3 mL/100 g body weight equithesin (2.12% $MgSO_4$, 10% ethanol, 39.1% 1,2-propanolol, 0.98% sodium pentobarbital, and 4.2% chloral hydrate (Sigma-Aldrich). Using a stereotaxic device (Stoelting), 10 mm, 23-gauge steel guide cannulas were bilaterally installed over the anterior insular cortex (aIC), according to the coordinates (with reference to bregma): AP 1.2 mm, ML ±5.5 mm, DV 5.5 mm(75). Acrylic dental cement was applied to the cannulas as well as over two anchoring screws fastened to the skull, in order to fix the cannulas in place. A 7 day period of recovery was then given to the rats, within which time they received antibiotics (0.5mg/kg of Baytril©, enrofloxacin) and analgesic treatment (0.5 mg/kg norocarp) for the 3 days following surgery. In order to infuse QR2 inhibitor YB-808 20 min prior to taste learning, the guide cannula stylus was removed and a 28-gauge injection cannula inserted, up to 0.5mm beyond the end of the guide cannula. The injection cannula was fitted with PE20 tubing to a Hamilton microsyringe and 1 µL of vehicle (0.002% DMSO) or YB-808 (20 µM) was delivered at 0.5 µL/min. In order to prevent withdrawal of the injected content from the injection site, cannulas were kept in place for an additional minute prior to removal.

Mice

Mice were anesthetized under 2% isoflurane, using an induction box (HME109, Highland Medical Equipment). They were placed in a stereotaxic device (Kopf Stereotaxic Alignment System, model 1900) under continuous 1% isoflurane anesthesia. Guide cannulas were implanted bilaterally to CA1 (from bregma: −1.9 mm AP, ±1.4 mm, −1.6 mm DV), cemented to the skull and fitted with 28-gauge dummy cannulas extending 0.2 mm beyond the tip of the 1.2 mm guide cannulas. The mice were allowed at least 7 days of recovery before experimentation. The QR2 inhibitor YB-537 was dissolved in saline (0.9%). A total of 1µL of 5µM of YB-537 or vehicle was infused bilaterally to CA1, via a 28-gauge infusion cannula projecting 0.4 mm (drug delivery depth bregma: −1.6 mm DV) beyond the guide cannula, connected by polyethylene tubing to a 10µL syringe (Hamilton) over the course of 1min. The injection cannula was

kept for 60s inside the guide cannula in order prevent osmotic seepage of the doses upward through the cannula tract. Twenty minutes following the injection, animals underwent delay fear conditioning. Following experimentation, animals were killed, brains were sliced in coronal sections, and cannula implantation was validated by imaging.

Behavior

Incidental Taste Learning

In this paradigm, the innate neophobic response exhibited toward a new, unfamiliar taste is utilized. Taste neophobia is highly conserved across species, as it is the first line of defense against potentially poisonous, newly discovered foodstuffs. Hence, when an animal first encounters a new taste, it consumes very little. However, once a memory for the taste is formed and no associated ill effect is learned, the taste becomes familiar and the animal consumes it more freely, especially if it is a pleasant, palatable taste. Thus, the stronger the memory for the safe, palatable taste, the more likely the animal will consume it(32, 56). Therefore, in order to test whether the QR2 inhibitors could help rats remember a novel taste and enhance their memory for it, they were cannulated to the anterior insular cortex, which is necessary for taste memory formation and within which is the primary gustatory cortex(76), and allowed 7 days to recover. Then, rats were taught to drink from two pipettes each filled with 10 mL of water during a 20 min period, over the course of 3 days. On the fourth day, they were given a novel, palatable taste (two pipettes each filled with 10 mL of 0.3% NaCl), 30 min following a local microinfusion of YB-808 or vehicle. On the fifth day, rats were once again given water in the pipettes. On the sixth day, the rats were presented with a choice test, in which they are given two pipettes of water and two pipettes of NaCl (10 mL in each pipette). The memory for the novel taste is then assessed following 20min of liquid consumption, by calculating a preference index thus: [novel taste consumed/(novel taste consumed+water consumed)]*100.

Delay Fear Conditioning

This conditioning paradigm involves hippocampal CA1 dependent learning of the context, and amygdala-dependent learning of the cue(33). Mice were transported to the conditioning room, which is lit by red light

only, and kept there for 2 min. They were then placed inside a Habitest Operant Cage, within a Habitest Isolation Cubicle (Coulbourn), on a modular shock floor made of 16 metal grids, connected to Precision Animal Shockers (Coulbourn) with illumination inside the cage coming from a 20 W bulb. Themice were given 2 min to explore, during which baseline freezing was measured. Then, a 20 s, 4 kHz, 80 dB tone was given, co-terminating with the start of a 2 s, 0.5 mA foot-shock, which was repeated a further two times, each having 1 min interval. Following the third, and last of these bouts, 1 min was given prior to the animals being removed from the chambers. Mice, which have now associated the foot shock with the tone and the context can be tested for both hippocampal- or amygdala-dependent memory, the latter of which provides an internal control, as mice were only microinjected with YB-537 to the hippocampus, and not the amygdala. The next day, the animals were returned to the room under the same conditions, and were placed back into the chambers, and freezing to the context was measured over the course of 5 min. The next day, the mice were once again returned to the conditioning room, except the room was lit with white light, while the chambers were darkened, the chamber floor was covered with a flat, smooth plastic cover, one of the walls was fitted with a paper sticker and the chamber was scented with diluted (10%) window cleaner (Sano). In this unfamiliar context, the protocol from the first day was repeated, except without the foot-shocks. Freezing for the cue was recorded, staring with the sounding of the first tone. All measurements were taken with a Sentec stc-tb33usb-at camera, and analysis was done with Freeze Frame software (Actimetrics).

Morris Water Maze

Morris water maze (MWM) was carried out as previously described(58). At the same time each consecutive day, mice were placed in the dimly lit room containing the maze for 10 min, within cages containing dry bedding (not the home cages). The maze was obscured by a non-light-permeable curtain. The maze consisted of a 1.2 m pool, with RT (21-23 °C) water colored light grey, to hide the submerged (under 1-2 cm) transparent, 10 cm in diameter plastic escape platform, which was placed at the south- western quadrant of the pool. Mice were trained 4 times a day, using 60 s trials every 30 min during which they were placed into the pool, each time from a different quadrant, and allowed to swim and find the escape platform. Upon reaching the platform, mice were removed from the pool. If the mice failed to find the pool within 60 s,

they were carefully placed on the escape platform and held there for 15 s prior to being taken out of the pool. All trials were filmed with a video tracking system using EthoVision 14 (Noldus Information Technology), and escape latency (time to find the submerged escape platform) was determined by manual video analysis (due to automated detection settings unable to discern all mice coat colors – black, white and brown - against the opaque water).

Open Field

Mice were individually placed within a cage and taken to a dimly lit room containing an open field arena 50 x 50 cm in size. They were given 10 min prior to being placed within the arena, where for a period of 5 min they were allowed to explore. Two weeks later, this was repeated. The mice were filmed with an Ikegami ICD-49E camera with EthoVision 14 (Noldus Information Technology). The floor was either white or black, depending on mouse coat color, to allow automatic analysis of movement parameters, apart from rearing which was manually counted.

Novel Object Recognition

Following the first exploration of the open field arena (described above), mice were returned to the same arena the next day, while the arena now contained two identical objects. Mice were allowed to explore the objects and the arena 3 times for 10 min, with an inter trial interval of 10 min. The following day one of the objects was replaced with a novel object, and the mice were returned to the arena and allowed to explore for 10 min. Mouse movement, exploration and nuzzling was automatically recorded with an Ikegami ICD-49E camera with EthoVision 14 (Noldus Information Technology). Discrimination of the novel object was assessed by calculating (time exploring novel object – time exploring familiar object)/(time exploring novel object + time exploring familiar object).

Nesting

Each home cage was given six identical portions of cotton wool tubes and 24 h later the nest made was photographed from above. The images were given the names of the respective cages, which were coded

(see ‘Blinding Measures for Animal Experiments’ section), and were analyzed by an experimenter that was blind to the conditions, groups and cage codes. Scores were given at a scale of 1 to 5, as previously reported(37).

Immunohistochemistry

A week after the completion of all behavioral experiments, 5xFAD mice were anesthetized with isoflurane, and once fully anesthetized, were transcardially perfused with 4% paraformaldehyde (PFA), dilutes in 0.1% phosphate buffered saline (PBS, Sigma-Aldrich). Brains were then briefly removed and placed in chilled, 4% PFA for 48 h, followed by immersion in 30% sucrose in 0.1 M PBS for a further 48 h. Brains were then stored at -80°C, until they were sliced into 40µm coronal sections using a Leica CM 1950 cryostat. Slices were then washed x3 in PBS and blocked for 1 h at RT using 10% normal donkey serum (DNS) and 0.2% triton (Sigma-Aldrich) in PBS. Antibodies, including 4-HNE (1:500, AbCam, ab48506), Iba1 (1:2,000, AbCam, ab5076), GFAP (Abcam, ab53554), phospho-tau (1:1,000, Thermo, MN1020) and amyloid β (1:1,000, AbCam, ab201060) diluted in PBS with 10% DNS were incubated at 4°C overnight. The next day, the slices were washed x3 times in PBS, and secondary antibodies including donkey anti goat Alexa Fluor 568 (AbCam, ab175704), donkey anti-mouse Cy 5 (Jackson Immuno Research, 715-175-151) and donkey anti-rabbit DyLight 488 (AbCam, ab98488) all diluted 1:500 in PBS with 1% BSA, were applied to the slices at RT for 2 h. Following the incubation, the slices were washed x3 times in PBS, mounted onto glass slides, were uniformly covered in DAPI containing Vectashield (H-1200) and coverslips were added. Images were then taken using a confocal microscope (Olympus IX83), with identical acquisition parameters across every sample per antibody. Three slices were used per mouse per antibody. Tiling images were taken of the dorsal CA1 (Bregma: -1.58mm to -2.30mm; with each antibody having a slice from anterior, medial and posterior regions of this range) using a x20 objective, acquiring a Z-stack of the whole section.

Image Analysis

Analysis of the images was done blind, by an experimenter unaware of the experimental conditions or details, using Imaris (Bitplane) software. Surface reconstruction module was used to extract the data as volumes and / or signal intensities of the used markers (4-HNE, phospho-tau, amyloid β and Iba1).

Marker volume and intensity were normalized to the corresponding brain volume. This was done in order to normalize the values to variations in Z-stack volumes and/ or changes to shape and size that needed to be performed during analysis due to debris, vasculature, corpus callosum elimination, or any other obstructions or changes in shape. The normalized value was averaged for each triplicate and presented here.

Amyloid β 42 Measurement

Following completion of treatment with YB-537 or vehicle for 1 month in drinking water, mice were sacrificed, and brains were quickly removed and placed on ice. Brains were then bisected, and the cerebellum removed. Both hemispheres were then flash frozen in liquid nitrogen, with one hemisphere being used for Amyloid β 42 detection and the other used for WB. For Amyloid β 42 detection the hemisphere was first weighed, and then homogenised on ice using 1 mL of 0.1% Triton X-100 in TBS with complete protease inhibitor (Roche) and phosphatase inhibitor cocktail (Roche). Homogenates were then centrifuged at 100,000g for 1 h at 4° C, and supernatant containing the soluble fraction of Amyloid β 42 was collected and stored at -20° C overnight. The pellet, containing insoluble Amyloid β 42 was re-suspended in 1 mL of 5M guanidine HCl, placed on a rocker at RT for 2h, and then stored overnight at 4° C, with later storage being done at -20° C. The insoluble fraction was then diluted 1:5 in TBS, and centrifuged for 30 min at 13,000g and 4° C. The resulting supernatant was then used to quantify the insoluble fraction. Protein determination was done on both soluble and insoluble fractions thus prepared, using a BCA kit (Thermo). Human Amyloid (aa1-42) Quantikine ELISA Kit was then used as per the manufacturer's instructions (R&D Systems, DAB142). Results obtained were then quantified as ng Amyloid β 42 / mL sample / brain hemisphere weight.

Statistical Analysis

Experimental grouping was randomly allocated, in both rats and mice. The size for each group was based on previously published results by means of similar methods, with the use of an online power calculator (https://www.stat.ubc.ca/~rollin/stats/ssize/n2.html). Shapiro-Wilk normality tests were done for the collected data. Analysis of normally distributed data was done using parametric tests (i.e. - unpaired students t-test, one-way ANOVA followed by Tukey's or Sidak post hoc analysis) and for data not normally

distributed, non-parametric tests (i.e. - Mann-Whitney tests or Kruskal-Wallis followed by Dunn's multiple comparisons tests). Data are presented as means with SEM. All statistical analysis were done using GraphPad Prism 7 and 9 software, unless stated otherwise.

Supplementary Figures and Tables

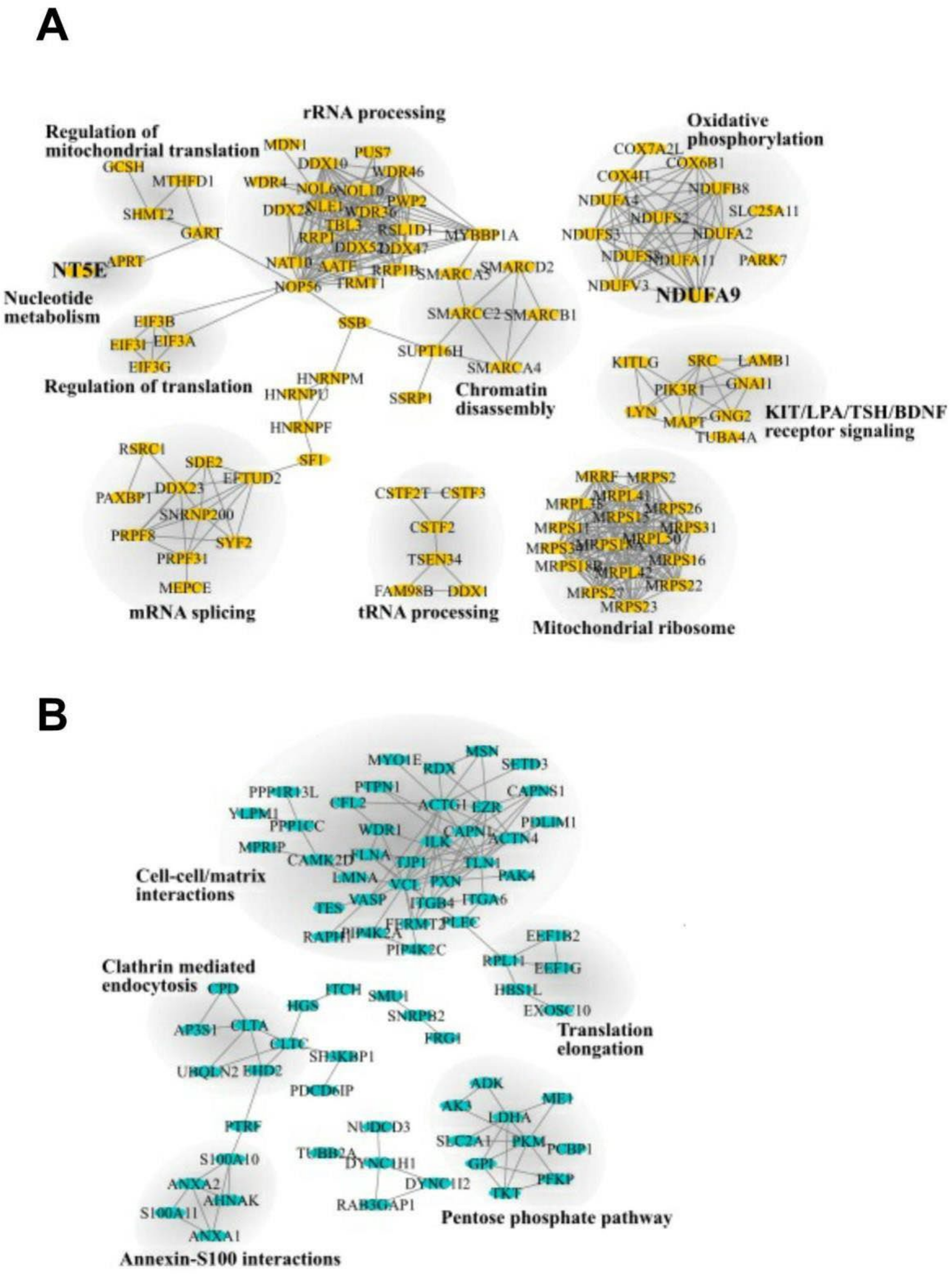

Supplementary Figure 1. Proteome of QR2Δ HCT116 Cell Lines Opposingly Overlaps Key Pathways in Alzheimer's Disease.

STRING association network for 258 higher expressed **(A)** and 171 lower expressed **(B)** proteins between QR2Δ and control (Confidence ≥ 0.8). Proteins used later for verification are marked in boldface.

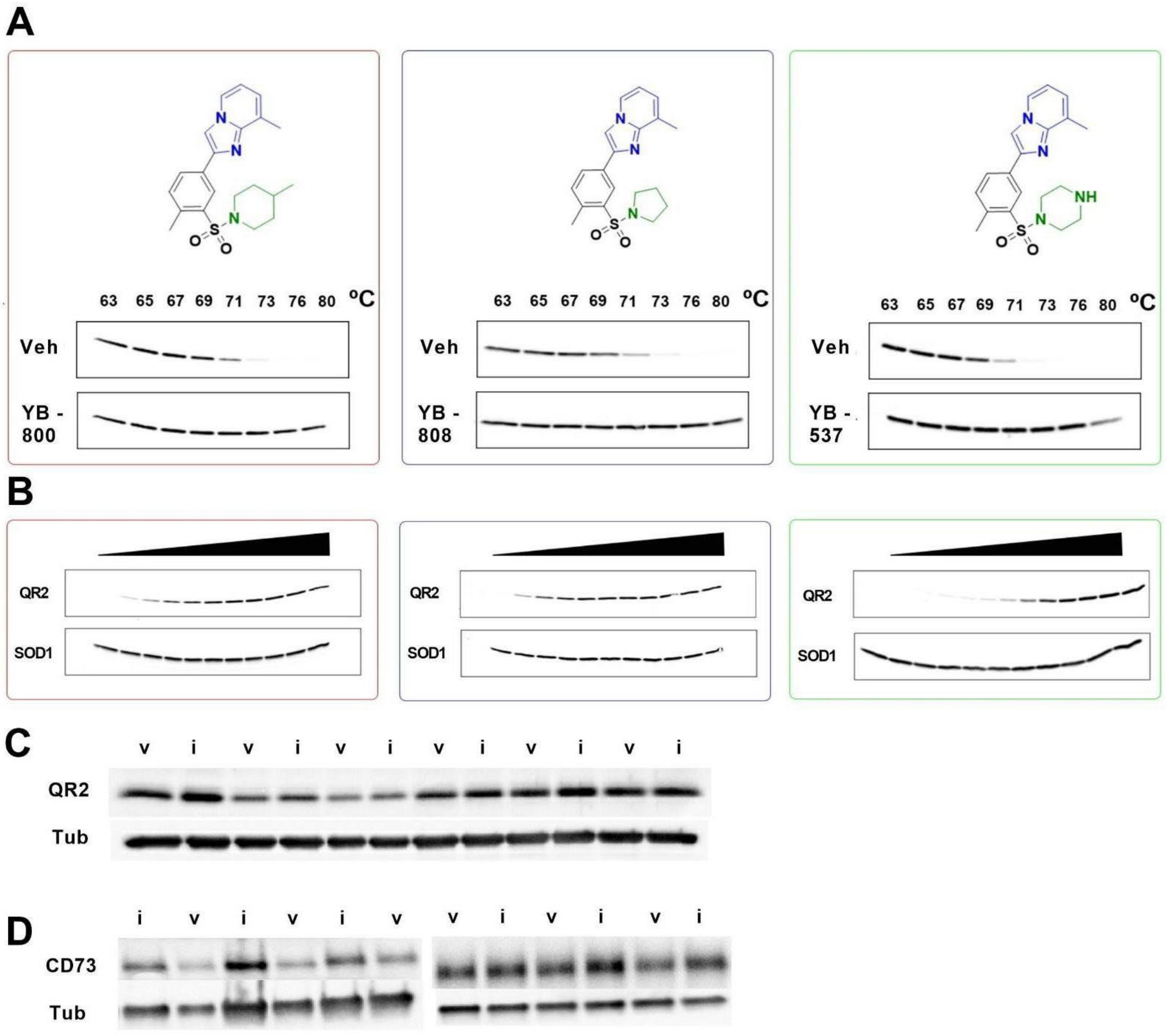

Supplementary Figure 2. Novel Inhibitors Stabilize QR2 in Cellular Thermal Shift Assays and Increase CD73 Expression Similarly to QR2 KO.

(A) QR2 thermal aggregation curves in HEK293T cells showing increased levels of solubilized receptor at elevated temperatures in the presence of different inhibitors (5 μM concentration) versus vehicle.

(B) Amount of stabilized soluble QR2 following exposure to 73ºC, in the presence of increasing concentrations of the different compounds (western blot data for QR2 as well as corresponding SOD1 levels).

(C) Western blot images of QR2 and Tubulin from HCT116 lysates following 4 d incubations with YB-800 (2 μM)**.**

(D) Western blot images of CD73 and Tubulin from HCT116 lysates following 4 d incubations with YB-800 (2 μM)**.**

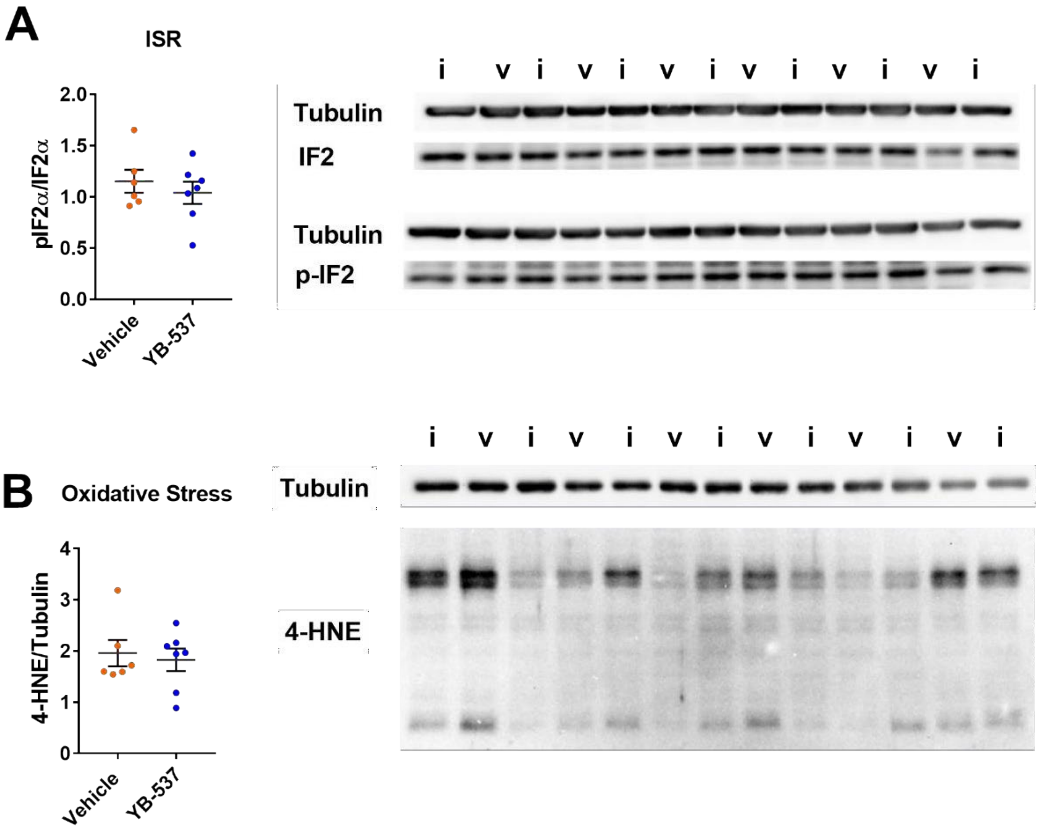

Supplementary Figure 3. Acute QR2 Inhibition in the Brains of Healthy 3–4-Month-Old C57BL/6 Mice Does Not Alter Markers of Metabolic Stress.

(A) Phosphorylation levels of eIF2α in the CA1 formation of the dorsal hippocampus are unaltered (unpaired t test, p=0.4871) 3 h following local microinjection of 5 μM YB-537, compared to controls.

(B) 4-HNE is unchanged (unpaired t test, p=0.7014) in the CA1 formation of the dorsal hippocampus 3 h following local microinjection of 5 μM YB-537, compared to controls.

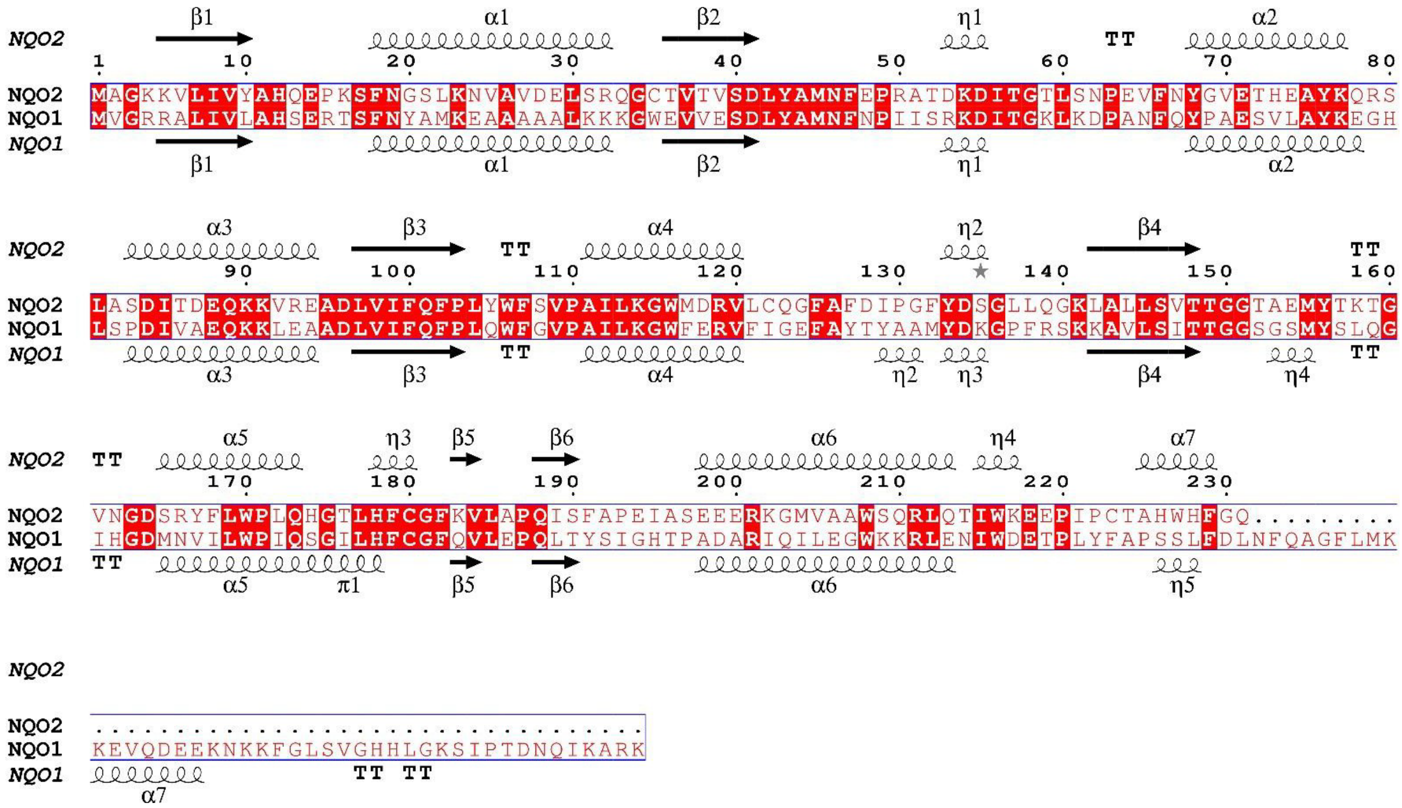

Supplementary Figure 4. Comparison of Human QR2 and QR1 Amino-Acid Sequence and Structural Motifs.

Secondary structure elements of QR2 are labeled above- and of QR1 below the corresponding sequence; α and η-helices are spirals and β-strands are arrows. The residues conserved in both proteins are in red blocks. The letter 'T' stands for turn. The sequence alignment was performed using MultAlin(77). The figure was created using ESPript(78).

A **Plasma**

Sample	Administration	Dose, mg/kg	Pharmacokinetic Parameters							
			Tmax, min	Cmax, ng/ml(g)	$AUC_{0 \to t\ min}$ (AUClast) ng*min/ml(g)	$AUC_{0 \to \infty}$ (AUCINF_obs) ng*min/ml	$T_{1/2}$ (HL_Lambda_z), min	K_{el} (Lambda_z), min^{-1}	V_d (Vz_obs) ml/kg	Bioavailability, %
Plasma	IV	10	-	2010	47400	52000	16.6	0.0417	5000	**82**
	PO	50	15.0	2030	200000	213000	60.2	0.0115	ND	

B **Brain**

Animal	Administration	Dose, mg/kg	Pharmacokinetic Parameters							
			Tmax, min	Cmax, ng/g	$AUC_{0 \to t=240min}$ (AUClast) ng*min/g	$AUC_{0 \to \infty}$ (AUCINF_obs), ng*min/g	$T_{1/2}$ (HL_Lambda_z), min	K_{el} (Lambda_z), min^{-1}	MRT (MRTlast), min	MRT (MRTinf), min
Mice	PO	50	60.0	203	30500	33100	63.5	0.0109	67.5	88.2

Supplementary Figure 5. Pharmacokinetics of YB-537 Oral and Intravenous Administration.

(A) YB-537 has 82% bioavailability when administered p.o. 50mg/kg in male mice, with a clearance rate half-life of 60.2 min.

(B) YB-537 arrives at the male mouse brain, peaking 1 h following p.o. administration (203 ng/g), and showing a clearance half-life of 63.5 min.

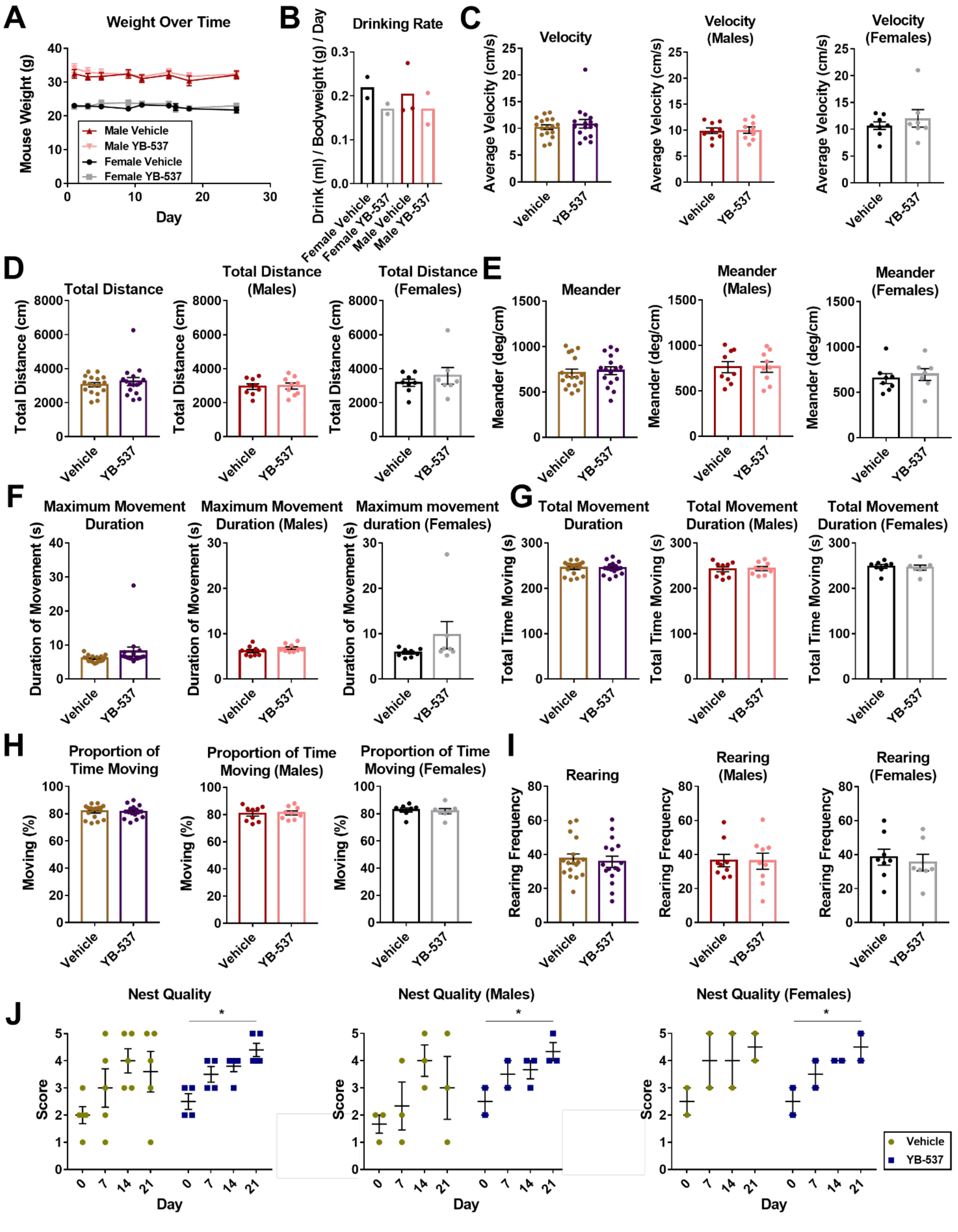
A
Weight Over Time
Mouse Weight (g)
Day
Male Vehicle
Male YB-537
Female Vehicle
Female YB-537
B
Drinking Rate
Drink (ml) / Bodyweight (g) / Day
Female Vehicle
Female YB-537
Male Vehicle
Male YB-537
C
Velocity
Velocity (Males)
Velocity (Females)
Average Velocity (cm/s)
Vehicle
YB-537
D
Total Distance
Total Distance (Males)
Total Distance (Females)
Total Distance (cm)
E
Meander
Meander (Males)
Meander (Females)
Meander (deg/cm)
F
Maximum Movement Duration
Maximum Movement Duration (Males)
Maximum movement duration (Females)
Duration of Movement (s)
G
Total Movement Duration
Total Movement Duration (Males)
Total Movement Duration (Females)
Total Time Moving (s)
H
Proportion of Time Moving
Proportion of Time Moving (Males)
Proportion of Time Moving (Females)
Moving (%)
I
Rearing
Rearing (Males)
Rearing (Females)
Rearing Frequency
J
Nest Quality
Nest Quality (Males)
Nest Quality (Females)
Score
Day
*
Vehicle
YB-537

Supplementary Figure 6. Chronic Drinking of 50 mg/kg YB-537 for of 1 Month Causes no Changes in General Mouse Physiology or Behavior but Improves Nesting Behavior Over Time.

(A) Weight of male and female, 8–9-month-old 5xFAD mice over the course of 25 days while consuming YB-537 or water (average weights – Females Vehicle 22.61 ± 0.167 g; Females YB-537 22.76 ± 0.192 g; Males Vehicle 31.72 ± 0.256 g; Males YB-537 32.56 ± 0.279 g).

(B) Drinking rate of water or YB-537 in the cages of the 5xFAD mice. Rate is calculated using the total weight of the mice in the cage, and volume drank over time, per cage (average drinking rate – Females Vehicle 0.219 ± 0.24 ml/g/day; Females YB-537 0.170 ± 0.011 ml/g/day; Males Vehicle 0.204 ± 0.035 ml/g/day; Males YB-537 0.170 ± 0.035 ml/g/day).

(C) No change is seen in the average velocity of 5xFAD mice, regardless of sex (both sexes – Vehicle 10.25 ± 0.442 cm/s, n=17; YB-537 10.9 ± 0.800 cm/s, n=16; Mann-Whitney test, p=0.8173; Males – Vehicle 9.88 ± 0.552 cm/s, n=9; YB-537 10.01 ± 0.611 cm/s, n=9; unpaired t test, t=0.1609 df=16, p=0.8742; Females – Vehicle 10.67 ± 0.713 cm/s, n=8; YB-537 12.03 ± 1.625 cm/s, n=7; Mann-Whitney test, p=0.7789).

(D) No change is seen in the total distance traveled by 5xFAD mice, regardless of sex (both sexes – Vehicle 3051 ± 129.8 cm, n=17; YB-537 3244 ± 237.9 cm, n=16; Mann-Whitney test, p=0.8173; Males – Vehicle 2944 ± 163 cm, n=9; YB-537 2983 ± 179.3 cm, n=9; unpaired t test, t=0.1572 df=16, p=0.8770; Females – Vehicle 3170 ± 209.5 cm, n=8; YB-537 3581 ± 484.5 cm, n=7; Mann-Whitney test, p=0.7789).

(E) No change is seen in the meandering of 5xFAD mice, regardless of sex (both sexes – Vehicle 708.9 ± 42.28 deg/cm, n=17; YB-537 733.4 ± 42.8 deg/cm, n=16; Mann-Whitney test, p=0.5334; Males – Vehicle 760 ± 61.19 deg/cm, n=9; YB-537 762.2 ± 58.17 deg/cm, n=9; unpaired t test, t=0.02672 df=16, p=0.9790; Females – Vehicle 651.4 ± 54.55 deg/cm, n=8; YB-537 696.3 ± 65.26 deg/cm, n=7; unpaired t test, t=0.5324 df=13, p=0.6035).

(F) A trend toward increased maximum movement bout duration of 5xFAD mice is seen when ingesting YB-537 (both sexes – Vehicle 5.996 ± 0.238 s, n=17; YB-537 8.061 ± 1.322 s, n=16; Mann-Whitney test,

p=0.0643; Males 6.169 ± 0.3541 s, n=9; YB-537 6.804 ± 0.2919 s, n=9; unpaired t test, t=1.385 df=16, p=0.1851; Females – 5.803 ± 0.324 s, n=8; YB-537 9.677 ± 3.012 s, n=7; Mann-Whitney test, p=0.2319).

(G) No change is seen in the total movement duration of 5xFAD mice, regardless of sex (both sexes – Vehicle 244.9 ± 3.544 s, n=17; YB-537 244.3 ± 3.402 s, n=16; Mann-Whitney test, p=0.6827; Males – Vehicle 241.9 ± 5.474 s, n=9; YB-537 243.4 ± 4.5 s, n=9; unpaired t test, t=0.218 df=16, p=0.8302; Females – Vehicle 248.3 ± 4.4 s, n=8; YB-537 245.5 ± 5.568 s, n=7; unpaired t test, t=0.3967 df=13, p=0.6981).

(H) No change is seen in the proportion of time moving in 5xFAD mice, regardless of sex (both sexes – Vehicle 81.63 ± 1.181 %, n=17; YB-537 81.44 ± 1.134 %, n=16; Mann-Whitney test, p=0.6827; Males – Vehicle 80.62 ± 1.825 %, n=9; YB-537 81.13 ± 1.5 %, n=9; unpaired t test, t=0.218 df=16, p=0.8302; Females – Vehicle 82.76 ± 1.467 %, n=8; YB-537 81.84 ± 1.856 %, n=7; unpaired t test, t=0.3966 df=13, p=0.6981).

(I) No change is seen in hind-limb rearing frequency of 5xFAD mice, regardless of sex (both sexes – Vehicle 37.38 ± 2.86, n=17; YB-537 35.72 ± 3.292, n=16; unpaired t test, t=0.3828 df=31, p=0.7045; Males – Vehicle 36.39 ± 3.666, n=9; YB-537 36.06 ± 4.703, n=9; Mann-Whitney test, p=0.9125; Females – Vehicle 38.5 ± 4.703, n=8; YB-537 35.29 ± 4.894, n=7; unpaired t test, t=0.4727 df=13, p=0.06443).

(J) Nest quality was significantly correlated to time under treatment for the YB-537 group, but not with controls, regardless of sex (both sexes – Vehicle Pearson r, p=0.1392; YB-537 Pearson r, p=0.0241; Males – Vehicle Pearson r, p=0.2650; YB-537 Pearson r, p=0.0345; Females – Vehicle Pearson r, p=0.1056; YB- 537 Pearson r, p=0.0173).

Data are shown as mean ± SEM**.** *p<0.05

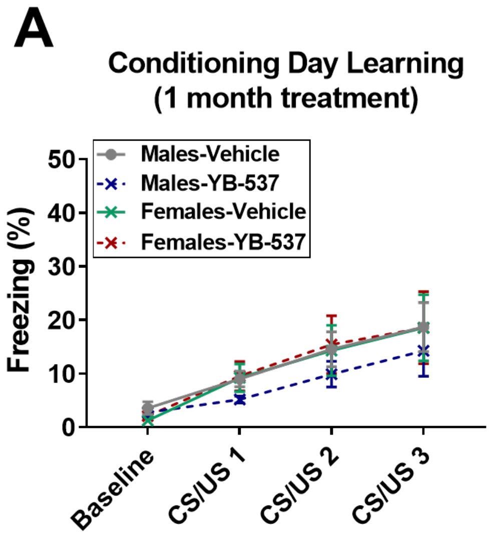

Supplementary Figure 7. 9-Month-Old 5xFAD Mice Drinking YB-537 or Vehicle Show Normal Behavioral Learning Outcomes During Fear Conditioning.

Male and female 5xFAD mice, with or without YB-537 in drinking water, show normal learning during delay fear conditioning, with no difference observed across groups (Two-Way RM ANOVA, Trial: p<0.0001, Groups: p=0.7917).

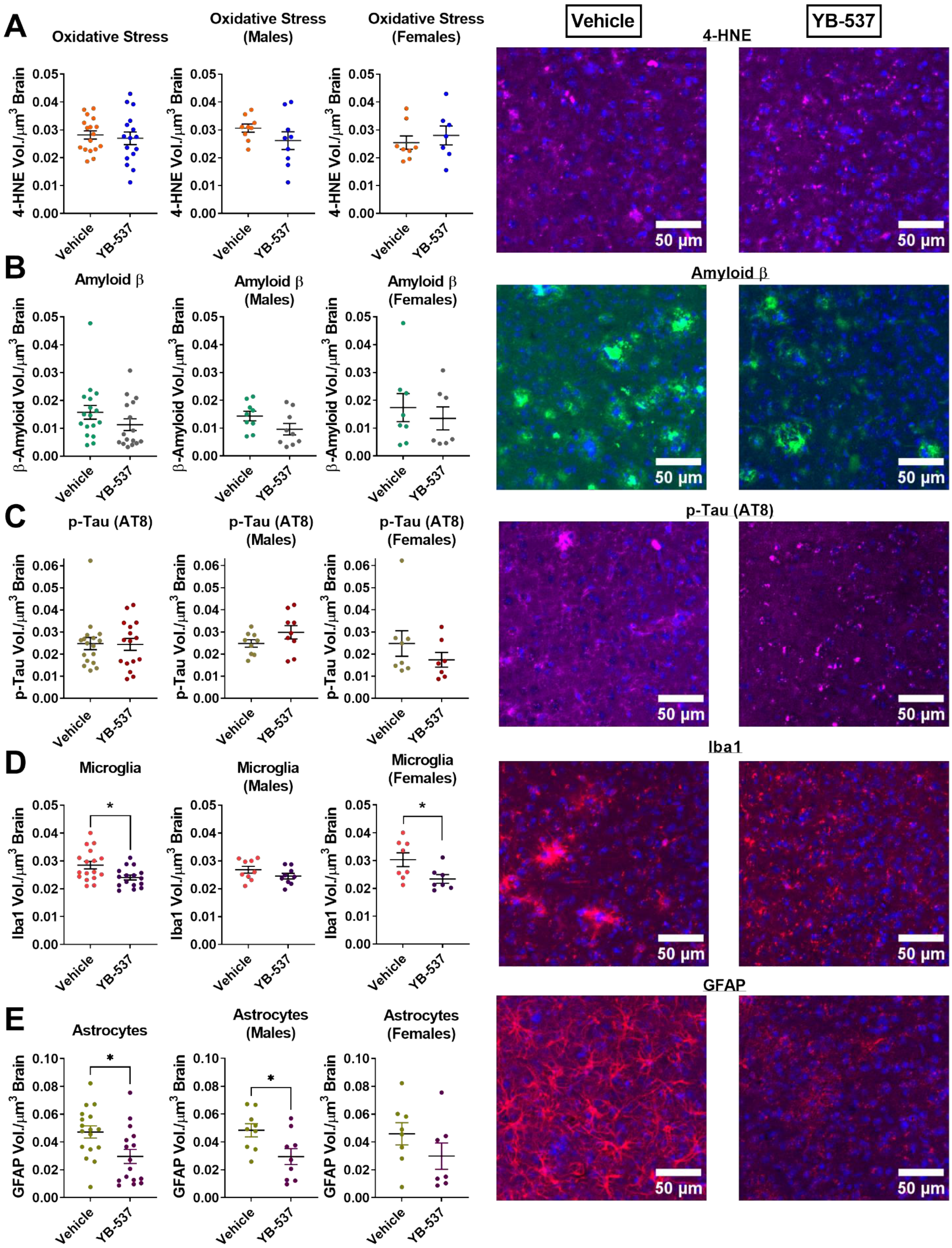

Supplementary Figure 8. Drinking YB-537 for 1 Month Significantly Reduces Brain Pathologies Associated with

Dementia in the Cortex of 9-Month-Old 5xFAD Mice.

(A) Oxidative stress, as indicated by 4-HNE, is not significantly altered in the total population of 5xFAD mice cortex following 1 month of drinking YB-537 (both sexes, unpaired t test, p=0.6535), or within the male (unpaired t test, p=0.2202) and female (unpaired t test, p=0.5356) populations.

(B) Amyloid β is not significantly reduced in the total population of 5xFAD mice cortex following 1 month of drinking YB-537 (both sexes, Mann-Whitney test, p=0.1463), is insignificantly reduced in the male population (unpaired t test, p=0.0958), and is unchanged in the female population (Mann-Whitney test, p=0.6943) populations.

(C) p-Tau is unchanged following 1 month of drinking YB-537 in the cortex of the total 5xFAD mouse population (Mann-Whitney test, p=0.8173), as well as male (unpaired t test, p=0.1652) and female (Mann-Whitney test, p=0.3357) populations.

(D) Iba1 is significantly reduced in 5xFAD mouse cortex following 1 month of drinking YB-537 in the total population (unpaired t test, p=0.0109), is insignificantly reduced in the male population (unpaired t test, p=0.1740), and is significantly reduced in the female population (unpaired t test, p=0.0389).

(E) GFAP is significantly reduced in 5xFAD mouse cortex following 1 month of drinking YB-537 in the total population (unpaired t test, p=0.0128), and also in the male population (unpaired t test, p=0.0217), while it is not significantly reduced in the female population (unpaired t test, p=0.2136).

N for all experiments: YB-537, 16 (9 males and 7 females); Vehicle, 17 (9 males and 8 females).

Data are shown as mean ±SEM**.** *p<0.05

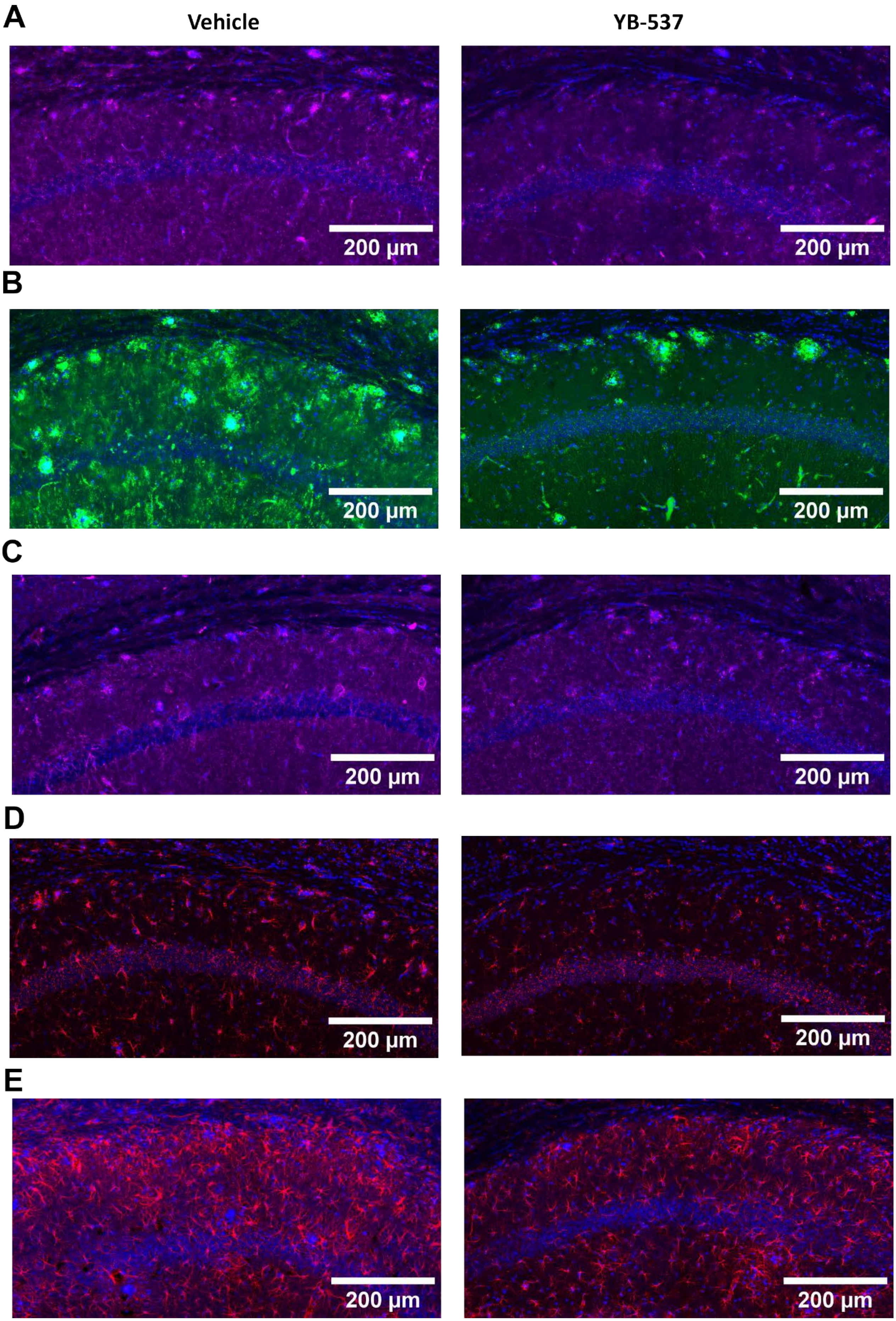
A
Vehicle
YB-537
200 µm
200 µm
B
200 µm
200 µm
C
200 µm
200 µm
D
200 µm
200 µm
E
200 µm
200 µm

Supplementary Figure 9. Representative Images from Female 5xFAD Mice Hippocampus Following 1 Month of YB-537 or Vehicle Ingestion.

(A) 4-HNE (magenta) and DAPI (blue) as imaged from 8–9-month-old female 5xFAD mice hippocampal CA1.

(B) Amyloid β (green) and DAPI (blue) as imaged from 8–9-month-old female 5xFAD mice hippocampal CA1.

(C) Phosphorylated Tau (magenta) and DAPI (blue) as imaged from 8–9-month-old female 5xFAD mice hippocampal CA1.

(D) Iba1 (red) and DAPI (blue) as imaged from 8–9-month-old female 5xFAD mice hippocampal CA1.

(E) GFAP (red) and DAPI (blue) as imaged from 8–9-month-old female 5xFAD mice hippocampal CA1.

Figure 7, right hand microscopy images are shown again in Supplemental figure 9.

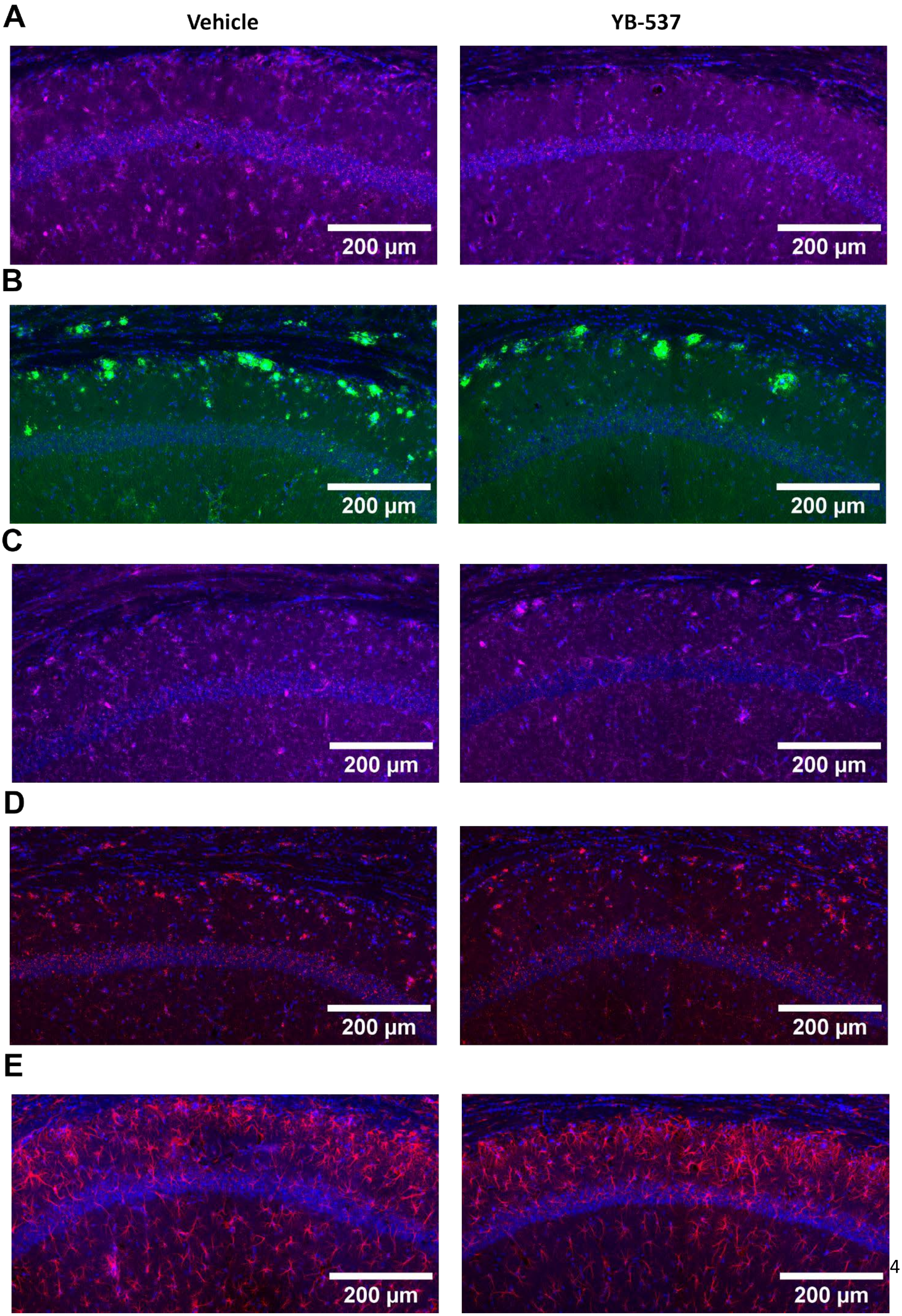

A
Vehicle
YB-537
200 µm
B
C
D
E

Supplementary Figure 10. Representative Images from Male 5xFAD Mice Hippocampus Following 1 Month of YB-537 or Vehicle Ingestion.

(A) 4-HNE (magenta) and DAPI (blue) as imaged from 8-9 month-old male 5xFAD mice hippocampal CA1.

(B) Amyloid β (green) and DAPI (blue) as imaged from 8–9-month-old male 5xFAD mice hippocampal CA1.

(C) Phosphorylated Tau (magenta) and DAPI (blue) as imaged from 8–9-month-old male 5xFAD mice hippocampal CA1.

(D) Iba1 (red) and DAPI (blue) as imaged from 8–9-month-old male 5xFAD mice hippocampal CA1.

(E) GFAP (red) and DAPI (blue) as imaged from 8–9-month-old male 5xFAD mice hippocampal CA1.

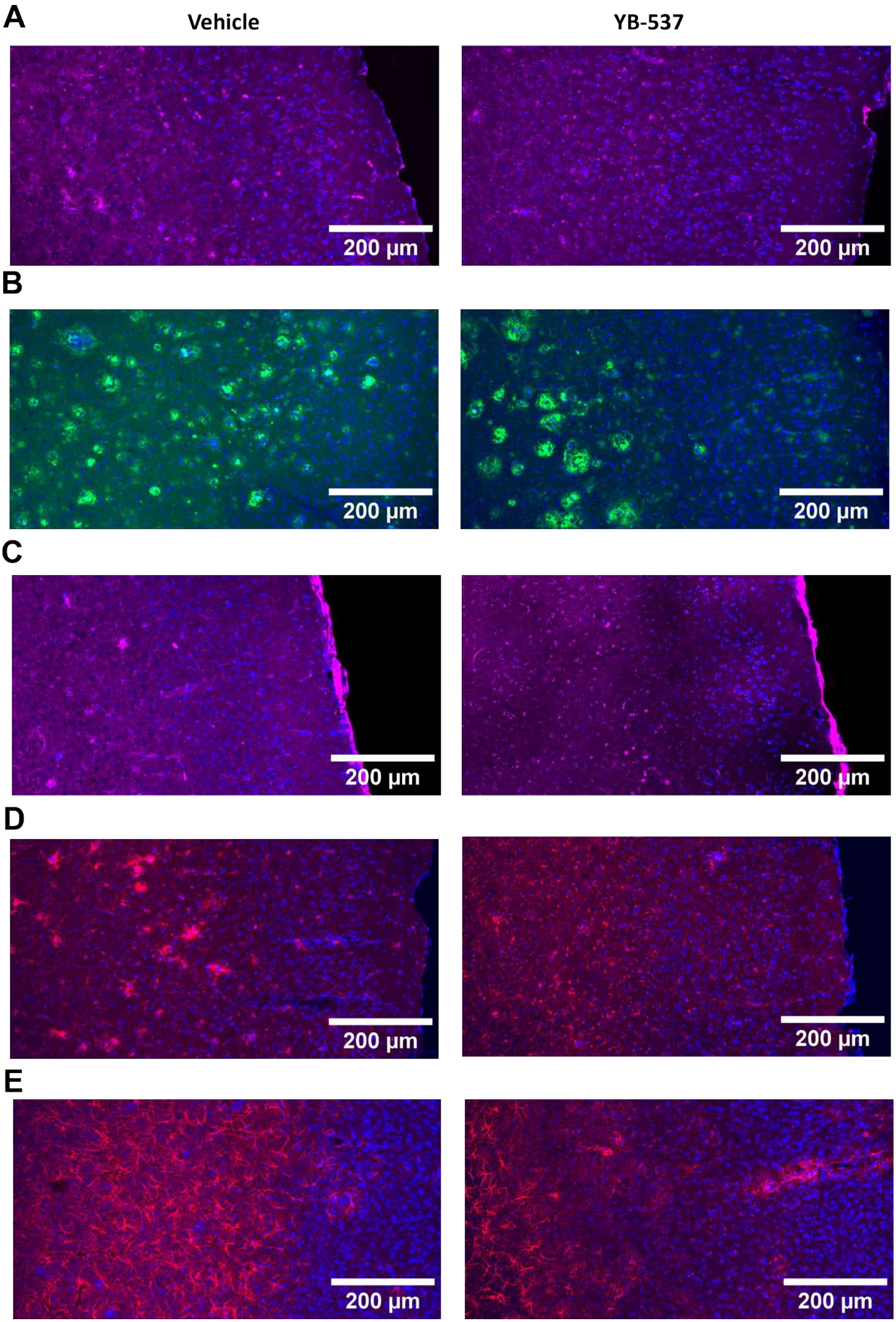
A
Vehicle
YB-537
200 µm
B
C
D
E

Supplementary Figure 11. Representative Images from Female 5xFAD Mice Cortex Following 1 Month of YB-537 or Vehicle Ingestion.

(A) 4-HNE (magenta) and DAPI (blue) as imaged from 8–9-month-old female 5xFAD mice cortex (left/ventral - right/dorsal).

(B) Amyloid β (green) and DAPI (blue) as imaged from 8–9-month-old female 5xFAD mice cortex (left/ventral - right/dorsal).

(C) Phosphorylated Tau (magenta) and DAPI (blue) as imaged from 8–9-month-old female 5xFAD mice cortex (left/ventral - right/dorsal).

(D) Iba1 (red) and DAPI (blue) as imaged from 8–9-month-old female 5xFAD mice cortex (left/ventral - right/dorsal).

(E) GFAP (red) and DAPI (blue) as imaged from 8–9-month-old female 5xFAD mice cortex (left/ventral - right/dorsal).

Supplemental Figure 8, right hand microscopy images are shown again in supplemental figure 11.

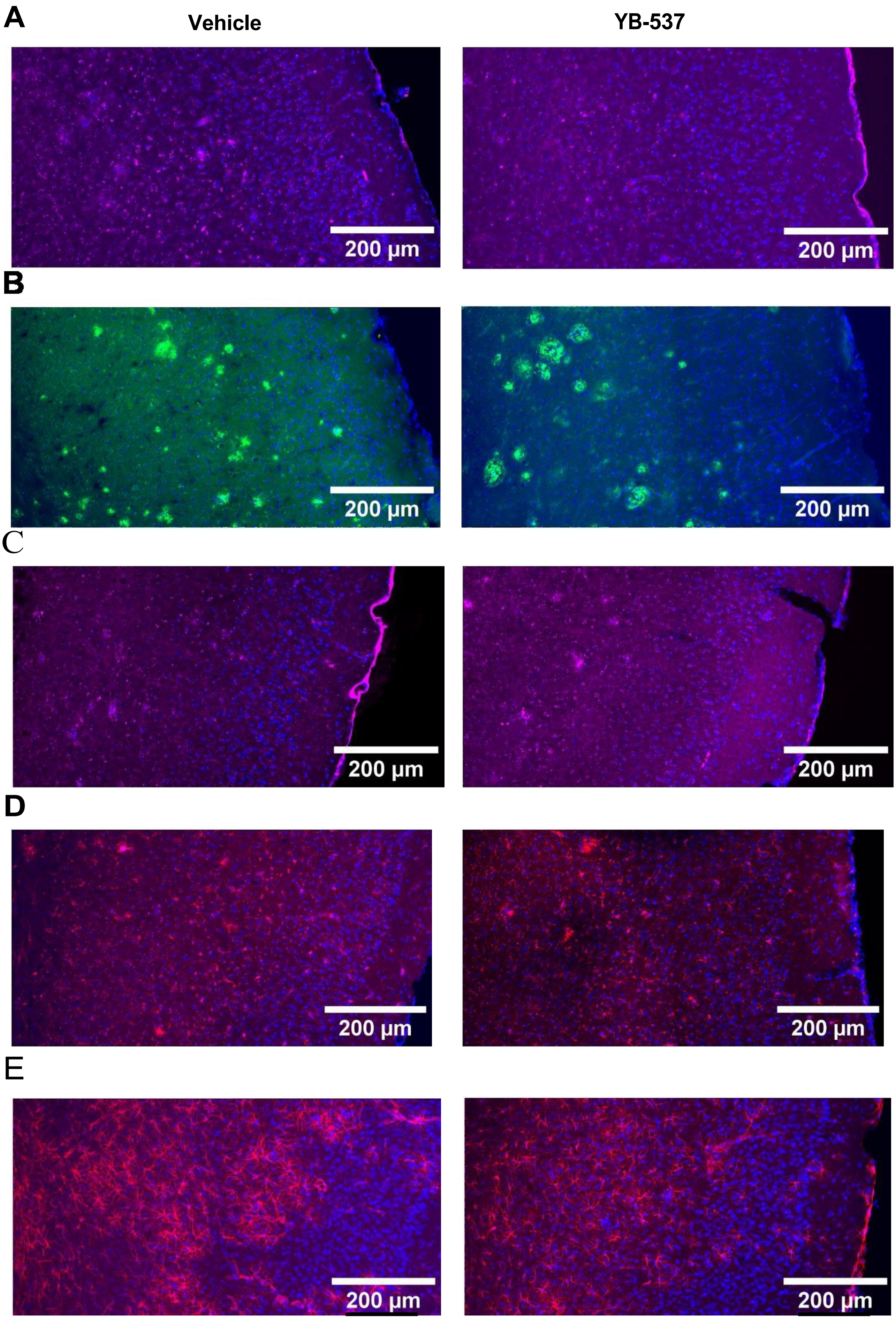

A
Vehicle
YB-537
200 µm
B
C
D
E

Supplementary Figure 12. Representative Images from Male 5xFAD Mice Cortex Following 1 Month of YB-537 or Vehicle Ingestion.

(A) 4-HNE (magenta) and DAPI (blue) as imaged from 8–9-month-old male 5xFAD mice cortex (left/ventral - right/dorsal).

(B) Amyloid β (green) and DAPI (blue) as imaged from 8-9-month-old male 5xFAD mice cortex (left/ventral - right/dorsal).

(C) Phosphorylated Tau (magenta) and DAPI (blue) as imaged from 8–9-month-old male 5xFAD mice cortex (left/ventral - right/dorsal).

(D) Iba1 (red) and DAPI (blue) as imaged from 8–9-month-old male 5xFAD mice cortex (left/ventral - right/dorsal).

(E) GFAP (red) and DAPI (blue) as imaged from 8–9-month-old male 5xFAD mice cortex (left/ventral - right/dorsal).

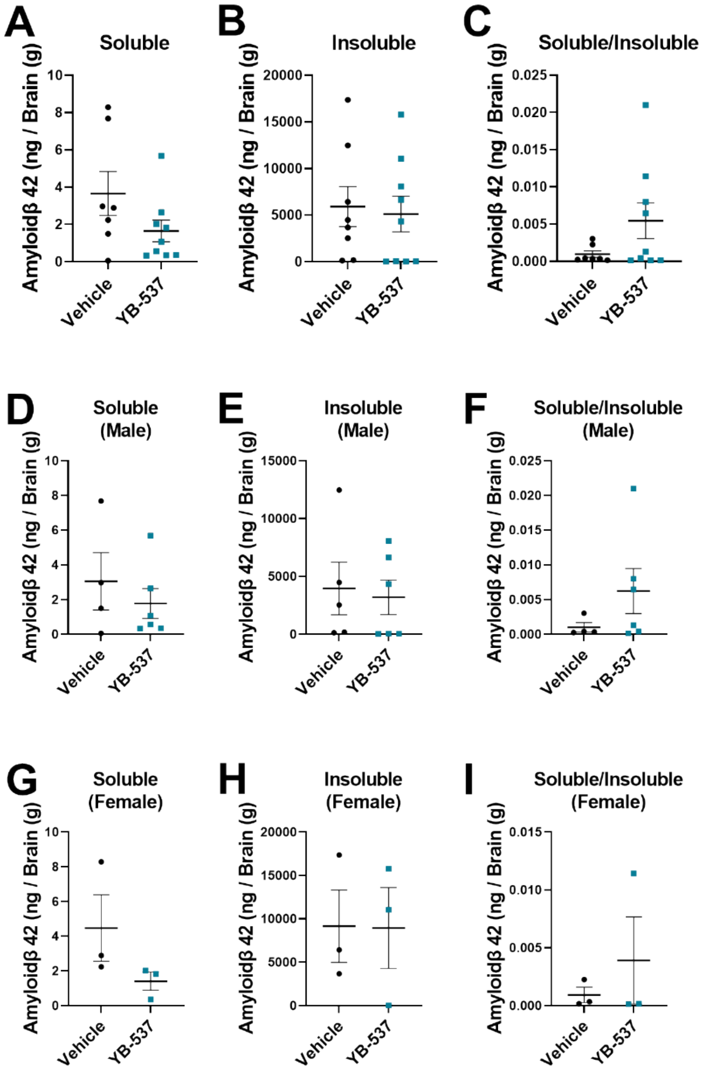

Supplementary Figure 13. Total Amyloid β 42 Levels are Insignificantly Reduced in the Whole Brain of 6-7 Month Old 5xFAD Mice.

(A) Soluble amyloid β 42 is insignificantly reduced in 5xFAD mice following 1 month of YB-537 ingestion, via drinking water (Mann-Whitney test, p=0.1416).

(B) Insoluble amyloid β 42 is insignificantly reduced in 5xFAD mice following 1 month of YB-537 ingestion, via drinking water (unpaired t test, p=0.7855).

(C) Ratio of soluble- to insoluble amyloid β 42 is insignificantly increased in 5xFAD mice following 1 month of YB-537 ingestion, via drinking water (Mann-Whitney test, p=0.5360).

(D) Soluble amyloid β 42 is insignificantly reduced in male 5xFAD mice following 1 month of YB-537 ingestion, via drinking water (Mann-Whitney test, p=0.6095).

(E) Insoluble amyloid β 42 is insignificantly reduced in male 5xFAD mice following 1 month of YB-537 ingestion, via drinking water (Mann-Whitney test, p=0.5368).

(F) Ratio of soluble- to insoluble amyloid β 42 is insignificantly increased in male 5xFAD mice following 1 month of YB-537 ingestion, via drinking water (Mann-Whitney test, p=0.2571).

(G) Soluble amyloid β 42 is insignificantly reduced in female 5xFAD mice following 1 month of YB-537 ingestion, via drinking water (unpaired t test, p=0.1978).

(H) Insoluble amyloid β 42 is insignificantly reduced in female 5xFAD mice following 1 month of YB-537 ingestion, via drinking water (Mann-Whitney test, p>0.9999).

(I) Ratio of soluble- to insoluble amyloid β 42 is insignificantly increased in female 5xFAD mice following 1 month of YB-537 ingestion, via drinking water (Mann-Whitney test, p=0.7000).

Data are shown as mean ±SEM**.**

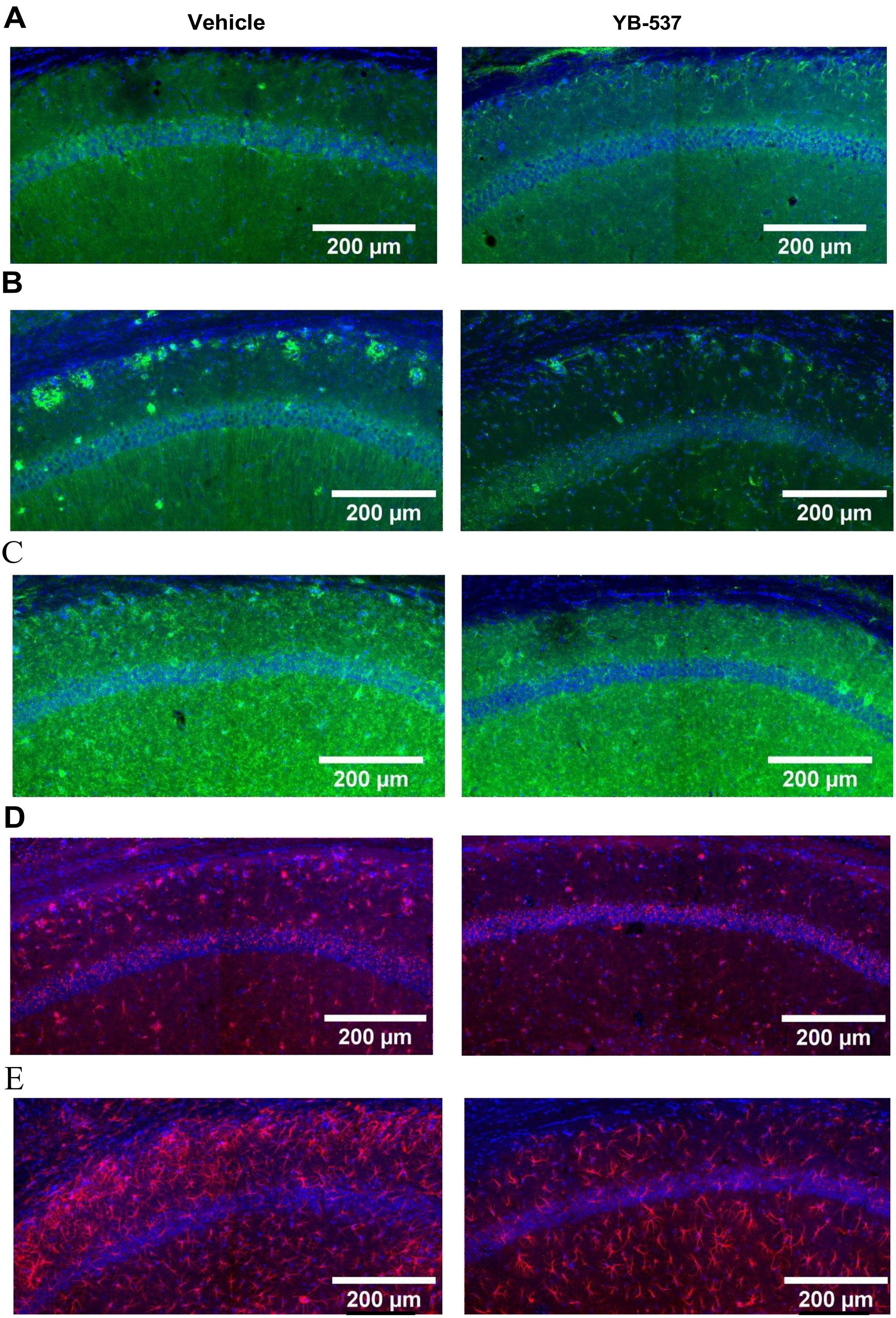
A
Vehicle
YB-537
200 µm
200 µm
B
200 µm
200 µm
C
200 µm
200 µm
D
200 µm
200 µm
E
200 µm
200 µm

Supplementary Figure 14. Representative Images from 9-Month-Old Male 5xFAD Mice Hippocampus Following 4 Month of YB-537 or Vehicle Ingestion.

(E) 4-HNE (magenta) and DAPI (blue) as imaged from 9-month-old male 5xFAD mice hippocampal CA1.

(F) Amyloid β (green) and DAPI (blue) as imaged from 9-month-old male 5xFAD mice hippocampal CA1.

(G) Phosphorylated Tau (magenta) and DAPI (blue) as imaged from 9-month-old male 5xFAD mice hippocampal CA1.

(H) Iba1 (red) and DAPI (blue) as imaged from 9-month-old male 5xFAD mice hippocampal CA1.

(F) GFAP (red) and DAPI (blue) as imaged from 9-month-old male 5xFAD mice hippocampal CA1.

Images in Figure 9 are shown again in Supplemental figure 14.

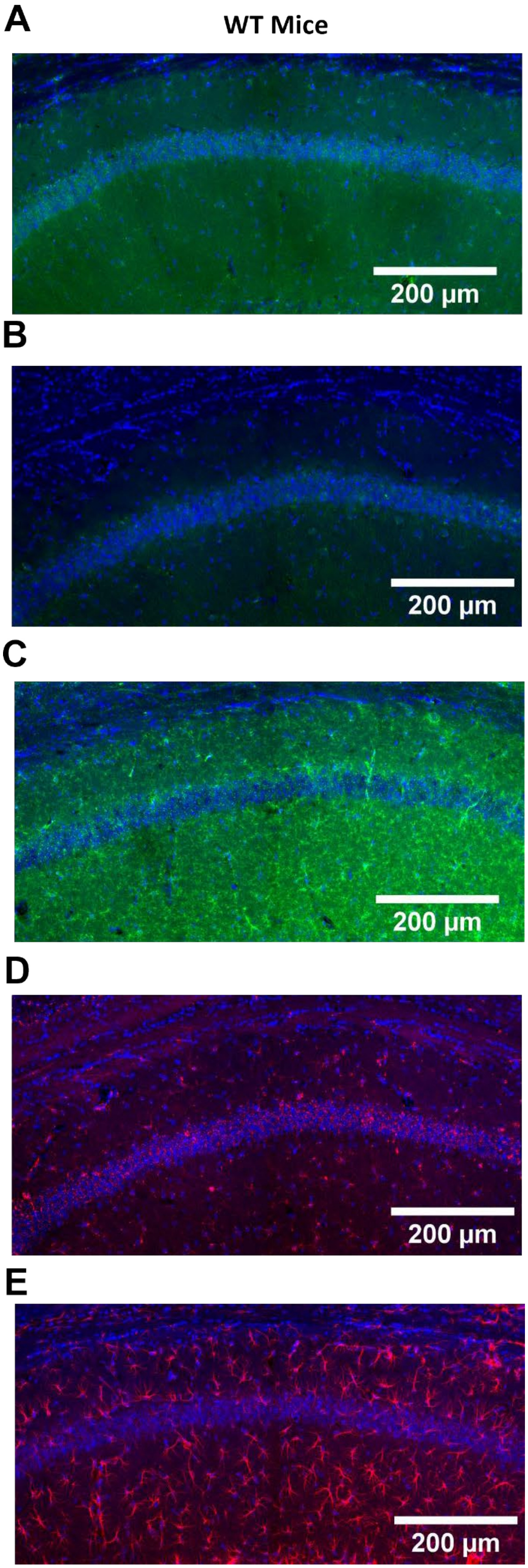
A
WT Mice
200 µm
B
200 µm
C
200 µm
D
200 µm
E
200 µm

Supplementary Figure 15. Representative Images from 9-Month-Old Male WT Mice Hippocampus.

(I) 4-HNE (magenta) and DAPI (blue) as imaged from 9-month-old male WT mice hippocampal CA1.

(J) Amyloid β (green) and DAPI (blue) as imaged from 9-month-old male WT mice hippocampal CA1.

(K) Phosphorylated Tau (magenta) and DAPI (blue) as imaged from 9-month-old male WT mice hippocampal CA1.

(L) Iba1 (red) and DAPI (blue) as imaged from 9-month-old male WT mice hippocampal CA1.

(G) GFAP (red) and DAPI (blue) as imaged from 9-month-old male WT mice hippocampal CA1.

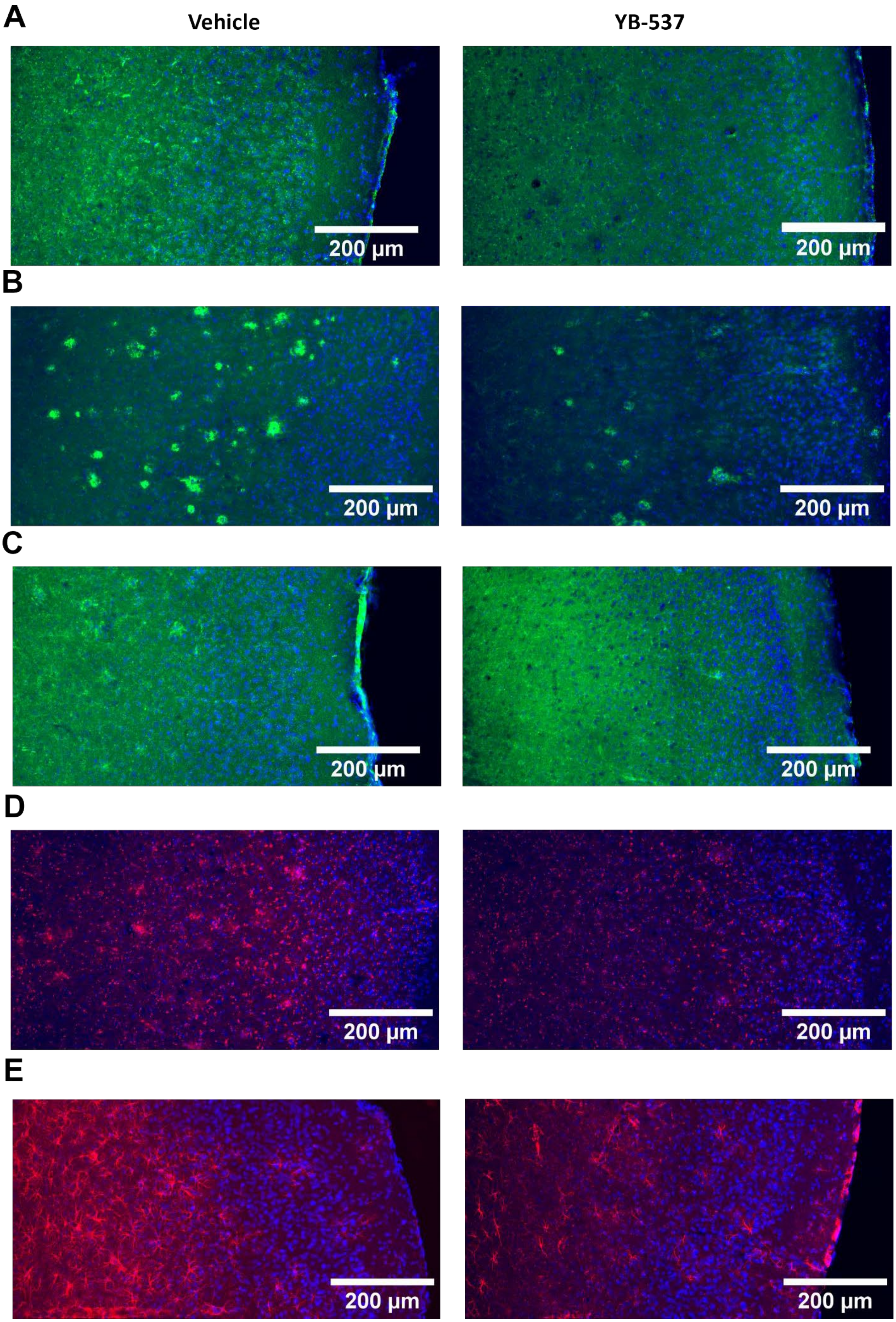
A
Vehicle
YB-537
200 µm
200 µm
B
200 µm
200 µm
C
200 µm
200 µm
D
200 µm
200 µm
E
200 µm
200 µm

Supplementary Figure 16. Representative Images from 9-Month-Old Male 5xFAD Mice Cortex Following 4 Month of YB-537 or Vehicle Ingestion.

(M) 4-HNE (magenta) and DAPI (blue) as imaged from 9-month-old male 5xFAD mice cortex.

(N) Amyloid β (green) and DAPI (blue) as imaged from 9-month-old male 5xFAD mice cortex.

(O) Phosphorylated Tau (magenta) and DAPI (blue) as imaged from 9-month-old male 5xFAD mice cortex.

(P) Iba1 (red) and DAPI (blue) as imaged from 9-month-old male 5xFAD mice cortex.

(H) GFAP (red) and DAPI (blue) as imaged from 9-month-old male 5xFAD mice cortex.

Images in Figure 9 are shown again in Supplemental figure 16.

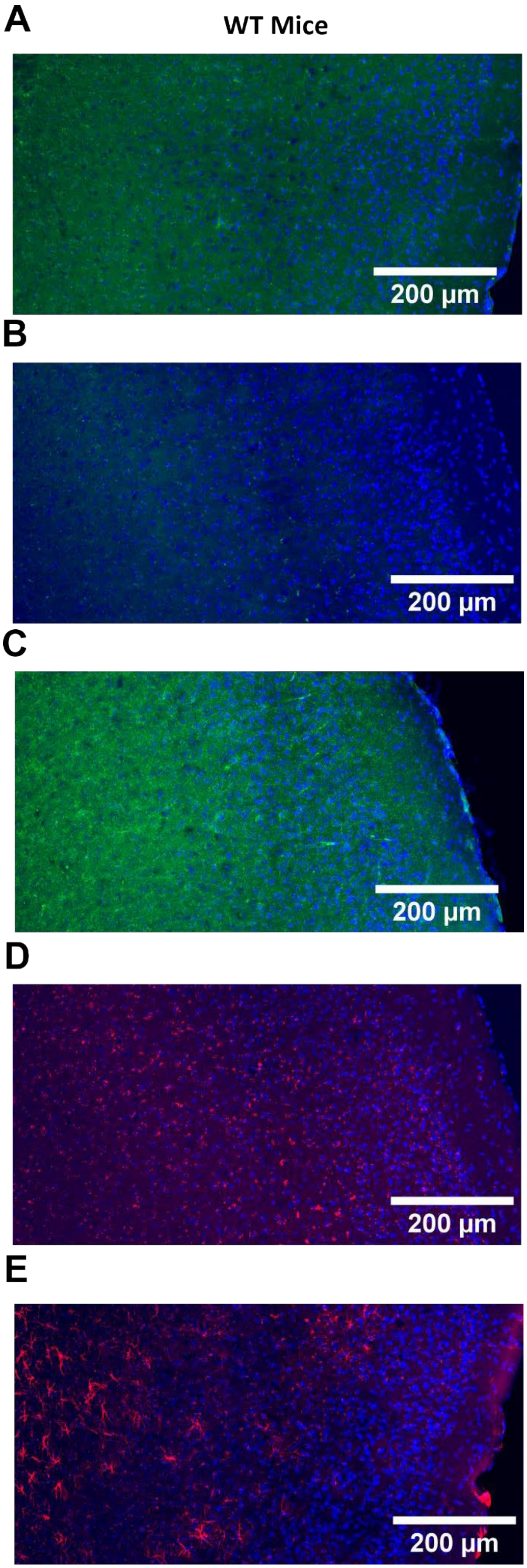
A
WT Mice
200 µm
B
200 µm
C
200 µm
D
200 µm
E
200 µm

Supplementary Figure 17. Representative Images from 9-Month-Old Male WT Mice Cortex.

(Q) 4-HNE (magenta) and DAPI (blue) as imaged from 9-month-old male WT mice cortex.

(R) Amyloid β (green) and DAPI (blue) as imaged from 9-month-old male WT mice cortex.

(S) Phosphorylated Tau (magenta) and DAPI (blue) as imaged from 9-month-old male WT mice cortex.

(T) Iba1 (red) and DAPI (blue) as imaged from 9-month-old male WT mice cortex.

(I) GFAP (red) and DAPI (blue) as imaged from 9-month-old male WT mice cortex.

Supplementary Table 1. Complete List of Sulfonamide HTS Hits and Newly Synthesized QR2 Inhibitors.

Compound	Structure	QR2 IC50 (µM)		QR1 IC50 (µM)		THLE2 IC50 (µM)
		Absorbance	Fluorescence	Absorbance	Fluorescence	
PCM-0075065*/&	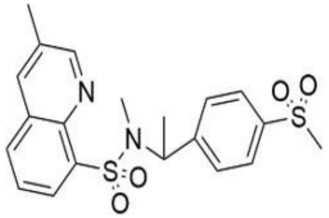	**0.87**	**1.22**	**>9.99**	**>9.90**	31.7
PCM-0104765*/&	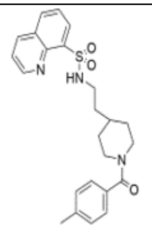	**>9.99**	**>9.90**	**>9.99**	**>9.90**	**---**
PCM-0105761*/&	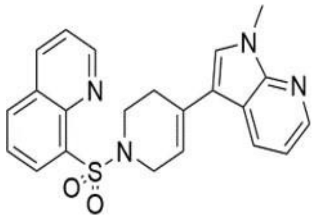	**7.15**	**>9.90**	**5.98**	**7.98**	**---**
PCM-0105933*/&	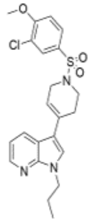	**>9.99**	**>9.90**	**>9.99**	**>9.90**	**---**

PCM-0119249*/&	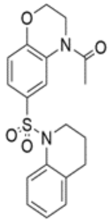	**>9.99**	**>9.90**	**3.48**	**4.71**	**---**
PCM-0119438*/&	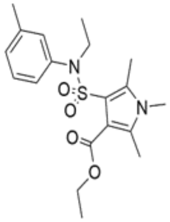	**8.98**	**3.59**	**>9.99**	**5.39**	**---**
PCM-0119478*/&	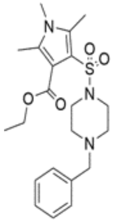	**>9.99**	**>9.90**	**>9.99**	**>9.90**	**---**
PCM-0119715*/&	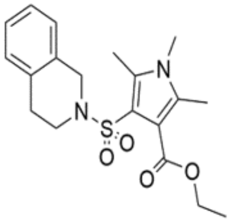	**3.87**	**3.19**	**>9.99**	**9.40**	**---**
PCM-0119717*/&	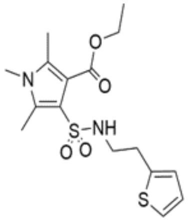	**5.20**	**9.86**	**7.95**	**>9.90**	**---**

PCM-0120203*/&	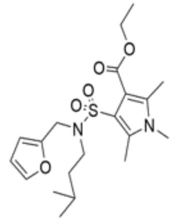	8.53	8.21	>9.90	3.79	---
PCM-0120247*/&	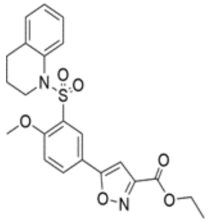	>9.99	>9.90	>9.99	>9.90	---
PCM-0121306*/&	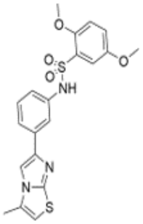	2.03	0.83	>9.99	8.77	---
PCM-0121432*/&	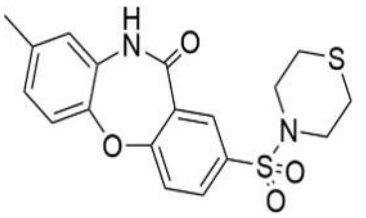	1.22	0.99	1.67	0.83	---
PCM-0124575*/&	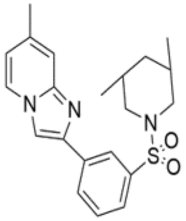	1.22	2.18	>9.99	>9.90	---

PCM-0124615*/&	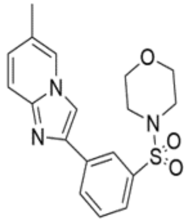	**1.65**	**3.25**	**>9.99**	**>9.90**	**---**
PCM-0124655*/&	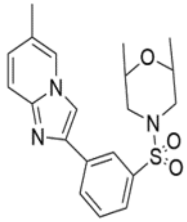	**3.03**	**4.42**	**>9.99**	**>9.90**	**---**
PCM-0124772*/&	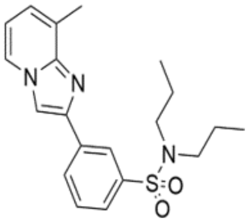	**0.75**	**0.83**	**>9.99**	**>9.90**	**---**
PCM-0125078*/&	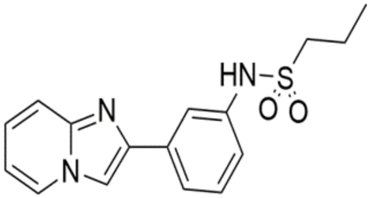	**1.05**	**1.29**	**4.51**	**7.70**	**---**
PCM-0127546*/&	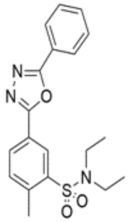	**3.12**	**4.82**	**>9.99**	**>9.90**	**---**

PCM-0128885*/&	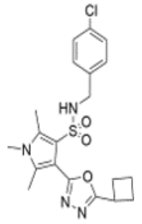	>9.99	>9.90	>9.99	>9.90	---
PCM-0129836*/&	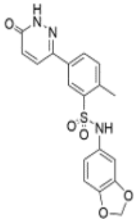	>9.99	>9.90	>9.99	>9.90	---
PCM-0130075*/&	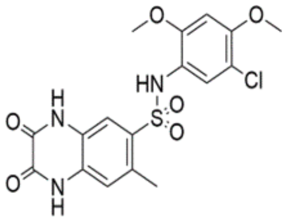	>9.99	>9.90	>9.99	>9.90	---
PCM-0130766*/&	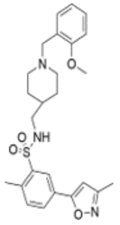	4.05	6.69	>9.99	>9.90	---
PCM-0131156*/&	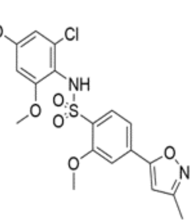	2.73	3.82	>9.99	>9.90	---

PCM-0136246[*/&]	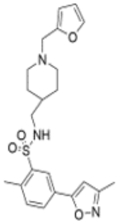	2.56	3.98	>9.99	>9.90	---
PCM-0136286[*/&]	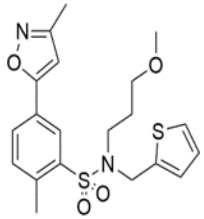	9.75	>9.90	>9.99	>9.90	---
PCM-0138586[*/&]	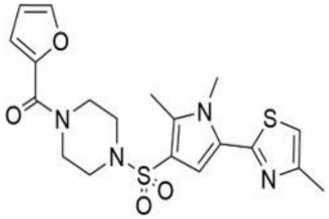	4.72	5.37	>9.99	>9.90	---
PCM-0150171[*/&]	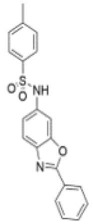	>9.99	2.21	4.58	1.55	---
PCM-0154188[*/&]	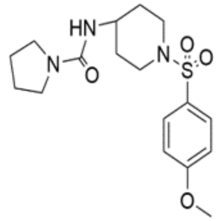	3.83	2.06	>9.99	>9.90	---

PCM-0175935[*/&]	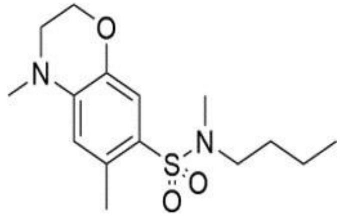	5.22	7.82	>9.99	>9.90	---
PCM-0181407[*/&]	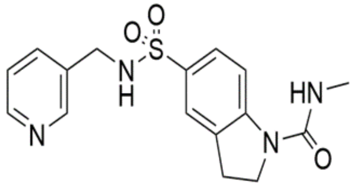	>9.99	>9.90	>9.99	>9.90	---
PCM-0184891[*/&]	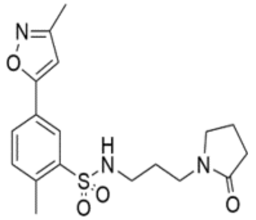	2.37	3.26	>9.99	>9.90	218.6
PCM-0184959[*/&]	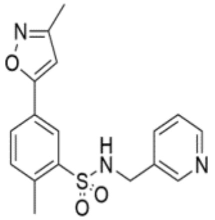	2.16	2.72	>9.99	>9.90	249.4
PCM-0184994[*/&]	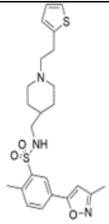	5.88	8.75	>9.99	>9.90	---

PCM-0184999[*/&]	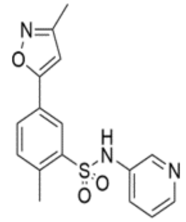	**>9.99**	**>9.90**	**>9.99**	**>9.90**	**---**
PCM-0185005[*/&]	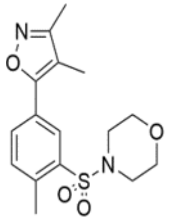	**>9.99**	**>9.90**	**>9.99**	**>9.90**	**---**
PCM-0185051[*/&]	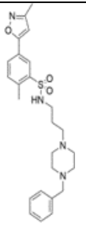	**3.19**	**6.20**	**>9.99**	**>9.90**	**---**
PCM-0190999[*/&]	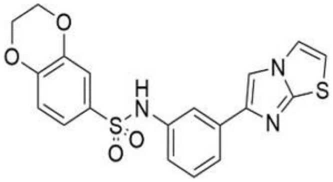	**4.03**	**5.62**	**<0.750**	**<0.83**	**---**
PCM-0195566[*/&]	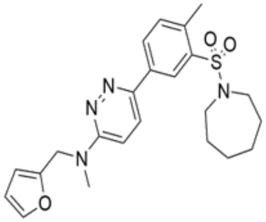	**7.27**	**6.26**	**>9.99**	**>9.90**	**---**

PCM-0195617[*/&]	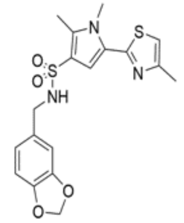	3.58	4.02	>9.99	>9.90	---
PCM-0197024[*/&]	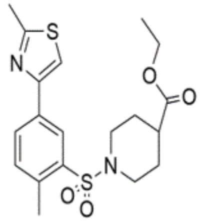	3.20	4.16	>9.99	>9.90	---
PCM-0200337[*/&]	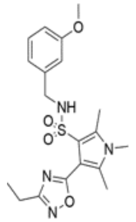	8.09	>9.90	>9.99	>9.90	---
PCM-0009158[*]	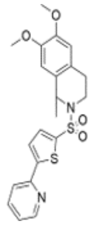	---	4.17	---	9.80	---
PCM-0009988[*]	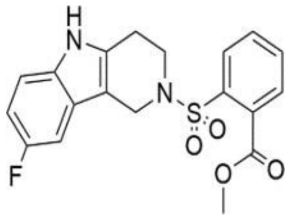	---	6.56	---	9.80	---

PCM-0011948*	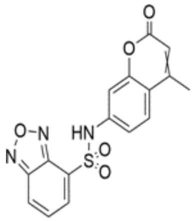	---	**6.69**	---	**5.46**	---
PCM-0023277*	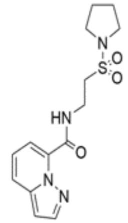	---	**5.03**	---	**4.92**	---
PCM-0023869*	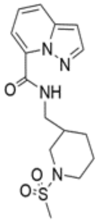	---	**5.15**	---	**5.10**	---
PCM-0025239*	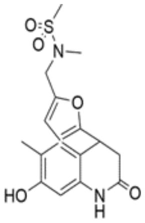	---	**8.51**	---	**8.16**	---
PCM-0033303*	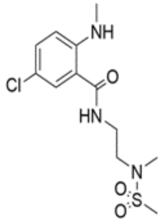	---	**5.02**	---	**4.97**	---

PCM-0069025*	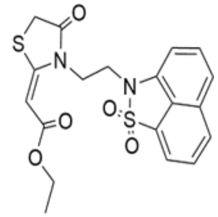	---	3.71	--	5.29	---
PCM-0071569*	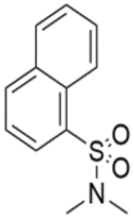	---	>9.80	---	>9.80	---
PCM-0072788*	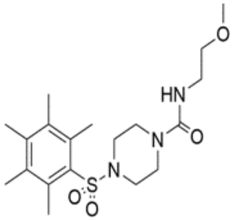	---	>9.80	---	>9.80	---
PCM-0077674*	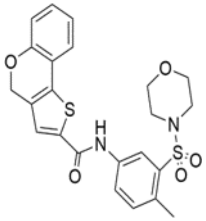	---	3.07	---	2.99	---
PCM-0081896*	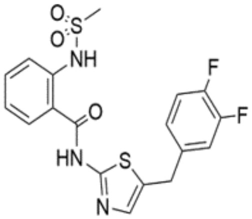	---	4.83	---	4.60	---

PCM-0083647*	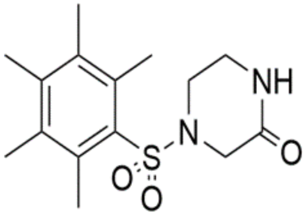	---	7.04	---	>9.80	---
PCM-0086066*	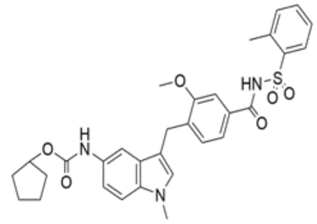	---	5.14	---	>9.80	---
PCM-0090313*	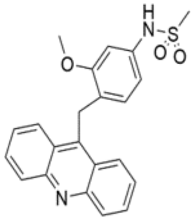	---	>9.80	---	>9.80	---
PCM-0105406*	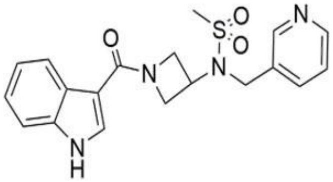	8.43	>9.90	>9.99	>9.90	---
PCM-0105813*	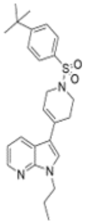	>9.99	>9.90	>9.99	>9.90	---

PCM-0105851*	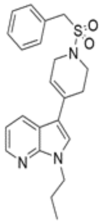	**9.05**	**>9.90**	**>9.99**	**>9.90**	**---**
PCM-0109036*	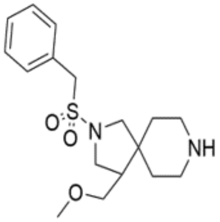	**>9.99**	**>9.90**	**>9.99**	**>9.90**	**---**
PCM-0120253*	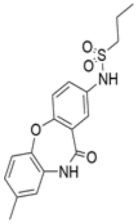	**8.89**	**>9.90**	**>9.99**	**4.60**	**---**
PCM-0120563*	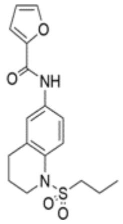	**>9.99**	**>9.90**	**>9.99**	**>9.90**	**---**
PCM-0121398*	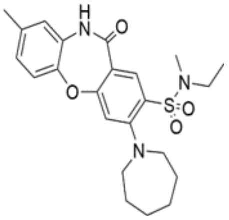	**4.22**	**>9.90**	**3.85**	**>9.90**	**---**

PCM-0121512*	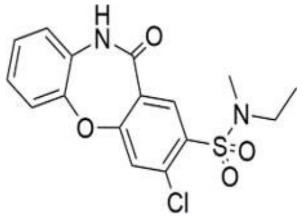	**2.06**	**5.14**	**9.49**	**>9.90**	**---**
PCM-0121635*	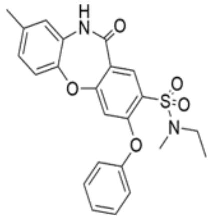	**1.23**	**0.83**	**5.54**	**2.53**	**---**
PCM-0124735*	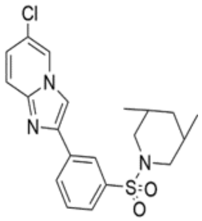	**>9.99**	**>9.90**	**>9.99**	**>9.90**	**---**
PCM-0129681*	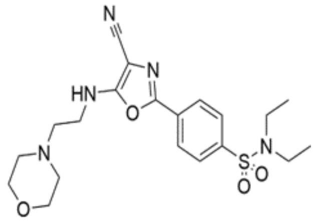	**>9.99**	**1.98**	**>9.99**	**2.04**	**---**
PCM-0140113*	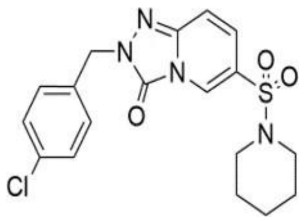	**>9.99**	**>9.90**	**>9.99**	**>9.90**	**---**

PCM-0172615*	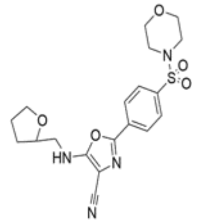	**>9.99**	**<0.83**	**>9.99**	**<0.83**	**---**
PCM-0172736*	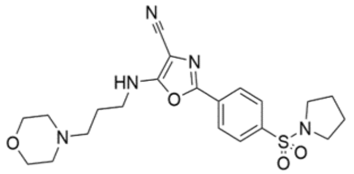	**>9.99**	**<0.83**	**>9.99**	**<0.83**	**---**
PCM-0172816*	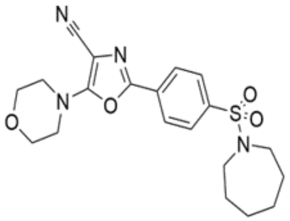	**>9.99**	**<0.83**	**>9.99**	**<0.83**	**---**
PCM-0181527*	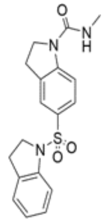	**>9.99**	**>9.90**	**>9.99**	**>9.90**	**---**
PCM-0184971*	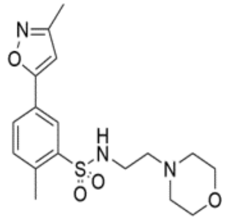	**>9.99**	**>9.90**	**>9.99**	**>9.90**	**---**

PCM-0193675*	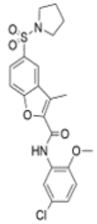	>9.99	>9.90	>9.99	>9.90	---
PCM-0195577*	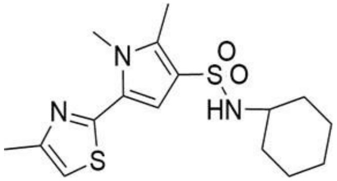	>9.99	>9.90	>9.99	>9.90	---
PCM-0198001*	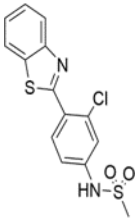	1.33	1.79	7.56	1.66	---
PCM-0199036*	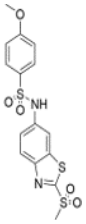	1.23	4.30	1.41	0.92	---
PCM-0206067*	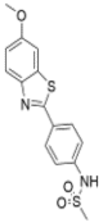	>9.99	<0.83	>9.99	<0.83	---

PCM-0207818*	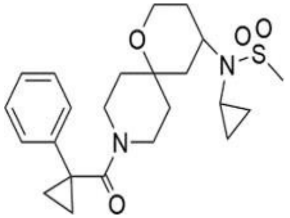	>9.99	>9.90	>9.99	>9.90	---
PCM-0124535*/#/&	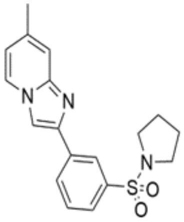	0.16	0.59	>9.99	>9.99	88.9
PCM-0124812*/#/&	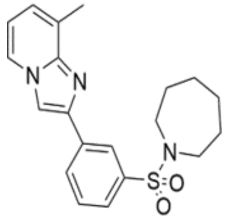	0.01	0.004	7.24	5.83	45.4
PCM-0127506*/#/&	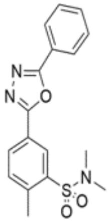	1.620	2.202	>9.99	>9.99	---
PCM-0127586**/#/&	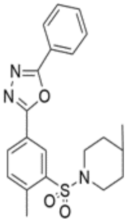	1.20	5.97	>9.99	>9.99	---

PCM-0186036[*#/&]	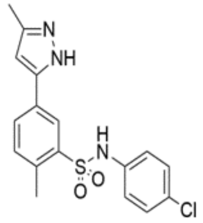	**1.16**	**2.36**	**>9.99**	**>9.99**	**---**
PCM-0197066[*#/&]	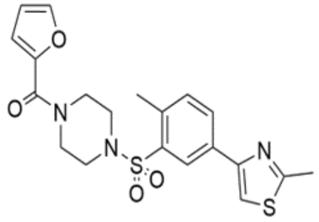	**1.28**	**4.65**	**>9.99**	**>9.99**	**---**
PCM-0197104[*#/&]	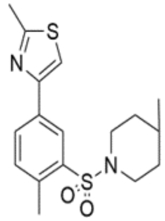	**4.58**	**7.40**	**>9.99**	**>9.99**	**---**
PCM-0212354[**#/&]	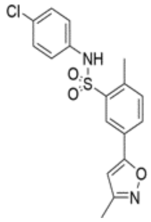	**>9.99**	**7.01**	**>9.99**	**>9.99**	**8.5**
PCM-0212385[**#/&]	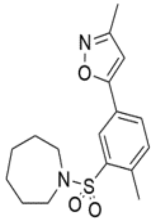	**1.24**	**1.92**	**>9.99**	**>9.99**	**23.8**

PCM-0212386**/#/&	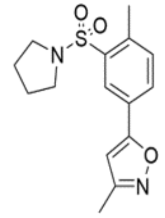	**0.75**	**0.42**	**>9.99**	**>9.99**	**65.1**
PCM-0212387**/#/&	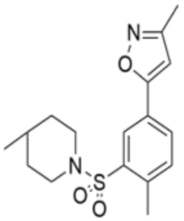	**1.83**	**2.89**	**>9.99**	**>9.99**	**50.0**
PCM-0212388**/#/&	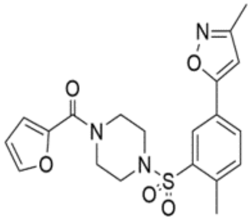	**1.25**	**1.19**	**>9.99**	**>9.99**	**123.5**
PCM-0212389**/#/&	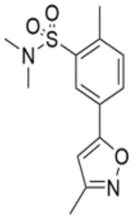	**1.71**	**2.31**	**>9.99**	**>9.99**	**215.1**
PCM-0213786**/#/&	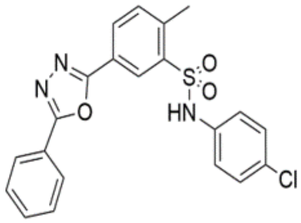	**>9.99**	**>9.99**	**>9.99**	**>9.99**	**---**

PCM-0213787**/#/&	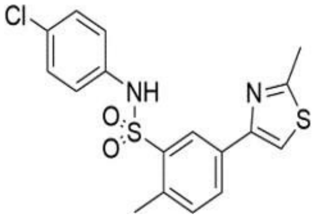	>9.99	>9.99	>9.99	>9.99	---
PCM-0213788**/#/&	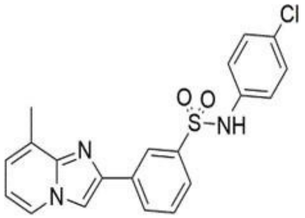	0.05	3.04	>9.99	>9.99	---
PCM-0213789**/#/&	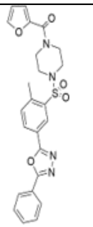	>9.99	5.71	>9.99	>9.99	---
PCM-0213790**/#/&	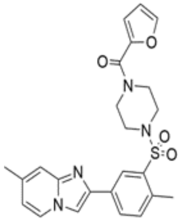	0.11	0.27	7.15	>9.99	---
PCM-0213791**/#/&	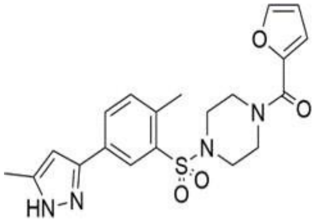	0.08	0.26	>9.99	>9.99	---

PCM-0213792**/#/&	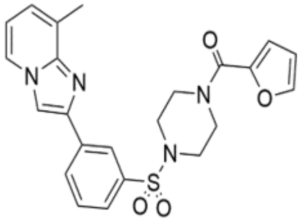	0.01	0.01	4.22	3.33	64.1
PCM-0213793**/#/&	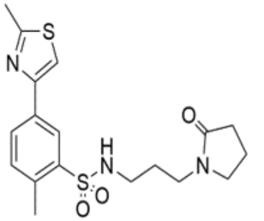	2.91	5.45	>9.99	>9.99	---
PCM-0213794**/#/&	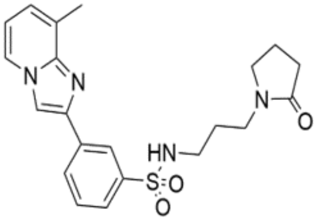	0.11	0.25	>9.99	>9.99	---
PCM-0213795**/#/&	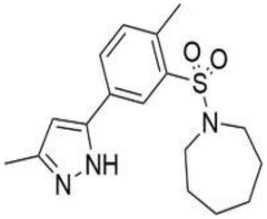	0.28	0.64	>9.99	>9.99	---
PCM-0213796**/#/&	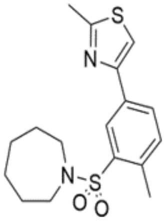	2.44	>9.99	>9.99	>9.99	---

PCM-0213797**/#/&	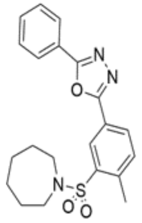	**1.06**	**3.38**	**>9.99**	**>9.99**	**---**
PCM-0213798**/#/&	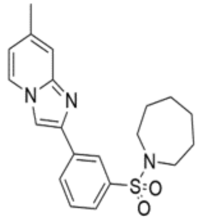	**0.41**	**1.27**	**>9.99**	**>9.99**	**---**
PCM-0213799**/#/&	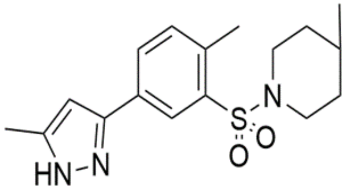	**0.27**	**0.90**	**>9.99**	**>9.99**	**---**
PCM-0213800 / YB-800**/#/&/$	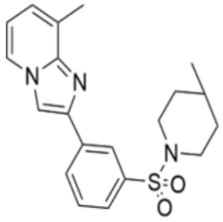	**0.02**	**0.03**	**>9.99**	**>9.99**	**78.4**
PCM-0213801**/#/&	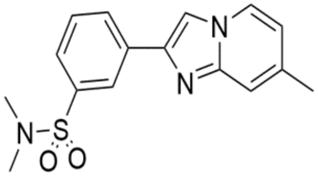	**0.35**	**0.70**	**>9.99**	**>9.99**	**---**

PCM-0213802**/#/&	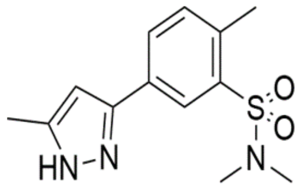	**0.16**	**0.39**	**>9.99**	**>9.99**	**---**
PCM-0213803**/#/&	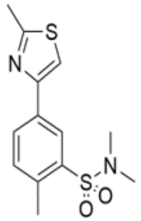	**0.10**	**2.80**	**>9.99**	**>9.99**	**---**
PCM-0213804**/#/&/$	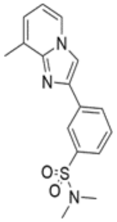	**0.02**	**0.19**	**>9.99**	**>9.99**	**>100.0**
PCM-0213805**/#/&	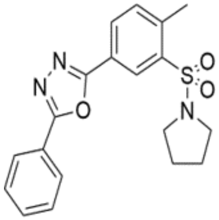	**0.25**	**1.48**	**>9.99**	**>9.99**	**---**

PCM-0213806[**/#/&/$]	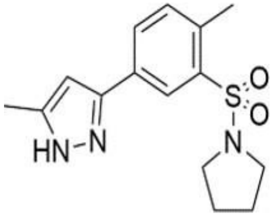	**0.05**	**0.17**	**>9.99**	**>9.99**	**---**
PCM-0213807[**/#/&]	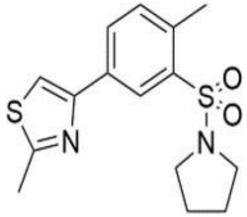	**0.37**	**1.55**	**>9.99**	**>9.99**	**---**
PCM-0213808 / YB-808[**/#/&/$]	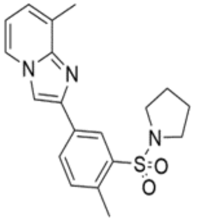	**0.01**	**0.01**	**>9.99**	**>9.99**	**59.7**
PCM-0213809[**/#/&]	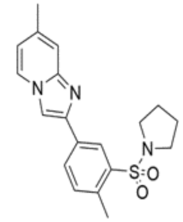	**0.18**	**0.51**	**>9.99**	**>9.99**	**---**
PCM-0213811[**/#/&]	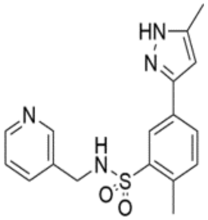	**0.98**	**3.16**	**>9.99**	**>9.99**	**---**

PCM-0213812**/#/&	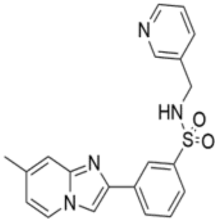	0.75	9.62	8.60	>9.99	---
PCM-0213813**/#/&	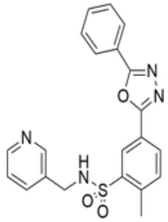	1.17	6.49	>9.99	>9.99	---
PCM-0213814**/#/&/$	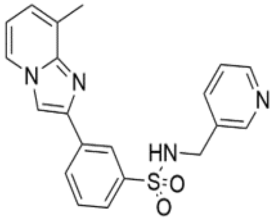	0.02	0.02	7.18	>9.99	>99.0
PCM-0213815**/#/&	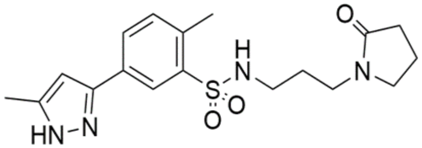	0.28	0.93	>9.99	>9.99	---

PCM-0213816**/#/&	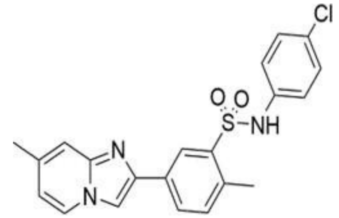	>9.99	>9.99	>9.99	>9.99	---
PCM-0213817**/#/&	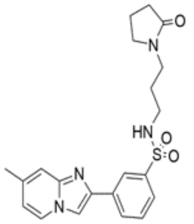	0.32	0.70	3.44	5.28	---
PCM-0213818**/#/&	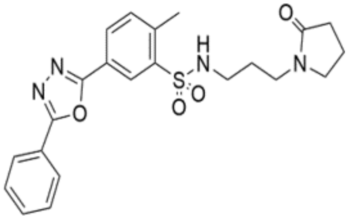	9.80	>9.99	>9.99	>9.99	---
PCM-0214338**/#/&/$	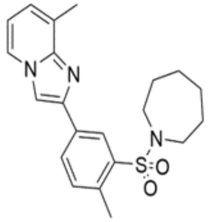	0.03	0.02	>9.99	>9.99	>99.0
PCM-0214540 / YB-540**/#/&/$$	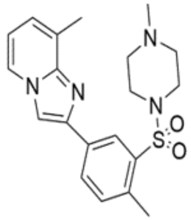	---	0.425	---	>9.99	---

PCM-0220537 / YB-537**/#/&/$$	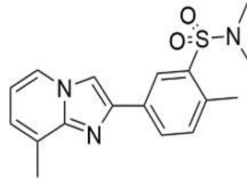	**---**	**0.003**	**---**	**18.4**	**>99.0**
PCM-0214339**/#/&	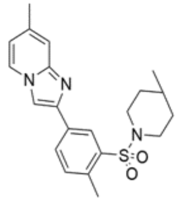	**4.49**	**3.96**	**---**	**---**	**59.4**

** or ** : HTS hit or Synthesis; # with NMR; & with LC-MS; $ or $$: not soluble in water or soluble in water*

Supplementary Table 2. Characterization of Selected Compounds.

IB-1-37 (8-methyl-2-(4-methyl-3-((4-methylpiperazin-1-yl)sulfonyl)phenyl)imidazo[1,2-a]pyridine)-

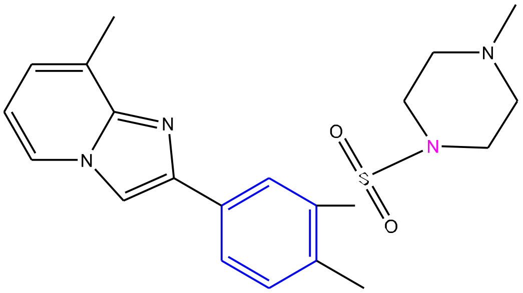

1 H NMR (400 MHz, MeOD) δ ppm 8.45 (s, 1H), 8.25 (d, J=6.48Hz, 1H), 8.21 (s, 1H), 8.05 (d, J=7.84Hz, 1H), 7.47 (d, J=7.96Hz, 1H), 7.09 (d, J=6.72Hz, 1H), 6.81 (dd, J=6.8Hz, 1H), 3.25 (t, J=4.48 Hz, 4H), 2.64 (s, 3H), 2.60 (s, 3H), 2.49 (t, J= 4.72Hz, 4H), 2.28 (s, 3H).

LRMS (ESI+): m/z =385.2.

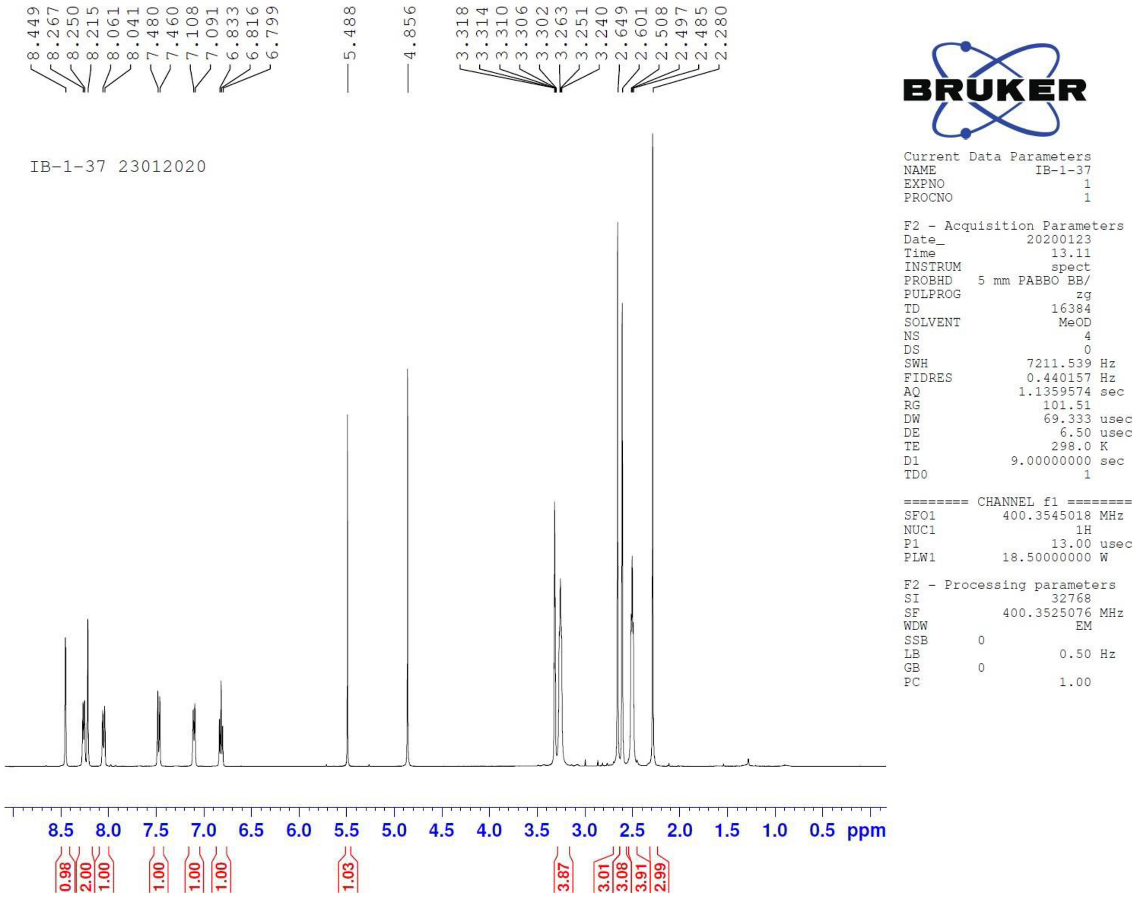

IB-1-42 (8-methyl-2-(4-methyl-3-(pyrrolidin-1-ylsulfonyl)phenyl)imidazo[1,2-a]pyridine)-

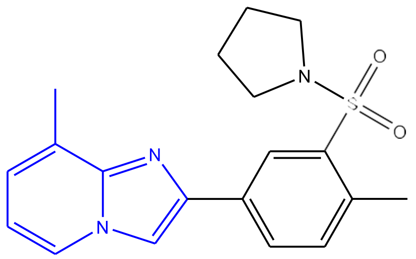

1 H NMR (400 MHz, MeOD) δ ppm 8.47 (s, 1H), 8.30 (d, J=6.76Hz, 1H), 8.24 (s, 1H), 8.06 (dd, J= 8, 1.44Hz, 1H), 7.49 (d, J=7.96Hz, 1H), 7.14 (d, J=6.88, 1H), 6.85 (dd, J=6.84Hz, 1H), 3.34-3.40 (m, 4H), 2.67 (s, 3H), 2.62 (s, 3H), 1.95 (quint, J=3.6Hz, 4H)

LRMS (ESI+): m/z =356.6.

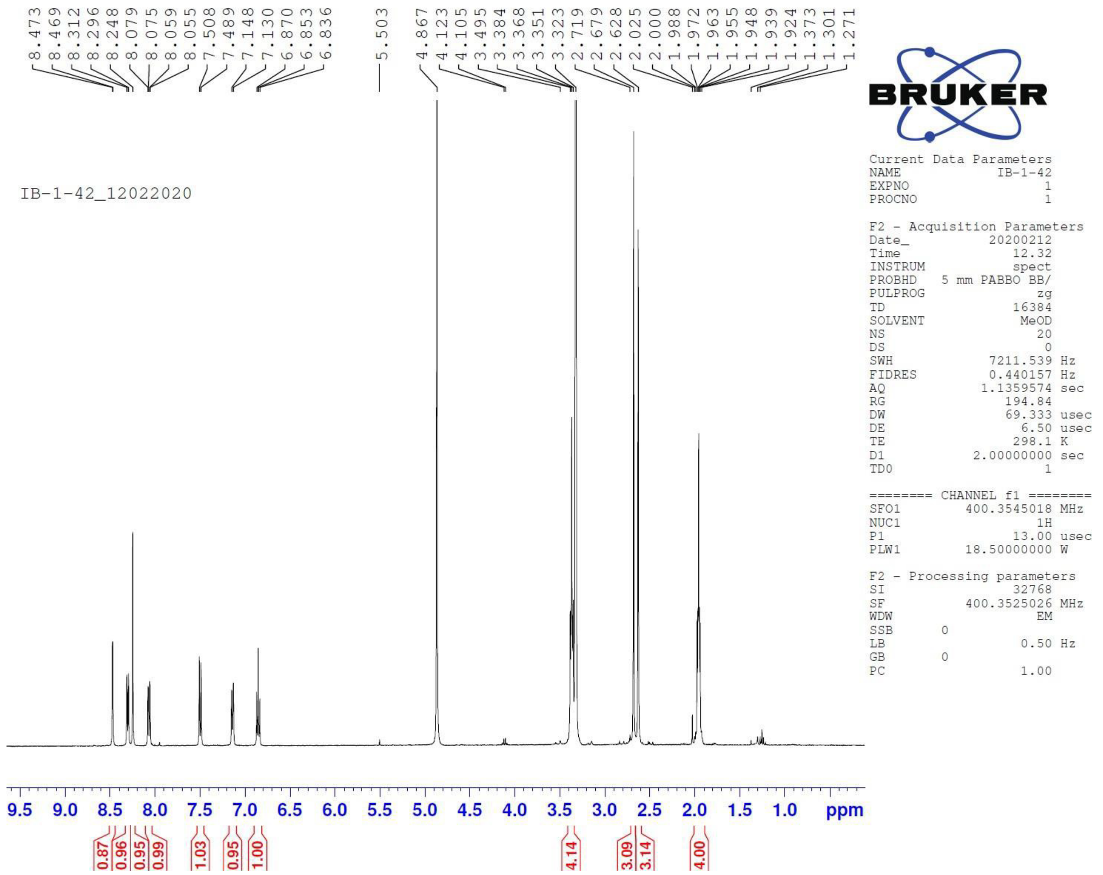

IB-1-16 (2-methyl-5-(7-methylimidazo[1,2-a]pyridin-2-yl)-N-(pyridin-3-ylmethyl)benzenesulfonamide)-

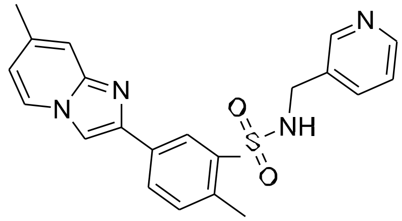

1 H NMR (400 MHz, MeOD) δ ppm 8.38 (d, J=1.6Hz, 2H), 8.27-8.34 (m, 2H), 8.14 (s, 1H), 7.95 (dd, J= 8.32, 1.6Hz, 1H), 7.70 (d, J= 7.88 Hz, 1H), 7.39 (d, J= 7.92 Hz, 1H), 7.34 (s, 1H), 7.22-7.29 (m, 1H), 6.81 (dd, J= 6.72, 1.6Hz, 1H), 4.23 (s, 2H), 2.63 (s, 3H), 2.43 (s, 3H).

LRMS (ESI+): m/z = 393.3.

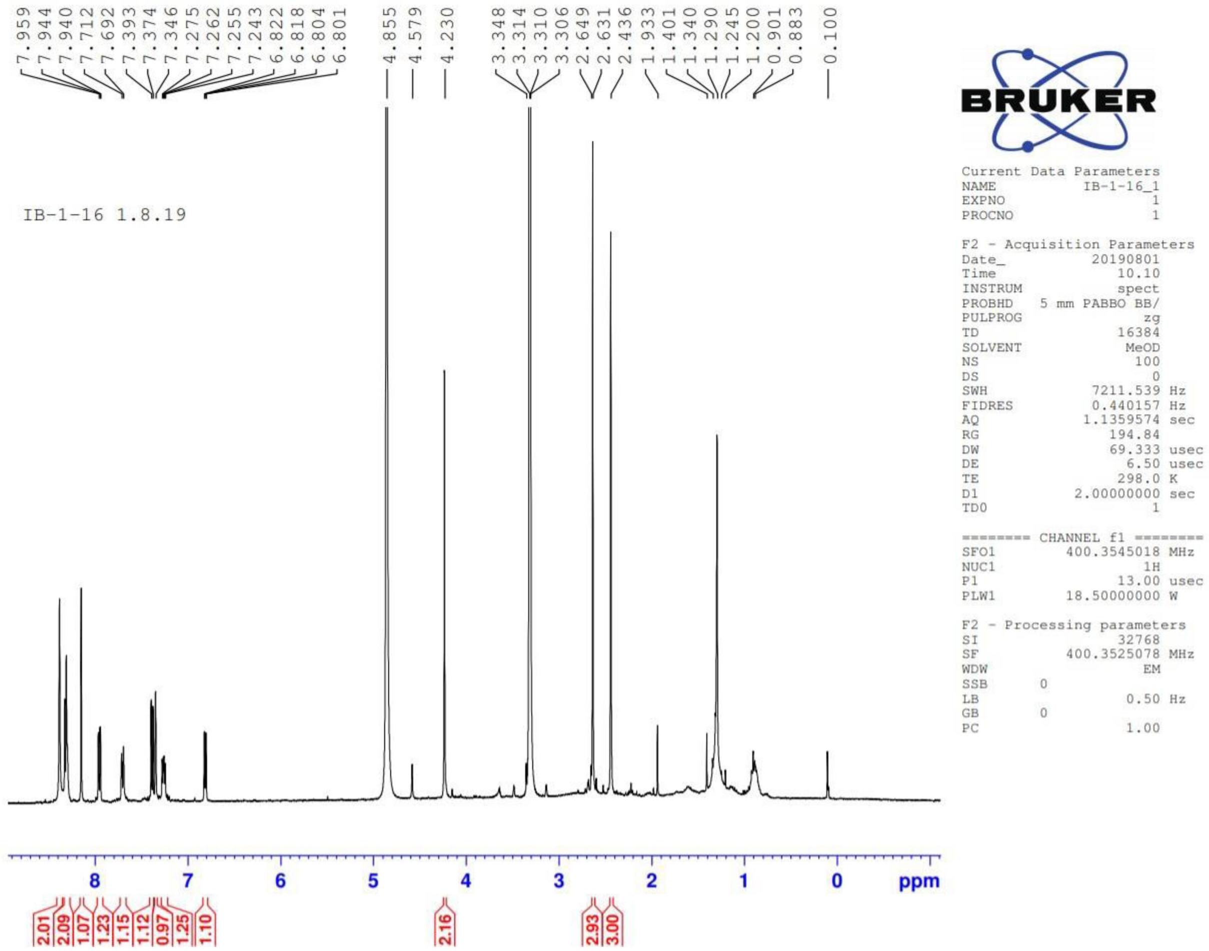

KAS-2-685 (4-((2-methyl-5-(8-methylimidazo[1,2-a]pyridin-2-yl)phenyl)sulfonyl)piperazin-1-ium chloride)-

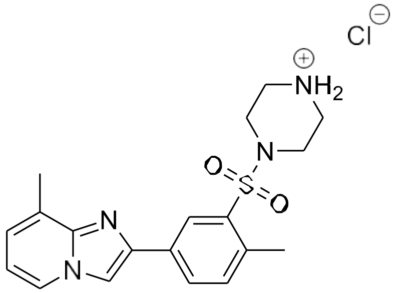

1 H NMR (300 MHz, DMSO-d_6) δ ppm 9.39 (s, 2H), 8.89 (s,1H), 8.66 (d, J= 6.45Hz, 1H), 8.39-8.53 (m, 1H), 8.29 (dd, J= 8.25, 0.42Hz, 1H), 7.71 (d, J= 8.04Hz, 1H), 7.62 (s, 1H), 7.30 (s, 1H), 3.33-3.47 (m, 4H), 3.05-3.26 (m, 4H), 2.68 (s, 3H), 2.64 (s, 3H).

LRMS (ESI+): m/z = 371.3.

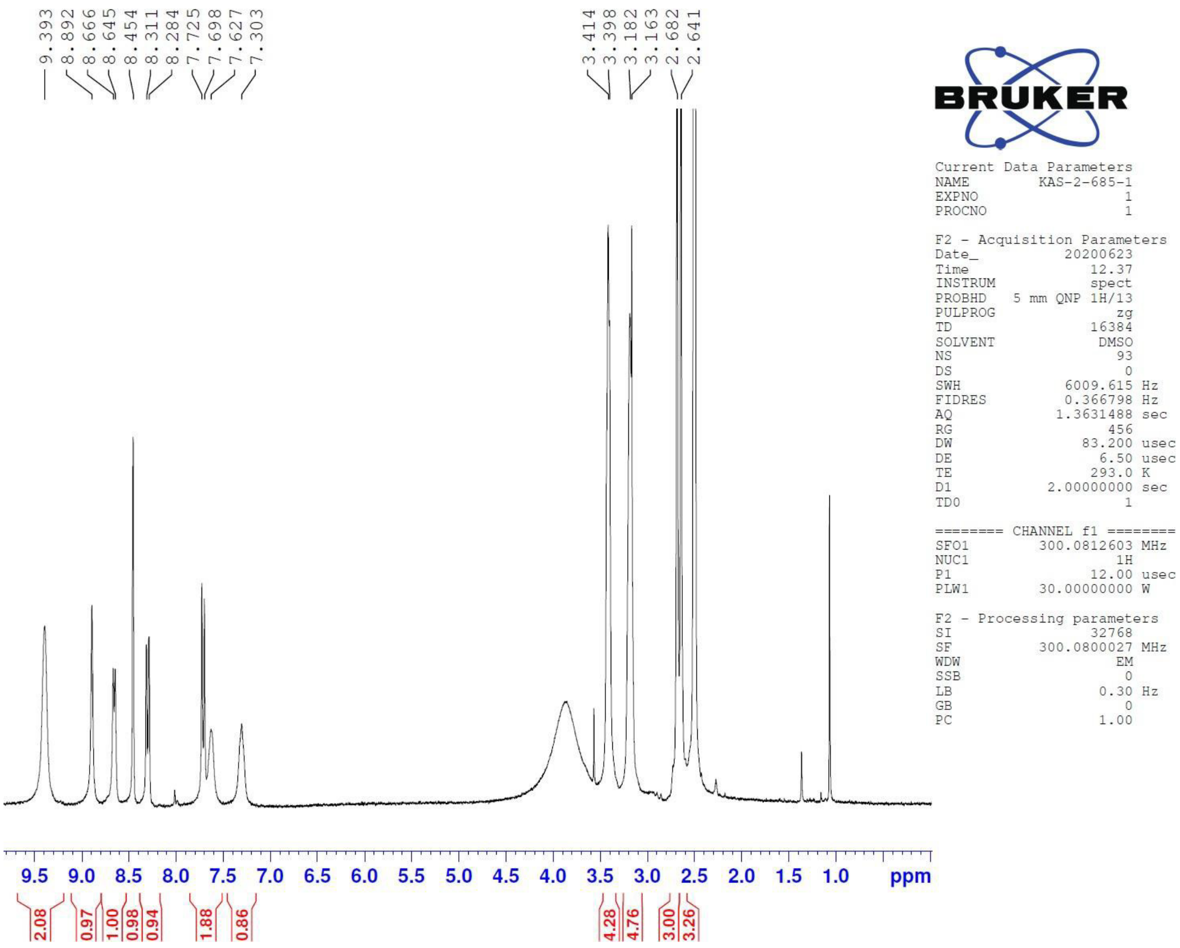

KAS-2-408 (8-methyl-2-(4-methyl-3-((4-methylpiperidin-1-yl)sulfonyl)phenyl)imidazo[1,2-a]pyridine)-

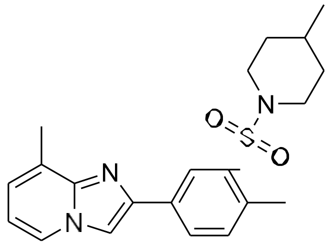

1 H NMR (400 MHz, DMSO-d_6) δ ppm 8.49 (s, 1H), 8.33-8.44 (m, 2H), 8.10 (dd, J= 8, 1.2Hz, 1H), 7.50 (d, J= 8Hz, 1H), 7.08 (d, J= 6.8Hz, 1H), 6.82 (dd, J= 6.4Hz, 1H), 3.61 (d, J= 12.08 Hz, 2H), 2.60-2.67 (m, 2H), 2.58 (s, 3H), 2.53 (s, 3H), 1.67 (d, J= 1.6 Hz, 2H), 1.44 (s, 1H), 1.02-1.18 (m, 2H), 0.88 (d, J= 7.6Hz, 3H).

LRMS (ESI+): m/z = 384.4.

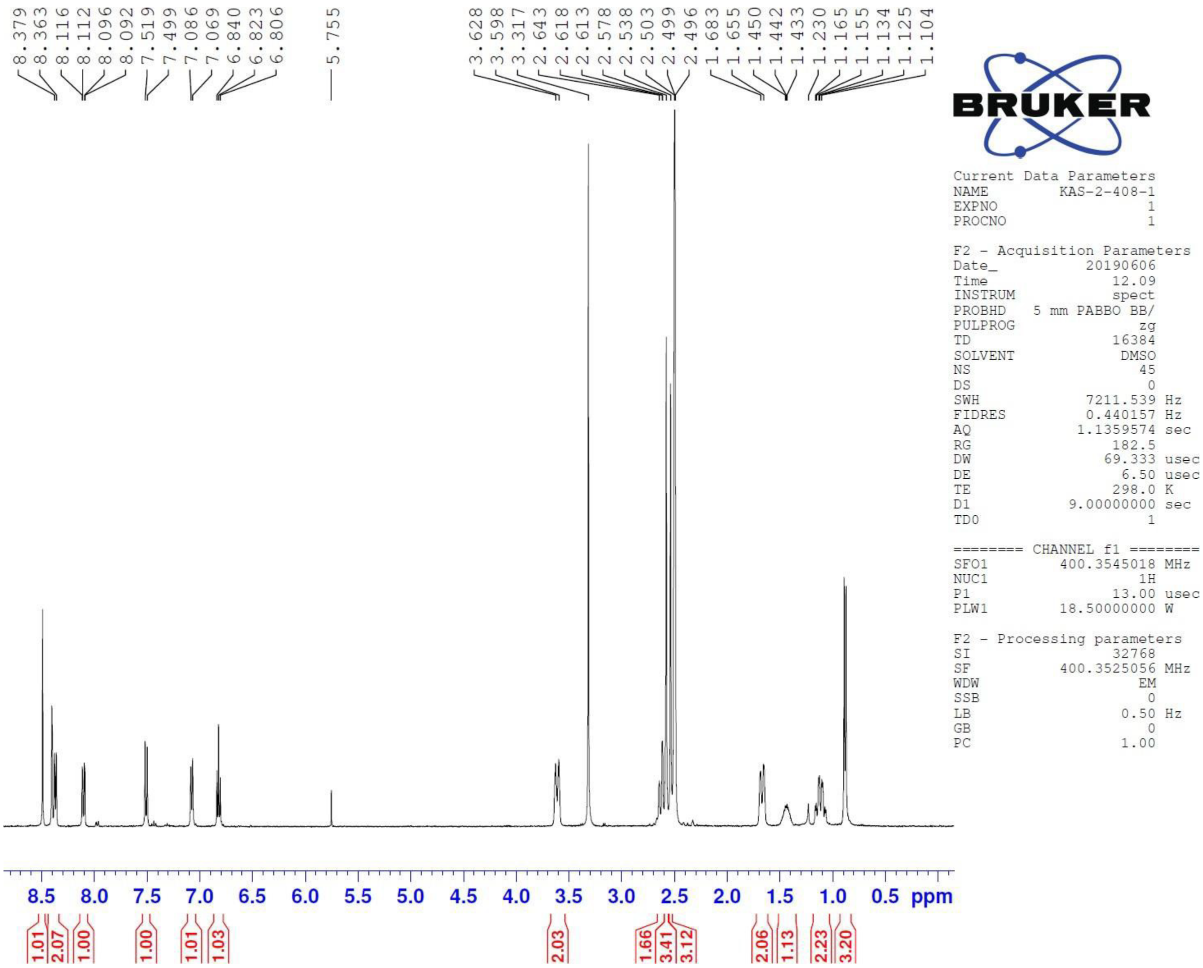

GS-1-37 (N-(4-chlorophenyl)-2-methyl-5-(7-methylimidazo[1,2-a]pyridin-2-yl)benzenesulfonamide)-

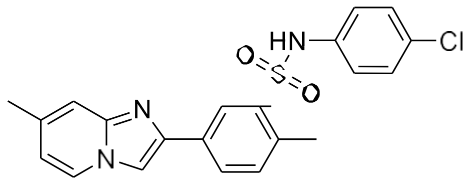

1 H NMR (400 MHz, MeOD) δ ppm 8.47 (d, J= 1.72Hz, 1H), 8.30 (d, J= 6.92Hz, 1H), 8.12 (s, 1H), 7.96 (dd, J= 8.08, 1.76Hz, 1H), 7.40 (d, J= 7.84Hz, 1H), 7.34 (s, 1H), m (7.15- 7.20, 2H), m (7.06- 7.12, 2H), 6.80 (dd, J= 6.8, 1.8Hz, 1H), 2.64 (s, 3H), 2.43 (s, 3H).

LRMS (ESI+): m/z = 412.3.

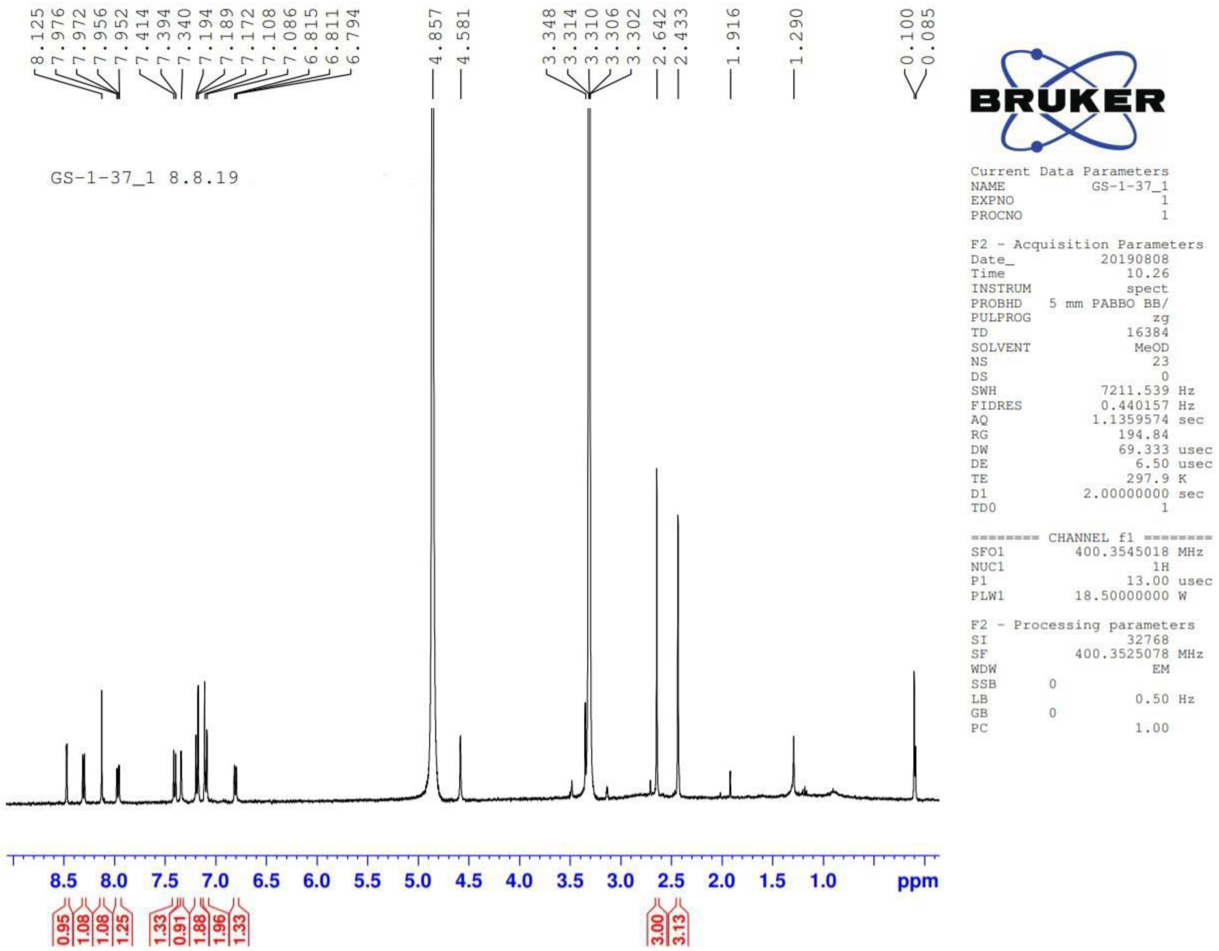

GS-1-43 (2-methyl-N-(3-(2-oxopyrrolidin-1-yl)propyl)-5-(5-phenyl-1,3,4-oxadiazol-2-yl)benzenesulfonamide)-

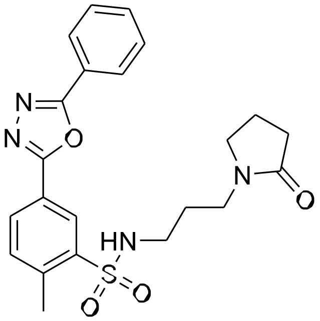

1 H NMR (400 MHz, MeOD) δ ppm 8.64 (d, J= 1.88, 1H), 8.26 (dd, J=7.52, 1.56Hz, 1H), 8.17 (dd, J= 7.52, 1.56Hz, 2H), m (7.58- 7.68, 4H), 3.40 (t, J= 8Hz, 2H), m (3.25-3.29, 2H), 2.94 (t, J=7.6Hz, 2H), 2.75 (s, 3H), 2.33 (t, J=8Hz, 2H), m (1.95-2.05, 2H), m (1.67-1.76, 2H).

LRMS (ESI+): m/z = 441.4.

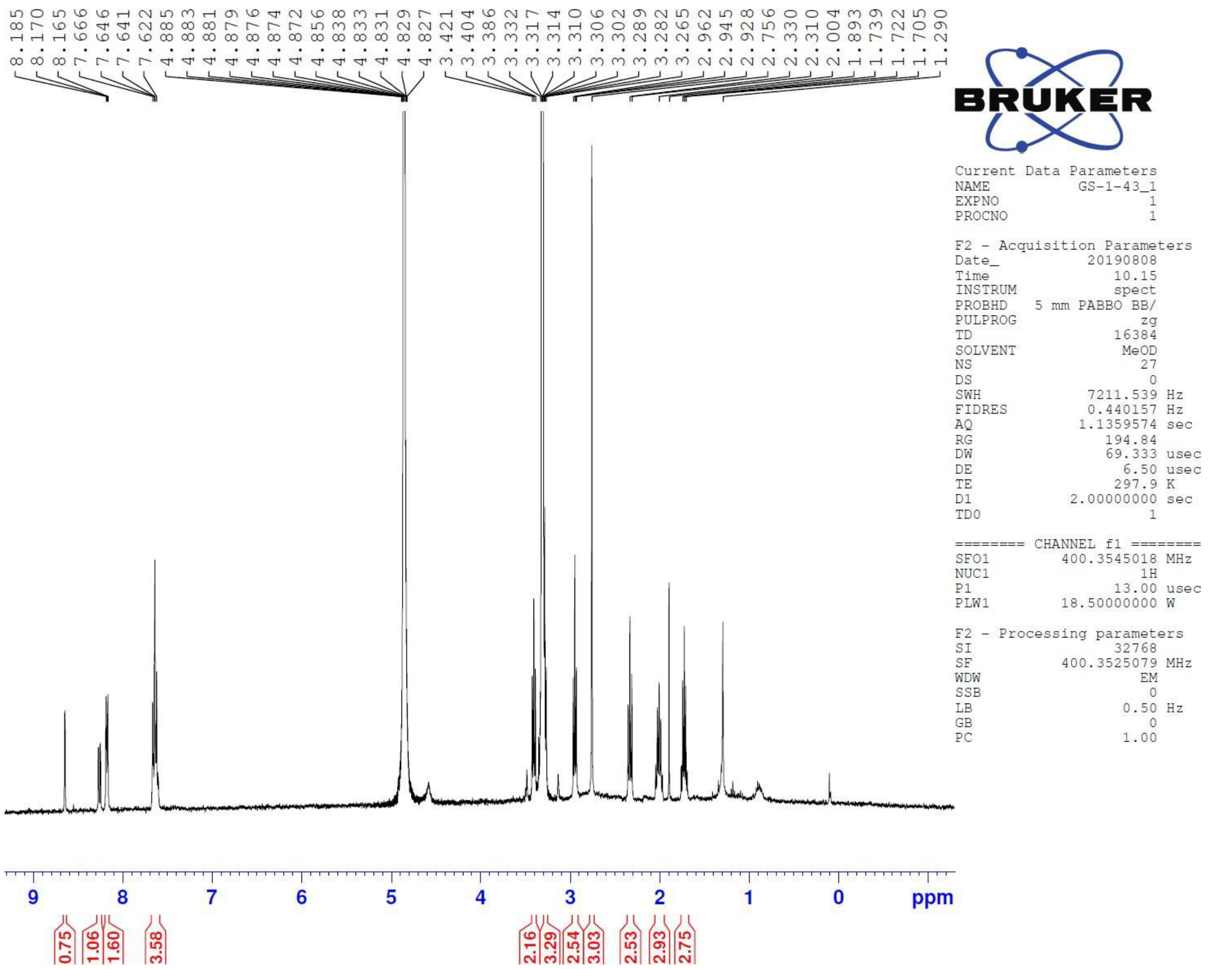

GS-1-16 (furan-2-yl(4-((2-methyl-5-(8-methylimidazo[1,2-a]pyridin-2-yl)phenyl)sulfonyl)piperazin-1-yl)methanone)-

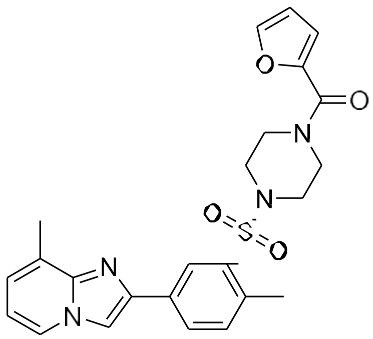

1 H NMR (400 MHz, MeOD) δ ppm 8.50 (d, J=1.48Hz, 1H), 8.30 (d, J=6.8Hz, 1H), 8.26 (s, 1H), 8.09 (dd, J= 7.86, 1.52Hz, 1H), 7.67 (s, 1H), 7.52 (d, J= 8.4Hz, 1H), 7.13 (d, J= 6.88Hz, 1H), 7.05 (d, J= 3.52Hz, 1H), 6.85 (t, J= 6.88Hz, 1H), 6.54-6.59 (m, 1H), 3.32-3.38 (m, 8H), 2.69 (s, 3H), 2.63 (s, 3H).

LRMS (ESI+): m/z =465.4.

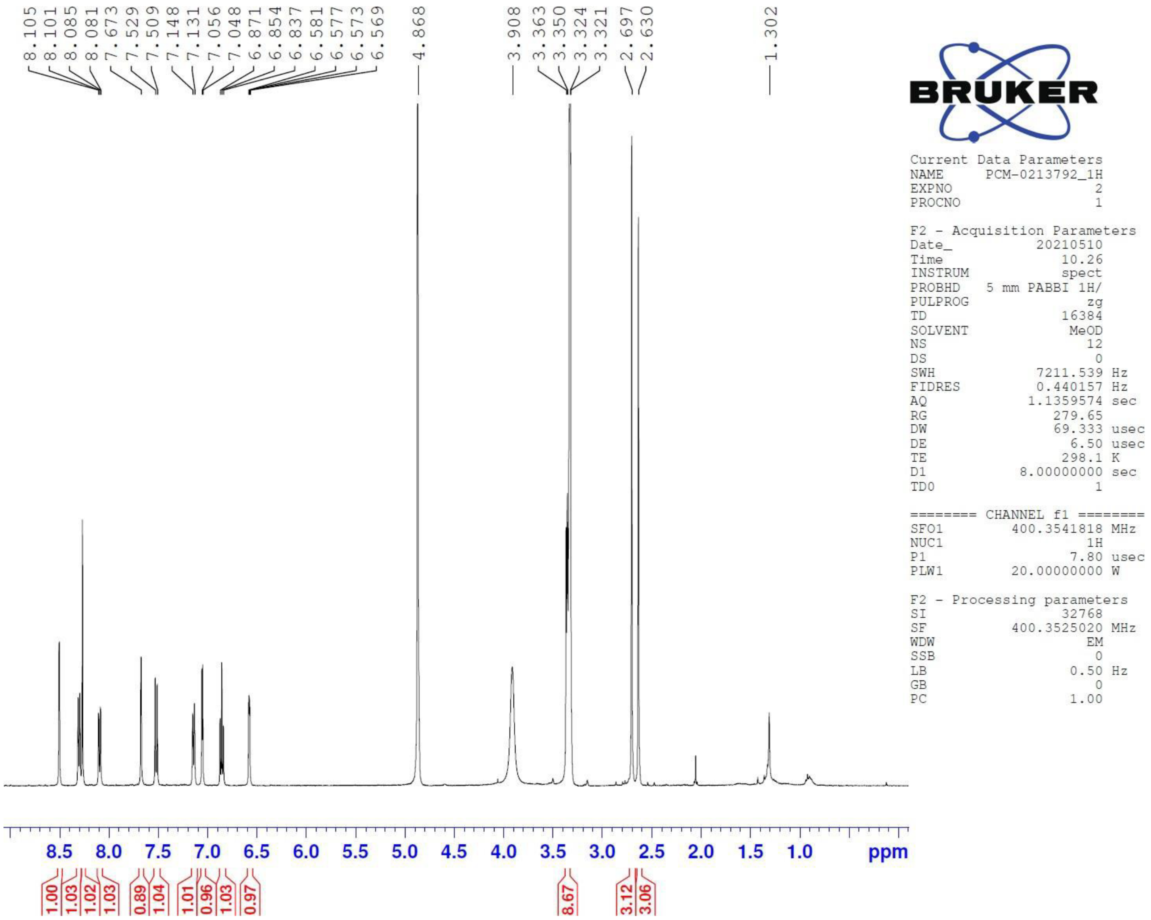

GS-1-14 (furan-2-yl(4-((2-methyl-5-(5-phenyl-1,3,4-oxadiazol-2-yl)phenyl)sulfonyl)piperazin-1-yl)methanone)-

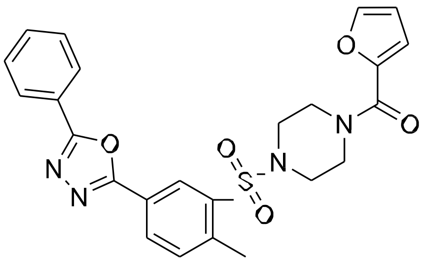

1 H NMR (400 MHz, MeOD) δ ppm 8.63 (d, J= 1.56Hz, 1H), 8.30 (dd, J= 7.87, 1.62Hz, 1H), 8.17 (dd, J= 7.46, 1.6Hz, 2H), 7.56-7.72 (m, 5H), 7.04 (d, J= 3.48Hz, 1H), 6.53-6.59 (m, 1H), 3.30-3.40 (m, 7H),2.75 (s, 3H).

LRMS (ESI+): m/z =479.3.

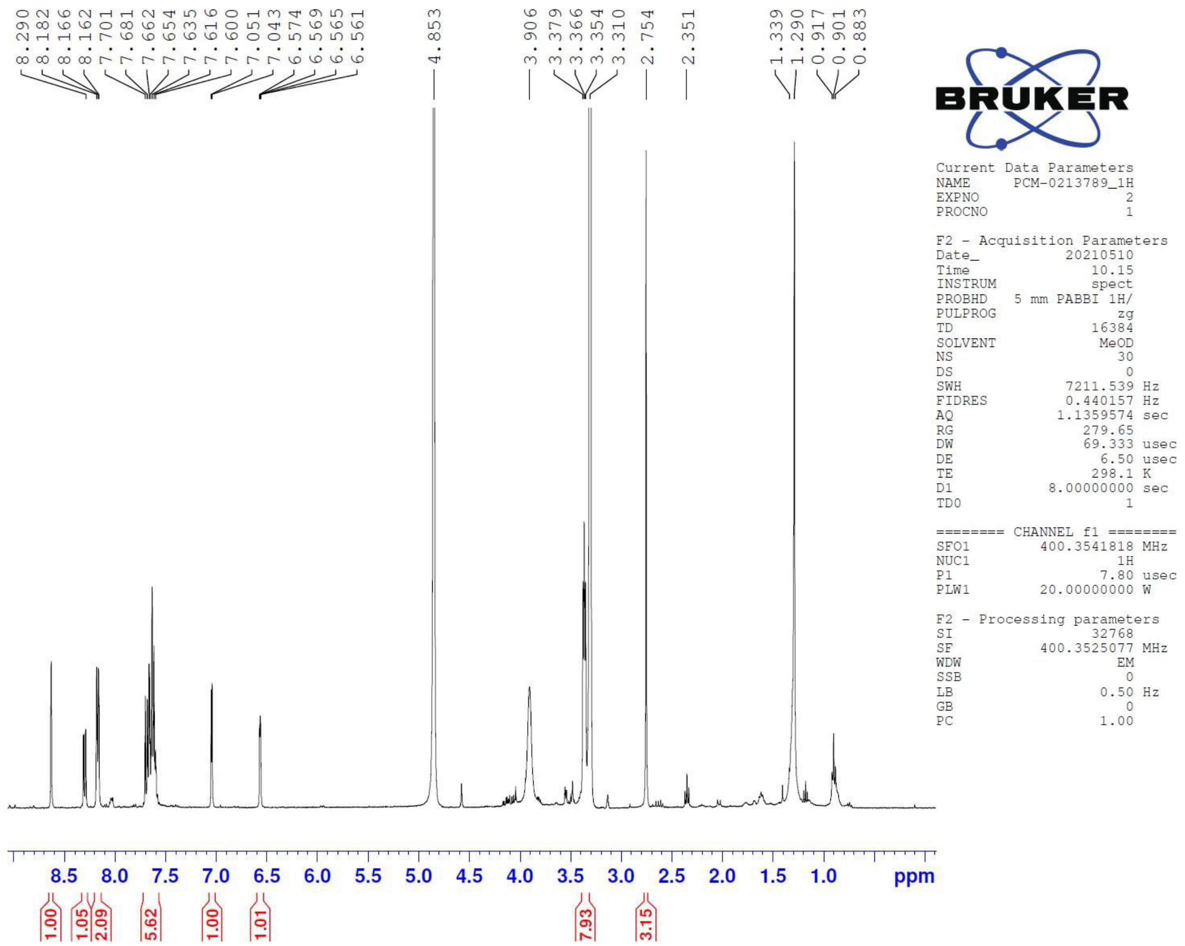

Table 3. Detailed Statistical Analysis.

Figure	Statistical analysis	Post hoc tests
**Fig. 1c**	(HCT116, 1.840e-017 ± 2.554e-017 AU, n=5; QR2Δ HCT116, -0.322 ± 0.091 AU, n=5; **unpaired t test**, t=3.523 df=8, p=0.0078)	
**Fig. 1d**	HCT116, 1 ± 0.222, n=6; QR2Δ HCT116, 0.070 ± 0.015, n=6**;** **unpaired t test**, t=4.169 df=10, p=0.0019	
**Fig. 1e**	HCT116, 1 ± 0.2406, n=6; QR2Δ HCT116, 2.295 ± 0.278, n=6; **unpaired t test**, t=3.52 df=10, p=0.0055	
**Fig. 1f**	HCT116, 1 ± 0.137, n=6; QR2Δ HCT116, 1.979 ± 0.172, n=6; **unpaired t test**, t=4.448 df=10, p=0.0012	
**Fig. 3d**	Vehicle-WT, 1.84e-017 ± 2.554e-017 AU, n=5; YB-800-WT -0.098 ± 0.038 AU, n=5; **unpaired t test**, t=2.557 df=8, p=0.0338	

<table>
<tr><td></td><td>/

Vehicle-QR2Δ -1.13e-017 ± 3.025e-017 AU, n=5; YB-800-QR2Δ -0.009 ± 0.049 AU, n=5; **unpaired t test**, t=0.2001 df=8, p=0.8464</td><td></td></tr>
<tr><td>**Fig. 3e**</td><td>HCT116-Vehicle 1 ± 0.083, n=6, n=6; HCT116-NG800 1.182 ± 0.137, n=6; **unpaired t test**, t=1.129 df=10, p=0.2851</td><td></td></tr>
<tr><td>**Fig. 3f**</td><td>HCT116-Vehicle 1.000 ± 0.039, n=6; HCT116-YB-800 1.478 ± 0.082, n=6; **Mann-Whitney test**, p=0.0022</td><td></td></tr>
<tr><td></td><td></td><td></td></tr>
<tr><td>**Fig. 4b**</td><td>Vehicle 56.410 ± 4.905 %; YB-808 68.67 ± 3.575 %; **unpaired t test**, t=2.037 df=33, p=0.0498</td><td></td></tr>
<tr><td>**Fig. 4e**</td><td>**Two Way Repeated Measures ANOVA**, Interaction F (3, 33) = 2.268, p=0.0989; Trial F (3, 33) = 28.14, p<0.0001; Treatment F (1, 11) = 3.951, p=0.0723; Subjects F (11, 33) = 8.476, p<0.0001)</td><td><table>
<tr><th>Sidak's multiple comparisons test</th><th>Mean Diff.</th><th>95.00% CI of diff.</th><th>Adjusted P Value</th></tr>
<tr><td></td><td></td><td></td><td></td></tr>
<tr><td>YB-537 - Vehicle</td><td></td><td></td><td></td></tr>
<tr><td>Baseline</td><td>2.006</td><td>-8.740 to 12.75</td><td>0.9813</td></tr>
<tr><td>CS/US 1</td><td>6.751</td><td>-3.996 to 17.50</td><td>0.3724</td></tr>
<tr><td>CS/US 2</td><td>9.311</td><td>-1.436 to 20.06</td><td>0.1129</td></tr>
<tr><td>CS/US 3</td><td>10.21</td><td>-0.5384 to 20.96</td><td>0.0685</td></tr>
</table></td></tr>
</table>

Sidak's multiple comparisons test	Mean Diff.	95.00% CI of diff.	Summary	Adjusted P Value
Vehicle				
CS/US 1 vs. Baseline	4.877	-1.472 to 11.23	ns	0.1814
CS/US 2 vs. Baseline	7.619	1.269 to 13.97	*	0.0136
CS/US 3 vs. Baseline	11.07	4.722 to 17.42	***	0.0002
CS/US 2 vs. CS/US 1	2.741	-3.608 to 9.091	ns	0.6509
CS/US 3 vs. CS/US 1	6.194	-0.1551 to 12.54	ns	0.0580
CS/US 3 vs. CS/US 2	3.453	-2.896 to 9.802	ns	0.4659
YB-537				
CS/US 1 vs. Baseline	9.622	2.764 to 16.48	**	0.0032
CS/US 2 vs. Baseline	14.92	8.065 to 21.78	****	<0.0001
CS/US 3 vs. Baseline	19.27	12.42 to 26.13	****	<0.0001
CS/US 2 vs. CS/US 1	5.302	-1.556 to 12.16	ns	0.1771
CS/US 3 vs. CS/US 1	9.652	2.794 to 16.51	**	0.0031
CS/US 3 vs. CS/US 2	4.350	-2.508 to 11.21	ns	0.3319

**Fig. 4f**	Vehicle 23.990 ± 3.078 %; YB-537 44.740 ± 5.484 %; **unpaired t test**, t=3.432 df=11, p=0.0056	

**Fig. 4g**	Vehicle 21.690 ± 2.453 %; YB-537 31.480 ± 7.058 %; **unpaired t test**, t=1.395 df=11, p=0.1904	

Fig. 6a

Two Way Repeated Measures ANOVA, Interaction $F (5, 155) = 1.78$, $p=0.1201$; Time $F (5, 155) = 24.9$, $p<0.0001$; Treatment $F (1, 31) = 3.539$, $p=0.0694$; Subjects $F (31, 155) = 5.139$, $p<0.0001$)

Sidak's multiple comparisons test	Mean Diff.	95.00% CI of diff.	Adjusted P Value
YB-537 - Vehicle			
Day 1	-2.551	-15.71 to 10.61	0.9963
Day 2	-3.229	-16.39 to 9.935	0.987
Day 3	-6.754	-19.92 to 6.409	0.6825
Day 4	-1.653	-14.82 to 11.51	0.9997
Day 5	-12.73	-25.89 to 0.4364	0.0637
Day 6	-12.86	-26.03 to 0.3006	0.0591

Sidak's multiple comparisons test	Mean Diff.	95.00% CI of diff.	Summary	Adjusted P Value
Vehicle				
Day 2 vs. Day 1	-4.551	-15.7 to 6.599	ns	0.9789
Day 3 vs. Day 1	-5.357	-16.51 to 5.793	ns	0.9202
Day 4 vs. Day 1	-17.08	-28.23 to -5.933	***	0.0002
Day 5 vs. Day 1	-13.47	-24.62 to -2.321	**	0.0066
Day 6 vs. Day 1	-19.95	-31.1 to -8.795	****	<0.0001
Day 3 vs. Day 2	-0.8059	-11.96 to 10.34	ns	>0.9999
Day 4 vs. Day 2	-12.53	-23.68 to -1.382	*	0.0155
Day 5 vs. Day 2	-8.921	-20.07 to 2.23	ns	0.2451
Day 6 vs. Day 2	-15.39	-26.54 to -4.244	***	0.001
Day 4 vs. Day 3	-11.73	-22.88 to -0.5764	*	0.0311
Day 5 vs. Day 3	-8.115	-19.26 to 3.035	ns	0.3859
Day 6 vs. Day 3	-14.59	-25.74 to -3.438	**	0.0022
Day 5 vs. Day 4	3.612	-7.538 to 14.76	ns	0.9979
Day 6 vs. Day 4	-2.862	-14.01 to 8.288	ns	0.9999
Day 6 vs. Day 5	-6.474	-17.62 to 4.677	ns	0.7416
YB-537				

Day 2 vs. Day 1	-5.229	-16.72 to 6.264	ns	0.9472
Day 3 vs. Day 1	-9.56	-21.05 to 1.933	ns	0.1962
Day 4 vs. Day 1	-16.19	-27.68 to -4.692	***	0.0007
Day 5 vs. Day 1	-23.65	-35.14 to -12.15	****	<0.0001
Day 6 vs. Day 1	-30.26	-41.75 to -18.76	****	<0.0001
Day 3 vs. Day 2	-4.331	-15.82 to 7.162	ns	0.99
Day 4 vs. Day 2	-10.96	-22.45 to 0.537	ns	0.0752
Day 5 vs. Day 2	-18.42	-29.91 to -6.925	****	<0.0001
Day 6 vs. Day 2	-25.03	-36.52 to -13.53	****	<0.0001
Day 4 vs. Day 3	-6.625	-18.12 to 4.868	ns	0.751
Day 5 vs. Day 3	-14.09	-25.58 to -2.594	**	0.0055
Day 6 vs. Day 3	-20.7	-32.19 to -9.204	****	<0.0001
Day 5 vs. Day 4	-7.463	-18.96 to 4.031	ns	0.5742
Day 6 vs. Day 4	-14.07	-25.57 to -2.579	**	0.0055
Day 6 vs. Day 5	-6.609	-18.1 to 4.884	ns	0.754

Fig. 6b

Two Way Repeated Measures ANOVA, Interaction F (5, 80) = 1.063, p=0.3870; Time F (5, 80) = 12.09, p<0.0001; Treatment F (1, 16) = 2.837, p=0.1115; Subjects F (16, 80) = 5.805, p<0.0001

Sidak's multiple comparisons test	Mean Diff.	95.00% CI of diff.	Adjusted P Value
YB-537 - Vehicle			
Day 1	-1.535	-20.25 to 17.18	>0.9999
Day 2	-6.867	-25.59 to 11.85	0.907
Day 3	-11.96	-30.68 to 6.759	0.4295
Day 4	-3.817	-22.54 to 14.9	0.9949
Day 5	-12.33	-31.05 to 6.386	0.3933
Day 6	-15.11	-33.83 to 3.614	0.1806

	Mean Diff.	95.00% CI of diff.	Summary	Adjusted P Value
Sidak's multiple comparisons test				
Vehicle				
Day 2 vs. Day 1	-1.587	-17.26 to 14.08	ns	>0.9999
Day 3 vs. Day 1	-3.437	-19.11 to 12.23	ns	>0.9999
Day 4 vs. Day 1	-14.97	-30.64 to 0.7001	ns	0.0732

Day 5 vs. Day 1	-12.65	-28.32 to 3.022	ns	0.2277
Day 6 vs. Day 1	-17	-32.67 to -1.333	*	0.0232
Day 3 vs. Day 2	-1.85	-17.52 to 13.82	ns	>0.9999
Day 4 vs. Day 2	-13.38	-29.05 to 2.287	ns	0.163
Day 5 vs. Day 2	-11.06	-26.73 to 4.609	ns	0.4251
Day 6 vs. Day 2	-15.42	-31.09 to 0.2538	ns	0.0575
Day 4 vs. Day 3	-11.53	-27.2 to 4.137	ns	0.3586
Day 5 vs. Day 3	-9.211	-24.88 to 6.459	ns	0.7132
Day 6 vs. Day 3	-13.57	-29.24 to 2.104	ns	0.1494
Day 5 vs. Day 4	2.322	-13.35 to 17.99	ns	>0.9999
Day 6 vs. Day 4	-2.033	-17.7 to 13.64	ns	>0.9999
Day 6 vs. Day 5	-4.356	-20.03 to 11.31	ns	0.9996
YB-537				
Day 2 vs. Day 1	-6.919	-22.59 to 8.752	ns	0.9548
Day 3 vs. Day 1	-13.86	-29.53 to 1.808	ns	0.1293
Day 4 vs. Day 1	-17.25	-32.92 to -1.581	*	0.02
Day 5 vs. Day 1	-23.45	-39.12 to -7.776	***	0.0003
Day 6 vs. Day 1	-30.57	-46.24 to -14.9	****	<0.0001
Day 3 vs. Day 2	-6.944	-22.61 to 8.726	ns	0.9534
Day 4 vs. Day 2	-10.33	-26 to 5.337	ns	0.5368
Day 5 vs. Day 2	-16.53	-32.2 to -0.8573	*	0.0307
Day 6 vs. Day 2	-23.66	-39.33 to -7.985	***	0.0003
Day 4 vs. Day 3	-3.389	-19.06 to 12.28	ns	>0.9999
Day 5 vs. Day 3	-9.583	-25.25 to 6.087	ns	0.6559
Day 6 vs. Day 3	-16.71	-32.38 to -1.041	*	0.0276
Day 5 vs. Day 4	-6.194	-21.86 to 9.476	ns	0.9825
Day 6 vs. Day 4	-13.32	-28.99 to 2.348	ns	0.1677
Day 6 vs. Day 5	-7.128	-22.8 to 8.543	ns	0.9428

Fig. 6c	**Two Way Repeated Measures ANOVA**, Interaction F (5, 65) = 1.095, p=0.3719; Time F (5, 65) = 11.89, p<0.0001; Treatment F (1, 13) = 0.6717, p=0.4272; Subjects F (13, 65) = 4.14, p<0.0001

Sidak's multiple comparisons test	Mean Diff.	95.00% CI of diff.	Summary	Adjusted P Value
YB-537 - Vehicle				
Day 1	-3.48	-23.08 to 16.12	ns	0.9976
Day 2	1.348	-18.25 to 20.94	ns	>0.9999
Day 3	0.008036	-19.59 to 19.6	ns	>0.9999
Day 4	1.166	-18.43 to 20.76	ns	>0.9999
Day 5	-12.99	-32.58 to 6.606	ns	0.3834
Day 6	-10.08	-29.67 to 9.52	ns	0.6709

Sidak's multiple comparisons test	Mean Diff.	95.00% CI of diff.	Summary	Adjusted P Value
Vehicle				
Day 2 vs. Day 1	-7.885	-25.16 to 9.384	ns	0.9388
Day 3 vs. Day 1	-7.517	-24.79 to 9.753	ns	0.958
Day 4 vs. Day 1	-19.46	-36.73 to -2.191	*	0.0159
Day 5 vs. Day 1	-14.4	-31.67 to 2.872	ns	0.1869
Day 6 vs. Day 1	-23.25	-40.52 to -5.984	**	0.0018
Day 3 vs. Day 2	0.3687	-16.9 to 17.64	ns	>0.9999
Day 4 vs. Day 2	-11.58	-28.84 to 5.695	ns	0.5044
Day 5 vs. Day 2	-6.513	-23.78 to 10.76	ns	0.9881
Day 6 vs. Day 2	-15.37	-32.64 to 1.901	ns	0.1231
Day 4 vs. Day 3	-11.94	-29.21 to 5.326	ns	0.4531
Day 5 vs. Day 3	-6.881	-24.15 to 10.39	ns	0.9803
Day 6 vs. Day 3	-15.74	-33.01 to 1.532	ns	0.1042
Day 5 vs. Day 4	5.063	-12.21 to 22.33	ns	0.9992
Day 6 vs. Day 4	-3.794	-21.06 to 13.48	ns	>0.9999
Day 6 vs. Day 5	-8.856	-26.13 to 8.414	ns	0.8626
YB-537				
Day 2 vs. Day 1	-3.057	-21.52 to 15.41	ns	>0.9999

**Day 3 vs. Day 1**	**-4.029**	**-22.49 to 14.43**	**ns**	**>0.9999**
**Day 4 vs. Day 1**	**-14.81**	**-33.28 to 3.648**	**ns**	**0.2324**
**Day 5 vs. Day 1**	**-23.91**	**-42.37 to - 5.445**	******	**0.0031**
**Day 6 vs. Day 1**	**-29.85**	**-48.31 to - 11.39**	********	**<0.0001**
**Day 3 vs. Day 2**	**-0.9714**	**-19.43 to 17.49**	**ns**	**>0.9999**
**Day 4 vs. Day 2**	**-11.76**	**-30.22 to 6.705**	**ns**	**0.5872**
**Day 5 vs. Day 2**	**-20.85**	**-39.31 to - 2.388**	*****	**0.0156**
**Day 6 vs. Day 2**	**-26.79**	**-45.26 to - 8.331**	*******	**0.0006**
**Day 4 vs. Day 3**	**-10.79**	**-29.25 to 7.677**	**ns**	**0.7159**
**Day 5 vs. Day 3**	**-19.88**	**-38.34 to - 1.416**	*****	**0.0253**
**Day 6 vs. Day 3**	**-25.82**	**-44.28 to - 7.359**	******	**0.001**
**Day 5 vs. Day 4**	**-9.093**	**-27.56 to 9.369**	**ns**	**0.8945**
**Day 6 vs. Day 4**	**-15.04**	**-33.5 to 3.427**	**ns**	**0.2141**
**Day 6 vs. Day 5**	**-5.943**	**-24.41 to 12.52**	**ns**	**0.9976**

**Fig. 6d**	Vehicle 0.0543 ± 0.07876, n=17; YB-537 0.1785 ± 0.08786, n=16; **unpaired t test**, t=1.055 df=31, p=0.2995 / **One sample t test** Vehicle vs. 0, t=0.6894 df=16, p=0.5004; **One sample t test** YB-537 vs 0, t=2.032 df=15, p=0.0603	
**Fig. 6e**	Vehicle 0.06542 ± 0.1076, n=9; YB-537 0.06432 ± 0.1285, n=9; **unpaired t test**, t=0.006588	

	df=16, p=0.9948<br><br>/<br><br>**One sample t test** Vehicle vs 0, t=0.6081 df=8, p=0.5600;<br><br>**One sample t test** YB-537 vs 0, t=0.5006 df=8, p=0.6302	
**Fig. 6f**	Vehicle 0.04179 ± 0.1233, n=8;<br>YB-537 0.3253 ± 0.09705, n=7;<br>**unpaired t test**, t=1.768 df=13, p=0.1004<br><br>/<br><br>**One sample t test** Vehicle vs 0, t=0.3389 df=7, p=0.7447;<br><br>**One sample t test** YB-537 vs 0, t=3.352 df=6, p=0.0154	
**Fig. 6g**	Vehicle 23.78 ± 3.357%, n=17;<br>YB-537 34.93 ± 2.513%, n=16;<br>**unpaired t test**, t=2.634 df=31, p=0.0131	

**Fig. 6h**	Vehicle 30.95 ± 4.421%, n=9; YB-537 39.51 ± 2.666%, n=9; **unpaired t test**, t=1.658 df=16, p=0.1168	
**Fig. 6i**	Vehicle 15.71 ± 3.465%, n=8; YB-537 29.03 ± 3.69%, n=7; **unpaired t test**, t=2.632 df=13, p=0.0207	
**Fig. 6j**	Vehicle 13.56 ± 2.409%, n=17; YB-537 16.47 ± 1.689%, n=16; **Mann-Whitney test**, p=0.1000	
**Fig. 6k**	Vehicle 14.97 ± 4.12%, n=9; YB-537 19.43 ± 2.289%, n=9; **Mann-Whitney test**, p=0.0939	

**Fig. 6l**	Vehicle 11.97 ± 2.385%, n=8; YB-537 12.66 ± 1.739%, n=7; **unpaired t test**, t=0.2266 df=13, p=0.8243	

**Fig. 7a**	Both sexes – Vehicle 0.053 ± 0.005, n=16; YB-537 0.049 ± 0.003, n=16; **Mann-Whitney test**, p=0.8965<br><br>/<br><br>Males – Vehicle 0.055 ± 0.007, n=8; YB-537 0.053 ± 0.005, n=9; **Mann-Whitney test**, p=0.6730<br><br>/<br><br>Females – Vehicle 0.055 ± 0.007, n=8; YB-537 0.053 ± 0.005, n=9; **unpaired t test**, t=0.2375 df=15, p=0.8155	
**Fig. 7b**	Both sexes – Vehicle 0.049 ± 0.0031, n=17; YB-537 0.040 ± 0.003, n=16; **unpaired t test**, t=2.015 df=31, p=0.0526	

	/ <br>Males - Vehicle 0.041 ± 0.002, n=9; YB-537 0.038 ± 0.004, n=9; **unpaired t test**, t=0.5887 df=16, p=0.5643) <br>/ <br>Females - Vehicle 0.059 ± 0.003, n=8; YB-537 0.044 ± 0.004, n=7; **unpaired t test**, t=2.56 df=13, p=0.0237	
**Fig. 7c**	Both sexes – Vehicle 0.054 ± 0.004, n=17; YB-537 0.048 ± 0.006, n=16; **unpaired t test**, t=0.7351 df=31, p=0.4678 <br>/ <br>Males - Vehicle 0.055 ± 0.006, n=9; YB-537 0.064 ± 0.006, n=9; **unpaired t test**, t=0.9768 df=16, p=0.3432 <br>/ <br>Females - Vehicle 0.052 ± 0.008, n=8; YB-537 0.027 ± 0.006, n=7; **unpaired t test**, t=2.373 df=13,	

	p=0.0337	
**Fig. 7d**	Both sexes - Vehicle 0.039 ± 0.002, n=17; YB-537 0.032 ± 0.002, n=16; **unpaired t test**, t=1.851 df=31, p=0.0738<br><br>/<br><br>Males - Vehicle 0.040 ± 0.002, n=9; YB-537 0.038 ± 0.003, n=9; **unpaired t test**, t=0.5915 df=16, p=0.5625<br><br>/<br><br>Females - Vehicle 0.037 ± 0.004, n=8; YB-537 0.025 ± 0.002, n=7; **unpaired t test**, t=2.309 df=13, p=0.0380	

Fig. 8a

Two Way Repeated Measures ANOVA, Interaction F (6, 51) = 0.5436, p=0.7725; Trial F (3, 51) = 117.4, p<0.0001; Treatment F (2, 17) = 1.183, p=0.3303; Subjects F (17, 51) = 7.606, p<0.0001

Tukey's multiple comparisons test	Predicted mean diff.	95.00% CI of diff.	Adjusted P Value
**Baseline**			
Vehicle vs. YB-537	-3.614	-16.68 to 9.456	0.7859
WT vs. YB-537	-0.8792	-16.46 to 14.71	0.9900
WT vs. Vehicle	2.735	-13.52 to 18.99	0.9144
**CS/US 1**			
Vehicle vs. YB-537	-9.965	-23.03 to 3.105	0.1686
WT vs. YB-537	-5.145	-20.73 to 10.44	0.7098
WT vs. Vehicle	4.820	-11.44 to 21.08	0.7582
**CS/US 2**			
Vehicle vs. YB-537	-7.862	-20.93 to 5.208	0.3257
WT vs. YB-537	-1.959	-17.54 to 13.63	0.9512
WT vs. Vehicle	5.902	-10.35 to 22.16	0.6609
**CS/US 3**			
Vehicle vs. YB-537	-6.376	-19.45 to 6.694	0.4757
WT vs. YB-537	2.306	-13.28 to 17.89	0.9332
WT vs. Vehicle	8.681	-7.574 to 24.94	0.4115

Tukey's multiple comparisons test	Predicted mean diff.	95.00% CI of diff.	Adjusted P Value
**YB-537**			
CS/US 1 vs. Baseline	23.[illegible]9	15.67 to 32.31	<0.0001
CS/US 2 vs. Baseline	33.[illegible]9	25.27 to 41.91	<0.0001
CS/US 3 vs. Baseline	38.[illegible]3	30.61 to 47.25	<0.0001
CS/US 2 vs. CS/US 1	9.5[illegible]7	1.275 to 17.92	0.0178
CS/US 3 vs. CS/US 1	14.[illegible]4	6.615 to 23.26	<0.0001
CS/US 3 vs. CS/US 2	5.3[illegible]0	-2.982 to 13.66	0.3321
**Vehicle**			
CS/US 1 vs. Baseline	17.[illegible]4	8.204 to 27.08	<0.0001
CS/US 2 vs. Baseline	29.[illegible]4	19.90 to 38.78	<0.0001
CS/US 3 vs. Baseline	36.[illegible]7	26.73 to 45.60	<0.0001
CS/US 2 vs. CS/US 1	11.[illegible]0	2.264 to 21.14	0.0095
CS/US 3 vs. CS/US 1	18.[illegible]3	9.090 to 27.96	<0.0001
CS/US 3 vs. CS/US 2	6.8[illegible]6	-2.610 to 16.26	0.2321
**WT**			
CS/US 1 vs. Baseline	19.[illegible]3	7.242 to 32.21	0.0006
CS/US 2 vs. Baseline	32.[illegible]1	20.02 to 44.99	<0.0001
CS/US 3 vs. Baseline	42.[illegible]1	29.63 to 54.60	<0.0001
CS/US 2 vs. CS/US 1	12.[illegible]8	0.2999 to 25.27	0.0429
CS/US 3 vs. CS/US 1	22.[illegible]9	9.905 to 34.87	<0.0001
CS/US 3 vs. CS/US 2	9.6[illegible]5	-2.878 to 22.09	0.1858

Fig. 8b

YB-537 44.08 ± 6.526 %, n=9; Vehicle 24.48 ± 4.013 %, n=7; WT 49.75 ± 3.859 %, n=4; **One Way ANOVA**, F(2,17)=4.686, p=0.0239.

Tukey's multiple comparison	Mean Diff.	95.00% CI of diff.	Adjusted P Value
YB-537 vs. Vehicle	19.60	-0.03495 to 39.24	0.0504
YB-537 vs. WT	-5.667	-29.08 to 17.75	0.8108
Vehicle vs. WT	-25.27	-49.69 to -0.8459	0.0420

Fig. 8c

YB-537 51.93 ± 4.461 %, n=9; Vehicle 36.53 ± 5.312 %, n=7; WT

<table>
<tr><td></td><td>54.84 ± 9.183 %, n=4; One Way ANOVA, F(2,17)=2.884, p=0.0835.</td><td></td></tr>
<tr><td></td><td></td><td></td></tr>
<tr><td>Fig. 9a Hipp.</td><td>Vehicle 0.0144 ± 0.0009, n=7; WT 0.0123 ± 0.0025, n=4; YB-537 0.0164 ± 0.0012, n=9; one-way ANOVA, F (2, 17) = 1.818, p=0.1926.</td><td></td></tr>
<tr><td>Fig. 9a Ctx.</td><td>Vehicle 0.0173 ± 0.0010, n=7; WT 0.0110 ± 0.0004, n=4; YB-537 0.0132 ± 0.0007, n=9; one-way ANOVA, F (2, 17) = 10.79, p=0.0009.</td><td><table>
<tr><th>Tukey’s multiple comparison</th><th>Mean Diff.</th><th>95.00% CI of diff.</th><th>Adjusted P Value</th></tr>
<tr><td>Vehicle vs. WT</td><td>0.006281</td><td>0.002531 to 0.01003</td><td>0.0013</td></tr>
<tr><td>Vehicle vs. YB-537</td><td>0.004138</td><td>0.001123 to 0.007154</td><td>0.0070</td></tr>
<tr><td>WT vs. YB-537</td><td>-0.002143</td><td>-0.005738 to 0.001453</td><td>0.3029</td></tr>
</table></td></tr>
<tr><td>Fig. 9b Hipp.</td><td>Vehicle 0.0074 ± 0.0014, n=7; WT 0.0012 ± 0.0001, n=4; YB-537 0.0029 ± 0.0006, n=9; Kruskal-Wallis, p<0.0001.</td><td><table>
<tr><th>Dunn’s multiple comparison</th><th>Mean Rank Diff.</th><th>Adjusted P Value</th></tr>
<tr><td>Vehicle vs. WT</td><td>13.04</td><td>0.0013</td></tr>
<tr><td>Vehicle vs. YB-537</td><td>7.063</td><td>0.0535</td></tr>
<tr><td>WT vs. YB-537</td><td>-5.972</td><td>0.2789</td></tr>
</table></td></tr>
<tr><td>Fig. 9b Ctx.</td><td>Vehicle 0.0142 ± 0.0027, n=7; WT 0.0018 ± 0.00008, n=4; YB-537 0.0025 ± 0.0006, n=9; Kruskal-Wallis, p=0.0003.</td><td><table>
<tr><th>Dunn’s multiple comparison</th><th>Mean Rank Diff.</th><th>Adjusted P Value</th></tr>
<tr><td>Vehicle vs. WT</td><td>8.714</td><td>0.0563</td></tr>
<tr><td>Vehicle vs. YB-537</td><td>9.937</td><td>0.0026</td></tr>
<tr><td>WT vs. YB-537</td><td>1.222</td><td>>0.9999</td></tr>
</table></td></tr>
<tr><td>Fig. 9c Hipp.</td><td>Vehicle 0.0325 ± 0.0052, n=7; WT 0.0399 ± 0.0047, n=4; YB-537 0.0311 ± 0.0033, n=9; one-way ANOVA, F (2, 17) = 0.8324,</td><td></td></tr>
</table>

<table>
<tr><td></td><td>p=0.4520.</td><td></td></tr>
<tr><td>Fig. 9c Ctx.</td><td>Vehicle 0.0180 ± 0.0032, n=7; WT 0.0181 ± 0.0015, n=4; YB-537 0.0171 ± 0.0018, n=9; one-way ANOVA, F (2, 17) = 0.0454, p=0.9557.</td><td></td></tr>
<tr><td>Fig. 9d Hipp.</td><td>Vehicle 0.0267 ± 0.0022, n=7; WT 0.0191 ± 0.0003, n=4; YB-537 0.0209 ± 0.0012, n=9; Kruskal-Wallis, p=0.0011.</td><td><table>
<tr><th>Dunn’s multiple comparison</th><th>Mean Rank Diff.</th><th>Adjusted P Value</th></tr>
<tr><td>Vehicle vs. WT</td><td>11.50</td><td>0.0058</td></tr>
<tr><td>Vehicle vs. YB-537</td><td>7.111</td><td>0.0512</td></tr>
<tr><td>WT vs. YB-537</td><td>-4.389</td><td>0.6510</td></tr>
</table></td></tr>
<tr><td>Fig. 9d Ctx.</td><td>Vehicle 0.0336 ± 0.0017, n=7; WT 0.0268 ± 0.0013, n=4; YB-537 0.0218 ± 0.0011, n=9; Kruskal-Wallis, p<0.0001.</td><td><table>
<tr><th>Dunn’s multiple comparison</th><th>Mean Rank Diff.</th><th>Adjusted P Value</th></tr>
<tr><td>Vehicle vs. WT</td><td>4.536</td><td>0.6638</td></tr>
<tr><td>Vehicle vs. YB-537</td><td>10.84</td><td>0.0008</td></tr>
<tr><td>WT vs. YB-537</td><td>6.306</td><td>0.2284</td></tr>
</table></td></tr>
<tr><td>Fig. 9e Hipp.</td><td>Vehicle 0.0355 ± 0.0042, n=7; WT 0.0265 ± 0.0024, n=4; YB-537 0.0239 ± 0.0027, n=9; One Way ANOVA, F(2,17)=3.361, p=0.0589.</td><td><table>
<tr><th>Tukey’s multiple comparison</th><th>Mean Diff.</th><th>95.00% CI of diff.</th><th>Adjusted P Value</th></tr>
<tr><td>Vehicle vs. WT</td><td>0.009004</td><td>-0.005500 to 0.02351</td><td>0.2757 Vehicle</td></tr>
<tr><td>vs. YB-537</td><td>0.01157</td><td>-9.293e-005 to 0.02323</td><td>0.0520</td></tr>
<tr><td>WT vs. YB-537</td><td>0.002565</td><td>-0.01134 to 0.01647</td><td>0.8847</td></tr>
</table></td></tr>
<tr><td>Fig. 9e Ctx.</td><td>Vehicle 0.0294 ± 0.0056, n=7; WT 0.0077 ± 0.0019, n=4; YB-537 0.010 ± 0.0035, n=9; Kruskal-Wallis, p=0.0116.</td><td><table>
<tr><th>Dunn’s multiple comparison</th><th>Mean Rank Diff.</th><th>Adjusted P Value</th></tr>
<tr><td>Vehicle vs. WT</td><td>7.321</td><td>0.1450</td></tr>
<tr><td>Vehicle vs. YB-537</td><td>8.016</td><td>0.0215</td></tr>
<tr><td>WT vs. YB-537</td><td>0.6944</td><td>>0.9999</td></tr>
</table></td></tr>
</table>

Enamine Biology Services

STUDY REPORT P022621a

Pharmacokinetic Study of NG-537 in C57BL/6J Mice Following Intravenous and Peroral Administration

Date: March 14, 2021

Chervonotkatska 67, 02660, Kiev, Ukraine
e-mail: info@bienta.net
www.bienta.net

CONTENTS……………………………………………….PAGE

1. STUDY RESPONSIBILITIES 2

2. STUDY OBJECTIVE 2

3. MATERIALS AND METHODS 2

3.1 REAGENTS AND CONSUMABLES 2

3.2 EQUIPMENT 2

3.3 STUDY DESIGN 3

3.4 SAMPLE PROCESSING 3

3.5 SAMPLES ANALYSIS 3

3.6 HPLC-MS/MS CONDITIONS 3

3.7 PREPARATION OF CALIBRATION STANDARDS 4

3.8 METHOD VALIDATION RESULTS 4

3.9 PHARMACOKINETIC METHOD ANALYSIS 5

4. RESULTS 6

5. ANALYSIS OF BRAIN AND LIVER SAMPLES……………...11

5. 1 SAMPLE PROCESSING……………………………….11

5.2 PREPARATION OF CALIBRATION STANDARDS…………..11

5.3 METHOD VALIDATION…………………………...11

5.4 CALIBRATION CURVES……………………………...12

5.5 COMPOUND CONCENTRATION IN BRAIN AND LIVER SAMPLES……………..14

1. Study Responsibilities

Study Director	Yuliia Holota, Ph.D.
In Vivo Scientists	Iryna Pishel, Ph.D.
	Alexander Tarasov, M.S.
	Maria Sokolenko, L.T.
Scientists	Volodymyr Iurchenko, Ph.D.
	Yulya Tokaryeva, M.S.
	Illia Petrov, M.S.

2. Study Objective

The purpose of this study was to determine the pharmacokinetic characteristics of compound **NG-537** in C57BL/6J mice following intravenous (IV) and peroral (PO) administration. Levels of **NG-537** were determined in blood plasma, brain and liver over time after a single dose.

3. Materials and Methods

3.1 Reagents and consumables

DMSO Chromasolv Plus, HPLC grade, ≥99.7% (Sigma-Aldrich, USA; Cat #34869)

Acetonitrile Chromasolv, gradient grade, for HPLC, ≥99.9% (Sigma-Aldrich, USA; Cat #34851)

Methanol Chromasolv Plus, for HPLC, ≥99.9% (Sigma-Aldrich, USA; Cat 34860)

Formic acid for mass spectrometry, ~98% (Fluka, USA; Cat #94318)

Physiological saline (“Yuria-Pharm”, Ukraine, S/# AA13249/1-1)

Amyl alcohol (UOS, Ukraine)

2,2,2-Tribromoethanol 97% (Sigma-Aldrich; Cat # T48402)

Compound **Thiamethoxam** was used as internal standard (**IS**).

Compound **NG-537** was supplied as dry powder. The batches of working formulations were prepared 20 min prior to the *in vivo* study. The vehicle was saline. To prepare the formulation, saline was added to the test compound. The mixture was vortexed for 1 min. The compound was fully dissolved in the vehicle.

3.2 Equipment

Gradient HPLC system (Shimadzu, Japan)

MS/MS detector API 3000 PE with TurboIonSpray Electrospray module (PE Sciex, Canada)

VWR Membrane Nitrogen Generators N2-04-L1466. nitrogen purity 99%+ (VWR, USA)

Water purification system Millipore Milli-Q Gradient A10 (Millipore, France)

Fixed Speed Vortex Mixer “IKA Lab Dancer” (IKA®-Werke GmbH & Co. KG, Germany; IP-40)

Centrifuge 4-15C (Qiagen) (Sigma, Germany)

Ultrasonic bath (Daihan, Korea; WUC-A03H)

3.3 Study design

Study design, animal selection, handling and treatment were all in accordance with the Enamine PK study protocols and the Institutional Animal Care and Use Guidelines. Animal treatment and plasma samples preparation were conducted by the Animal Laboratory personnel at Enamine/Bienta. Male C57BL/6J mice (9-11 weeks old, body weight from 26.1 g to 39.8 g and average body weight across all groups 31.9 g SD = 3.3 g) were used in this study. The animals were randomly assigned to the treatment groups before the pharmacokinetic study; all animals were fasted for 4 h before dosing. Seven time points (5, 15, 30, 60, 240, 480, and 1440 min) were set for this pharmacokinetic study. Each of the time point treatment group included 4 animals. There was also control group of one animal. Dosing was done according to the treatment schedule outlined in Table 1. Mice were injected IP with 2,2,2-tribromoethanol at the dose of 250 mg/kg prior to drawing the blood. Blood collection was performed from the left ventricle of the heart in tubes containing 10 µl of heparin solution (400 ui/ml). The brain and liver were perfused before sampling. The animal died due to exsanguination during perfusion under anesthesia. All samples were immediately processed, flash-frozen and stored at -70°C until subsequent analysis.

Table 1. Study design.

Number of Mice (male)	Compound ID	Formulation	Delivery Route	Target Dose Level (mg/kg)	Target Dose Concentration (mg/ml)	Target Dose Volume (ml/kg)
28	**NG-537_HCl**	Saline	IV	10	2	5
1	Vehicle dosed		IV	0	0	5
28	**NG-537_HCl**	Saline	PO	50	10	5
1	Vehicle dosed		PO	0	0	5

3.4 Samples processing

Plasma samples (40 µl) were mixed with 200 µl of **IS** solution. After mixing by pipetting and centrifuging for 4 min at 6,000 rpm, 2 µl of each supernatant was injected into LC-MS/MS system.

Solution of compound **Thiamethoxam** (400 ng/ml in water-methanol mixture 1:9, v/v) was used as internal standard (**IS**) for quantification of **NG-537** in plasma samples.

3.5 Samples analysis

Analyses of samples were conducted by the Bioanalytical Laboratory personnel at Enamine/Bienta. Concentrations of **NG-537** were determined using high performance liquid chromatography/tandem mass spectrometry (HPLC-MS/MS). Shimadzu HPLC system consisted 2 isocratic pumps LC-10ADvp, an autosampler SIL-20AC, a sub-controller FCV-14AH and a degasser DGU-14A. Mass spectrometric analysis was performed using API 3000 (triple-quadrupole) instrument from AB Sciex (Canada) with an electro-spray (ESI) interface. The data acquisition and system control was performed using Analyst 1.5.2 software (AB Sciex, Canada).

3.6 HPLC-MS/MS Conditions

Chromatographic Conditions:

Column: Zorbax Eclipse Plus C18 (2.1 x 50 mm, 3.5 µm)

Mobile phase A: Acetonitrile : Water : Formic acid = 50 : 950 : 1

Mobile phase B: Acetonitrile : Formic acid = 100 : 0.1

Linear gradient: 0 min 0% B, 1.10 min 100% B, 1.20 min 100% B, 1.21 min 0% B, 3.2 min stop

Elution rate: 400 µL/min. A divert valve directed the flow to the detector from 1.3 to 1.8 min

Column temperature: 30°C

MS/MS Detection:

Scan type: Positive MRM, Ion source: Turbo spray, Ionization mode: ESI

Nebulize gas: 15 L/min, Curtain gas: 8 L/min, Collision gas: 4 L/min

Ionspray voltage: 5000 V, Temperature: 400°C

Table 2. Other MS parameters

Compound ID	Parent, m/z	Daughter, m/z	Time, ms	DP, V	FP, V	EP, V	CE, V	CXP, V
**NG-537**	371.035	222.3	70	21	80	11	45	18
Thiamethoxam	292.100	211.3	70	41	260	11	21	38

3.7 Preparation of calibration standards

Calibration standards for quantification of NG-537 in plasma samples. Compound **NG-537** (as hydrochloride) was dissolved in DMSO, and resulting solution with concentration of 2 mg/ml was used for calibration standards preparation (stock solution). The stock solution was consecutively diluted with **IS** to get a series of calibration solutions with final concentrations of 40 000, 10 000, 5 000, 2 000, 1 000, 500, 250, 100, 50, 20, 10, 5 and 2 ng/ml. Calibration curve was constructed using blank mouse plasma samples. To obtain calibration standards, blank plasma samples (40 µl) were mixed with 200 µl of corresponding calibration solution. After mixing by pipetting and centrifugation for 4 min at 6000 rpm, 2 µl of each supernatant was injected into LC-MS/MS system.

3.8. Method Validation Results

Specificity: Mice blank plasma had no interference with compound **NG-537** and IS, as shown in Figure 1.

Figure 1. Chromatographic Graphs

Mice blank plasma

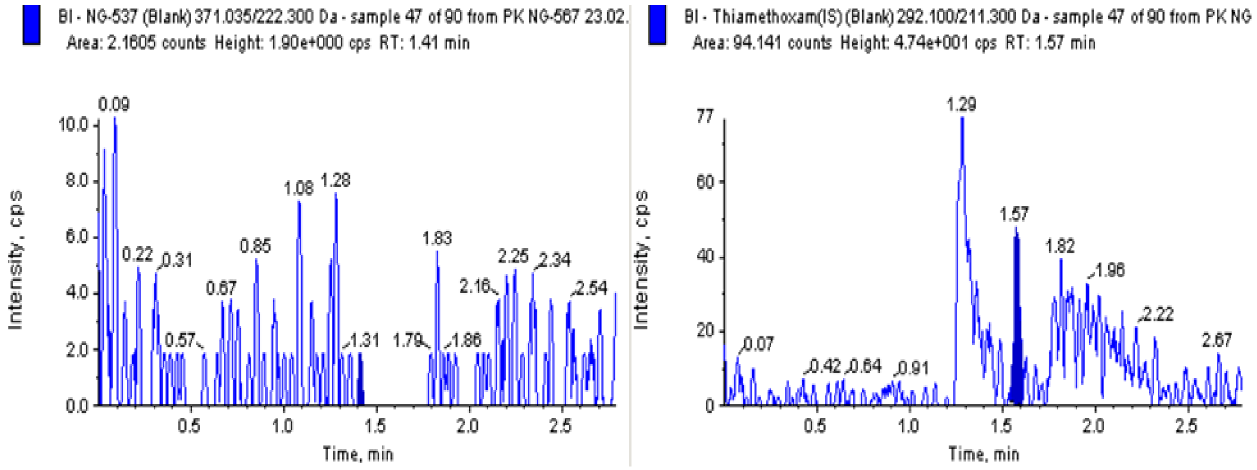

Blank plasma spiked with compound **NG-537** (10 ng/ml) and IS

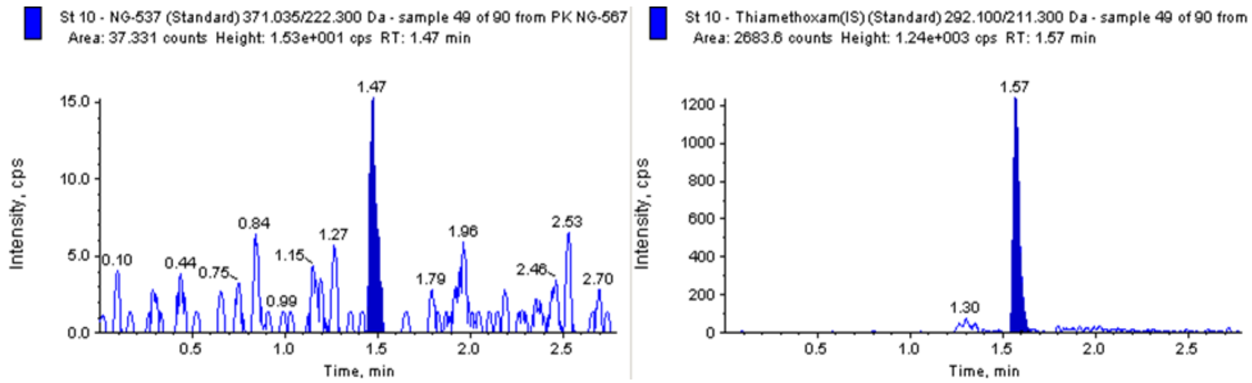

Calibration curve

The regression analysis of compound **NG-537** was performed by plotting the peak area ratio (y) against the compound concentration in calibration standards (x, ng/ml). The validity of the calibration curves (relationship between peak area ratio and compound concentration) is proved by the correlation coefficients (R) calculated for the quadratic regression (Figure 2).

Figure 2A. Calibration curve for the quantification of NG-537 in plasma samples (weight=1/x)

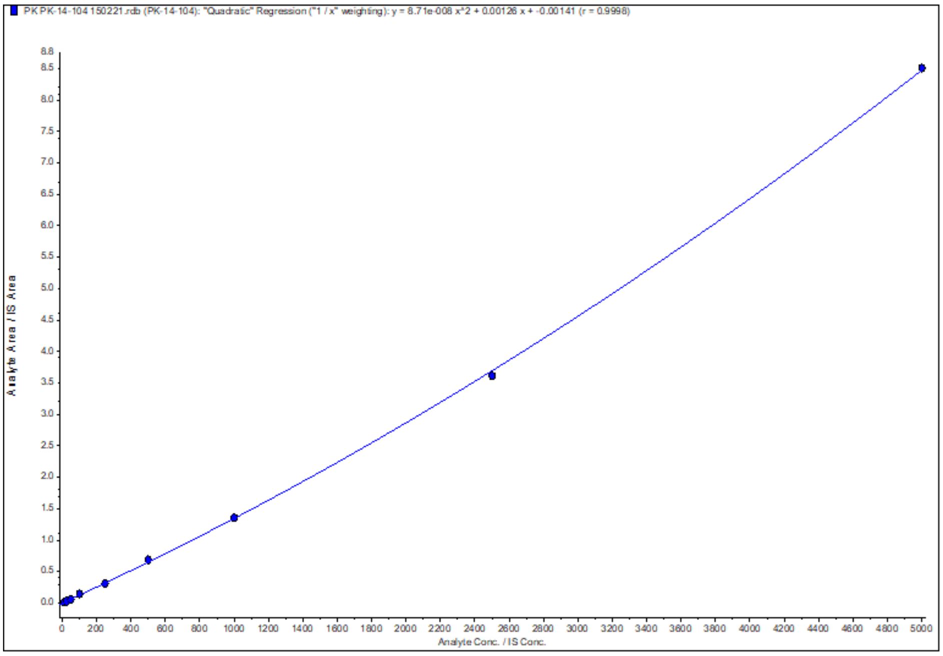

Correlation coefficient = 0.9997

3.9. Pharmacokinetic Method Analysis

The concentrations of **NG-537** in plasma samples below the lower limit of quantitation (LLOQ = 10 ng/ml) were designated as zero. The pharmacokinetic data analysis was performed using noncompartmental, bolus injection or extravascular input analysis models in WinNonlin 5.2 (PharSight). Data below LLOQ were presented as missing to improve validity of T½ calculations.

The oral bioavailability was calculated as:

$$F\ (\%) = \frac{Dose_{IV} \times AUC_{(0-\infty)PO}}{Dose_{PO} \times AUC_{(0-\infty)IV}} \times 100\%$$

4. Results

The individual and average **NG-537** concentrations data in plasma for perorally- and intravenously-dosed groups are listed in Tables 3, 5 and graphically presented in Figures 3, 4. Selected noncompartmental pharmacokinetic parameters for plasma are listed in Tables 4 and 6. Please note that elimination curve approximation is performed automatically by WinNonlin PK program using a standardized procedure. Resulting calculated elimination rate K_{el} and the parameters derived from it, such as terminal elimination half-life $T_{1/2}$, may not always properly reflect the observed pharmacokinetic processes. In some cases, alternative analyses of the observed concentration-time dependency may be required. Any comparison of resulting PK parameter values and conclusions about compound and/or formulation properties must be based on full PK curve data and take into account specific experimental designs.

Table 3. Plasma concentrations of NG-537 in male C57BL/6J mice following intravenous (10 mg/kg) administration

Sample collection time point, min	Plasma concentration (ng/ml)						
	Mouse A	Mouse B	Mouse C	Mouse D	Mean	SD	SE
0	BQL				**BQL**	ND	ND
5	2250	1763	2817	1209	**2010**	686	343
15	863	1001	1370	836	**1018**	246	123
30	646	436	389	255	**432**	162	81
60	356	78	220	109	**191**	126	63
240	BQL	BQL	BQL	BQL	**BQL**	ND	ND
480	BQL	BQL	BQL	BQL	**BQL**	ND	ND
1440	BQL	BQL	BQL	BQL	**BQL**	ND	ND

BQL - Below the lower limit of quantitation (LLOQ)
ND - Not determined

Table 4. Selected pharmacokinetic parameters for NG-537 in male C57BL/6J mice following intravenous (10 mg/kg) administration

Animal	Administration	Dose, mg/kg	Pharmacokinetic Parameters									
			Tmax, min	C_0, ng/ml	$AUC_{0\rightarrow t=60min}$ (AUClast) ng*min/ml	$AUC_{0\rightarrow\infty}$ (AUCINF_obs), ng*min/ml	$T_{1/2}$ (HL_Lambda_z), min	K_{el} (Lambda_z), min^{-1}	MRT (MRTlast), min	MRT (MRTinf), min	V_d (Vz_obs), ml/kg	CL (Cl_obs), ml/min/kg
Mice	IV	10	-	2824.53	47400	52000	16.6	0.0417	15.4	21.4	5000	190

Figure 3. Plasma concentration-time curve of NG-537 in male C57BL/6J mice following intravenous (10 mg/kg) administration (n=4)

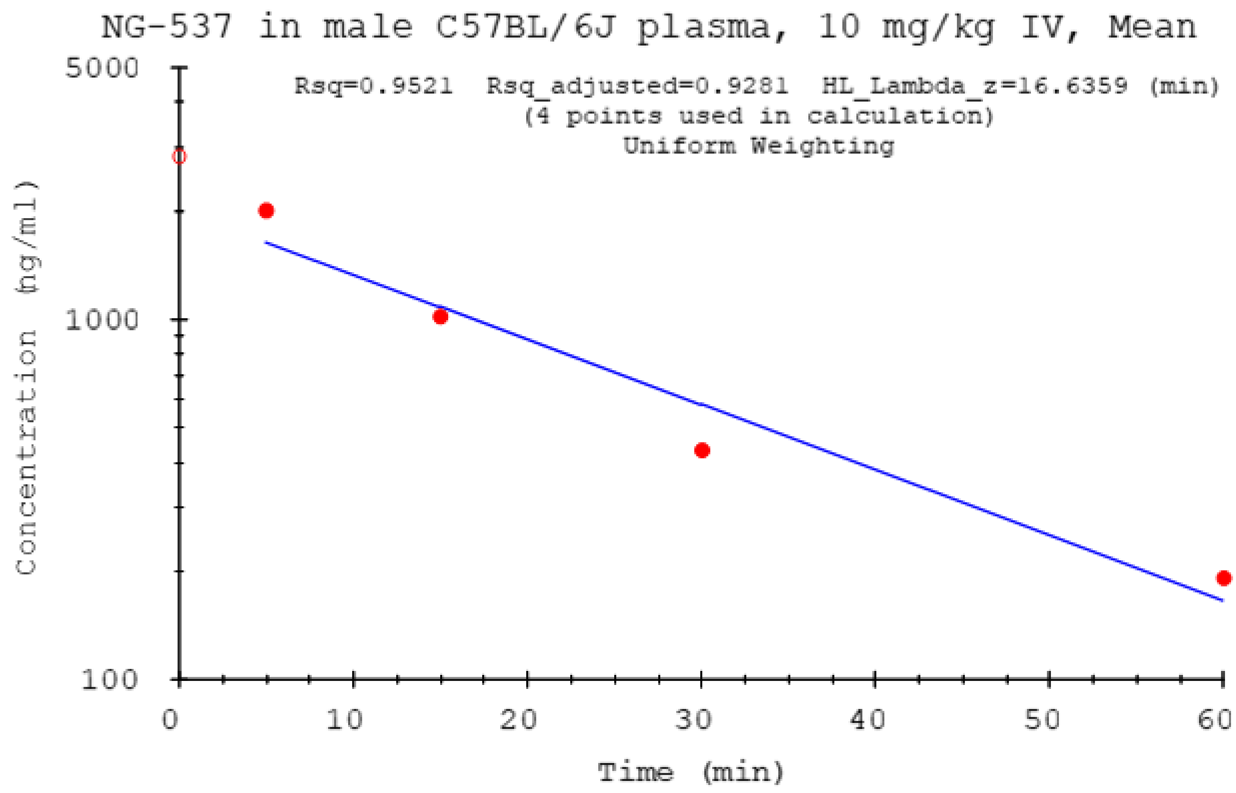

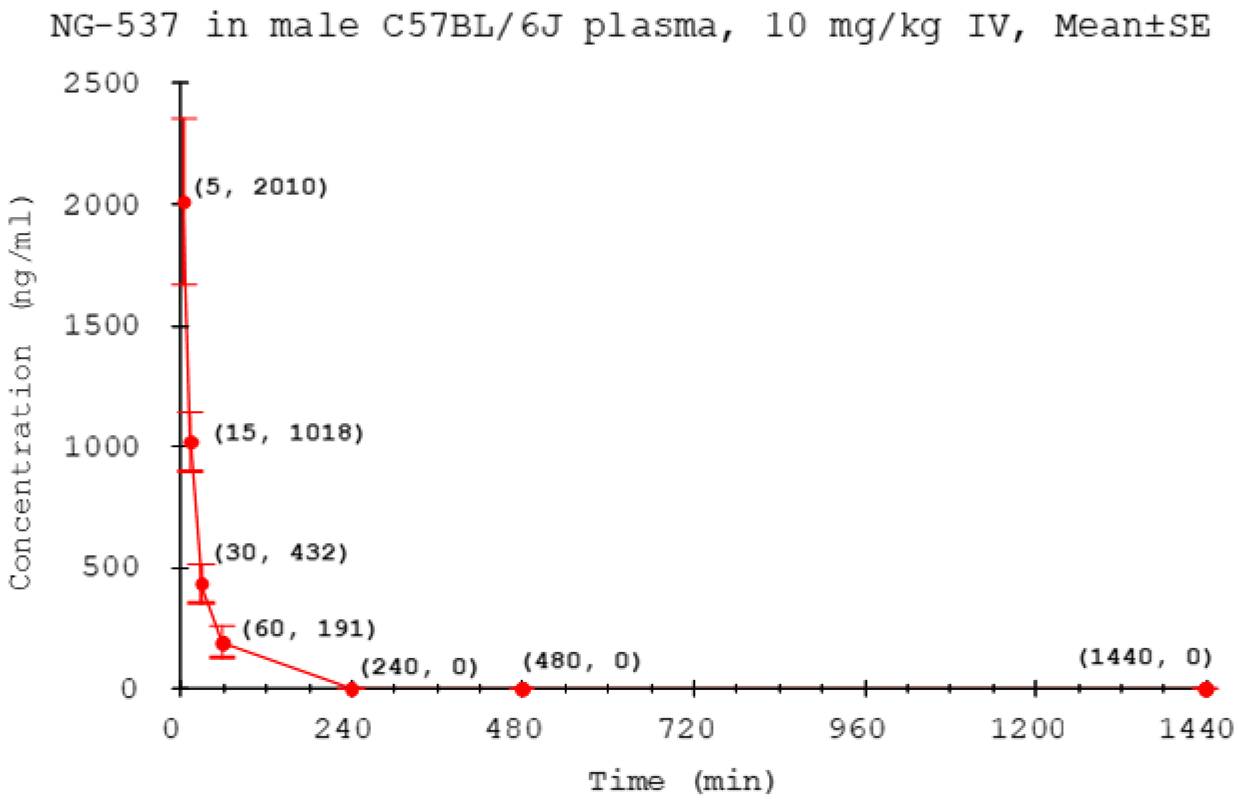

Table 5. Plasma concentrations of NG-537 in male C57BL/6J mice following oral (50 mg/kg) administration

Sample collection time point, min	Plasma concentration (ng/ml)						
	Mouse A	Mouse B	Mouse C	Mouse D	Mean	SD	SE
0	BQL				**BQL**	ND	ND
5	78	260	401	1064	**451**	430	215
15	1715	1721	2715	1977	**2032**	471	236
30	1727	1258	2296	1618	**1725**	430	215
60	686	889	1834	1120	**1132**	500	250
240	170	203	62	170	**151**	62	31
480	BQL	15*	BQL	BQL	**BQL**	ND	ND
1440	BQL	BQL	BQL	BQL	**BQL**	ND	ND

BQL - Below the lower limit of quantitation (LLOQ)
ND - Not determined
*Grubbs' outlier test: Significant outlier. $P < 0.05$

Table 6. Selected pharmacokinetic parameters (plasma) for NG-537 in male C57BL/6J mice following oral (50 mg/kg) administration

Animal	Administration	Dose, mg/kg	Pharmacokinetic Parameters							
			Tmax, min	Cmax, ng/ml	$AUC_{0\to t=240min}$ (AUClast) ng*min/ml	$AUC_{0\to\infty}$ (AUCINF_obs), ng*min/ml	$T_{1/2}$ (HL_Lambda_z), min	K_{el} (Lambda_z), min^{-1}	MRT (MRTlast), min	MRT (MRTinf), min
Mice	PO	50	15.0	2030	200000	213000	60.2	0.0115	59.8	76.2

Figure 4. Plasma concentration-time curve of NG-537 in male C57BL/6J mice following oral (50 mg/kg) administration (n=4)

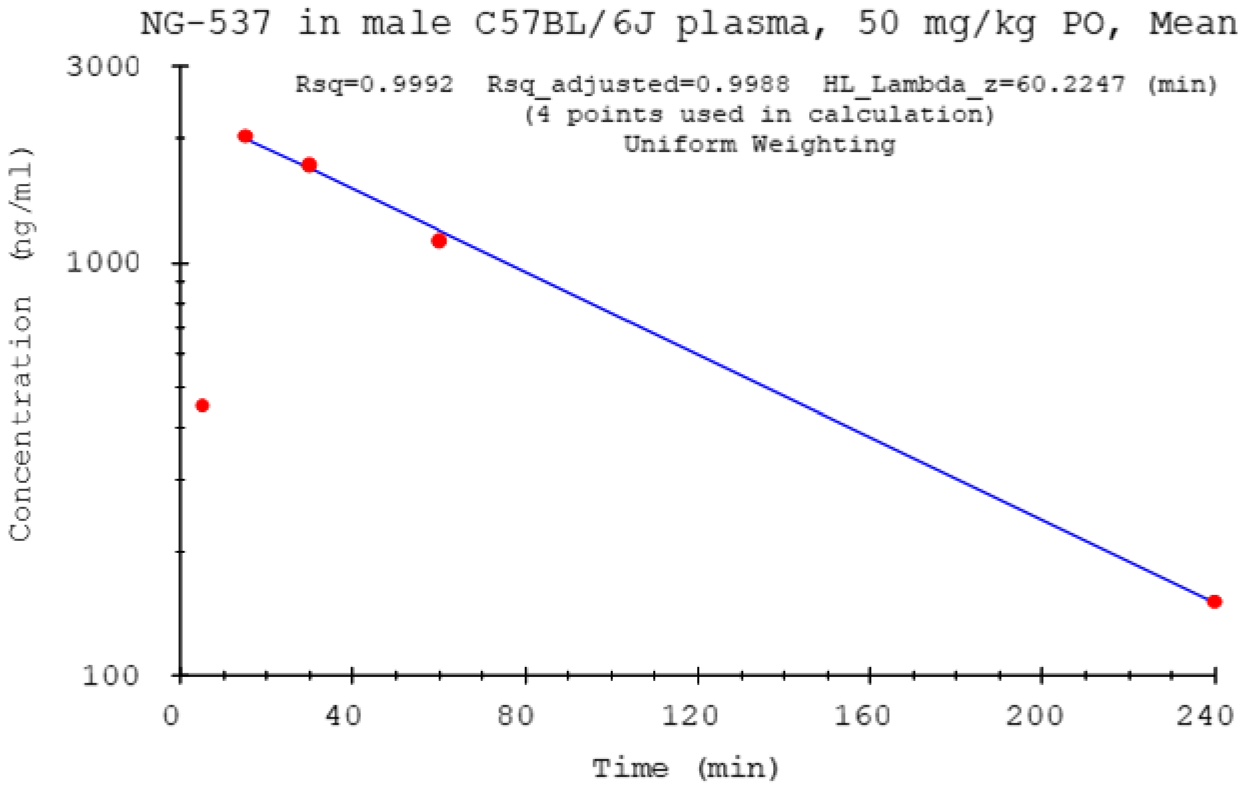

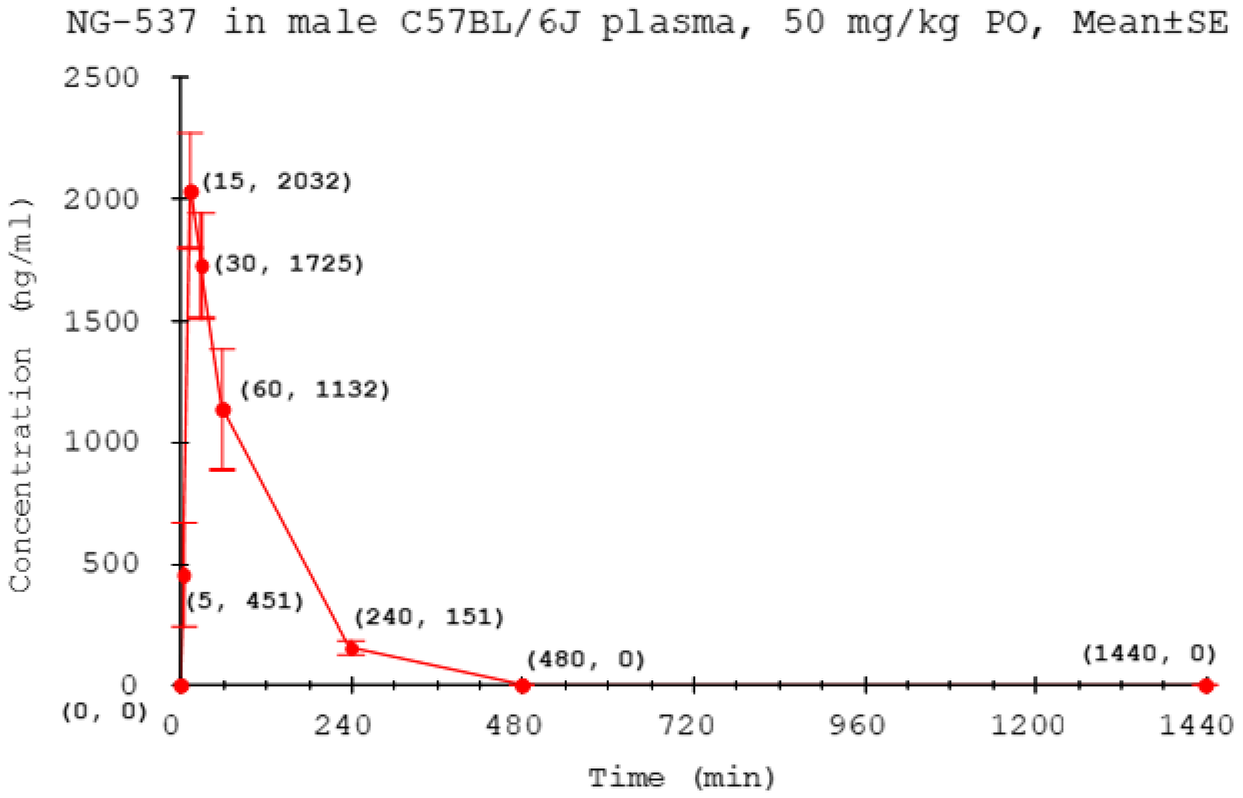

Summary: The pharmacokinetic parameters for **NG-537** in blood plasma are shown in the summary table below (Table 7). The calculated oral bioavailability for compound **NG-537** is **82%**. Figure 5 summarizes the results of PK study for compound **NG-537** in mice. No obvious adverse effects were observed during this PK study.

Table 15. Selected pharmacokinetic parameters for NG-537 in male C57BL/6J mice

Sample	Administration	Dose, mg/kg	Pharmacokinetic Parameters							
			Tmax, min	Cmax, ng/ml(g)	$AUC_{0\to t\,min}$ (AUClast) ng*min/ml(g)	$AUC_{0\to\infty}$ (AUCINF_obs) ng*min/ml	$T_{1/2}$ (HL_Lambda_z), min	K_{el} (Lambda_z), min^{-1}	V_d (Vz_obs) ml/kg	Bioavailability, %
Plasma	IV	10	-	2010	47400	52000	16.6	0.0417	5000	**82**
	PO	50	15.0	2030	200000	213000	60.2	0.0115	ND	

Note: in Table 7, C_{max} is indicated for IV route, in contrast to C_0 from Table 4

Figure 5. Concentration-time curves for NG-537 in male C57BL/6J mice following IV and PO administration

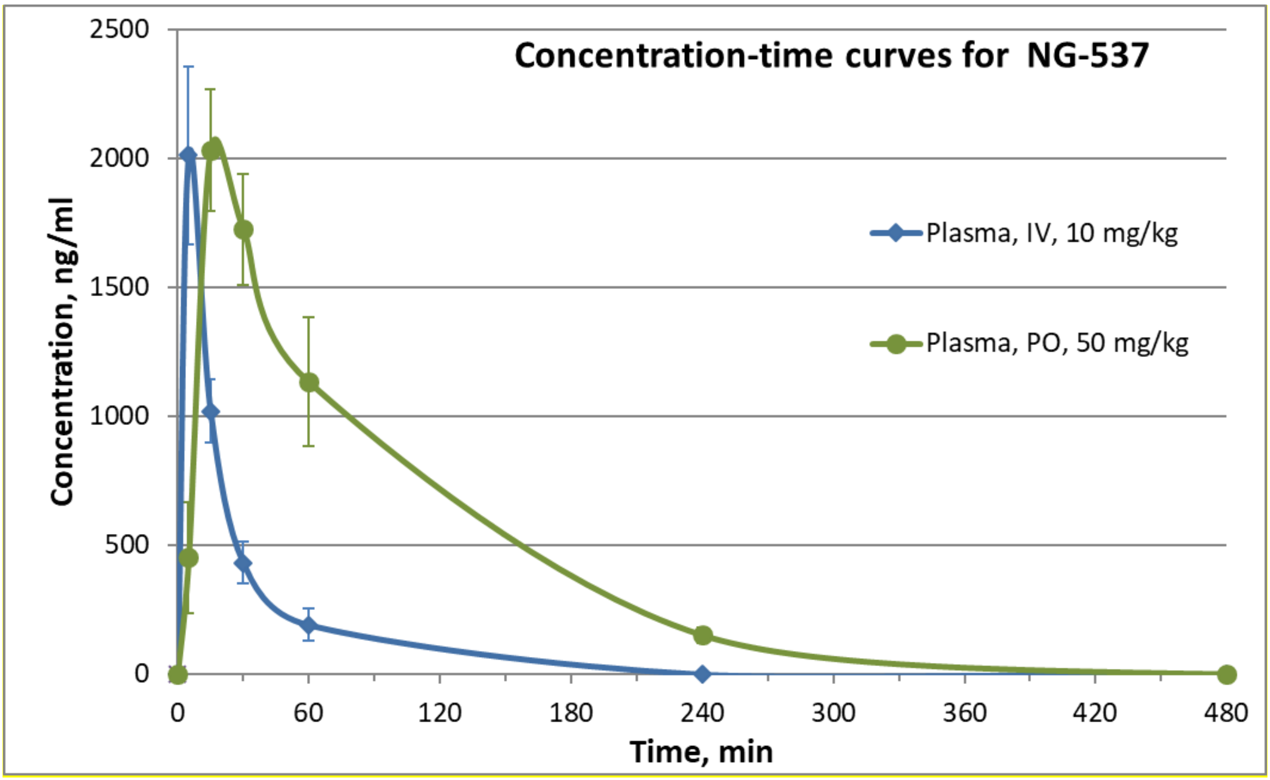

5. Analysis of brain and liver samples

5.1 Samples processing

Liver samples (weight 200 mg ± 1 mg) were homogenized in 800 µl of **IS1000(90)** using zirconium oxide beads (115 mg ± 5 mg) in The Bullet Blender® homogenizer for 30 seconds at speed 8. After this, the samples were centrifuged for 4 min at 14,000 rpm, and 2 µl of each supernatant was injected into LC-MS/MS system. Solution of compound **Thiamethoxam** (1000 ng/ml in water-methanol mixture 1:9, v/v) was used as internal standard (**IS1000(90)**) for quantification of **NG-537** in liver samples.

Brain samples (weight 200 mg ± 1 mg) were homogenized in 800 µl of **IS1000(80)** using zirconium oxide beads (115 mg ± 5 mg) in The Bullet Blender® homogenizer for 30 seconds at speed 8. After this, the samples were centrifuged for 4 min at 14,000 rpm, and 2 µl of each supernatant was injected into LC-MS/MS system. Solution of compound **Thiamethoxam** (1000 ng/ml in water-methanol mixture, 1:4, v/v) was used as internal standard (**IS1000(80)**) for quantification of NG-537 in brain samples.

5.2 Preparation of Calibration Standards

Calibration standards for quantification of NG-537 in liver samples. The compound **NG-537** (as hydrochloride) was dissolved in DMSO, and resulting solution with concentration of 2 mg/ml was used for calibration standards preparation (stock solution). The stock solution was consecutively diluted with **IS1000(90)** to get a series of calibration solutions with final concentrations of 20 000, 10 000, 5 000, 2 000, 1 000, 500, 200, 100 and 50 ng/ml. Calibration curve was constructed using blank mouse liver samples. To obtain calibration standards, blank liver samples (weight 100 mg ± 1 mg) were homogenized in 400 µl of corresponding calibration solution using zirconium oxide beads (115 mg ± 5 mg) in The Bullet Blender® homogenizer for 30 seconds at speed 8. After this, the samples were centrifuged for 4 min at 14 000 rpm, and 2 µl of each supernatant was injected into LC-MS/MS system.

Calibration standards for quantification of NG-537 in brain samples. The stock solution of compound **NG-537** was consecutively diluted with **IS1000(80)** to get a series of calibration solutions with final concentrations of 2 000, 1 000, 500, 200, 100, 50, 20, 10 and 5 ng/ml. Calibration curve was constructed using blank mouse brain samples. To obtain calibration standards, blank brain samples (weight 200 mg ± 1 mg) were homogenized in 800 µl of corresponding calibration solution using zirconium oxide beads (115 mg ± 5 mg) in The Bullet Blender® homogenizer for 30 seconds at speed 8. After this, the samples were centrifuged for 4 min at 14 000 rpm, and 2 µl of each supernatant was injected into LC-MS/MS system.

5.3 Method Validation

Specificity: Mice blank liver had no interference with compound **NG-537** and **IS**, as shown in Figure 6.

Figure 6. Chromatographic Graphs

Mouse blank liver

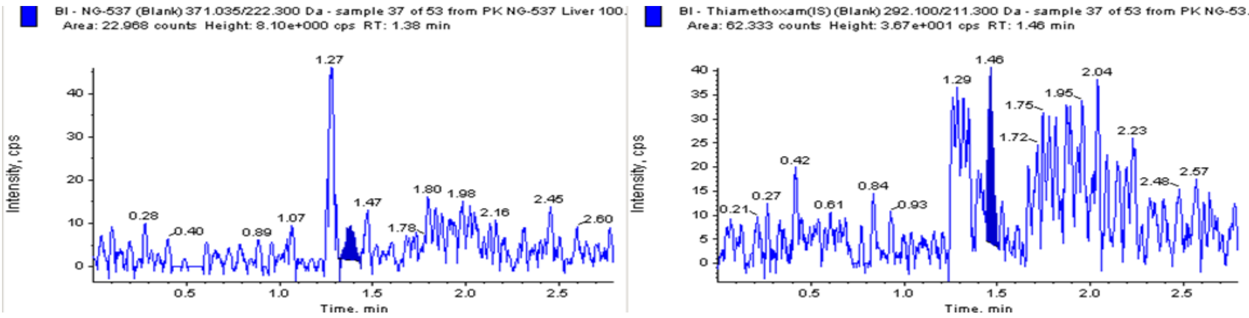

Blank liver spiked with compound **NG-537** (200 ng/g) and IS

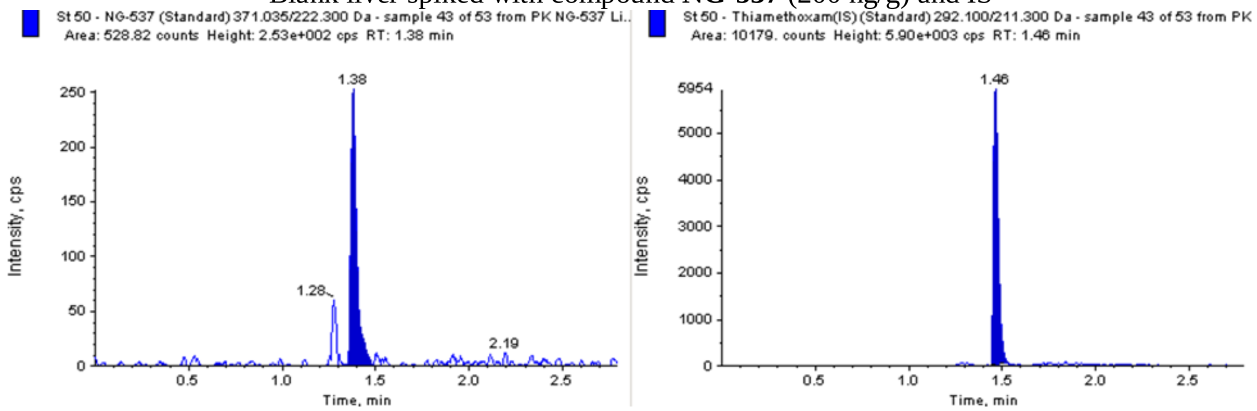

Mouse blank brain

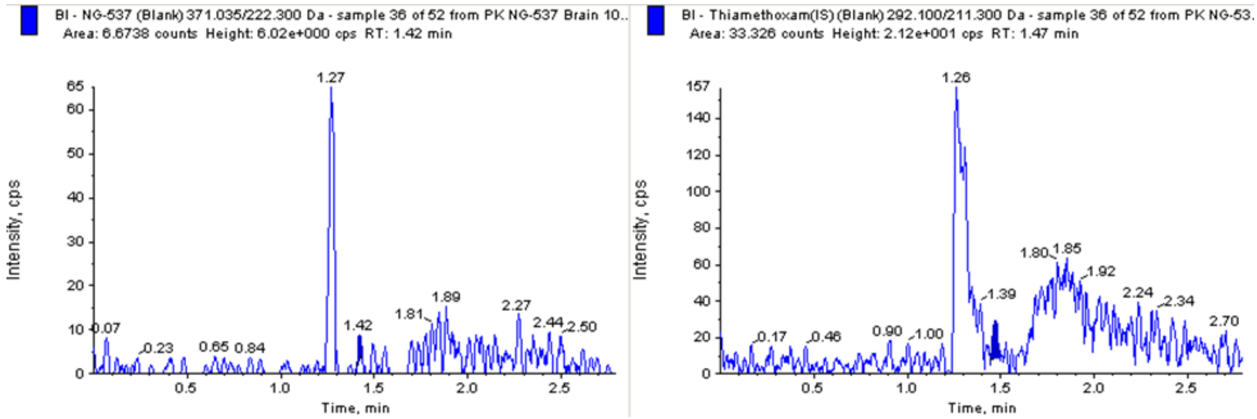

Blank brain spiked with compound **NG-537** (20 ng/g) and IS

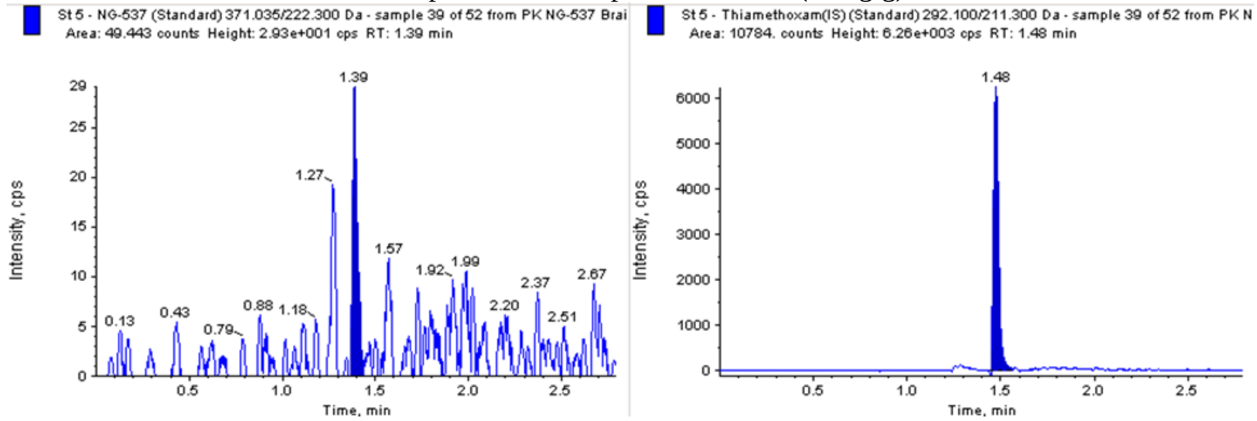

5.4 Calibration curves

The regression analysis of compound **NG-537** was performed by plotting the peak area ratio (y) against the compound concentration in calibration standards (x, ng/ml). The validity of the calibration curve (relationship between peak area ratio and compound concentration) is proved by the correlation coefficients (R) calculated for the quadratic regressions (Figures 7A-B).

Figure 7A. Calibration curve for the quantification of NG-537 in the liver samples (weight=1/x)

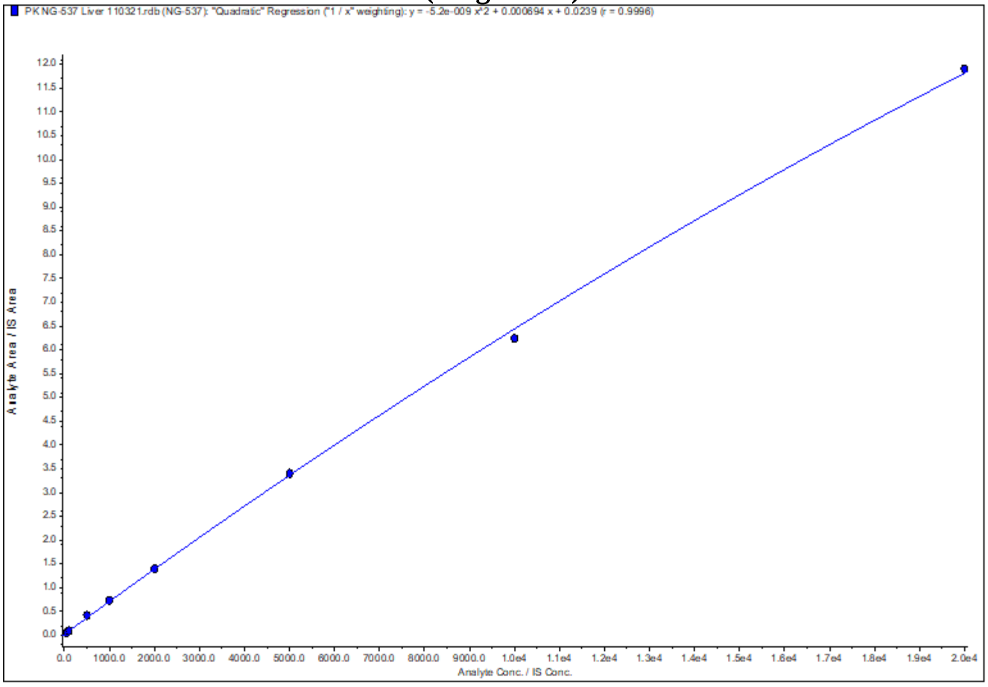

Correlation coefficient = 0.9996

Figure 7B. Calibration curve for the quantification of NG-537 in the brain samples (weight=1/x)

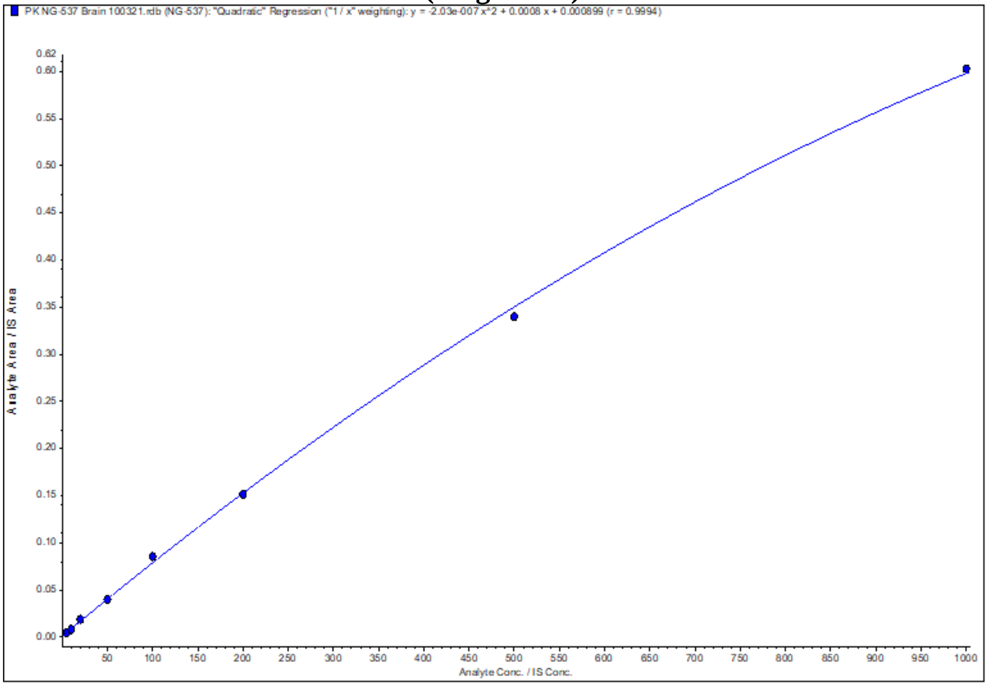

Correlation coefficient = 0.9994

The concentrations of the test compound below the lower limits of quantitation (LLOQ = 200 ng/g for liver and 20 ng/g for brain samples) were designated as zero.

5.5 COMPOUND CONCENTRATION IN BRAIN AND LIVER SAMPLES

The **NG-537** concentrations data in liver and brain samples selected by the Customer are listed in Tables 16, 18, 19 and graphically presented in Figures 8 and 9. Selected noncompartmental pharmacokinetic parameters are listed in Tables 18 and 21.

Table 16. Liver concentrations of NG-537 in male C57BL/6J mice following intravenous (10 mg/kg) administration

Sample collection time point, min	Liver concentration (ng/g)						
	Mouse A	Mouse B	Mouse C	Mouse D	Mean	SD	SE
0	BQL				**BQL**	ND	ND
15	11976	17352	12944	10288	**13140**	3015	1507

BQL - Below the lower limit of quantitation (LLOQ)
ND - Not determined

Table 17. Liver concentrations of NG-537 in male C57BL/6J mice following oral (50 mg/kg) administration

Sample collection time point, min	Liver concentration (ng/g)						
	Mouse A	Mouse B	Mouse C	Mouse D	Mean	SD	SE
0	BQL				**BQL**	ND	ND
15	64360	46240	98240	70920	**67690**	17758	8879
60	14584	20972	45720	30724	**28000**	13550	6775
240	5328	4248	1812	4200	**3897**	1484	742

BQL - Below the lower limit of quantitation (LLOQ)
ND - Not determined

Table 18. Selected pharmacokinetic parameters (liver) for NG-537 in male C57BL/6J mice following oral (50 mg/kg) administration

Animal	Administration	Dose, mg/kg	Pharmacokinetic Parameters							
			Tmax, min	Cmax, ng/g	$AUC_{0\to t=240min}$ (AUClast) ng*min/g	$AUC_{0\to\infty}$ (AUCINF_obs), ng*min/g	$T_{1/2}$ (HL_Lambda_z), min	K_{el} (Lambda_z), min^{-1}	MRT (MRTlast), min	MRT (MRTinf), min
Mice	PO	50	15.0	67700	5530000	5850000	56.8	0.0122	54.9	69.5

Figure 8. Liver concentration-time curve of NG-537 in male C57BL/6J mice following oral (50 mg/kg) administration (n=4)

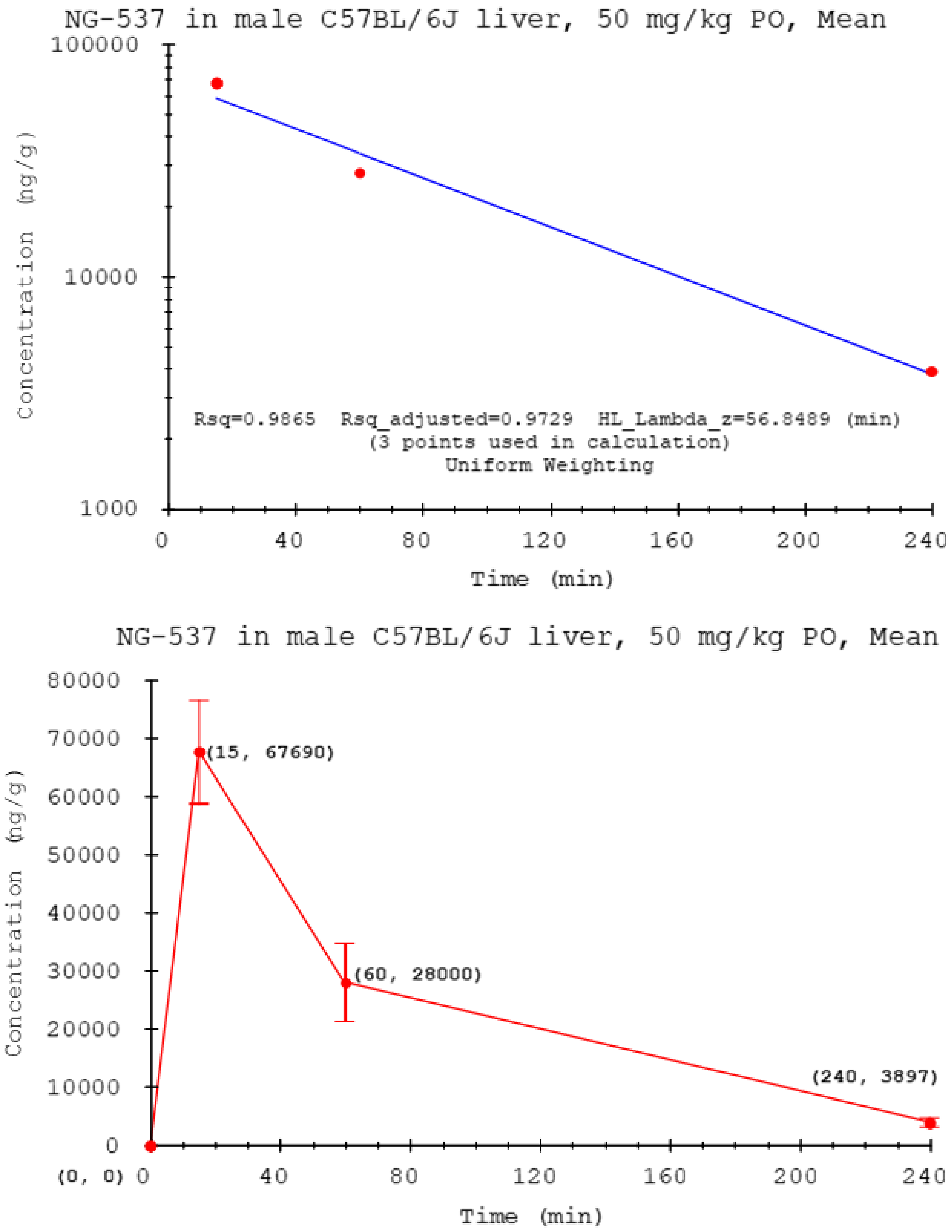

Table 19. Brain concentrations of NG-537 in male C57BL/6J mice following intravenous (10 mg/kg) administration

Sample collection time point, min	Brain concentration (ng/g)						
	Mouse A	Mouse B	Mouse C	Mouse D	Mean	SD	SE
0	BQL				**BQL**	ND	ND
15	2018	982	494	800	**1074**	661	331

BQL - Below the lower limit of quantitation (LLOQ)
ND - Not determined

Table 20. Brain concentrations of NG-537 in male C57BL/6J mice following oral (50 mg/kg) administration

Sample collection time point, min	Brain concentration (ng/g)						
	Mouse A	Mouse B	Mouse C	Mouse D	Mean	SD	SE
0	BQL				**BQL**	ND	ND
15	162	161	227	139	**172**	38	19
60	148	158	264	241	**203**	58	29
240	37	49	BQL	29	**28**	21	10

BQL - Below the lower limit of quantitation (LLOQ)
ND - Not determined

Table 21. Selected pharmacokinetic parameters (brain) for NG-537 in male C57BL/6J mice following oral (50 mg/kg) administration

Animal	Administration	Dose, mg/kg	Pharmacokinetic Parameters							
			Tmax, min	Cmax, ng/g	$AUC_{0\rightarrow t=240min}$ (AUClast) ng*min/g	$AUC_{0\rightarrow\infty}$ (AUCINF_obs), ng*min/g	$T_{1/2}$ (HL_Lambda_z), min	K_{el} (Lambda_z), min^{-1}	MRT (MRTlast), min	MRT (MRTinf), min
Mice	PO	50	60.0	203	30500	33100	63.5	0.0109	67.5	88.2

Figure 9. Brain concentration-time curve of NG-537 in male C57BL/6J mice following oral (50 mg/kg) administration (n=4)

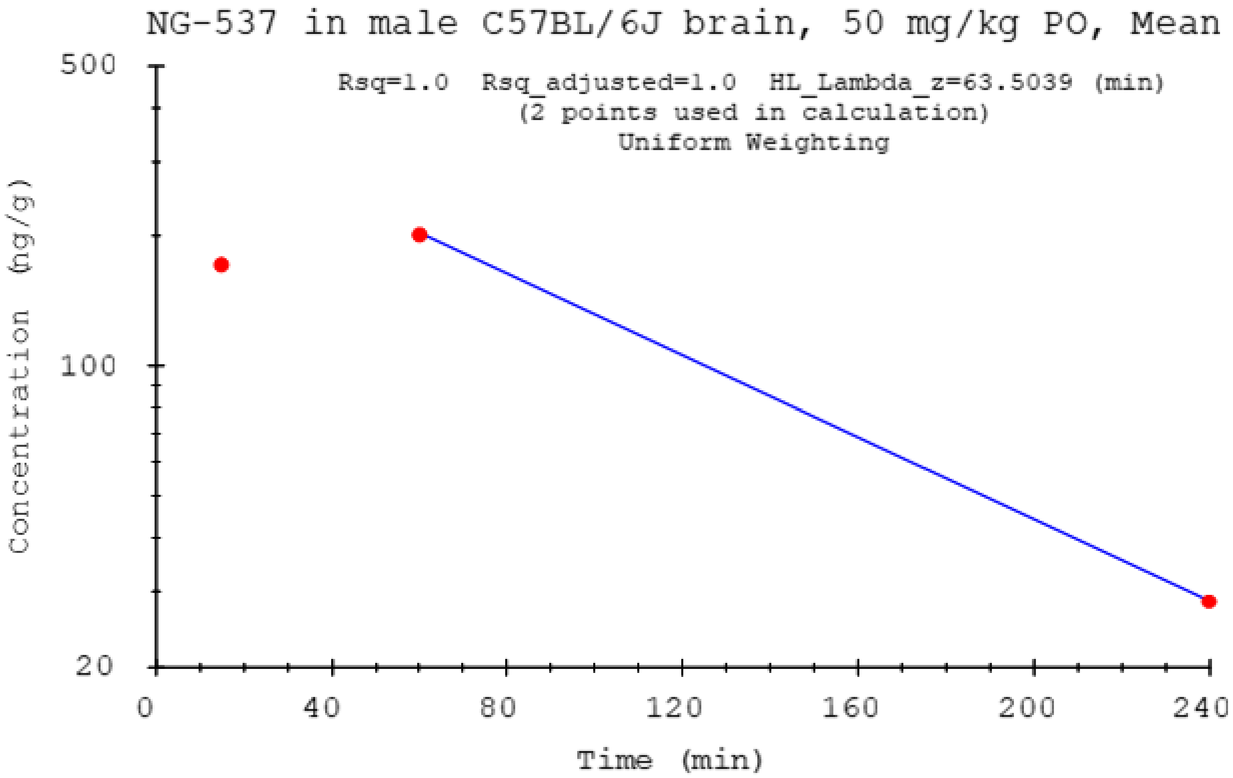

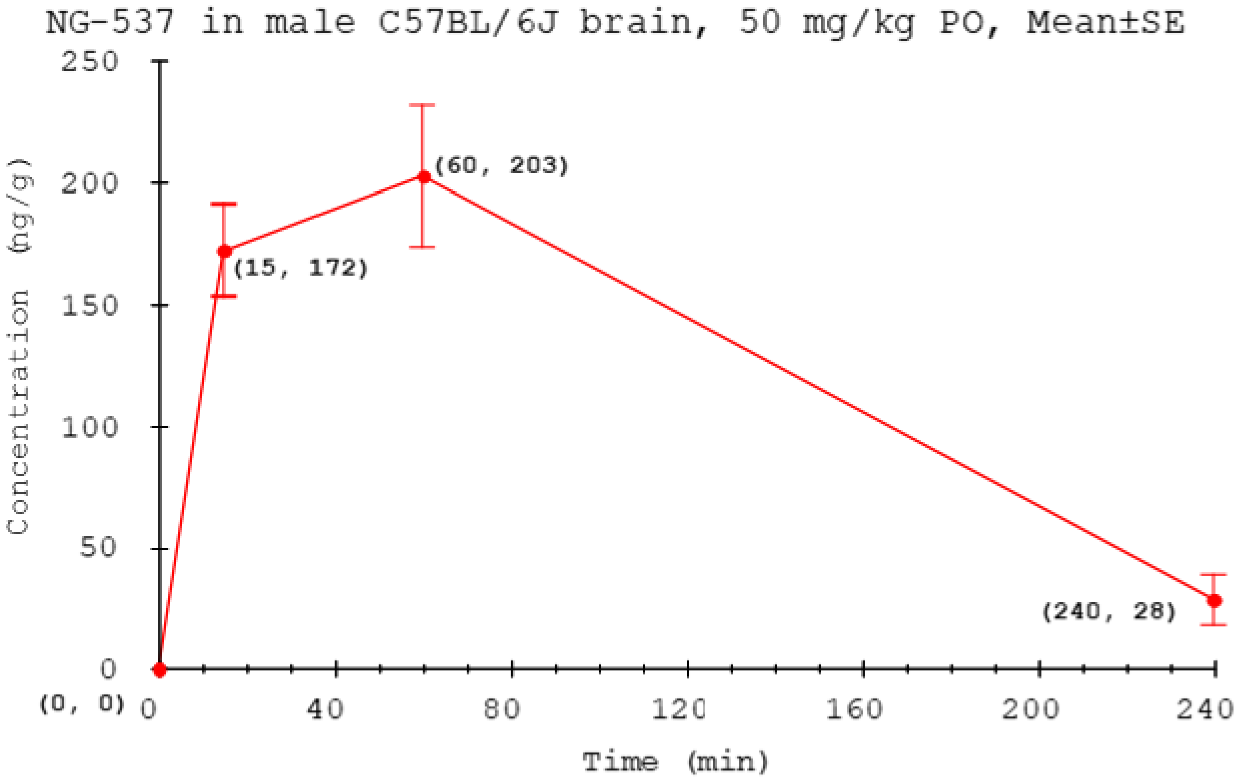

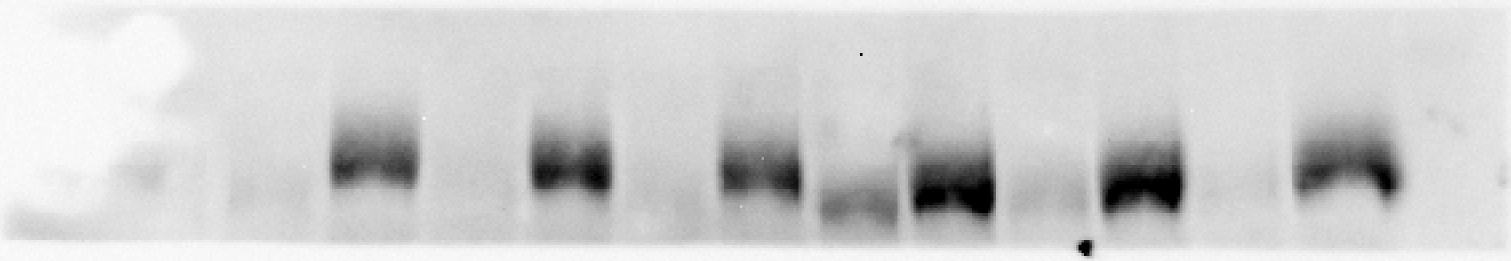

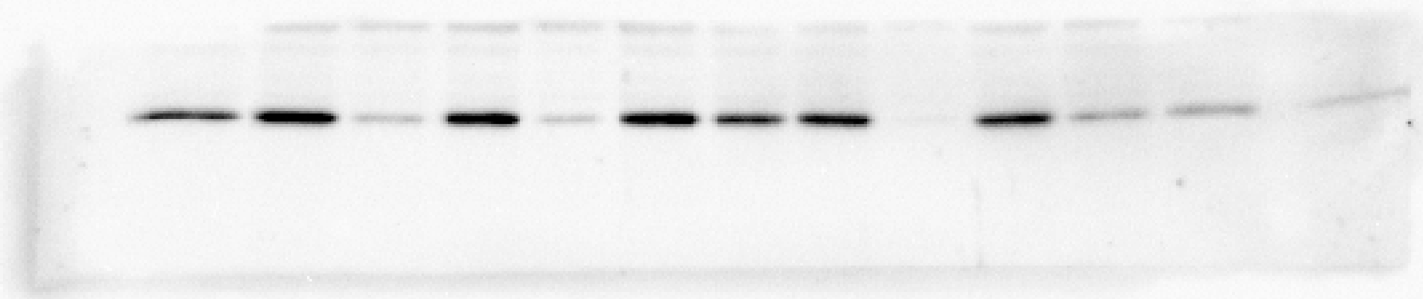

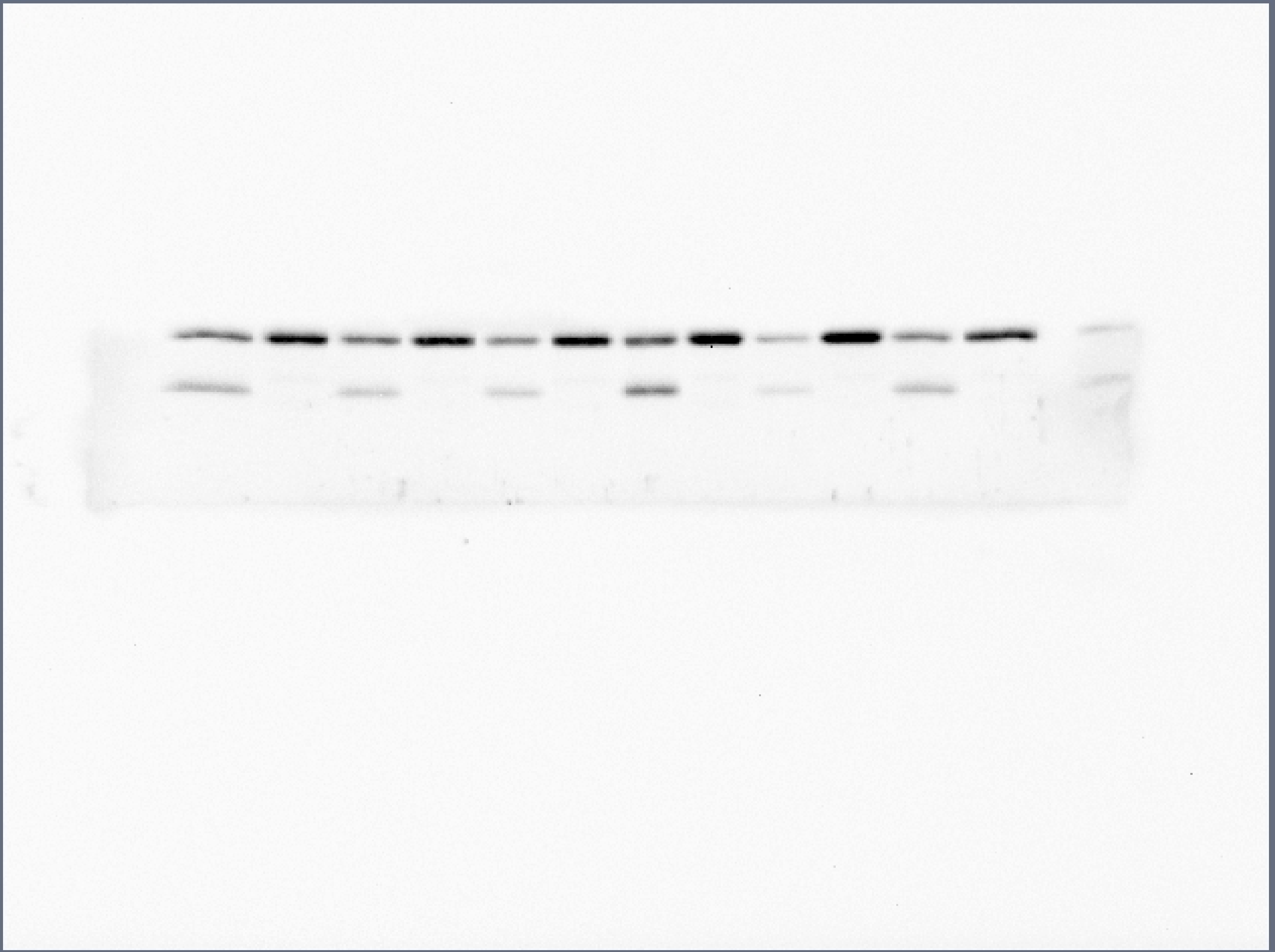

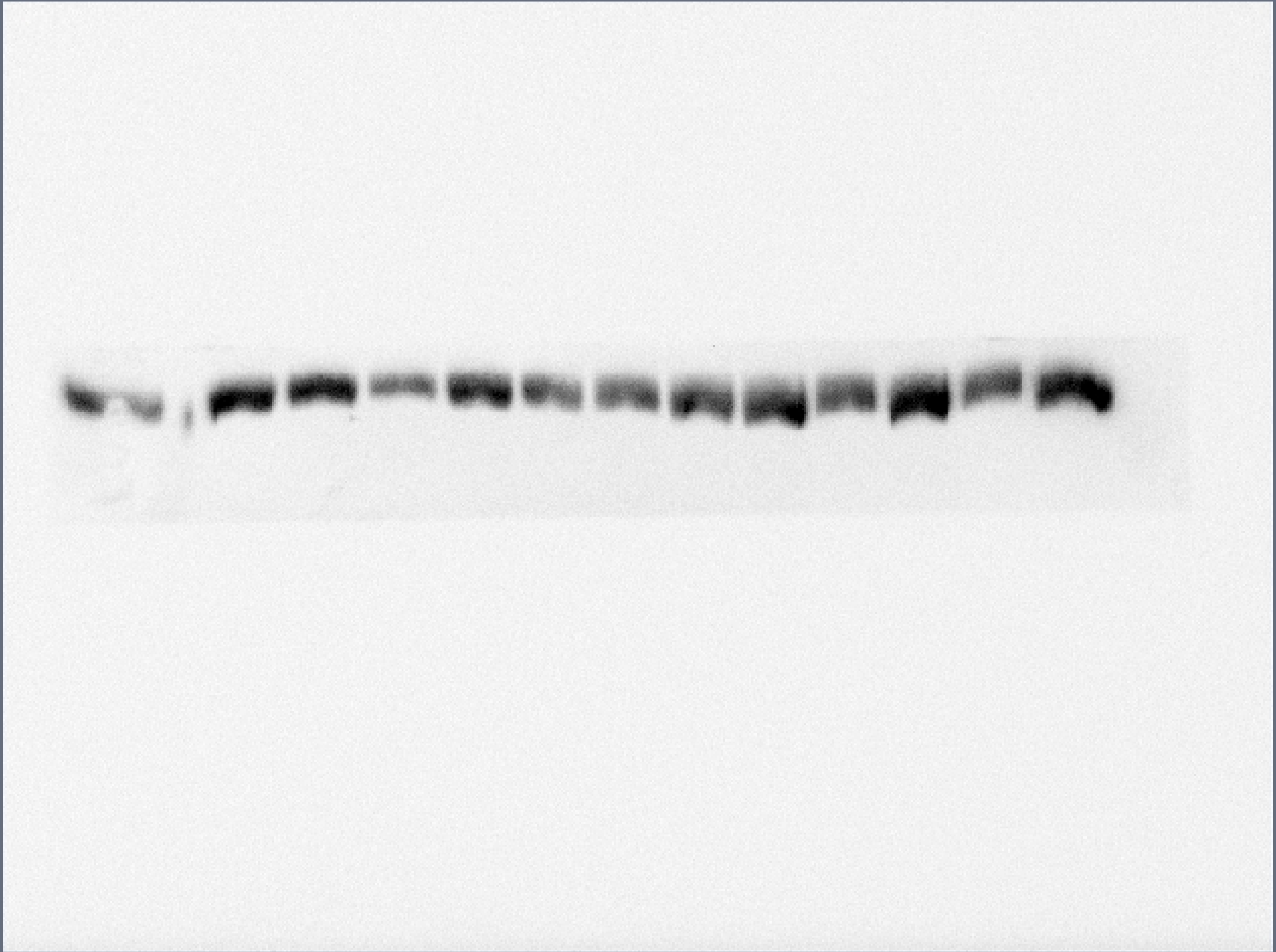

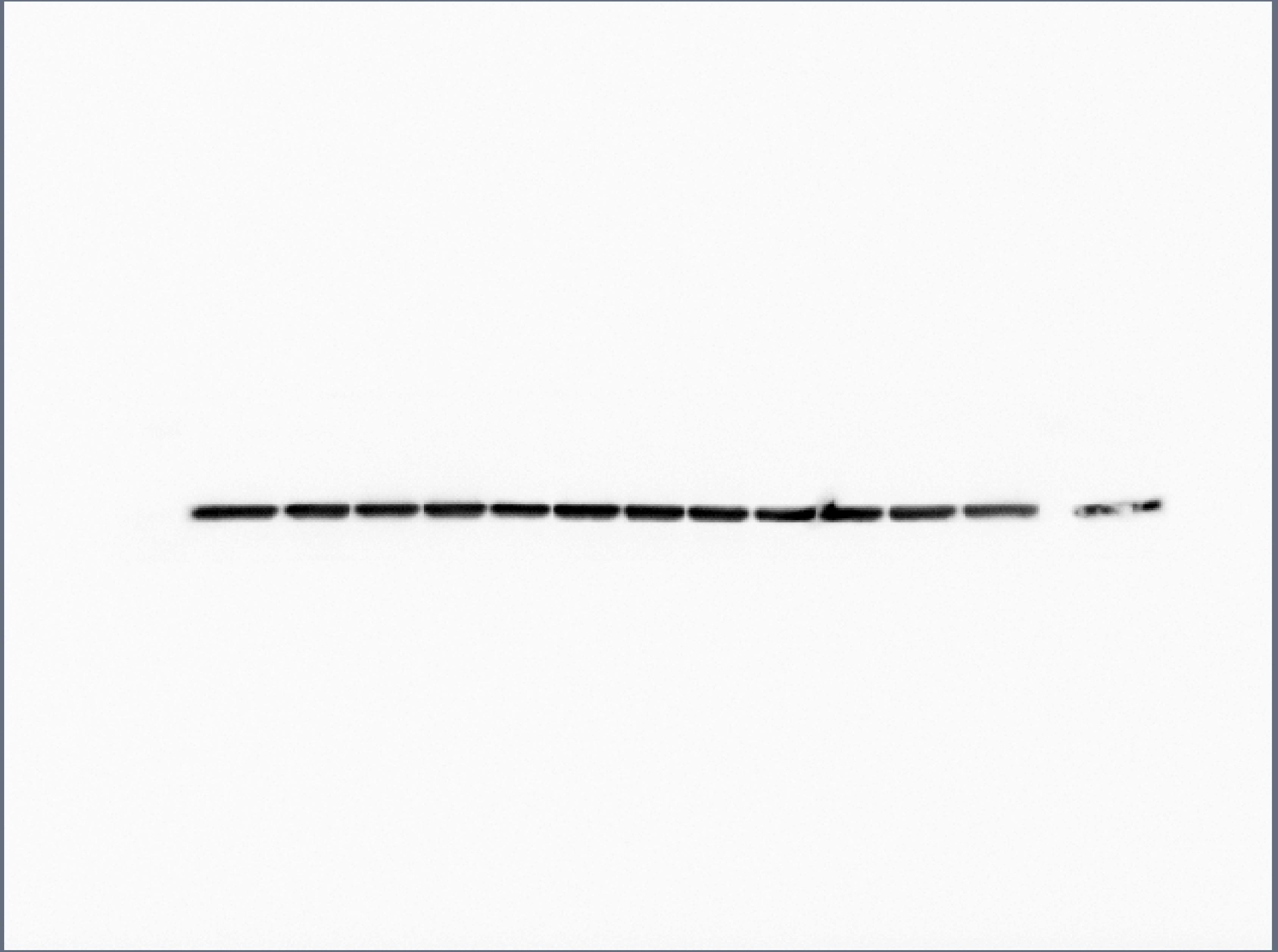

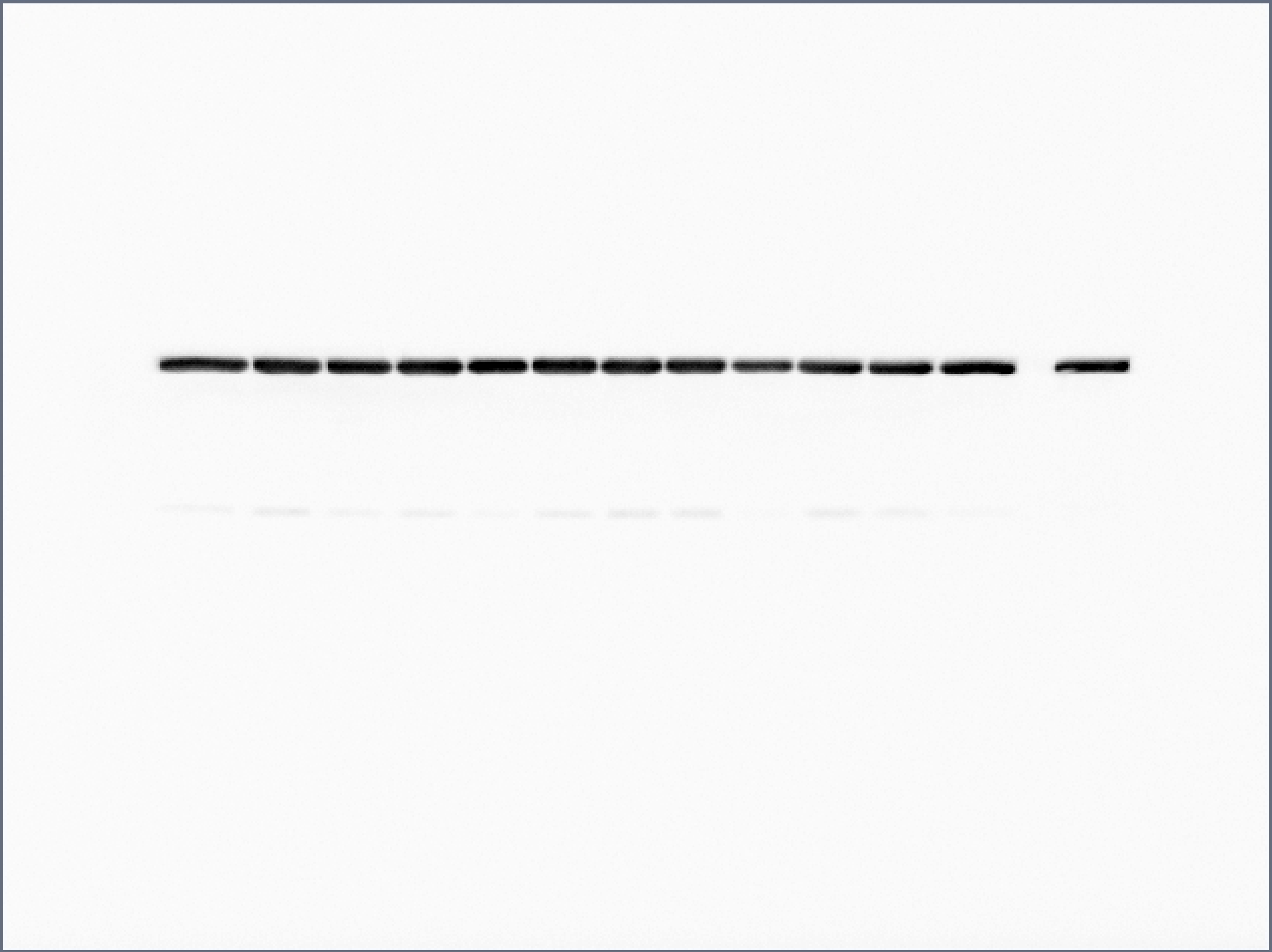

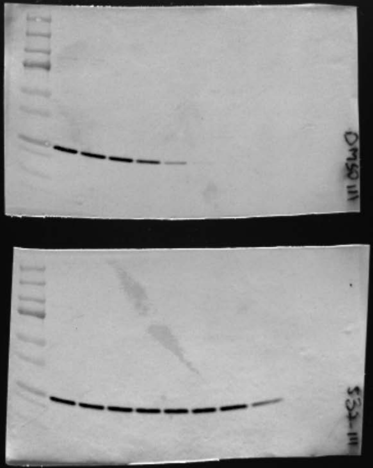
DMSO III

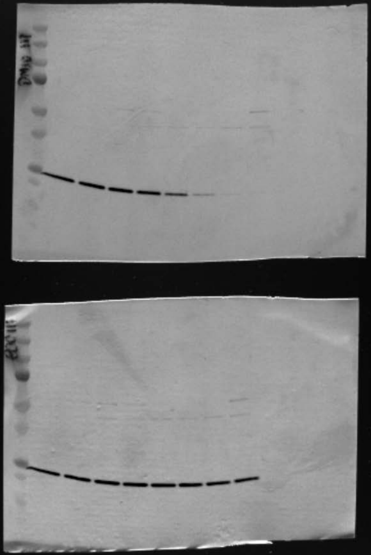

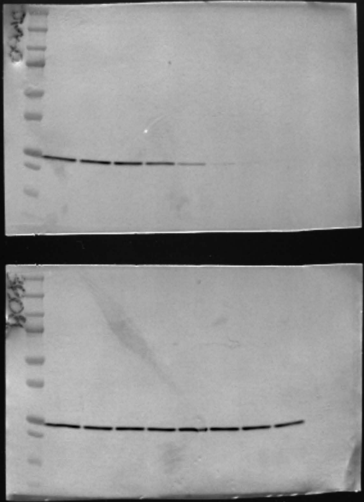

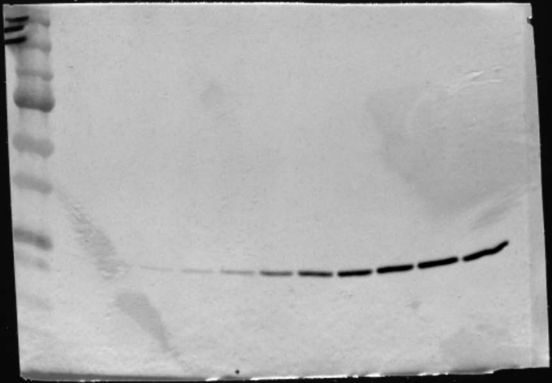

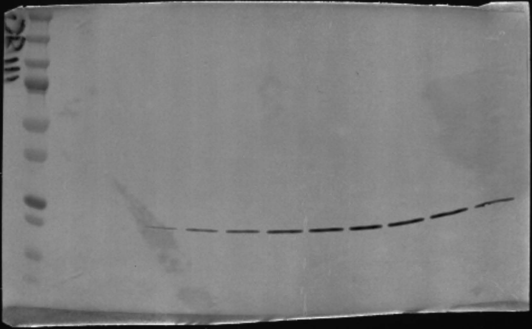

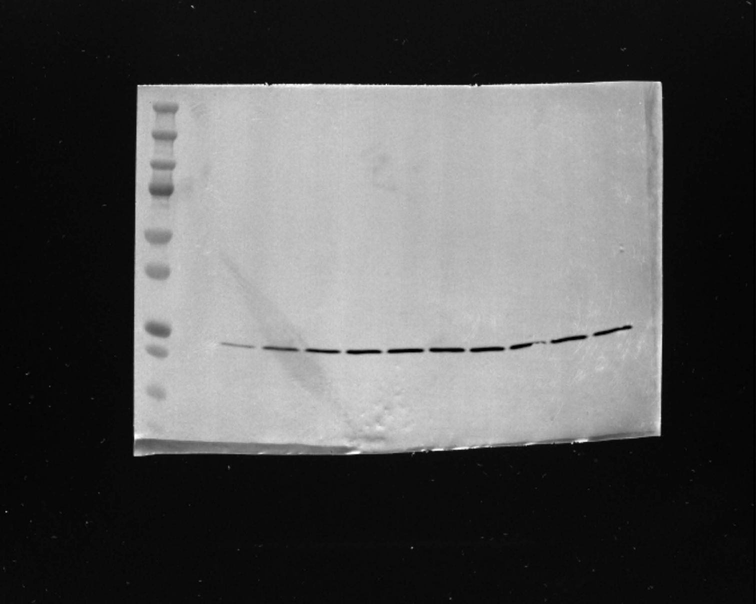

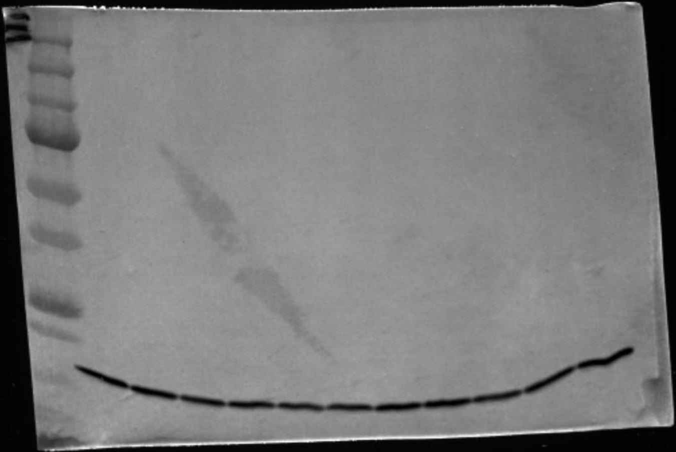

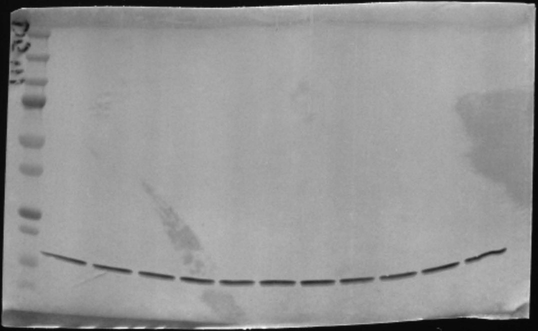

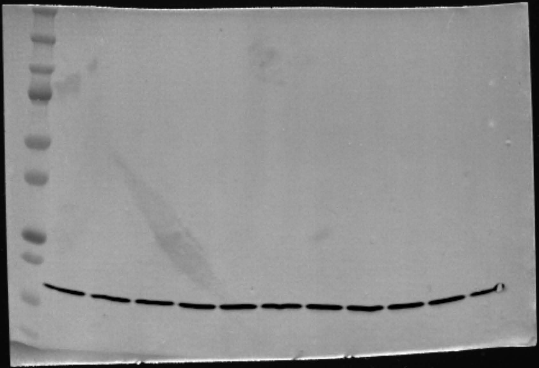

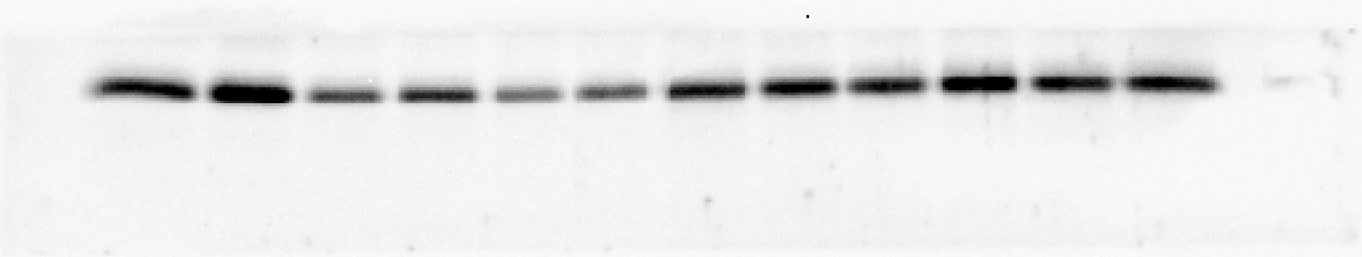

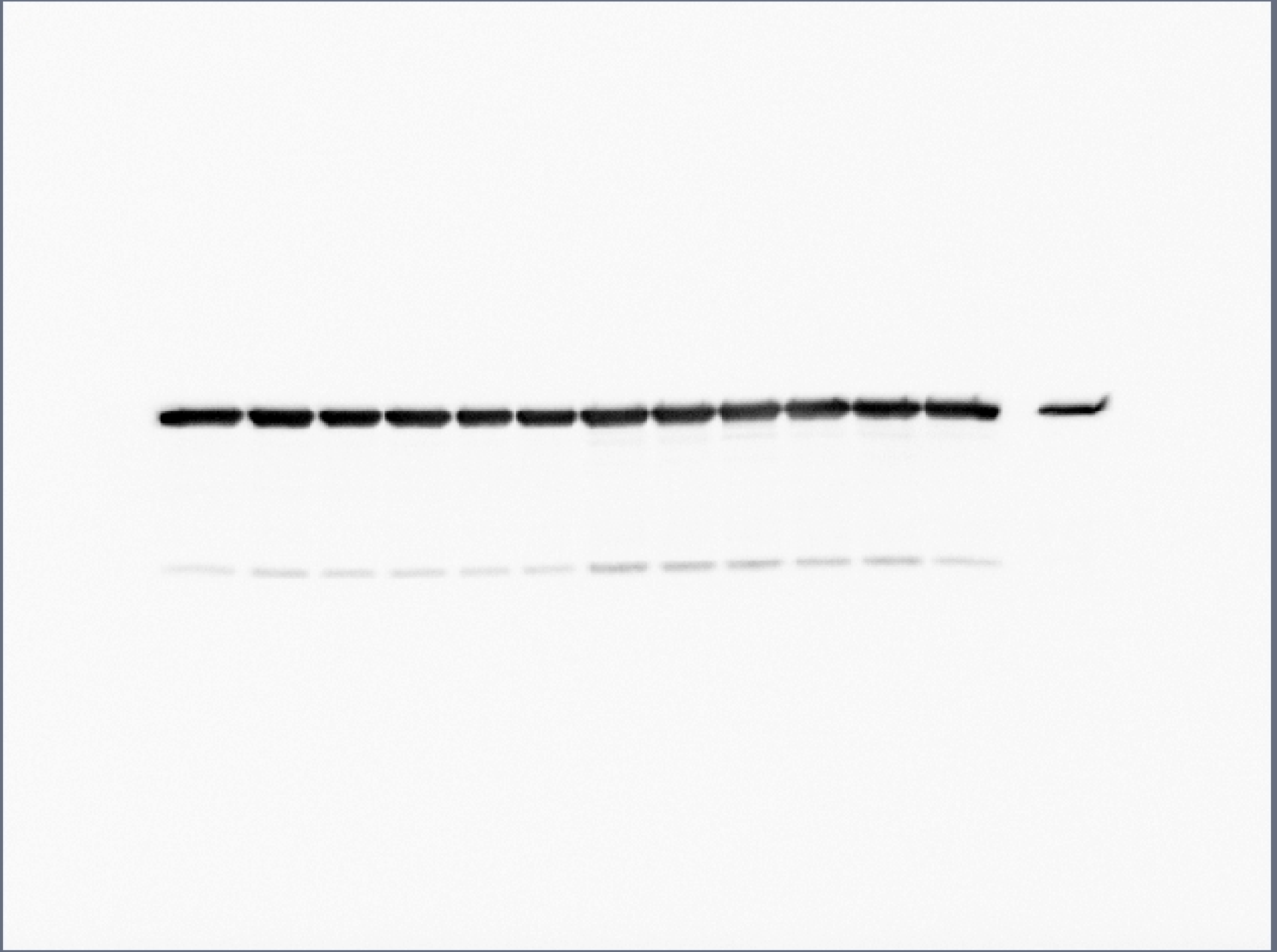

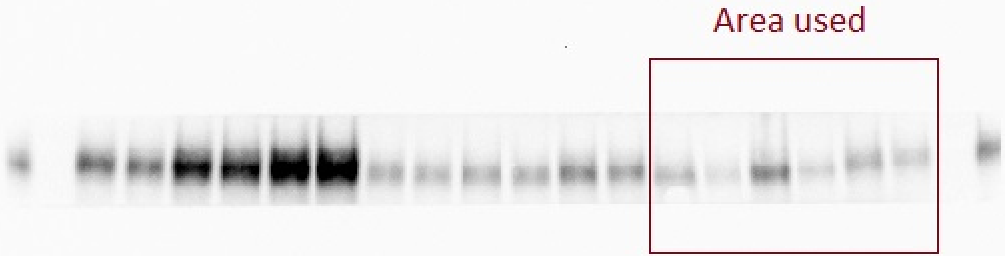
Area used

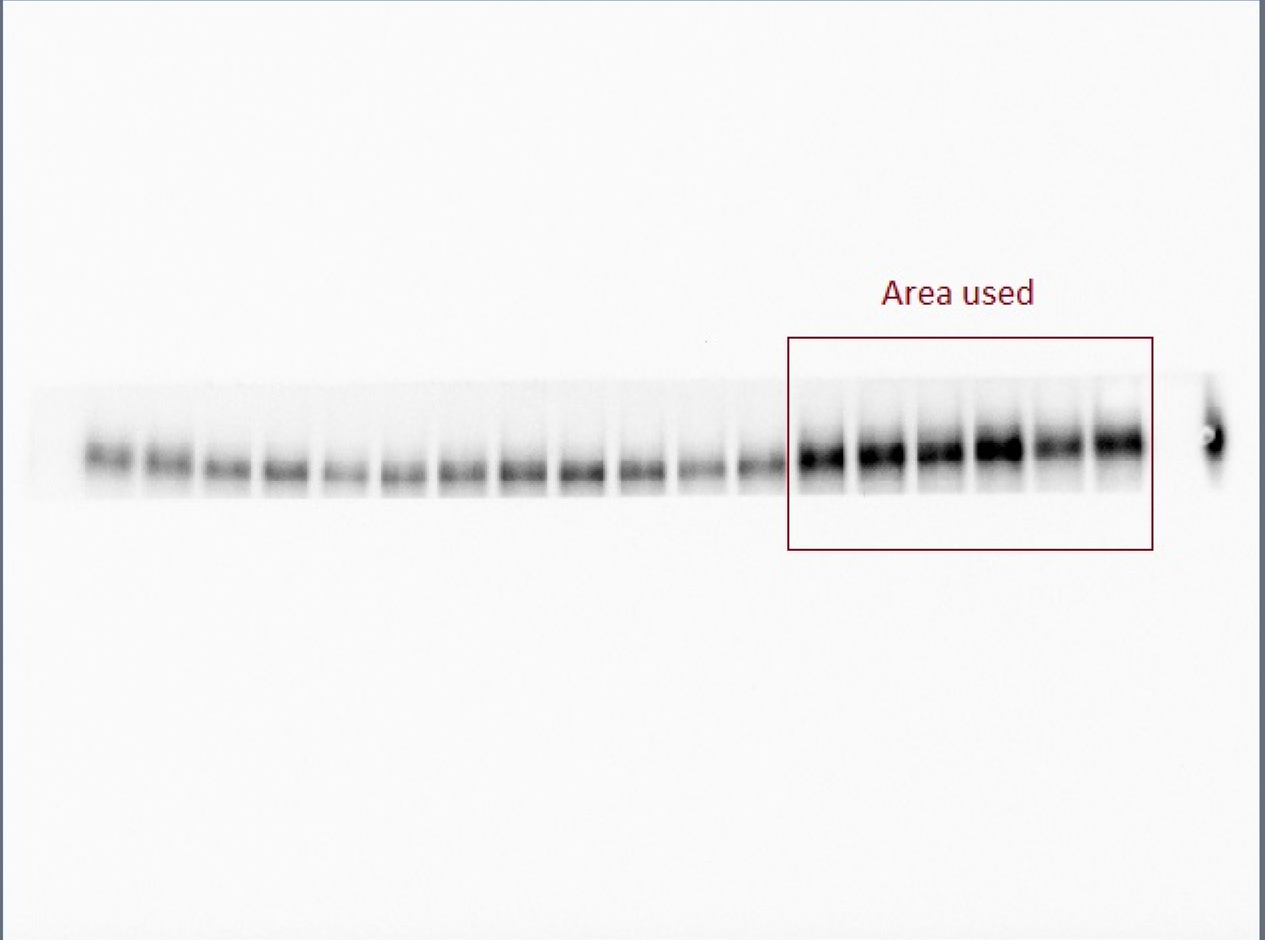
Area used

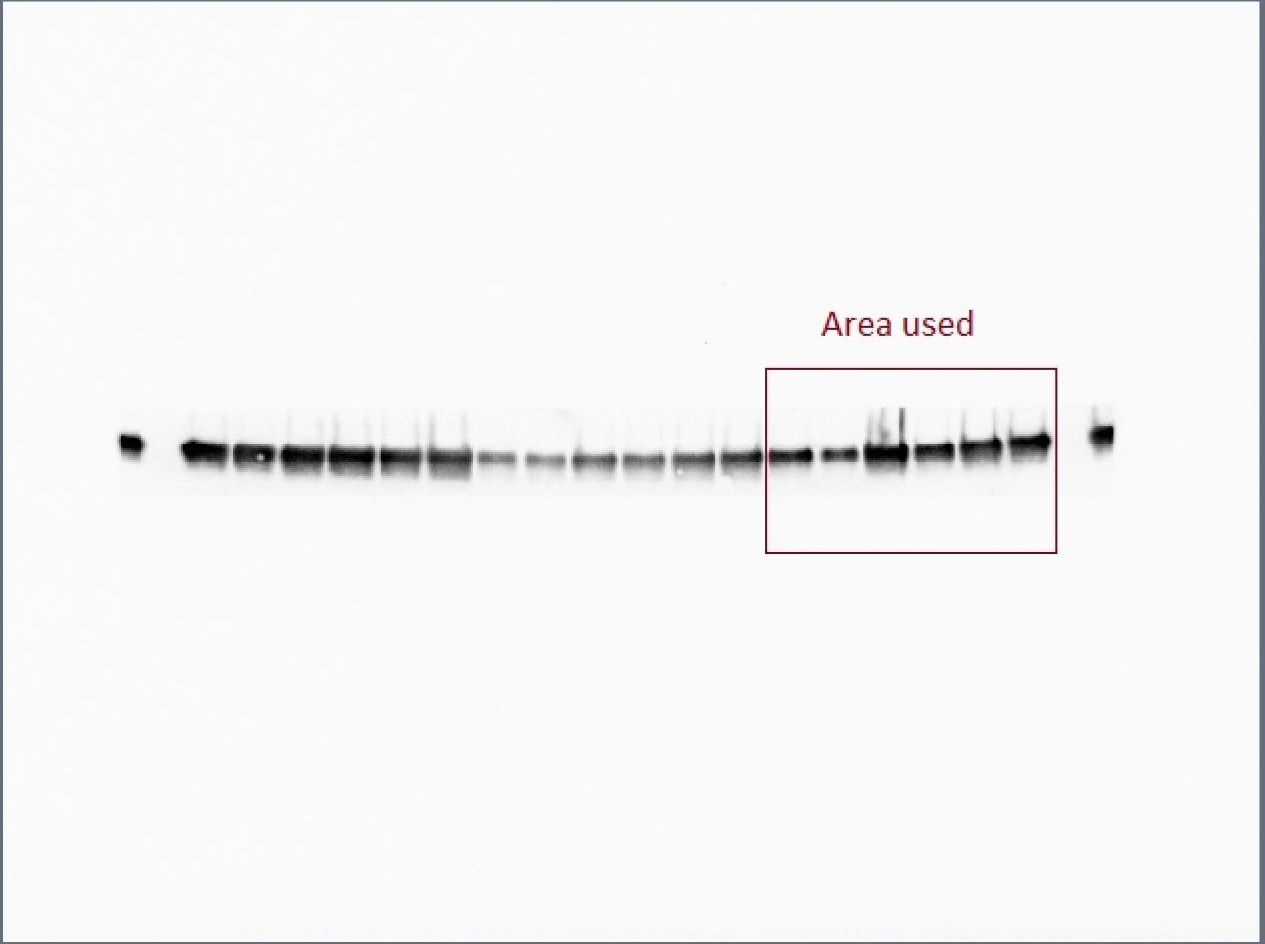
Area used

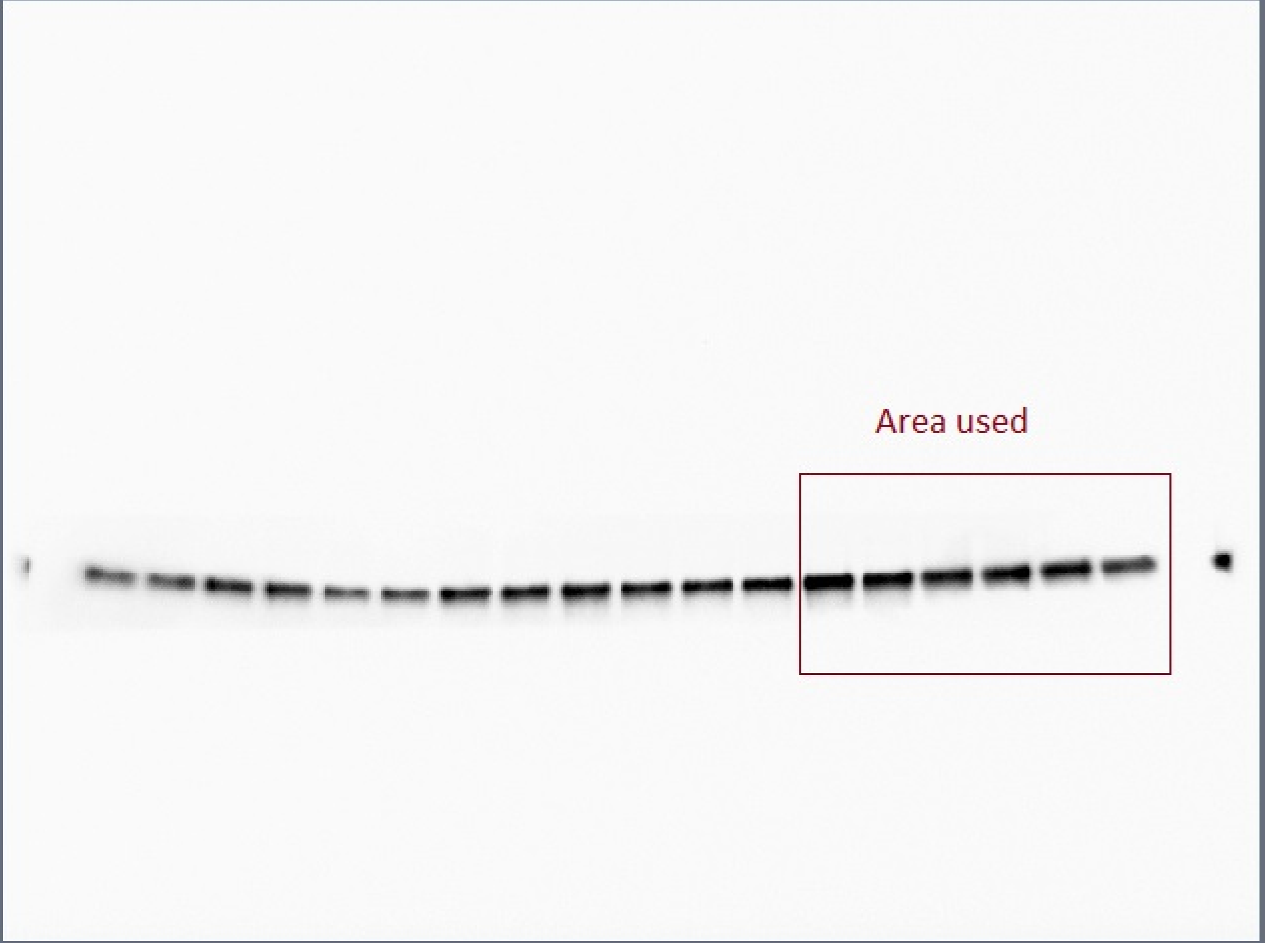
Area used

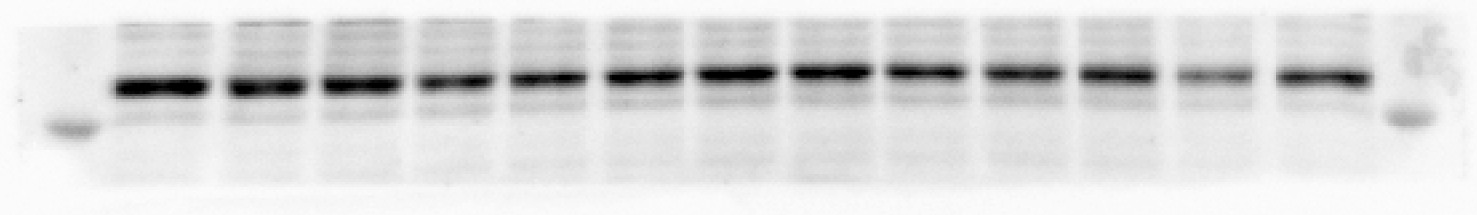

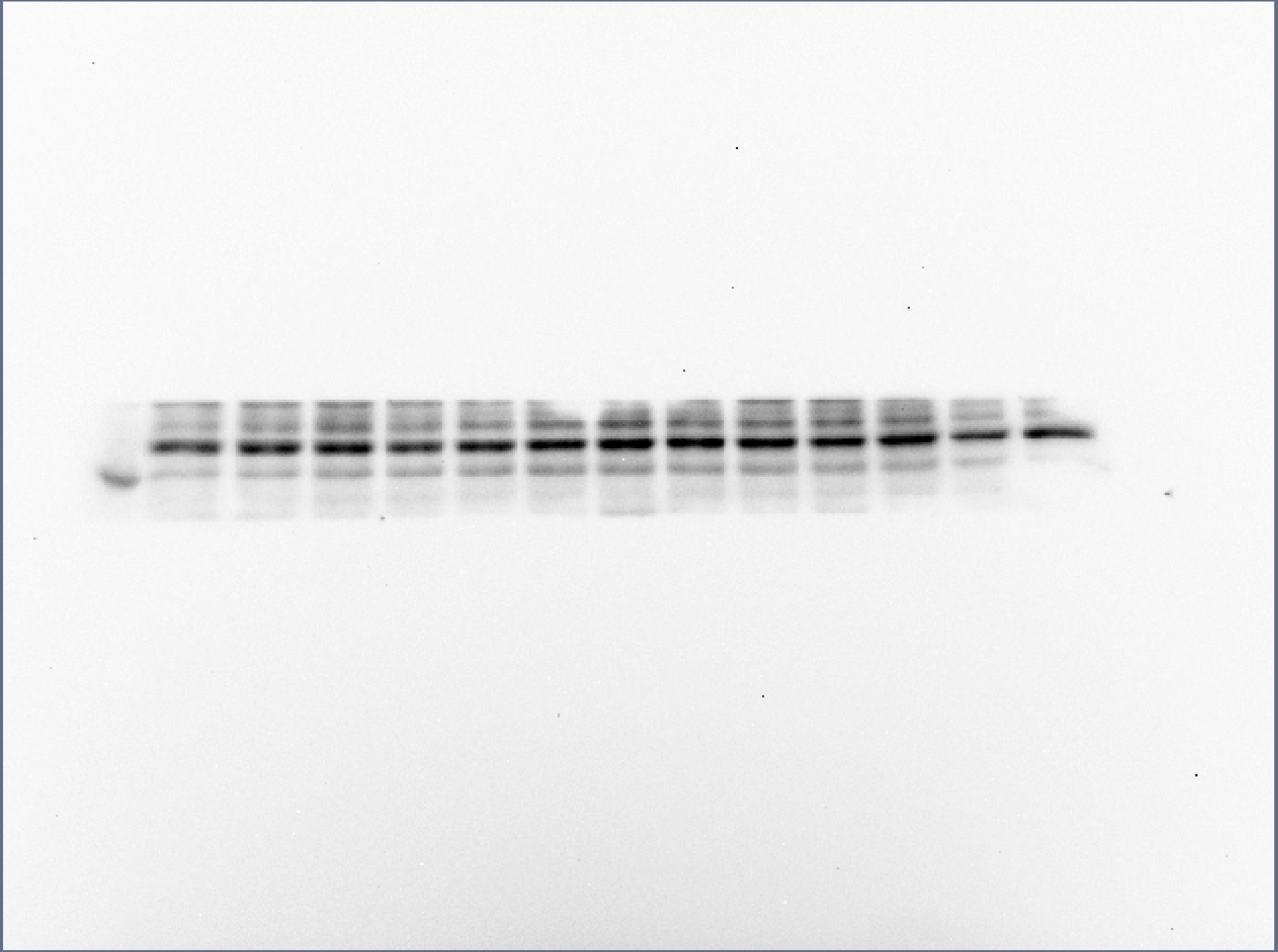

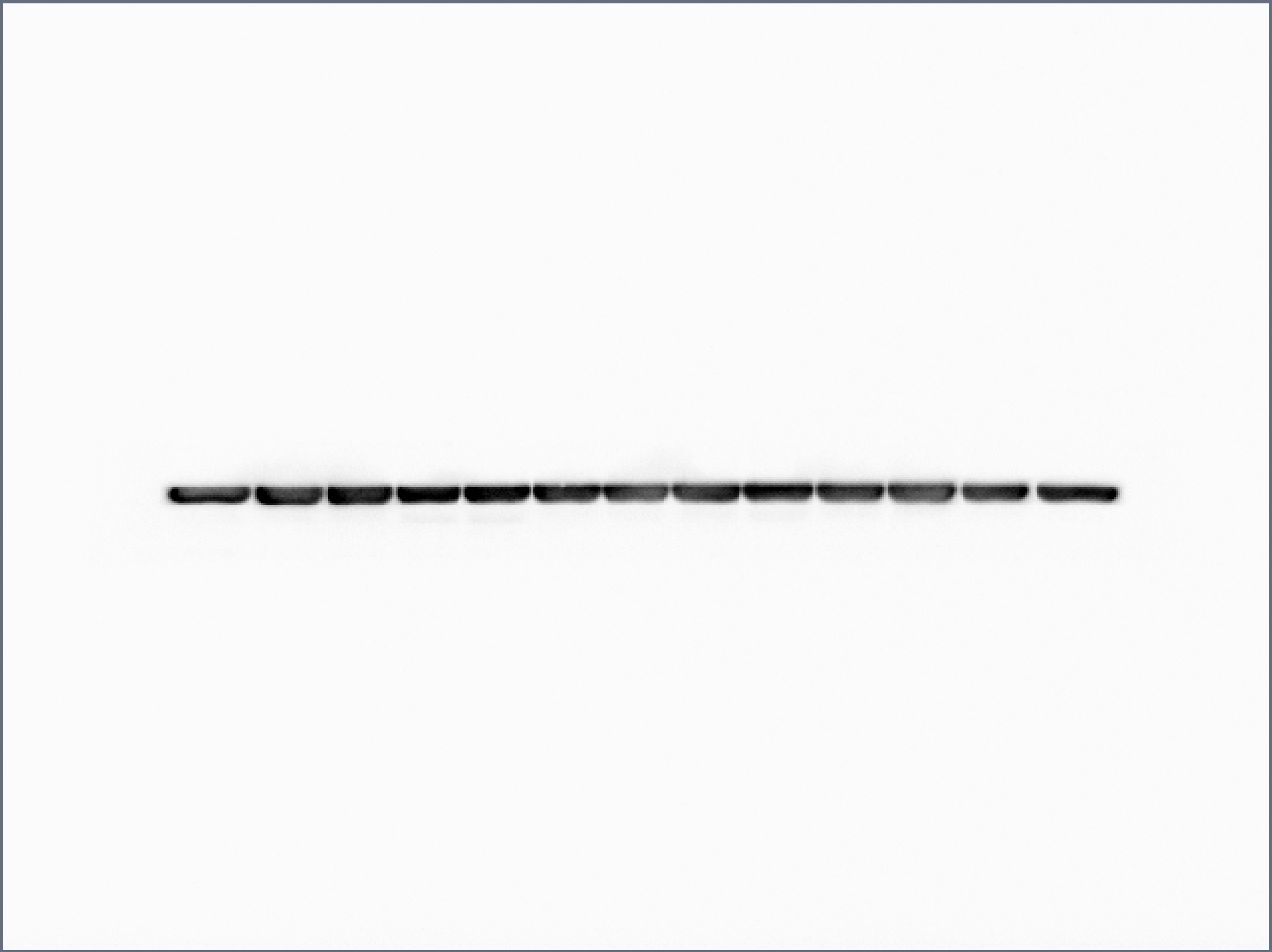

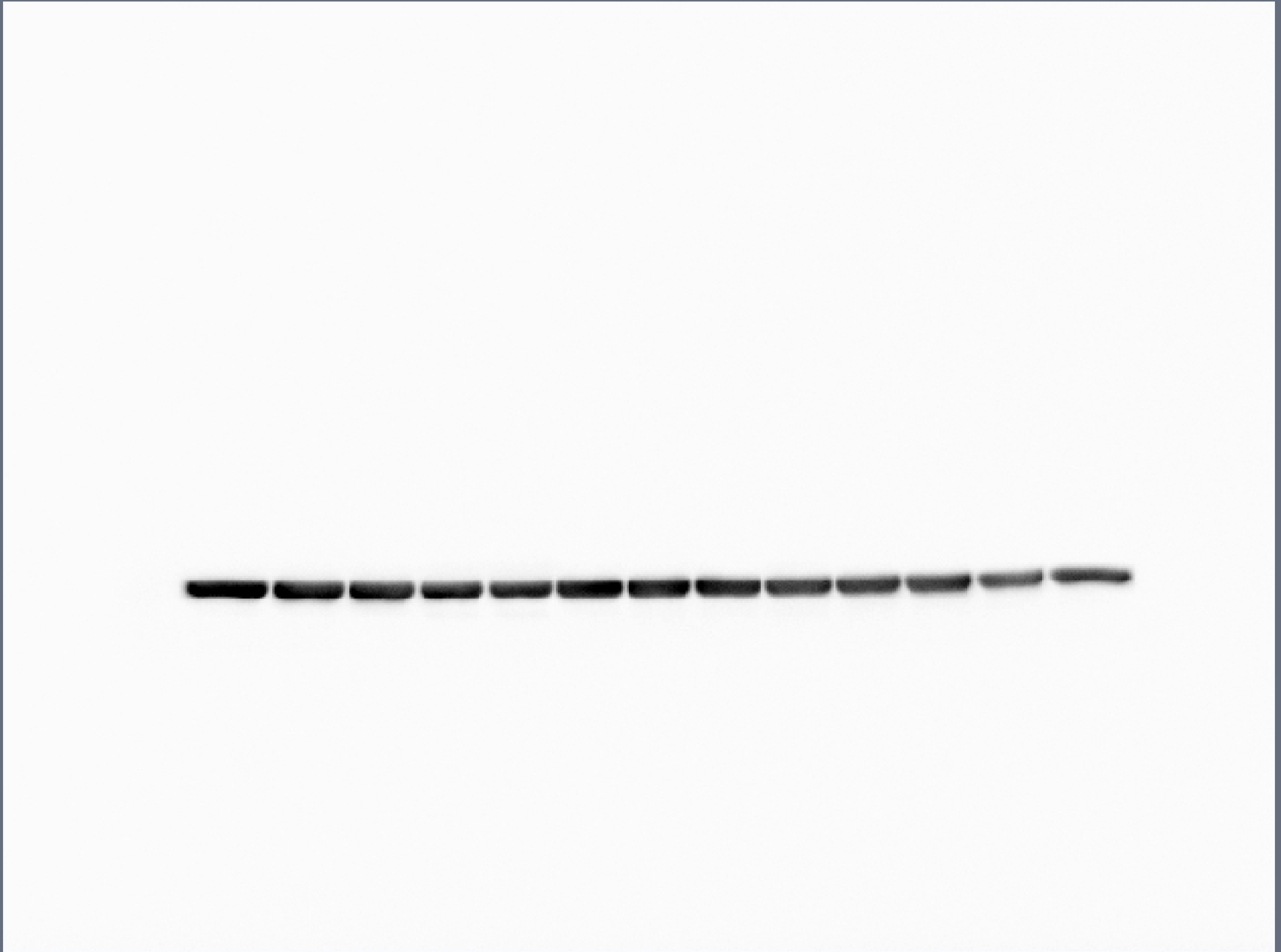

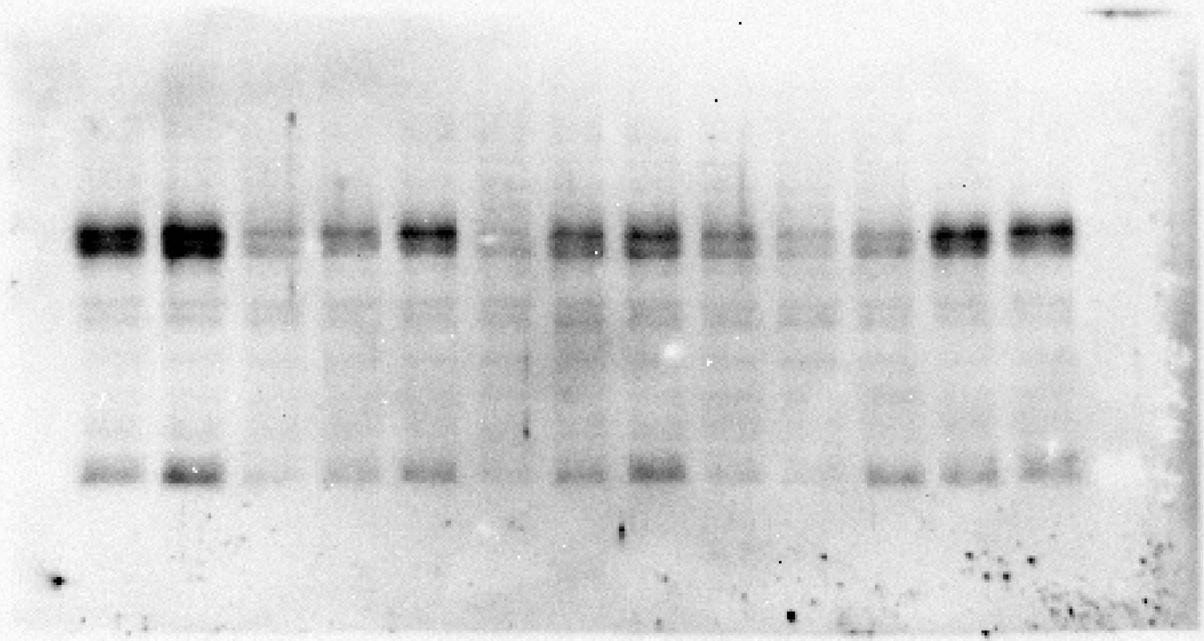

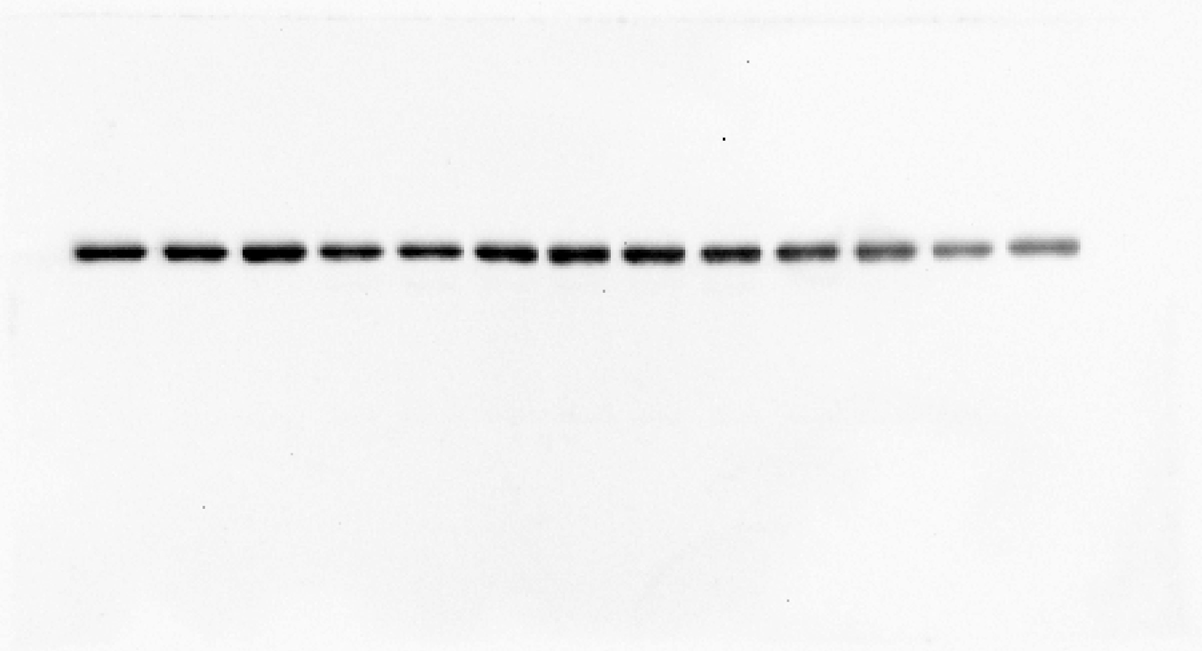

Supplement: Supplemental data [file jci-133-162120-s211.pdf]
